# Establishing the Fatty Acid Photodecarboxylase *Cv*FAP as a Platform for Photobiocatalytic Radical Transformations

**DOI:** 10.1021/acscatal.6c01333

**Published:** 2026-04-06

**Authors:** Florian Weissensteiner, Cristina Berga, Emilia Iglesias-Moncayo, Sara Salehi, Isabel Oroz-Guinea, Klaus Zangger, Ferran Feixas, Marc Garcia-Borràs, Wolfgang Kroutil, Christoph K. Winkler

**Affiliations:** † Institute of Chemistry, 27267 University of Graz, Heinrichstraße 28, Graz 8010, Austria; ‡ Institut de Química Computacional i Catàlisi and Departament de Química, 16738 Universitat de Girona, Girona 17003, Spain; § BioTechMed Graz, Graz 8010, Austria; ∥ Field of Excellence BioHealth, University of Graz, Graz 8010, Austria; # ICREA, Pg. Lluís Companys 23, 08010 Barcelona, Spain

**Keywords:** photobiocatalysis, fatty acid photodecarboxylase, isomerization, enzyme engineering, radical, C–C-coupling, cyclization

## Abstract

The fatty acid photodecarboxylase from *Chlorella variabilis* (*Cv*FAP) has emerged as a versatile photoenzyme for radical generation from readily available carboxylic acids. Here, we demonstrate that *Cv*FAP can be repurposed as a platform for diverse photobiocatalytic transformations beyond its native decarboxylation activity. Using engineered variants, including Y466A, we establish four distinct classes of reactivity: (i) the intermolecular Giese-type coupling of fatty acids with cycloalkenones, (ii) the intramolecular decarboxylative radical cyclization via nucleophilic radicals and isolated C=C bonds of the same philicity, (iii) a cysteine-mediated (*Z*) → (*E*) photoisomerization of unsaturated fatty acids, and (iv) a radical carbohydroxylation reaction that demonstrates the enzyme’s capability of olefin bis-functionalization. Directed evolution further improves the catalytic performance for intramolecular cyclization, highlighting the tunability of *Cv*FAP toward selective radical pathways. Computational analyses provide insight into substrate positioning and the origins of reactivity, supporting a model in which active-site engineering modulates competition between radical coupling and the native proton-coupled electron transfer pathway. Overall, this work establishes *Cv*FAP as a broadly applicable scaffold for non-native photobiocatalytic transformations and expands the scope of enzymatic radical chemistry.

## Graphical abstract

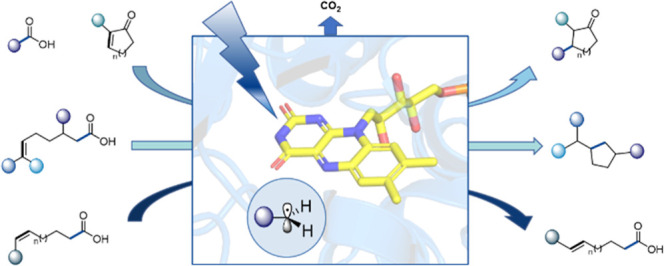

## Introduction

The increasing structural complexity and functional diversity of modern fine chemicals, together with the urgent demand for sustainable production methodologies, calls for the development of selective, efficient, and reliable alternative synthesis strategies.^1^ Biocatalysis has emerged as a powerful tool for highly selective functional group interconversions;^2,3^ however, the scope of native enzymatic C–C bond formation remains largely restricted to derivatives of the enzymes’ natural substrates.^4^ To expand the synthetic utility of biocatalysis, new generalizable methods are being developed, which enable the rapid construction of complex molecules starting from simple building blocks.^3^ In recent years, significant progress has been made in expanding the catalytic repertoire of enzymes toward new-to-nature transformations based on radical intermediates, mainly driven by detailed mechanistic understanding of the involved enzymes and advances in enzyme engineering.^5–7^ Despite their utility in forging bonds that are challenging to access by traditional two-electron mechanisms, radical reactions often suffer from poor (stereo)­selectivity and homocouplings. Enzymes, with their structurally defined active sites, offer precise control over these reactive intermediates for selective bond formation reactions.^3,6^ One approach relies on chemical photocatalysts for the external generation of free radicals, followed by selective transformations within the active site of an enzyme, mediated by cofactors such as pyridoxal phosphate (PLP)^8,9^ or thiamine.^10,11^ Distinct from these chemoenzymatic systems, a growing number of photoenzymatic processes has been reported.^7,12,13^ In these cases, natural enzymes and their variants are repurposed for new-to-nature reactions via excitation of the bound cofactors.

Examples include alcohol dehydrogenases,^17^ Baeyer–Villiger monooxygenases,^18^ or lactate monooxygenases.^19^ However, the most broadly applicable photoenzymatic platform to date is the family of ene-reductases (EREDs), promoted by the pioneering work of Hyster and co-workers, among others.^6,12,20^ These enzymes catalyze diverse reductive radical reactions under light irradiation without the need for external photocatalysts. The platforms’ reaction scope encompasses new reductions of functional groups^21^ and various selective intra- and inter-molecular coupling reactions,^22,23^ accepting diverse starting materials. To further expand the scope of enzyme-controlled radical chemistry toward new reactions and substrate classes, we sought to establish an enzyme platform beyond EREDs that is capable of promoting a large diversity of radical reactions while being tunable via enzyme engineering. Our attention is focused on the fatty acid photodecarboxylases (FAPs), in particular, the orthologue from *Chlorella variabilis* (*Cv*FAP). FAPs catalyze, as their natural reaction, the redox-neutral decarboxylation of fatty acids under blue light irradiation, using a flavin adenine dinucleotide (FAD) cofactor as a photosensitizer (Figure 1A).^14,24^

**Figure 1:**
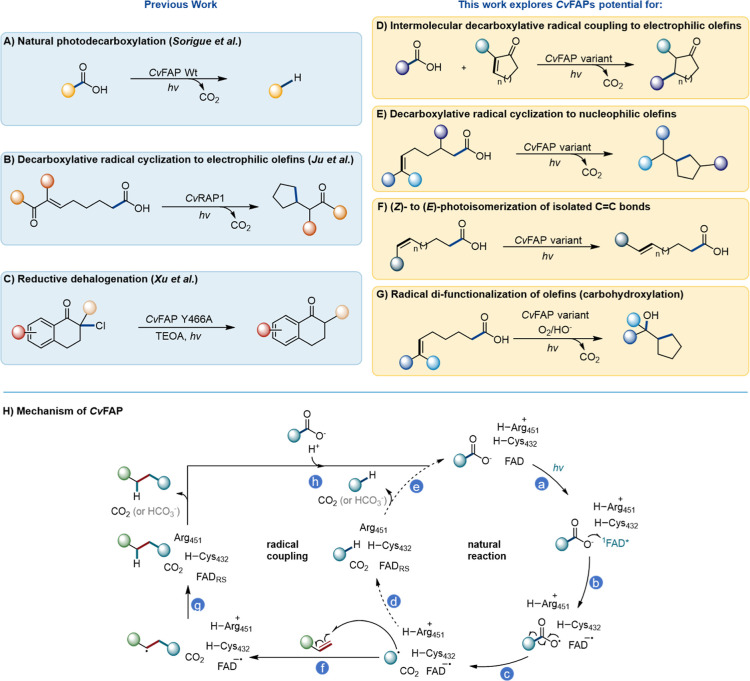
(A–G) Scope of previously reported reactions catalyzed by FAPs and the unprecedented reactions explored in this work.^14–16^ (H) Reaction mechanism of the natural decarboxylation of fatty acids catalyzed by FAPs (steps a–e) and proposed mechanism for the redirection of the central mechanistic radical intermediate toward radical coupling reactions (steps a–c and f–h). FAD = flavin adenine dinucleotide; FAD_RS_ = red-shifted FAD.

In detail, the mechanism proceeds via excitation of the FAD cofactor to its singlet excited state (^1^FAD*, Figure 1H, step a), which initiates a forward electron transfer (fET) from the bound deprotonated carboxylate substrate (step b), leading to almost immediate decarboxylation and formation of a nucleophilic alkyl radical along with a flavin semiquinone anion (FAD^•–^, step c).^14,25–27^ The next step is still under investigation, and different mechanisms have been discussed,^25,26^ but it likely proceeds via a proton-coupled electron transfer (PCET) forming the final alkane product. Computational and spectroscopic analyses identified R451 as a proton donor and FAD^•–^ as the electron source, mediated by two active-site water molecules (step d).^25^ As the photodecarboxylation activity is significantly reduced^25^ (or completely lost^26^) when a conserved cysteine (C432) is exchanged for a serine, this residue is most likely also involved in this step. Finally, product release and binding of a new substrate complete the catalytic cycle (step e). We hypothesized that this highly reactive, nucleophilic radical could serve as a central intermediate for new radical coupling reactions if suitable (electrophilic) radical acceptors are provided (step f). This would enable inter-molecular or intra-molecular radical bond formations analogous to classical Giese-type additions but now occurring under enzymatic control. Subsequently, the resulting radical may undergo a PCET event similar to the natural mechanism (step g), closing the alternative catalytic cycle.

While our work was ongoing, a conceptually related idea was reported by Yang and co-workers, demonstrating an intramolecular radical cyclization using FAPs.^15^ Specifically, the authors showed a cyclization reaction via the coupling of the intermediary nucleophilic radicals with electrophilic alkenes bearing an electron-withdrawing group, confirming the mechanistic hypothesis that the fleeting radical intermediate can be redirected to engage with radical acceptors (Figure 1B). A second report by Xu et al. further underscored the potential of *Cv*FAP for promiscuity by applying it for the reductive dehalogenation of halogenated tetralones (Figure 1C), demonstrating the enzymes’ capability to also catalyze reductive reactions, distinct from their natural redox-neutral activity.^16^ Together, these activities already hint at the FAP family’s unexplored potential as a platform for radical biocatalysis.

Herein, we report our efforts to repurpose *Cv*FAP and engineered variants as an enzymatic platform for diverse biocatalytic new-to-nature reactions utilizing radicals. We demonstrate the first intermolecular decarboxylative Giese-type coupling between nucleophilic free radicals formed from fatty acids and electrophilic cycloalkenones under FAP catalysis (Figure 1D). Furthermore, we show that FAPs can promote intramolecular C–C bond formation with isolated, nucleophilic olefins, which is mechanistically distinct from the electrophile-directed cyclization reported by the Yang group (Figure 1E).^15^ This reaction also serves as a model to illustrate the evolvability of *Cv*FAP, tailoring the enzyme to favor coupling over the natural pathway. In the course of our investigations, we identified two additional promiscuous activities of *Cv*FAP variants: a light-driven (*Z*) → (*E*) photoisomerization of isolated C=C bonds in fatty acid substrates (Figure 1F), and an oxygen-dependent carbohydroxylation of alkenes (Figure 1G).

While the primary aim of this study was to demonstrate the scope of reactivity that is accessible with fatty acid photodecarboxylases, the identified transformations are relevant from a synthetic perspective. Intermolecular Giese-type couplings from carboxylic acids enable the direct utilization of abundant and renewable feedstocks without the need for prior activation. The reported intramolecular cyclization expands the scope of biocatalytic radical C–C bond formations by enabling reactions between radical intermediates and acceptors of the same philicity. While the observed photoisomerization provides insight into the enzyme’s reaction mechanism, the carbohydroxylation reaction offers a potentially valuable strategy for generating molecular complexity from simple carboxylic acid precursors by forming two bonds in a single step.

Together, these four reactivities, along with an enzyme engineering campaign, highlight the broad catalytic potential of the FAP family and provide a foundation for the future reaction expansion of this emerging class of photoenzymes.

## Results

### Intermolecular Decarboxylative Radical Coupling with Electrophilic C=C Bonds

While decarboxylative photobiocatalytic Giese-type 1,4-additions have already been reported using EREDs, stereoselectivity was achieved only at the final hydrogen atom transfer (HAT) step, but not during the formation of the new C–C bond.^28^ In contrast, the reported decarboxylative cyclization catalyzed by *Cv*FAP variants could be engineered to high enantioselectivity.^15^ Therefore, we began our exploration of the scope of *Cv*FAP’s radical reactivity by challenging it with catalyzing the intermolecular decarboxylative radical coupling between a fatty acid and activated enones as electrophilic radical acceptors (Figure 2A).

**Figure 2:**
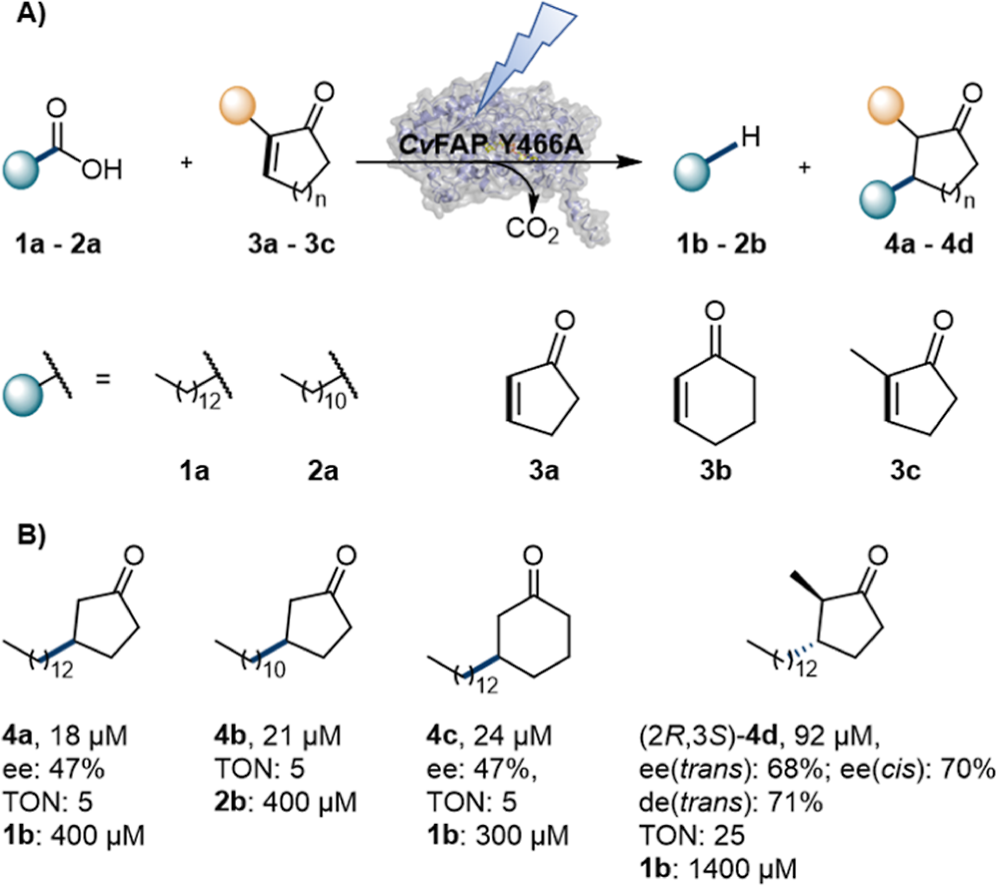
Photobiocatalytic intermolecular Giese-type 1,4-additions of fatty acids onto activated cycloalkenones. (A) Reaction scheme. (B) Product scope (**4a**–**4d**). Reaction conditions: *Cv*FAP variant Y466A (lyophilized CFE, 20 mg/mL, corresponding to 3.7 nmol of *Cv*FAP Y466A), fatty acid (**1a**–**2a**, 10 mM), enone (**3a**–**3c**, 20 mM), 30% v/v DMSO (degassed), in Tris·HCl buffer (100 mM, pH 8.5, degassed) with a final volume of 1 mL, anaerobic (nitrogen atmosphere); illumination in a custom photoreactor (blue LEDs, 455 nm, 36 μE/L),^29^ 16 h at 25 °C and 500 rpm; triplicates.

Previous studies have shown that *Cv*FAP can simultaneously bind two molecules, namely, the carboxylic acid substrate and a decoy alkane (C7–C17).^30^ However, while alkanes readily fit into the narrow substrate-binding tunnel of the wild-type enzyme, the binding of an additional electrophilic radical acceptor likely requires more space. To address this, we chose the Y466A variant of *Cv*FAP for initial tests, as it has a significantly enlarged active site, resulting in an expanded substrate scope.^31^ We anticipated that this mutation would create the required space to accommodate a coupling partner bearing an electrophilic (activated) C=C bond. Testing this hypothesis using myristic acid (**1a**) as a fatty acid radical precursor and cyclopent-2-enone (**3a**) as a radical acceptor, we were pleased to observe the formation of 18 ± 1 μM of the intermolecular coupling product **4a** with an ee of 47 ± 3%, alongside 0.4 mM of tridecane (**1b**), the product of the native reaction (Figure 2B). Control experiments using only the cofactor FAD (1 mol %), *E. coli* BL21 (DE3) cells without harboring an empty expression vector, or in the absence of light or enzyme showed no formation of **4a**, confirming the finding of a new photobiocatalytic coupling reaction.

The obtained enantioselectivity strongly suggests that the C–C bond formation occurred within the chiral environment of the enzyme’s active site, especially considering that previous intermolecular photobiocatalytic Giese-type couplings showed no stereoselectivity for the newly formed bond.^28^ Importantly, no coupling product was observed with wild-type *Cv*FAP, which produced only **1b**. This confirms our hypothesis that the enlarged active site of *Cv*FAP Y466A is necessary for binding the two substrates and enabling the coupling reaction. Interestingly, a substrate control reaction including only **1a** (no **3a**), showed a 3-fold increase in the formation of the natural product **1b** (1.2 ± 0.1 mM). This observation suggests that the presence of ketone **3a** may lead to enzyme inhibition or inactivation, either directly by the ketone or via reactive radical intermediates formed during the reaction. Moving to the shorter fatty acid **2a** as a radical precursor resulted in the same reactivity, affording 21 ± 1 μM of the desired coupling product **4b**. Likewise, cyclohex-2-enone (**3b**) was accepted as the coupling partner, yielding 24 ± 1 μM of **4c** with an ee of 47 ± 3%. Interestingly, when using **1a** with enone **3c**, which bears a methyl substituent in the α-position, we observed a 5-fold increase in the formation of the intermolecular adduct, yielding 92 ± 2 μM **4d,** which equals 25 total turnovers to the coupling product. Stereochemical analysis revealed a diastereomeric excess de­(*trans*/*cis*) of 71 ± 1 in favor of the *trans*-isomer (2*R*,3*S*), with ee­(*trans*) = 68 ± 1% and ee­(*cis*) = 70 ± 6%. Moreover, the formation of the natural linear product (**1b**) increased four times (1.4 ± 0.0 mM), to a level comparable to either the control reaction using **1a** without enone or the reactivity of the wild-type enzyme. This indicates that the previously observed enzyme inhibition or inactivation with **3a** or **3b** does not occur to a significant extent in the presence of **3c** (see Table S17 for complete control data).

### Decarboxylative Radical Cyclization with Nucleophilic C=C Bonds

Most photobiocatalytic radical coupling reactions pair radicals and radical acceptors according to their philicity to maximize reactivity.^8,10,22^ A previously reported example of radical cyclization reaction catalyzed by *Cv*FAP variants utilizes the nucleophilic radical intermediate to couple with an electrophilic radical acceptor.^15^ In principle, however, such nucleophilic radical species can also attack nucleophilic C=C bonds, although at slower rates.^32^ We therefore interrogated whether *Cv*FAP is capable of catalyzing such thermodynamically more demanding cyclizations (Figure 3A). We first started with the *Cv*FAP Y466A variant, possessing an enlarged active site, and screened a substrate panel comprising carboxylic acids of varying chain lengths (C_7_–C_14_), bearing either internal (**8a**–**10a**) or terminal C=C bonds (**5a**–**7a**) in positions 5, 6, 7, and 8 for radical cyclization. According to Baldwin’s empirical rules,^33^ exo-trig attack yielding 5–7 membered rings, and endo-trig attack leading to 6–8 membered products, are preferred. The Beckwith rules state that the formation of 5-exo products is kinetically favored.^34^

**Figure 3:**
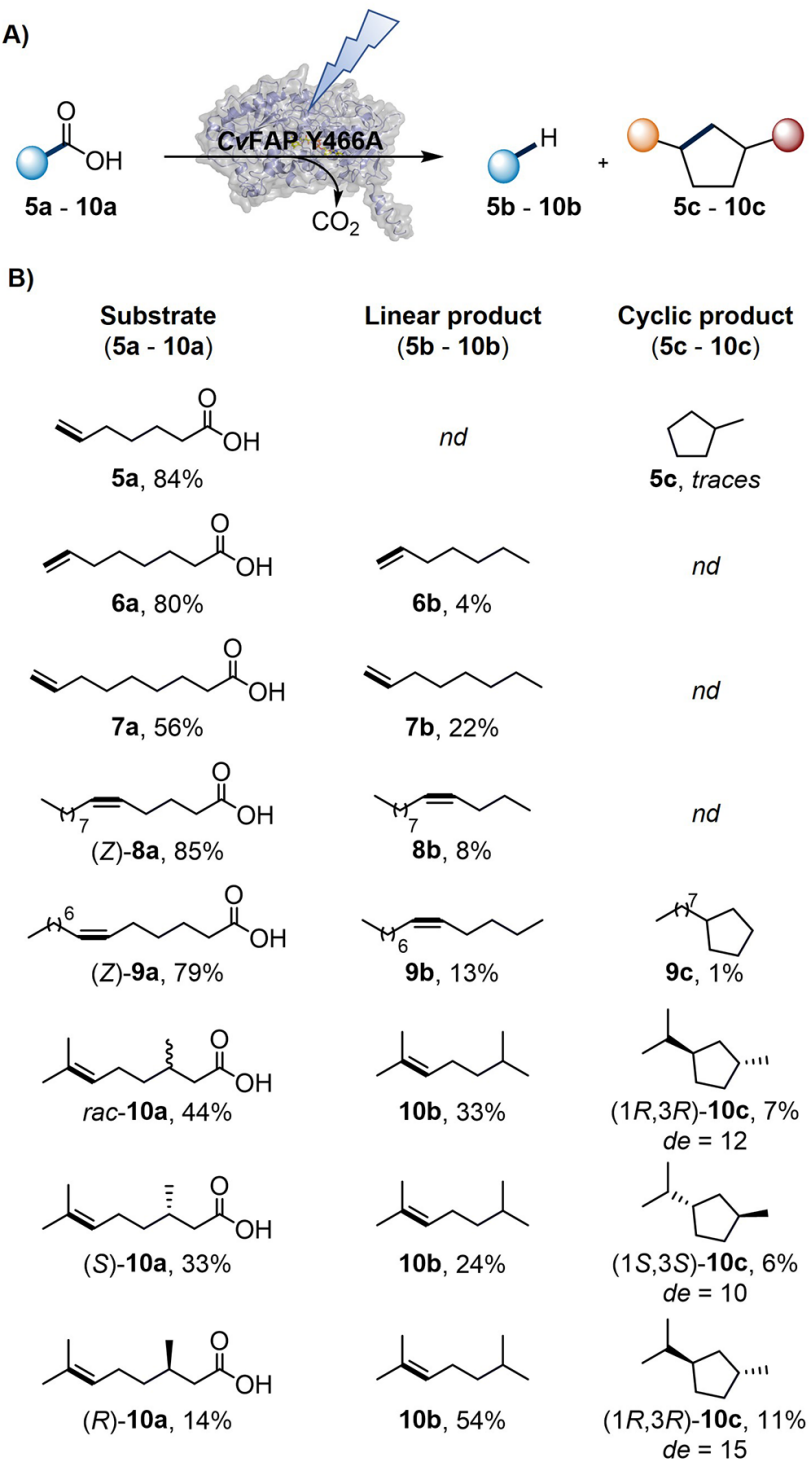
Photobiocatalytic decarboxylative radical cyclization of carboxylic acids bearing nucleophilic C=C bonds. (A) General reaction scheme. (B) Substrate scope. Reaction conditions: *Cv*FAP Y466A (lyophilized CFE, 20 mg/mL, corresponding to 3.7 nmol of *Cv*FAP Y466A), carboxylic acid (**5a**–**10a**, 10 mM), 30% v/v DMSO, in Tris·HCl buffer (100 mM, pH 8.5) with a final volume of 1 mL, aerobic; illumination in a custom photoreactor (blue LEDs, 455 nm, 36 μE/L),^29^ 16 h at 25 °C and 500 rpm; triplicates.

Cyclization activity was found for several of the tested substrates (Figure 3B). Starting from **5a**, traces of cyclopentane analogue **5c** were detected. This low amount of product formation might be explained by product-loss due to the high volatility of **5c**, or by the fact that the final PCET of the native reaction (Figure 1H, step d; ∼10^–7^ s)^25^ simply outcompetes the radical attack on the terminal C=C bond (estimated cyclization time based on radical clock data: ∼10^–5^ s).^32^ In contrast, substrates (*Z*)-**8a**–**9a**, bearing internal C=C bonds at position 5, and 6, respectively, would generate more stable secondary radical intermediates upon either exo-*trig*, or endo-trig attack, which facilitates cyclization rates (estimated cyclization time: ∼10^–6^ s).^32^

For substrate (*Z*)-**8a,** neither the 4-exo nor 5-endo cyclized products were detected, while in the case of (*Z*)-**9a,** the 5-exo-trig product **9c** was formed in up to 0.1 mM (27 turnovers), next to 1.3 mM (351 turnovers) of the natural linear product **9b**.

Intriguingly, in addition to decarboxylation and cyclization, we observed a minor, enzyme-dependent photoisomerization of substrates (*Z*)-**8a** and (*Z*)-**9a** to their thermodynamically more stable (*E*)-isomers. As the mechanism of this photoisomerization was unclear, we investigated it further (vide infra). Encouraged by these results, we next tested citronellic acid (**10a**), a substrate featuring a disubstituted C=C bond.

This substrate would generate an even more stable tertiary radical intermediate upon exo-attack of the C=C bond, potentially accelerating the cyclization reaction even further. In addition, it offered an opportunity to probe both enantiomeric discrimination at the preexisting stereocenter and stereocontrol over the newly formed bond. Comparing biotransformations of enantiopure (*R*)-**10a** and (*S*)-**10a** with *rac*-**10a** clearly showed a preference of *Cv*FAP Y466A for the (*R*)-enantiomer, yielding approximately two times more linear product and up to 1.1 mM (300 turnovers) of the carbocycle **10c** (5-exo-trig). Surprisingly, only a modest *de* of 10% in favor of the *trans*-configured product (1*S*,3*S*-**10c**) was observed starting from (*S*)-**10a** and a *de* of 15% for (1*R*,3*R*)-**10c** starting from (*R*)-**10a**.

Control experiments using (*R*)-**10a** confirmed that the formation of a cyclic product requires the presence of both *Cv*FAP Y466A and light. Furthermore, no **10c** was detected using only FAD, and only traces (<0.1 mM **10c**) were found when using wild type *Cv*FAP, which instead predominantly generated the linear product (up to 4.8 mM, see Table S18 for all control experiments).

These reactivity trends are in line with the estimated stabilities of the radical intermediates calculated using density functional theory (DFT) calculations (see Table S3 for details). Substrates with high barriers for the cyclization of the radical intermediate (Δ*G* ^‡^ ≥ 15 kcal/mol) and poorly stabilized cyclized radicals (ΔΔ*H* ≈ 0–12 kcal/mol, e.g., **7a** and **8a**) showed no detectable cyclization, forming only linear products. Substrates **5a** and **9a**, with moderate barriers (Δ*G* ^‡^ ≈ 8–10 kcal/mol) and limited radical stabilization, afforded only trace cyclic products. In contrast, substrate **10a** displayed a low cyclization barrier and strongly stabilized the radical intermediate, consistent with higher experimental conversion to the cyclic product.

In order to explore the feasibility of the proposed mechanism involving substrate (*R*)-**10a**, we carried out DFT calculations using a model system that includes the flavin aromatic core, the substrate, and the R451 guanidino group (see Figure 4 and Section 4.1 in the Supporting Information for all computational details), indicating that, upon singlet photoexcitation of the complex system (substrate-cofactor model complex **M-(R)-10a**) and induced electron transfer from substrate **10a** to the FAD cofactor, an open-shell singlet radical pair (**10a** ^•^ + FAD^•–^, **M-(R)-10a_OSS_**) is easily formed. This enables an almost barrierless decarboxylation (**TS1**_**OSS**_, Δ*E* ^‡^ = 0.7 kcal·mol^–1^), generating the mechanistically relevant reactive primary C-terminal radical (**M-Int1_T_** / **M-Int1**_**OSS**_). **M-Int1** can further react following the native PCET pathway toward the decarboxylated linear product in a kinetically and thermodynamically (Δ*E* = −36.6 kcal·mol^–1^) favored process. Alternatively, the linear radical intermediate can undergo a radical exo-trig-attack to the double bond to form a 5-membered ring radical, which then undergoes a final spontaneous PCET. Calculations show that the activation barriers for the exo-trig-attacks leading to the different possible enantiomers are low in energy and energetically accessible (**TS3**_**M‑(R,R)**_ **TS3**_**M‑(R,S)**_, Δ*E* ^‡^ = 5–6.9 kcal·mol^–1^), with no clear enantiopreference in the absence of the protein chiral active site environment.

**Figure 4:**
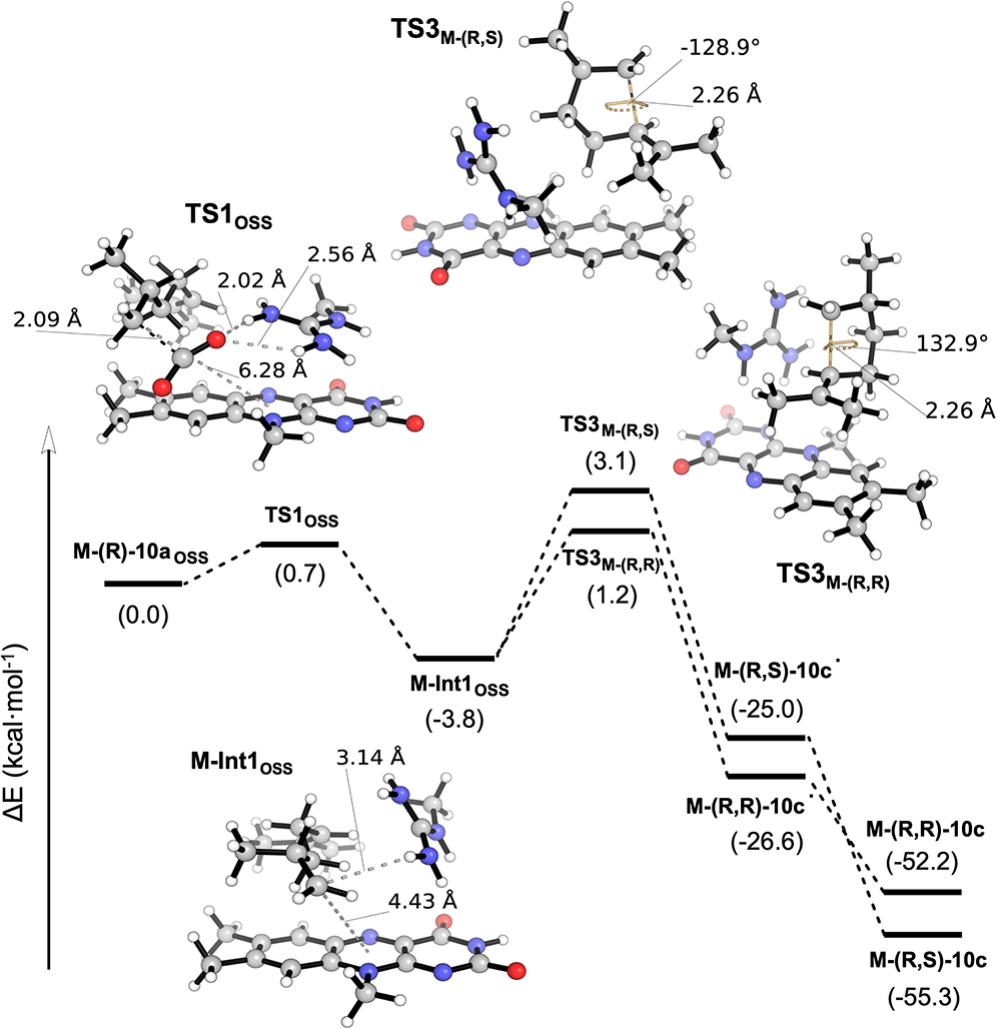
DFT-calculated reaction energy profile for the proposed intramolecular decarboxylative radical cyclization pathway, obtained from a truncated model system that includes the flavin aromatic core, substrate (*R*)-**10a,** and the R451 guanidino group. Optimized geometries of key transition states and intermediates, leading to (1*R*,3*R*)-**10c and** (1S,3*R*)-**10c**, are shown. Electronic energy values are given in kcal·mol^–1^. Key distances and angles are given in Angstrom (Å) and degrees (°), respectively. For details, see Section 4.1 in the Supporting Information.

Based on these intrinsic mechanistic preferences, it is hypothesized that the *Cv*FAP Y466A active site is able to promote cyclization by a geometric preorganization of the linear substrate that favors C–C bond formation while preventing the native PCET of the linear radical intermediate. This hypothesis is interrogated in the next sections.

### (*Z*) → (*E*) Photoisomerization of Isolated C=C Bonds

During the studies on the radical cyclization, we observed an unexpected C=C isomerization of substrates (*Z*)-**8a** and (*Z*)-**9a**, which yielded up to 4.6 ± 0.4 and 2.9 ± 0.3 mM of the corresponding (*E*)-isomers, alongside the expected linear and/or cyclic products (Figure 5A). A related reactivity was previously reported by Yang and co-workers, who described that *Cv*FAP variants and even free FAD catalyze the photoisomerization of the α,β-unsaturated esters that were investigated as substrates in their study.^15^ Even though (*E/Z*)-photoisomerizations via energy-transfer catalysis or direct excitation typically result in photostationary states favoring the respective (*Z*)-isomer,^35,36^ a flavin-mediated energy transfer seemed the most plausible explanation.^15^ However, different from this report, control experiments using free FAD alone failed to promote the isomerization of (*Z*)-**8a** and (*Z*)-**9a** (Figure 5B).

**Figure 5:**
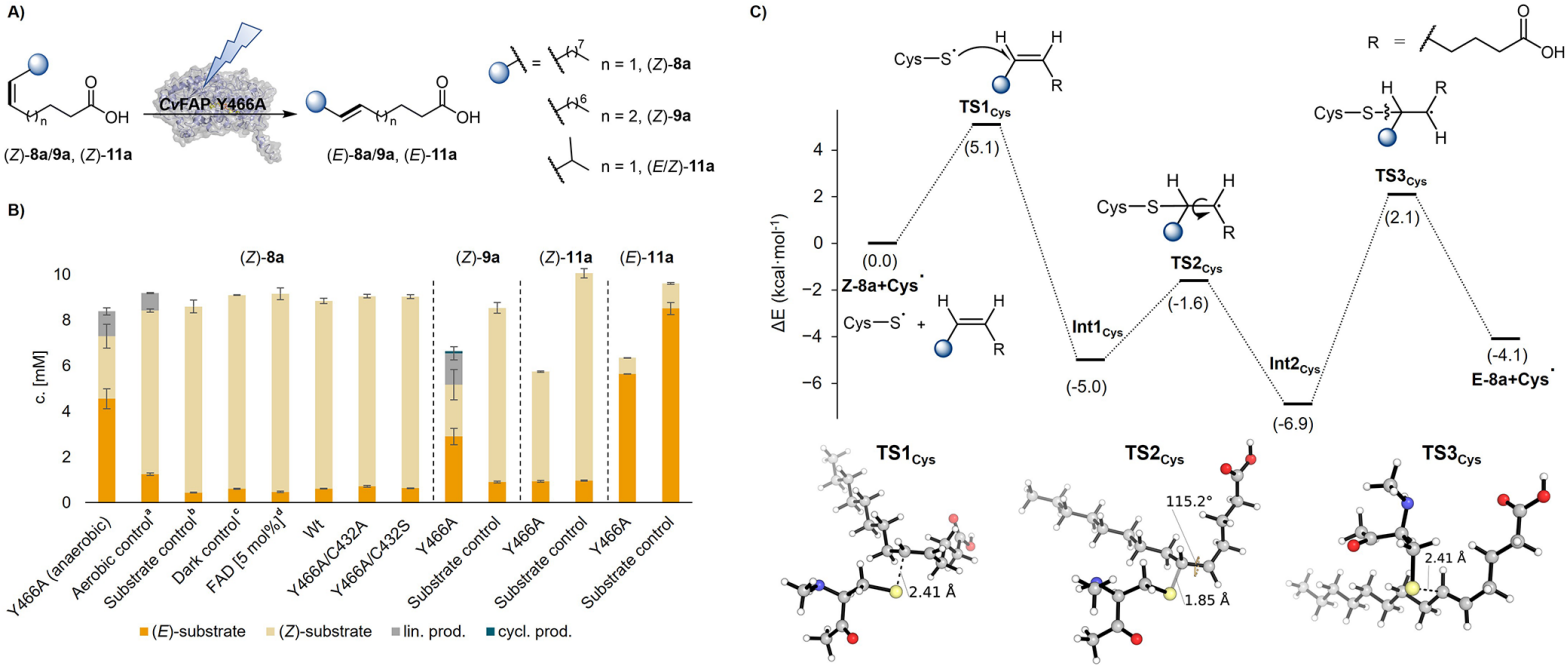
Photobiocatalytic (*Z*) → (*E*) photoisomerization of isolated C=C bonds, catalyzed by *Cv*FAP variant Y466A. (A) Reaction scheme. (B) Substrate scope and control reactions. Reaction conditions: all reactions except the aerobic control reactions were performed under anaerobic (nitrogen atmosphere) conditions; *Cv*FAP wild-type or variants (lyophilized CFE, 20 mg/mL corresponding to 4.5 nmol of *Cv*FAP Y466A), fatty acid ((*Z*)-**8a**, (*Z*)-**9a**, (*Z*)-**11a** or (*E*)-**11a**, 10 mM), 30% v/v DMSO (degassed), in Tris·HCl buffer (100 mM, pH 8.5, degassed) with a final volume of 1 mL; illumination in a custom photoreactor (blue LEDs, 455 nm, 36 μE/L),^29^ 16 h at 25 °C and 500 rpm. ^a^Aerobic control: set up of the experiment under an air atmosphere. ^b^Substrate control: with a substrate and without enzymes under irradiation. ^c^Dark control: in the absence of light. ^d^FAD control: FAD (500 μM), without enzymes under irradiation; Wt = wild type; lin. prod. = linear product (**8b** or **9b**); cycl. prod. = cyclic product (**9c**). Light reactions and controls performed in triplicate. (C) DFT-calculated reaction energy profile for the proposed thiyl-radical-mediated (*Z*) → (*E*) isomerization of **8a**. The model system used includes substrate **8a** and cysteine thiyl radical. Relative electronic energies (Δ*E*, kcal·mol^–1^) are reported with respect to the (*Z* **)-8a** + **Cys** ^**·**^ reactant complex. Optimized geometries of key transition states are shown. Distances and angles are given in Angstrom (Å) and degrees (°), respectively.

Further controls proved that the isomerization of these substrates is light-dependent and exclusively catalyzed by variant Y466A (Figure 5B). Under aerobic conditions, the isomerization activity was significantly reduced. While an energy transfer mechanism based on flavin with an unselective photostationary state might be the mechanism driving the reported isomerization of the α,β-unsaturated esters,^15^ the triplet state energies (*E*_T_) of isolated C=C bonds are significantly higher (e.g., *E*_T_ = 340 kJ/mol for cyclohexene^37^) than the *E*_T_ of flavins (e.g., *E*_T_ of riboflavin = 209 kJ/mol),^36,38^ which argues against a similar triplet–triplet energy-transfer pathway for substates (*Z*)-**8a** and (*Z*)-**9a**.^39^

We therefore sought an alternative plausible mechanistic explanation. In nature, maleate isomerase catalyzes the C=C isomerization of maleate to fumarate via a cysteine-mediated ionic addition/elimination mechanism.^40^ Inspired by this, recently, a chemical (*Z*) → (*E*) isomerization reaction based on cysteine thiyl radicals was developed, which proceeds through radical addition, isomerization, and elimination.^41^ This is intriguing, since *Cv*FAP contains a highly conserved cysteine residue (C432) close to the reaction center, which has been proposed to either contribute to the binding of the substrate and stabilization of the radical intermediate,^26,27^ or the radical termination via HAT or PCET in the natural decarboxylation reaction.^25^ In addition, recent reports suggest it might be involved in quenching of the FAD triplet state, which can be formed upon excitation, if no substrate is bound.^42^ Molecular oxygen is a well-known quencher of triplet excited states and may therefore compete with the proposed quenching pathway involving C432. Accordingly, the observed oxygen sensitivity of the isomerization, combined with the structural positioning of C432, matches a mechanism in which a C432 thiyl-radical, which may have been formed via quenching of FAD,^3^ adds to the double bond, enabling isomerization followed by elimination.

To test this hypothesis, we generated two double variants using *Cv*FAP Y466A as a template, exchanging the cysteine at this position to either alanine or serine. Although such mutations are known to reduce native decarboxylation activity,^25^ the enzyme variant was reported to remain folded and active for promiscuous transformations such as reductive dehalogenation.^16^ Intriguingly, mutating this highly conserved residue completely abolished the isomerization activity, confirming the mechanistic involvement of C432 and supporting its direct role in the mechanism of *Cv*FAP.

Based on this, we computationally explored the proposed thiyl-radical catalyzed isomerization mechanism using (*Z*)-**8a** + **Cys** ^**·**^ as a model substrate (Figure 5C).^41^ Model DFT calculations considering a cysteine thiyl radical and (*Z*)-**8a** as a substrate indicate that the proposed mechanism is indeed intrinsically energetically accessible, proceeding through a rather low energy barrier initial radical addition to the alkene (**TS1**_**Cys**_, Δ*E* ^‡^ = 5.1 kcal·mol^–1^), generating a radical intermediate **Int1**_**Cys**_ that enables an easy C–C rotation (**TS2**_**Cys**_, Δ*E* ^‡^ = 3.4 kcal·mol^–1^) to form the slightly more stable radical intermediate **Int2**_**Cys**_, which after thiyl-radical elimination (**TS3**_**Cys**_, Δ*E* ^‡^ = 9.0 kcal·mol^–1^) leads to the isomerized, and thermodynamically favored, product (*E*)-**8a** + **Cys** ^**·**^. Based on our DFT calculations, alternative ionic or HAT-mechanisms are energetically inaccessible (see Section 4.4 of the Supporting Information for all details).

Testing the structurally different substrates (*Z*)-**11a** and (*E*)-**11a** did not result in (*Z*) → (*E*) and (*E*) → (*Z*) photoisomerization, respectively. The trend can be rationalized by the different length of the aliphatic chains, which position the double bond further away from the crucial C432 residue.

### Enzyme Engineering of *Cv*FAP for Decarboxylative Radical Cyclization

To establish FAPs as versatile templates for radical photobiocatalytic reaction development, we set out to demonstrate the evolvability of *Cv*FAP. Due to the higher conversions observed in the initial experiments for the decarboxylative intramolecular radical cyclization, this reaction was selected as a model system using citronellic acid (**10a**) as substrate. The fact that the experimentally observed product distributions for the tested cyclization reactions (Figure 3) do not match the intrinsic reactivity trends predicted by DFT calculations, which indicate PCET to be much more efficient than cyclization, suggests that substrate repositioning induced by the Y466A mutation plays an important role in steering the reaction outcome. Specifically, this repositioning appears to allow cyclization via C–C bond formation, even though the substrate is not fully geometrically preorganized for this pathway. We reasoned that improving substrate preorganization in the active site, together with slowing the competing natural PCET step, could further enhance cyclization over the native reaction. As a first step, we established a directed evolution workflow for photoenzymes. Genetic diversity was created via iterative rounds of site-saturation mutagenesis (SSM), using the 22c-trick^43^ and QQC (quick quality control) of the degree of saturation (see Section 5.1 of the Supporting Information). Libraries were screened in 96-well glass plates under irradiation with a Lumidox photoreactor equipped with a 445 nm LED array. Based on the initial results, *rac*-**10a** was chosen as the model substrate (Figure 6A). GC-FID was required as a medium-throughput analytical method due to the volatile nature of the products and the absence of chromophores. Because our goal was to favor the coupling reaction over the natural decarboxylation, the screening parameter for the selection of hit variants was the relative amount of the cyclization product (**10c**) in the mixture of products (**10b** + **10c**) formed during the transformation. This value proved to be very robust and reproducible across scales, enzyme concentrations, preparations, and the form of the applied enzyme (whole cells or CFE). To streamline the workflow, screenings were performed with whole cells, which supported the enzyme’s stability^44,45^ and eliminated the need for cell lysis. Identified hit variants were subsequently re-evaluated as lyophilized CFE on a 1 mL scale using both enantiomers of **10a** (see Section 5.3 of the Supporting Information). We started the engineering campaign using wild-type *Cv*FAP as a template, targeting position Y466. Confirming our initial assumption, Y466A emerged as the best variant at this position, producing 15% **10c** and 85% **10b** (Figure 6A).

**Figure 6:**
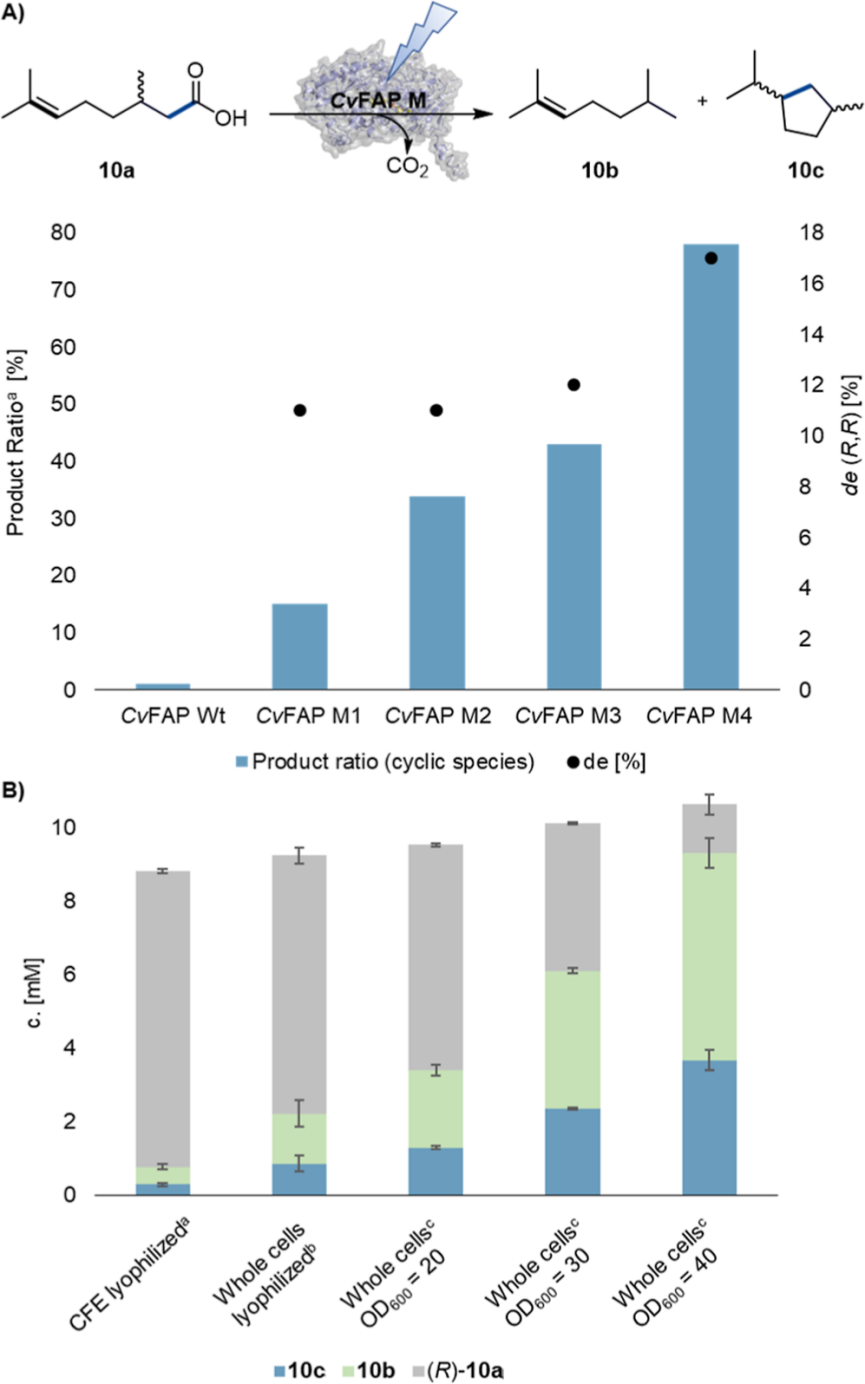
Directed evolution of *Cv*FAP for decarboxylative radical cyclization of carboxylic acids bearing nucleophilic C=C bonds. (A) Profile showing the hit variants of each round of directed evolution. *rac*-**10a** was used as a substrate for rounds 1–3, (*R*)-**10a** in round 4. ^a^Calculated based on the GC-FID area of formed products (**10c**/(**10c** + **10b**) *100). *Cv*FAP M1 = Y466A. *Cv*FAP M2 = Y466A/V453S, *Cv*FAP M3 = Y466A/V453S/G431S, *Cv*FAP M4 = Y466A/V453S/G431S/I130K. (B) Reaction engineering of *Cv*FAP M2 using (*R*)-**10a** under aerobic standard conditions. ^a^Enzyme preparation: lyophilized cell free extract (10 mg/mL, corresponding to a final total protein concentration of 0.94 mg/mL); ^b^enzyme preparation: lyophilized whole cells, 10 mg/mL; ^c^enzyme preparation: resuspended whole cells of different OD_600_ values (corresponding to a CDW of 9.7 ± 0.2 mg, 15.3 ± 0.3 mg and 20.0 ± 0.2 mg, respectively), directly after harvesting. Whole cells (OD_600_ = 40/mL) correspond to 11.1 nmol of *Cv*FAP M2.

In the second round, six positions (L386, I398, G431, V453, G462, and S574) were saturated and screened for improved cyclization activity. The best variant obtained in this round, *Cv*FAP M2 (Y466A/V453S), increased cyclization to 34% of the product mixture. Notably, variant Y466A/G462A, which was a crucial variant for increasing the cyclization activity over the natural decarboxylation in the study of Yang and co-workers,^15^ did not improve cyclization with our model substrate, albeit inverting diastereoselectivity, forming the *cis*-configured (1*S*,3*R*)-product with a *de* of 21% (Table S22). M2 served as a template for the third round, targeting residues F134, G431, A384, and L386. This delivered *Cv*FAP M3 (Y466A/V453S/G431S), with further increased cyclization (43%).

With this evolutionary trajectory in hand, we turned to computational modeling to unravel the origins of the improved selectivity toward cyclization and to guide further enzyme engineering. Computational models of *Cv*FAP WT, M1, and M2 containing the FAD cofactor and the substrate (*R*)-**10a** bound were built and used as starting points for MD simulations (see Section 4.2 in the Supporting Information for details). The simulations revealed marked differences in the substrate binding modes across variants (Figure 7A). In the wild-type enzyme, the substrate remains positioned far from the flavin, sampling predominantly nonproductive conformations. Variant M1 (Y466A) also shows large substrate-FAD distances due to an expanded active site, allowing highly flexible substrate conformations, including ones that are folded to enable C–C bond formation. Interestingly, variant M2 samples substantially shorter distances between the substrate and the FAD, enabling more catalytically relevant poses for both the initial photoinduced decarboxylation and the subsequent radical cyclization step. M2 also restricts substrate flexibility, yielding more frequent conformations with short intramolecular distances between the reacting atoms C2 and C6, geometrically preorganizing the substrate for 5-exo-trig cyclization.

**Figure 7:**
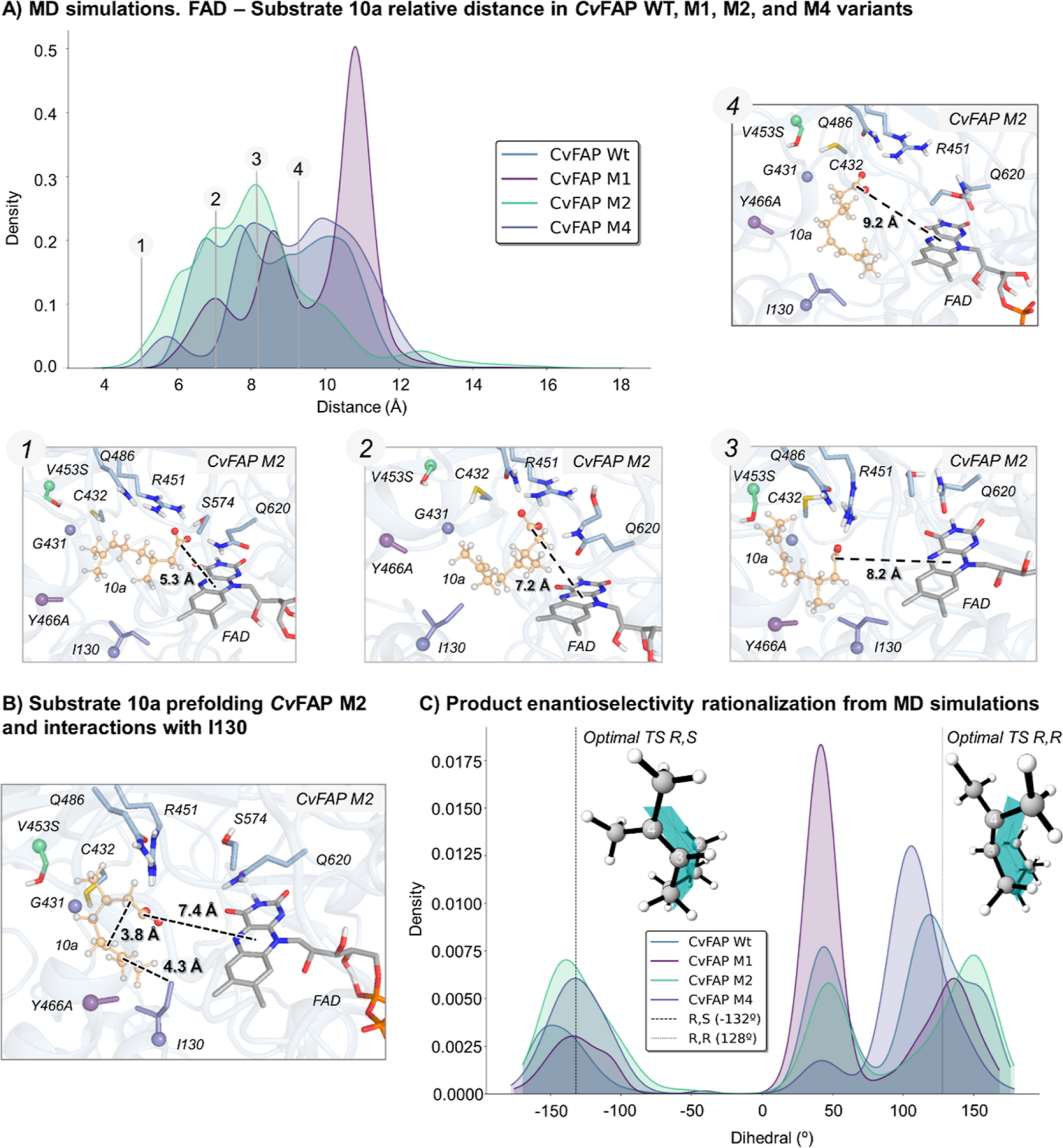
MD simulations of *Cv*FAP WT, M1, M2, and M4 variants. (A) Probability density distribution for the carboxylate group of (*R*)-**10a** -FAD distance obtained from MD simulations of *Cv*FAP WT, M1, M2, and M4 variants and representative snapshots of the substrate (*R*)-**10a** relative distance from FAD, obtained from *Cv*FAP M2 variant MD simulations. All distances are in Å. (B) Representative snapshot extracted from *Cv*FAP M2 variant MD simulations showing the prefolding state of substrate (*R*)-**10a** and interaction with I130. (C) Probability density distribution for the dihedral angle depicting products (1*R*,3*R*)-**10c** and (1*S*,3*R*)-**10c** when the C–C formation bond distance is below 3.5 Å obtained from MD simulations. The optimal TS dihedral angles calculated with DFT are shown for both product diastereomers as dashed lines. These results show the preference toward the (1*R*,3*R*)-**10c**.

Analysis of the ensemble of productive, cyclization-competent poses highlighted a recurring interaction between the terminal region of the alkyl chain and residue I130, suggesting that this position could play a direct role in modulating substrate geometric preorganization and stabilizing geometries that promote radical attack to the C=C. This observation guided the next round of evolution, targeting I130 to bias the enzyme toward binding conformations favoring C–C bond formation (Figure 7B). Finally, we investigated I130, yielding *Cv*FAP M4 (Y466A/V453S/G431S/I130K), which dramatically increased the cyclization activity, forming 80% of **10c** in the product mixture with a turnover number of 452.

Computational modeling for M4 variant revealed that the introduced K130 forms hydrogen bonds with the substrate carboxylate (in cooperation with either R451 or Q486), anchoring the substrate in a midregion of the active site pocket (≈7–8 Å) while still enabling catalytically relevant substrate-FAD distances (<6 Å). This altered binding mode strengthens the substrate’s folding and geometric preorganization for cyclization, exploring shorter C–C distances as compared to previous variants, and notably, sampling conformations preferentially leading to the experimentally observed *pro-R,R* diastereomer (see Figure 7C and Section 4.2 in the Supporting Information). At the same time, the slightly increased substrate-FAD distance in M4 disfavors the native PCET pathway, further tilting the balance toward radical cyclization. These observations reinforce the hypothesis that protein engineering not only favored substrate geometric preorganization for cyclization but also disfavored the native PCET pathway.

Interestingly, the screening of an M3 C432 SSM plate revealed almost no activity, again hinting at the mechanistic importance of this conserved residue. Only trace amounts of exclusive **10c** formation were observed with *Cv*FAP M3 C432A and *Cv*FAP M3 C432V, accompanied by the formation of an unexpected new cyclic tertiary alcohol, **10d** (see below). Moreover, the simulations revealed that in productive conformations, the substrate C=C bond is positioned near the conserved C432 side chain, consistent with its proposed involvement in the reaction mechanism and with its essential role in the observed (*Z*) → (*E*) isomerization activity described above.

Although the selectivity for the cyclized product increased throughout the rounds of directed evolution, overall conversion decreased (see Section 5.3 in the Supporting Information). To address this, we conducted reaction engineering. The best overall product formation of **10c** was found using *Cv*FAP M2, testing different forms of enzyme preparations, comparing *Cv*FAP M2 CFE, lyophilized CFE, and whole cells (Figure 6B). Almost complete conversion was achieved using freshly harvested and resuspended whole cells (40 OD_600_/mL), giving 3.7 mM cyclic product (332 turnovers). This is likely due to increased stabilization of *Cv*FAP in the whole cell preparation, resulting in an increased concentration of active enzyme (Figure S31). Despite its higher turnover number, *Cv*FAP M4 produced lower concentrations of **10c**, due to significantly reduced expression yields in the whole cell preparation.

Testing these variants in the Giese-type coupling (see Figure 2) showed that the formation of **4d** decreased with each additional mutation introduced during the evolution campaign, indicating that engineered variants are increasingly specialized for the targeted cyclization reaction.

### Carbohydroxylation of C=C Bonds

During the engineering of *Cv*FAP M3, we investigated the role of residue C432 and observed a strong reduction in the formation of the cyclic product **10c**. Intriguingly, we also detected a new product, cyclic alcohol **10d** (Figure 8A). The emergence of **10d** upon substitution of the conserved C432 with alanine suggests that this cysteine is involved in the back-electron transfer (or PCET) during the final step of the catalytic cycle of FAPs (Figure 1H, steps g or d). In its absence, this terminating step of the mechanism is slowed, permitting the reaction with oxygen. A related side reaction was observed when testing substrate **12a**, an unsaturated nitrile analogue of **10a**, with the *Cv*FAP single variant V453A (Figure 8B). This substrate contains an electrophilic C=C bond and is thus related to the substrates reported by Yang and co-workers,^15^ which should favor rapid cyclization.^32^ Surprisingly, when converted by the V453A variant of *Cv*FAP, **12a** yielded cyclic ketone **12c** (3% conversion, 0.3 mM) instead of the anticipated nitrile cyclization product. Control experiments revealed that the formation of **12d** was oxygen-dependent, suggesting that molecular oxygen participates in the reaction (Table S19). It is well established that carbon-centered radicals can react with ground-state molecular oxygen to generate peroxyl radicals, which in aqueous systems can further transform into alcohols (or ketones), either via hydroxylation of carbocations formed upon superoxide elimination, or through disproportionation processes.^46^ A plausible pathway involves radical carbohydroxylation of **12a**, producing the corresponding cyanohydrin intermediate, followed by cyanide elimination to the observed ketone **12c**. Taken together, these findings highlight a previously unrecognized reactivity of *Cv*FAP variants: a radical bis-functionalization of C=C bonds via carbohydroxylation.

**Figure 8:**
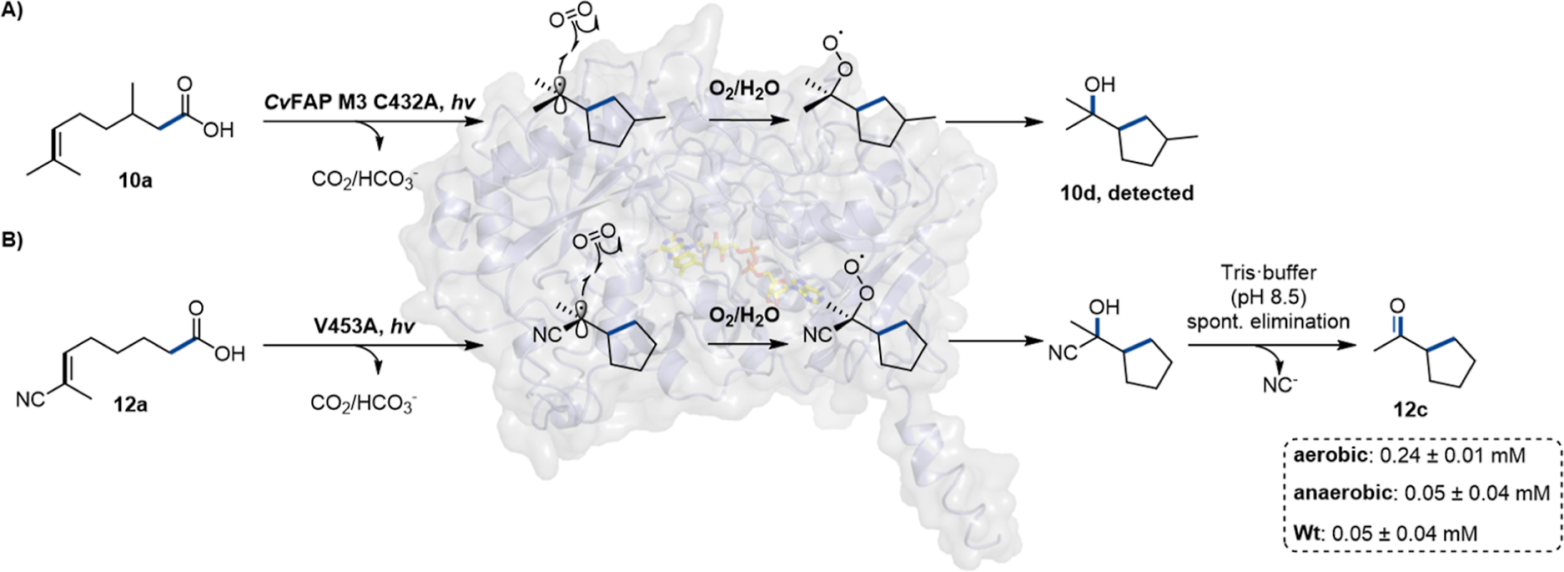
Carbohydroxylation of C=C bonds. (A) Conversion of **10a** to the tertiary alcohol **10d**; (B) carbohydroxylation of **12a,** followed by spontaneous cyanide elimination forming ketone **12c**. Reaction conditions: *Cv*FAP variants (lyophilized CFE, 20 mg/mL, corresponding to 2.7 nmol *Cv*FAP M3 C432A and 5.5 nmol of *Cv*FAP V453A), substrate (**10a**, **12a**: 10 mM), 30% v/v DMSO, in Tris·HCl buffer (100 mM, pH 8.5) with a final volume of 1.0 mL; illumination in a custom photoreactor (blue LEDs, 455 nm, 36 μE/L),^29^ 4–7 h at 25 °C and 500 rpm; aerobic: air atmosphere; anaerobic: under argon atmosphere (Schlenk line) using degassed DMSO and Tris·HCl buffer. Wt = wild type.

## Conclusion

In summary, we established four new classes of reactions that can be catalyzed by FAPs, placing the enzyme family among the most versatile templates for photobiocatalytic radical transformations. First, combining cycloalkenones with fatty acids enabled an intermolecular decarboxylative Giese-type coupling, catalyzed by *Cv*FAP Y466A. Next, we demonstrated that *Cv*FAP mediates coupling of radicals and acceptors of the same philicity by realizing the decarboxylative cyclization of carboxylic acids with internal C=C bonds. Exclusively five-membered rings (**5c**, **9c**, and **10c**) were formed via 5-exo-trig ring closures, with cyclization yields correlating with the stability of the radical intermediates. These investigations revealed a previously unknown cysteine-mediated (*Z*) → (*E*) photoisomerization of alkenes. Finally, we uncovered a new radical carbohydroxylation reaction, representing a C=C bond bis-functionalization, which converted substrates such as **10a** and **12a** into cyclic alcohols or ketones in up to 44 turnovers.

Directed evolution underscored the mutability of *Cv*FAP and the possibility of tailoring the enzyme for these diverse transformations. Engineering of *Cv*FAP supported by computational insights increased the intramolecular radical cyclization efficiency from an initial product ratio of 1% to 80% in variant M4. This variant reached a turnover number of 452, which is high for photobiocatalytic processes that often require enzyme loadings exceeding 1 mol %.^12^ However, overall conversion remained limited due to a decrease in expression yield, highlighting an important limitation that must be addressed in the future. In contrast, variant M2 enabled higher product formation when using whole cells, converting enantiopure (*R*)-**10a** into 3.7 mM **10c** alongside 5.6 mM of **10b**. As whole-cell systems have previously been shown to enhance *Cv*FAP stability,^44,47^ these results suggest that photoinactivation remains a significant limitation for the free enzyme, even for improved variants. Overall, these findings highlight the balance between reactivity and productivity in the engineering of radical photoenzymes and demonstrate that FAPs can be systematically optimized for novel activities.

Computational analyses revealed that the targeted mutations act synergistically to progressively reshape and enforce substrate folding and productive preorganization for cyclization while at the same time disfavoring the binding modes leading to the native PCET pathway. These mechanistic insights highlight how the active-site architecture of *Cv*FAP can be rationally tuned to control and redirect radical intermediates and enable new bond-forming reactivities.

Beyond expanding the catalytic repertoire of the FAP family, our studies also confirmed the significance of the conserved cysteine residue C432. While its exact role in the native decarboxylation reaction remains under debate, we demonstrated its direct involvement in the (*Z*) → (*E*) isomerization activity and found that its substitution promotes follow-up reactions of radical intermediates (e.g., the decreased formation of natural decarboxylation product in favor of carbohydroxylation for *Cv*FAP M3 C432A).

This work demonstrates photobiocatalytic reactivities with clear potential for synthetic chemistry applications, although further improvements in enzyme stability, expression, and substrate scope are required. Applications include selective radical couplings between partners of similar philicity, as well as transformations enabling the formation of two new bonds in a single step. The use of readily available renewable carboxylic acids as radical precursors further enhances the potential of this system.

Together, these insights not only expand the synthetic scope of FAPs but also deepen the mechanistic understanding of how this enzyme family controls radical reactivity.

## Methods

### General Method for the Decarboxylative Radical Cyclization with Nucleophilic C=C Bonds

The setup of the experiment was performed under aerobic conditions. Lyophilized CFE of *Cv*FAP Wt, variant Y466A, or the corresponding *E. coli* cells harboring an empty expression vector (20 mg/mL) was weighted into 1.5 mL screw-cap glass vials and rehydrated for 15 min at room temperature with Tris·HCl buffer (700 μL, 100 mM, pH 8.5). During the half-time of the rehydration process, the vials were gently flicked several times. Stock solution of fatty acids **5a**–**10a** (300 μL, 33.3 mM in DMSO) was added as the last component. The samples were irradiated for 16 h, at 500 rpm, 25 °C in an in-house built photoreactor,^29^ equipped with blue commercial LEDs (455 nm). The parameters duty range and duty cycle were set to 100 and 1% (corresponding to a light intensity of 36 μE/L), respectively. Biotransformations had a final volume of 1.0 mL and a final substrate concentration of 10 mM in Tris·HCl buffer (100 mM, pH 8.5), containing 30% (v/v) of DMSO. Samples were worked up on ice, using precooled extraction solvent. The samples were cooled on ice for 5 min, followed by acidification with aqueous HCl (6 M, 50 μL). The samples were vortexed for 1 min, transferred to 2.0 mL microcentrifuge tubes, and charged with brine (300 μL). The walls of the glass vials were rinsed with either EtOAc [500 μL, containing 10 mM 1-decanol as internal standard, *Z*-(**8a**–**9a**)] or MTBE (500 μL, containing 10 mM, *n*-dodecane as internal standard, **5a–7a** and **10a**), and the solvent was transferred to the respective microcentrifuge tubes. The tubes were vortexed for 1 min and centrifuged (2 min, 14,680 rpm, 4 °C), and 300 μL of the supernatant (organic phase) was transferred to another 1.5 mL microcentrifuge tube, charged with a spatula tip of anhydrous Na_2_SO_4_. After a second extraction cycle (500 μL of extraction solvent +IS), 1 min of vortexing, and centrifugation (2 min, 14,680 rpm, 4 °C), another 500 μL of the supernatant was withdrawn. The combined organic phases (800 μL) were vortexed for 1 min and centrifuged (2 min, 14,680 rpm, 4 °C). For the analysis of product formation, 500 μL of the organic phase was transferred into glass crimp vials and directly measured without derivatization. Samples for the quantification of the substrate were prepared by charging a glass crimp vial with the organic phase (100 μL), pyridine (100 μL), and BSTFA (100 μL) and the incubation for 1 h at 60 °C and 600 rpm in a benchtop thermoshaker. All samples were measured on a GC-FID with either of the methods described in Section 7 of the Supporting Information.

General procedures for all further biotransformations can be found in the Supporting Information.

## Supplementary Material



## Notes

DFT-optimized structures uploaded onto the IOCHEM-BD platform (www.iochem-bd.org)^48^ to facilitate data exchange and dissemination, according to the FAIR principles of OpenData sharing, experimental procedures, quick quality library controls, synthetic procedures, characterizations of synthesized compounds, quantifications, computational methods, and computational data (PDF)

## References

[1] Bai Y. R., Yang X., Chen K. T., Cuan X. D., Zhang Y. D., Zhou L., Yang L., Liu H. M., Yuan S. (2024). A comprehensive review of new small molecule drugs approved by the
FDA in 2022: Advance and prospect. Eur. J. Med.
Chem..

[2] Simic S., Zukic E., Schmermund L., Faber K., Winkler C. K., Kroutil W. (2022). Shortening Synthetic
Routes to Small Molecule Active Pharmaceutical Ingredients Employing
Biocatalytic Methods. Chem. Rev..

[3] Winkler C. K., Schrittwieser J. H., Kroutil W. (2021). Power of Biocatalysis for Organic
Synthesis. ACS Cent. Sci..

[4] Schmidt N. G., Eger E., Kroutil W. (2016). Building Bridges:
Biocatalytic
C-C-Bond Formation toward Multifunctional Products. ACS Catal..

[5] Hyster T. K. (2020). Radical
Biocatalysis: Using Non-Natural Single Electron
Transfer Mechanisms to Access New Enzymatic Functions. Synlett.

[6] Raps F. C., Hyster T. K. (2025). Emergent Mechanisms in Biocatalysis. ACS Cent. Sci..

[7] Alphand V., van Berkel W. J. H., Jurkas V., Kara S., Kourist R., Kroutil W., Mascia F., Nowaczyk M. M., Paul C. E., Schmidt S., Spasic J., Tamagnini P., Winkler C. K. (2023). Exciting Enzymes: Current State and Future Perspective
of Photobiocatalysis. ChemPhotoChem.

[8] Cheng L., Li D., Mai B. K., Bo Z., Cheng L., Liu P., Yang Y. (2023). Stereoselective amino
acid synthesis by synergistic photoredox-pyridoxal radical biocatalysis. Science.

[9] Wang T. C., Mai B. K., Zhang Z., Bo Z., Li J., Liu P., Yang Y. (2024). Stereoselective amino acid synthesis
by photobiocatalytic
oxidative coupling. Nature.

[10] Xing Z., Liu F., Feng J., Yu L., Wu Z., Zhao B., Chen B., Ping H., Xu Y., Liu A., Zhao Y., Wang C., Wang B., Huang X. (2025). Synergistic
photobiocatalysis for enantioselective triple-radical sorting. Nature.

[11] Xu Y., Chen H., Yu L., Peng X., Zhang J., Xing Z., Bao Y., Liu A., Zhao Y., Tian C., Liang Y., Huang X. (2024). A light-driven enzymatic
enantioselective radical acylation. Nature.

[12] Emmanuel M. A., Bender S. G., Bilodeau C., Carceller J. M., DeHovitz J. S., Fu H., Liu Y., Nicholls B. T., Ouyang Y., Page C. G., Qiao T., Raps F. C., Sorigue D. R., Sun S. Z., Turek-Herman J., Ye Y., Rivas-Souchet A., Cao J., Hyster T. K. (2023). Photobiocatalytic
Strategies for Organic Synthesis. Chem. Rev..

[13] Schmermund L., Jurkas V., Ozgen F. F., Barone G. D., Büchsenschütz H. C., Winkler C. K., Schmidt S., Kourist R., Kroutil W. (2019). Photo-Biocatalysis:
Biotransformations in the Presence of Light. ACS Catal..

[14] Sorigue D., Legeret B., Cuine S., Blangy S., Moulin S., Billon E., Richaud P., Brugiere S., Coute Y., Nurizzo D., Muller P., Brettel K., Pignol D., Arnoux P., Li-Beisson Y., Peltier G., Beisson F. (2017). An algal photoenzyme
converts fatty acids to hydrocarbons. Science.

[15] Ju S., Li D., Mai B. K., Liu X., Vallota-Eastman A., Wu J., Valentine D. L., Liu P., Yang Y. (2024). Stereodivergent photobiocatalytic
radical cyclization through the repurposing and directed evolution
of fatty acid photodecarboxylases. Nat. Chem..

[16] Xu W., Mou K., Lu Z., Kang X., Guo Y., Ding B., Chen Z., Wang Z., Wu Q. (2024). Catalytic Promiscuity
of Fatty Acid Photodecarboxylase Enables Stereoselective Synthesis
of Chiral alpha-Tetralones. Angew. Chem., Int.
Ed..

[17] Huang X., Feng J., Cui J., Jiang G., Harrison W., Zang X., Zhou J., Wang B., Zhao H. (2022). Photoinduced chemomimetic biocatalysis
for enantioselective intermolecular radical conjugate addition. Nat. Catal..

[18] Raps F. C., Rivas-Souchet A., Jones C. M., Hyster T. K. (2025). Emergence of a distinct mechanism
of C-N bond formation in photoenzymes. Nature.

[19] Li X., Page C. G., Zanetti-Polzi L., Kalra A. P., Oblinsky D. G., Daidone I., Hyster T. K., Scholes G. D. (2023). Mechanism and Dynamics
of Photodecarboxylation Catalyzed by Lactate Monooxygenase. J. Am. Chem. Soc..

[20] Liu S., Yang R., Xu J. (2025). Photoinduced
Ene-Reductase Catalysis via Electron Donor-Acceptor Complexes. ChemBioChem.

[21] Sandoval B. A., Kurtoic S. I., Chung M. M., Biegasiewicz K. F., Hyster T. K. (2019). Photoenzymatic Catalysis Enables
Radical-Mediated Ketone Reduction in Ene-Reductases. Angew. Chem., Int. Ed..

[22] Huang X., Wang B., Wang Y., Jiang G., Feng J., Zhao H. (2020). Photoenzymatic enantioselective
intermolecular radical hydroalkylation. Nature.

[23] Fu H., Lam H., Emmanuel M. A., Kim J. H., Sandoval B. A., Hyster T. K. (2021). Ground-State Electron
Transfer as an Initiation Mechanism for Biocatalytic C-C Bond Forming
Reactions. J. Am. Chem. Soc..

[24] Sorigue D., Legeret B., Cuine S., Morales P., Mirabella B., Guedeney G., Li-Beisson Y., Jetter R., Peltier G., Beisson F. (2016). Microalgae Synthesize Hydrocarbons from Long-Chain
Fatty Acids via a Light-Dependent Pathway. Plant
Physiol..

[25] Sorigue D., Hadjidemetriou K., Blangy S., Gotthard G., Bonvalet A., Coquelle N., Samire P., Aleksandrov A., Antonucci L., Benachir A., Boutet S., Byrdin M., Cammarata M., Carbajo S., Cuine S., Doak R. B., Foucar L., Gorel A., Grunbein M., Hartmann E., Hienerwadel R., Hilpert M., Kloos M., Lane T. J., Legeret B., Legrand P., Li-Beisson Y., Moulin S. L. Y., Nurizzo D., Peltier G., Schiro G., Shoeman R. L., Sliwa M., Solinas X., Zhuang B., Barends T. R. M., Colletier J. P., Joffre M., Royant A., Berthomieu C., Weik M., Domratcheva T., Brettel K., Vos M. H., Schlichting I., Arnoux P., Muller P., Beisson F. (2021). Mechanism and dynamics
of fatty acid photodecarboxylase. Science.

[26] Heyes D. J., Lakavath B., Hardman S. J., Sakuma M., Hedison T. M., Scrutton N. S. (2020). Photochemical mechanism
of light-driven fatty acid
photodecarboxylase. ACS Catal..

[27] Londi G., Salvadori G., Mazzeo P., Cupellini L., Mennucci B. (2025). Protein-Driven Electron-Transfer
Process in a Fatty
Acid Photodecarboxylase. JACS Au.

[28] Zhou J., Wang Z., Shen Z., Liu X. (2023). Decarboxylative 1,4-Addition of α-Oxocarboxylic Acids with
Michael Acceptors Enabled by Direct Excitation of Flavin-Dependent
“Ene”-Reductases. ACS Sustainable
Chem. Eng..

[29] Winkler C. K., Simić S., Jurkaš V., Bierbaumer S., Schmermund L., Poschenrieder S., Berger S. A., Kulterer E., Kourist R., Kroutil W. (2021). Accelerated Reaction Engineering
of Photo­(bio)­catalytic Reactions through Parallelization with an Open-Source
Photoreactor. ChemPhotoChem.

[30] Zhang W., Ma M., Huijbers M. M. E., Filonenko G. A., Pidko E. A., van Schie M., de Boer S., Burek B. O., Bloh J. Z., van Berkel W. J. H. (2019). Hydrocarbon synthesis
via photoenzymatic decarboxylation of carboxylic acids. J. Am. Chem. Soc..

[31] Zeng Y., Yin X., Liu L., Zhang W., Chen B. (2022). Comparative characterization and
physiological function of putative fatty acid photodecarboxylases. Mol. Catal..

[32] Griller D., Ingold K. U. (1980). Free-radical clocks. Acc. Chem. Res..

[33] Gilmore K., Mohamed R. K., Alabugin I. V. (2016). The Baldwin
rules: revised and extended. Wiley Interdiscip.
Rev. Comput. Mol. Sci..

[34] Hancock A. N., Schiesser C. H. (2013). Guidelines for radical reactions:
some thirty years
on. Chem. Commun..

[35] Nevesely T., Wienhold M., Molloy J. J., Gilmour R. (2022). Advances in the E →
Z Isomerization of Alkenes Using Small Molecule Photocatalysts. Chem. Rev..

[36] Metternich J. B., Gilmour R. (2015). A Bio-Inspired, Catalytic E →
Z Isomerization
of Activated Olefins. J. Am. Chem. Soc..

[37] Ni T., Caldwell R. A., Melton L. A. (1989). The relaxed
and spectroscopic energies
of olefin triplets. J. Am. Chem. Soc..

[38] Montalti, M. ; Credi, A. ; Prodi, L. ; Gandolfi, M. T. Handbook of Photochemistry; CRC Press, 2006.

[39] Dutta S., Erchinger J. E., Strieth-Kalthoff F., Kleinmans R., Glorius F. (2024). Energy transfer photocatalysis:
exciting modes of reactivity. Chem. Soc. Rev..

[40] Fisch F., Fleites C. M., Delenne M., Baudendistel N., Hauer B., Turkenburg J. P., Hart S., Bruce N. C., Grogan G. (2010). A covalent succinylcysteine-like
intermediate in the
enzyme-catalyzed transformation of maleate to fumarate by maleate
isomerase. J. Am. Chem. Soc..

[41] Kosaka S., Kurebayashi K., Yamato N., Tanaka H., Haruta N., Yamamoto M. (2025). Thiyl chemistry: cysteine-catalyzed maleate isomerization
via aqueous thiyl radical processes. Green Chem..

[42] Ma J., Kalapothakis J. M., Spacey H. J., Johannissen L. O., Shrimpton-Phoenix E., Shanmugam M., Sakuma M., Wood C. W., Barran P. E., Heyes D. J., Scrutton N. S. (2025). Triplet Quenching
by Active Site Cysteine Residues Improves Photo-stability in Fatty
Acid Photodecarboxylase. bioRxiv.

[43] Acevedo-Rocha C. G., Reetz M. T., Nov Y. (2015). Economical
analysis of saturation
mutagenesis experiments. Sci. Rep..

[44] Wu Y., Paul C. E., Hollmann F. (2021). Stabilisation of the Fatty Acid Decarboxylase
from *Chlorella variabilis* by Caprylic Acid. ChemBioChem.

[45] Simic S., Jakstaite M., Huck W. T. S., Winkler C. K., Kroutil W. (2022). Strategies for Transferring
Photobiocatalysis to Continuous Flow Exemplified by Photodecarboxylation
of Fatty Acids. ACS Catal..

[46] von Sonntag C., Schuchmann H. P. (1991). The Elucidation
of Peroxyl Radical Reactions in Aqueous Solution with the Help of
Radiation. ngew. Chem. Int. Ed..

[47] Chanquia S. N., Benfeldt F. V., Petrovai N., Santner P., Hollmann F., Eser B. E., Kara S. (2022). Immobilization and Application of
Fatty Acid Photodecarboxylase in Deep Eutectic Solvents. ChemBioChem.

[48] Berga, C. ; Garcia-Borràs, M. ; Feixas, F. Computational Data for this study; 2026, 10.19061/iochem-bd-4-96.

