# Supplementary material for: Establishing the Fatty Acid Photodecarboxylase CvFAP as a Platform for Photobiocatalytic Radical Transformations
Source: ACS Catal. 2026 Apr 6;16(8):7758–70. doi: 10.1021/acscatal.6c01333 (PMC13097135; doi:10.1021/acscatal.6c01333)
Supplement: Supplementary file 1 [file cs6c01333_si_001.pdf]

Supporting Information

Establishing the Fatty Acid Photodecarboxylase *Cv*FAP as a Platform for Photobiocatalytic Radical Transformations

Florian Weissensteiner[a], Cristina Berga[b], Emilia Iglesias-Moncayo[a], Sara Salehi[a], Isabel Oroz-Guinea[a,d], Klaus Zangger[a,c,d], Ferran Feixas[b], Marc Garcia-Borràs*[b,e], Wolfgang Kroutil[a,c,d], Christoph K. Winkler[a]*

[a] F. Weissensteiner, E. Iglesias-Moncayo, S. Salehi, I. Oroz-Guinea, K. Zangger, W. Kroutil, C. K. Winkler.
Institute of Chemistry, University of Graz, Heinrichstraße 28, 8010 Graz, Austria
[b] C. Berga, F. Feixas, M. Garcia-Borràs
Institut de Química Computacional i Catàlisi and Departament de Química, Universitat de Girona, 17003, Girona, Spain.
[c] K. Zangger, W. Kroutil
BioTechMed Graz, 8010 Graz, Austria
[d] K. Zangger, W. Kroutil, I. Oroz-Guinea
Field of Excellence BioHealth, University of Graz, 8010 Graz, Austria.
[e] M. Garcia-Borràs
ICREA, Pg. Lluís Companys 23, 08010 Barcelona, Spain

Corresponding Authors:

Christoph K. Winkler – Department of Chemistry, University of Graz, A8010, Graz, Austria; https://orcid.org/0000-0003-3068-9817; Email: christoph.winkler@uni-graz.at

Marc Garcia-Borràs – Institut de Química Computacional i Catàlisi and Departament de Química, Universitat de Girona, 17003, Girona, Spain; https://orcid.org/0000-0001-9458-1114; Email: marc.garcia@udg.edu

1 General Procedures for Biotransformations 2

2 Directed Evolution Workflow and Procedures 5

3 Computational Details 12

4 Supplementary Computational Data 15

5 Supplementary Experimental Data 47

6 Synthesis of Substrates and Reference Material 70

7 Analytical Methods 83

8 Chromatograms and Spectra 102

9 Primers and Sequences 139

10 References 151

1 General Procedures for Biotransformations

The set-up of the biotransformation at 1 mL scale was carried out in the absence of light when using *Cv*FAP Wt or its variants until the reactions were started *via* illumination using an in-house built photoreactor (blue light, 455 nm).[1] The dark control was performed using the same reactor in the absence of light. The substrate control was performed under the same irradiation conditions (blue light, 455 nm) omitting the enzyme. Further control reactions, such as FAD (1 or 5 mol%), $NADP^+$ (1 mol%) or NADPH (1 mol%) were illuminated with the substrate but without enzyme. All controls were set up and worked up as described in the respective sections.

1.1 Intermolecular decarboxylative radical coupling with electrophilic C=C bonds

The preparation of the experiment was performed under anaerobic conditions in a glove box [≤1 ppm O_2] using Tris·HCl buffer (100 mM, pH 8.5) and DMSO, which were degassed in three cycles of freeze-pump-thaw. The respective lyophilized CFEs (cell-free extracts) and substrates (enone and fatty acid) were weighted into 1.5 mL screw-cap glass vials outside of the glove box and then introduced into the inert chamber. The lids were opened for 10 min to remove residual oxygen from the headspace of the vials. The CFEs of *Cv*FAP Wt, variant Y466A or the corresponding *E. coli* cells harboring an empty expression vector (20 mg/mL) were rehydrated with Tris·HCl buffer (700 µL, 100 mM, pH 8.5) for 15 min. During half time of the rehydration process the vials were gently flicked several times. Stock solution of fatty acids **1a**-**2a** (150 µL, 66.7 mM in DMSO) and enones **3a**-**3c** (150 µL, 133.3 mM in DMSO) were added as last component. The samples were irradiated for 16 h, at 500 rpm, 25°C in an in-house built photoreactor[1], equipped with blue commercial LEDs (455 nm). The parameters duty range and duty cycle were set to 100, and 1% (corresponding to a light intensity of 36 µE/L), respectively. The biotransformations had a final volume of 1.0 mL and a final fatty acid and enone concentration of 10 mM, and 20 mM, respectively, in Tris·HCl buffer (100 mM, pH 8.5), containing 30% (v/v) of DMSO. The samples were worked up as follows: First the biotransformations were acidified with aqueous HCl (6 M, 50 µL), vortexed for 1 min transferred to 2.0 mL microcentrifuge tubes and charged with brine (300 µL). The walls of the glass vials were rinsed with EtOAc (500 µL, containing 10 mM *n*-decane as internal standard) and the solvent was transferred to the respective microcentrifuge tubes. The tubes were vortexed for 1 min, centrifuged (2 min, 14680 rpm, 4 °C) and 350 µL of the supernatant were transferred to another 1.5 mL microcentrifuge tube, dried with a spatula tip of anhydrous Na_2SO_4, vortexed and centrifuged (2 min, 14680 rpm, 4 °C). For the analysis of product formation, 200 µL of the organic extract were transferred into glass crimp vials with glass inlets and measured directly without any derivatization. Samples for the quantification of the substrate were derivatized by charging a glass crimp vial with the organic phase (100 µL), pyridine (100 µL) and BSTFA (100 µL) and incubation for 1 h at 60 °C and 600 rpm in a benchtop thermoshaker. All samples were measured on a

GC-FID with **method GC-1** as described in **section 7.1**. Determination of enantiomeric excess was carried out with **method GC6-GC8** as described in **section 7.2**.

1.2 Decarboxylative radical cyclization with nucleophilic C=C bonds

The setup of the experiment was performed under aerobic conditions. Lyophilized CFE of *Cv*FAP Wt, variant Y466A or the corresponding *E. coli* cells harboring an empty expression vector (20 mg/mL) was weighted into 1.5 mL screw-cap glass vials and rehydrated for 15 min at room temperature with Tris·HCl buffer (700 µL, 100 mM, pH 8.5). During half time of the rehydration process the vials were gently flicked several times. Stock solution of fatty acids **5a**-**10a** (300 µL, 33.3 mM in DMSO) were added as last component. The samples were irradiated for 16 h, at 500 rpm, 25 °C in an in-house built photoreactor[1], equipped with blue commercial LEDs (455 nm). The parameters duty range and duty cycle were set to 100, and 1% (corresponding to a light intensity of 36 µE/L), respectively. Biotransformations had a final volume of 1.0 mL and a final substrate concentration of 10 mM in Tris·HCl buffer (100 mM, pH 8.5), containing 30% (v/v) of DMSO. Samples were worked up on ice, using pre-cooled extraction solvent. The samples were cooled on ice for 5 min, followed by acidification with aqueous HCl (6 M, 50 µL). The samples were vortexed for 1 min, transferred to 2.0 mL microcentrifuge tubes and charged with brine (300 µL). The walls of the glass vials were rinsed with either EtOAc (500 µL, containing 10 mM 1-decanol as internal standard, *Z*-(**8a**-**9a**)) or MTBE (500 µL, containing 10 mM, *n*-dodecane as internal standard, **5a**-**7a**, **10a**) and the solvent was transferred to the respective microcentrifuge tubes. The tubes were vortexed for 1 min, centrifuged (2 min, 14680 rpm, 4°C) and 300 µL of the supernatant (organic phase) were transferred to another 1.5 mL microcentrifuge tube, charged with a spatula tip of anhydrous Na_2SO_4. After a second extraction cycle (500 µL extraction solvent + IS), 1 min of vortexing and centrifugation (2 min, 14680 rpm, 4 °C), another 500 µL of the supernatant were withdrawn. The combined organic phases (800 µL) were vortexed for 1 min and centrifuged (2 min, 14680 rpm, 4 °C). For the analysis of product formation 500 µL of the organic phase were transferred into glass crimp vials and directly measured without derivatization. Samples for the quantification of the substrate were prepared by charging a glass crimp vial with the organic phase (100 µL), pyridine (100 µL) and BSTFA (100 µL) and incubation for 1 h at 60 °C and 600 rpm in a benchtop thermoshaker. All samples were measured on a GC-FID with either of the methods described in **section 7**.

1.3 (*Z*)→(*E*) photoisomerization of isolated C=C bonds

The setup of the experiment was performed under anaerobic conditions in a glove box [≤1 ppm O_2] using Tris·HCl buffer (100 mM, pH 8.5) and DMSO, which was degassed in three cycles of freeze-pump-thaw. The respective lyophilized CFEs and substrates were weighted into 1.5 mL screw-cap glass vials outside of the glove box and then introduced into the inert chamber. The lids were opened for 10 min

to remove residual oxygen from the headspace of the vials. The CFE of *Cv*FAP Wt or its variants Y466A, Y466A/C432A and Y466A/C432S (20 mg/mL) were rehydrated with Tris·HCl buffer (700 µL, 100 mM, pH 8.5) for 15 min. During half time of the rehydration process the vials were gently flicked several times. The stock solution of substrates (*Z*)-**8a**, (*Z*)-**9a,** (*Z*)-**11a**, (*E*)-**11a** (300 µL, 33.3 mM in DMSO) were added as last component. The samples were irradiated for 16 h, at 500 rpm, 25 °C in an in-house built photoreactor[1], equipped with blue commercial LEDs (455 nm). The parameters duty range and duty cycle were set to 100, and 1% (corresponding to a light intensity of 36 µE/L), respectively. The biotransformations had a final volume of 1.0 mL and a final fatty acid concentration of 10 mM, in Tris·HCl buffer (100 mM, pH 8.5), containing 30% (v/v) of DMSO. Samples were worked up as described in **section 1.2** using EtOAc, containing 10 mM 1-decanol as internal standard. Samples for both the substrate and product quantification were measured on a GC-FID with **method GC4** as described in **section 7.**

1.4 Carbohydroxylation of C=C bonds

The setup of the experiment was performed as described in **section 1.2**, using either *Cv*FAP M3/C432A or V453A with substrates (*R*)-**10a** and (*E*/*Z*)-**12a**, respectively and a reaction time (illumination duration) of 4 h for compound **10a** and 7 h for compound **12a**. The work up was carried out as described in **section 1.2** but without acidification and using MTBE as extraction solvent, containing 10 mM *n*-dodecane as internal standard. For substrate (*E*/*Z*)-**12a** only the formation of cyclic product was followed. The samples were measured on a GC-FID with **method GC3** as described in **section 7**.

2 Directed Evolution Workflow and Procedures

2.1 Medium-throughput directed evolution workflow

The overall workflow for the medium throughput directed evolution of *Cv*FAP is depicted in **Figure S1**, including generation of genetic diversity using the 22c-trick[2] (SSM-libraries), Quick Quality Control (QQC) of the generated mutant libraries, cultivation and expression of whole cells in 96 well format, and photobiocatalytic screening in 96 well glass microtiter plates.

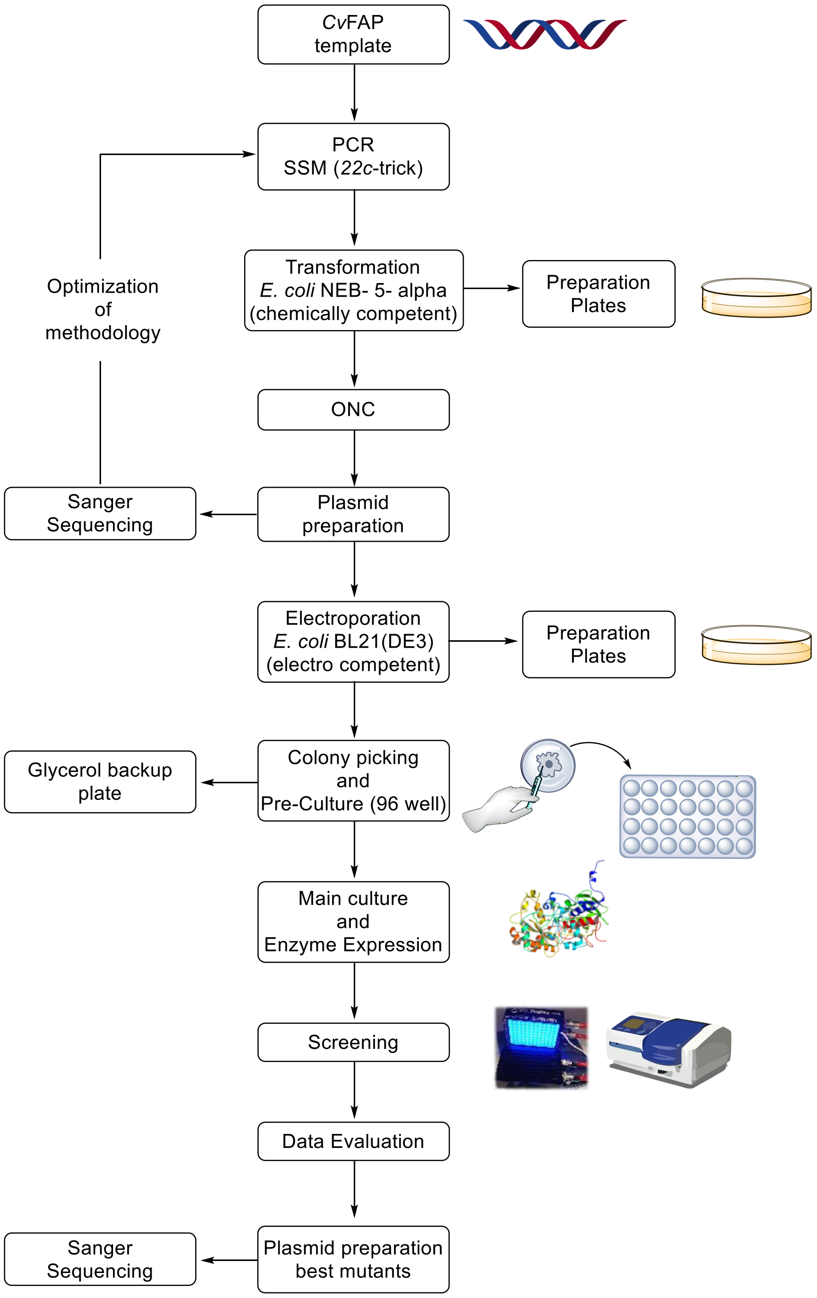

Figure S1. *Workflow for the site saturation mutagenesis of CvFAP towards photoenzymatic intramolecular cyclization reactions.*

2.2 PCR for the generation of SSM libraries

The PCR was carried out using the Q5® Site Directed Mutagenesis Kit from New England BioLabs (E0552S). Primers (see **section 9**) were either designed by NEBaseChanger or manually using CLC Workbench and ordered from Eurofins. For all the following steps, nuclease-free $d\mathrm{H_2O}$ was used. The primers were thawed to room temperature, followed by the addition of $d\mathrm{H_2O}$ as stated by the provider to generate 100 µM stock solutions. These stock solutions were then diluted 1:10 with $d\mathrm{H_2O}$ to obtain 10 µM solutions. A forward primer master mix solution was prepared by mixing 10 µL of the TGG-Fw-primer (10 µM), 90 µL of VHG-Fw-primer (10 µM) and 120 µL of NDT-Fw-primer solution (10 µM). The PCR reaction mixture was prepared on ice in PCR tubes with a final volume of 25 µL by mixing Q5 Hot Start High Fidelity 2x Master Mix (12.5 µL), forward primer (Master Mix, 10 µM, 1.25 µL), reverse primer (10 µM, 1.25 µL), template DNA (*Cv*FAP Wt or variants, c = 2 ng/µL, 1.0 µL) and $d\mathrm{H_2O}$ (9.0 µL). The PCR tubes were spun down for 10 s, placed in the PCR thermocycler, and incubated with the cycling conditions shown in **Table S1**.

Table S1. *PCR protocol for site saturation mutagenesis of selected positions.*

Step	Temperature	Time
Initial denaturation	98 °C	30-60 seconds
25 Cycles	98 °C	10 seconds
	Ta = variable	30 seconds
	72 °C	3:45 minutes
Final Extension	72 °C	2 minutes
Hold	4 °C	

2.3 KLD-treatment and transformation into NEB5-α competent *E. coli* cells

The PCR products of the previous step were subjected to a KLD treatment (Q5® Site Directed Mutagenesis Kit; NEB: E0552S). The KLD reaction was set up in a new PCR tube with a final volume of 10 µL, by mixing PCR product (1 µL), 2x KLD Reaction Buffer (5 µL), 10x KLD Enzyme Mix (1 µL) and $d\mathrm{H_2O}$ (3 µL). Samples were incubated at room temperature for 5 min, followed by a prolonged incubation at 37 °C for 45 min. The KLD buffer and enzyme mix were kept on ice during the whole procedure. Finally, the KLD-treated PCR product was used for transformation into NEB-5-alpha chemically competent *E. coli* cells. The cells (50 µL) were allowed to thaw on ice, followed by the addition of the KLD-treated PCR product (5 µL) and gentle mixing. The mixture was incubated on ice for 30 min, after which the mixture was heat shocked at 42 °C for 30 seconds, and then placed on ice for 5 minutes. Prewarmed SOC medium (37 °C, 950 µL) was added and samples were incubated in a thermoshaker (37 °C, 1 hour, 300 rpm). Finally, the transformant solutions (400 µL) were streaked on LB agar plates supplemented with kanamycin and the plates were incubated overnight at 37 °C.

Overnight cultures of the transformants of the generated libraries were prepared as described in **section 2.4** for the Quick Quality Control.

2.4 Plasmid miniprep

Prior to the isolation of respective plasmids overnight cultures (ONCs) were prepared as follows. A falcon tube was charged with LB medium (15 mL) and supplemented with kanamycin (0.1% v/v, 15 µL of a 50 mg/mL stock in *d*H_2O). The solution was inoculated with either transformant solution (400 µL) from **section 2.3** (for QQC of SSM libraries) or from individual wells of a glycerol back up plate from **section 2.7.3** (5 µL) for sequencing and evaluation of hit variants of SSM-trials. ONCs were incubated overnight in the thermoshaker (37 °C, 120 rpm, 16 h). Glycerol stocks were prepared by adding the respective ONC (800 µL) to an autoclaved glycerol solution (800 µL; 60% in *d*H_2O) and were stored at -80 °C.

The plasmid preparation was performed in duplicates according to the kit from Monarch (Plasmid Miniprep kit). First 4 mL of the ONCs were centrifuged (14680 rpm, 4 minutes) and the supernatant was discarded. The cell pellet was resuspended using plasmid resuspension buffer (200 µL). Afterwards, plasmid lysis buffer (200 µL) was added and the samples were incubated for 1 min at room temperature, observing a change of color to pink. Then, plasmid neutralization buffer (400 µL) was added and samples were gently inverted until color changed to yellow. After another 2 min of incubation samples were centrifuged (5 min, 14680 rpm) and the supernatant was transferred to the spin column, which was centrifuged (1 min, 14680 rpm), discarding the flowthrough. The centrifugation steps were repeated with plasmid wash buffer 1 (200 µL), and a second time with wash buffer 2 (400 µL). Finally, the column was transferred to a sterile 1.5 mL microcentrifuge tube, and prewarmed nuclease free *d*H_2O (50 µL, 50 °C) was added to the center of the column. After one minute of incubation the samples were centrifuged (1 min, 14680 rpm) and the concentration was determined using a Nanodrop (IMPLEN NanoPhotometer NP80). Obtained plasmid solutions were stored at -20 °C until further use.

2.5 Sanger sequencing and Quick Quality Control of the libraries

Plasmid solutions obtained in **section 2.4** were aliquoted (24 µL for forward and reverse run; 12 µL for mid primer run, respectively) and sent for Sanger Sequencing to Microsynth Austria GmbH. The quality of the library or the identification of hit variants from SSM-Plate trials was then evaluated and analyzed using the software CLC-Workbench and Chromas. The mid primer for *Cv*FAP was: CTGGACTGGAACCTGTTCTCTG. Results of the QQC are reported in **section 5.1**.

2.6 Transformation into electrocompetent *E. coli* BL21 (DE3) cells *via* electroporation

Tubes containing *in-house* prepared electrocompetent *E. coli* BL21(DE3) cells (50 µL) were thawed on ice. The cuvettes for electroporation (0.2 cm) were placed on ice prior to the electroporation. On ice and under sterile conditions the respective plasmid solution (2 µL) was transferred to the tube containing the electrocompetent cells (50 µL). The suspension was homogenized by gentle flicking and placed on ice for 1 min. The content of the microcentrifuge tube was transferred to the electroporation cuvette and tapped to ensure the suspension is in the bottom of the cuvette. The cuvette was inserted into the chamber of the Micro Pulser™ Electroporator (Bio-Rad) using method Ec2 (V = 2.5 kV, 0.2 cm cuvettes) for the electroporation. The cuvette was immediately charged with SOC medium (1 mL, preheated at 37 °C) under sterile conditions, the cell suspension was transferred to a 1.5 mL microcentrifuge tube and incubated in the thermoshaker (37 °C, 120 rpm, 1 h). Finally, 100 µL of the solution was streaked on a LB agar plate supplemented with kanamycin and the plate was incubated overnight at 37 °C.

2.7 Cell cultivation and expression of SSM libraries in 96 well format

2.7.1 Pre-culture preparation

For cultivation of the *E. coli* cells heterologously expressing *Cv*FAP variants from *C. variabilis*, pre-cultures were prepared in a 96 well format using sterile PS microtiter plates. A falcon tube was charged with sterile TB medium (25 mL), which was supplemented with sterile-filtered kanamycin (0.1% v/v, 25 µL of a 50 mg/mL stock in *d*H_2O). In each well of the 96 wells, the TB medium (200 µL, supplemented with the antibiotic) was added using a multichannel pipette. From the plates of the SSM libraries (**section 2.6**), 84 single colonies were picked using sterile toothpicks (autoclaved) and placed in the individual wells. Row G of the 96 well plate served as blank and control for cross contaminations and was not inoculated by single colonies. The plate was covered with an Aera Seal™ membrane and placed in the *d*H_2O air saturated thermoshaker (50 mm throw, 37 °C, 240 rpm, 3 h).

2.7.2 Main culture preparation

For main culture preparation, a falcon tube was charged with sterile TB Medium (25 mL), which was supplemented with sterile filtrated kanamycin (0.1% v/v, 25 µL of a 50 mg/mL stock in *d*H_2O). A new sterile 96 well PS microtiter plate was used and the sterile TB medium (200 µL, supplemented with the antibiotic) was added to each well. Afterwards, 10 µL of the previously prepared preculture MTP (**section 2.7.1**) was transferred to the corresponding well using a multichannel pipette. The microtiter plate was covered using an Aera Seal™ membrane and incubated in a *d*H_2O air saturated thermoshaker (50 mm throw, 37 °C, 240 rpm, 2 h). Gene expression was induced without cooling of the cultures, by adding IPTG (10 µL per well, 20 mM solution in water). The plate was transferred to an *d*H_2O air saturated orbital shaker (Cytomat 2C450; ThermoFisher) and incubated overnight (18 h, 17 °C,

600 rpm, 2 mm amplitude) in the absence of light, covered by a sterile lid. The cells were then pelleted in the absence of light by centrifugation (4 °C, 4500 rpm, 25 min) and the supernatant was discarded by decanting the liquid. Plates containing the pelleted cells were covered with a sterile lid and aluminum foil and stored at -80 °C until further use.

2.7.3 Glycerol back-up plate

Glycerol back-up plates were generated by adding a sample (100 µL) of the respective pre-culture plate (**section 2.7.1**) to a 96 well PS-MTP already charged with autoclaved glycerol (100 µL, 60% in *d*H_2O). Plates were covered with a sterile lid and stored in the -80°C freezer for the identification of hit variants of respective SSM-trials throughout the evolution campaign.

2.8 Screening of *Cv*FAP SSM libraries with *rac*-citronellic acid in MTPs

The procedures for the setup of the biotransformation were carried out in the absence of light. The PS-MTP containing the pelleted cells expressing the variants obtained in **section 2.7.2** were thawed at room temperature for 10 min. The whole cells were resuspended in Tris·HCl buffer (105 µL, 100 mM, pH 8.5) in a bio shaker (50 mm shaking throw, 250 rpm, 20 °C, 25 min) and were then transferred to the corresponding well in a 96 well quartz glass MTP, followed by the addition of (*rac*)-**10a** (45 µL of a 33.3 mM stock in DMSO). Additionally, each well was overlayed with *n*-dodecane (20 µL), acting as a water immiscible protective layer to prevent the loss of the volatile products. The quartz glass plate was covered with a solvent resistant foil (Zone-Free™ Sealing Films; Z721646-50EA: Merck) and placed in the Lumidox photoreactor, which was mounted on a bench top shaker and illuminated while shaking (445 nm, 500 rpm, and 24 mA/well, 25 °C for 4 h). Afterwards, the samples of each well were transferred to individual 1.5 mL microcentrifuge tubes. The whole work up was carried out on ice, using pre-cooled extraction solvent. In addition, the glass plate was cooled on ice for 5 min, prior to removing the solvent resistant foil. To each well EtOAc (150 µL, containing 10 mM D/L-limonene as IS) was added with a multichannel pipette. Each well was mixed a single time by pipetting up and down and then transferred to its respective microcentrifuge tube (1.5 mL). The samples were vortexed (1 min) and centrifuged (2 min, 14860 rpm, 4 °C). The organic phase (100 µL) was withdrawn without additional drying and transferred to glass crimp vials with glass inlet and analyzed on a GC-FID using **method GC-5** as described in in **section 7**.

2.9 Evaluation and characterization of *Cv*FAP hit variants

2.9.1 Production of *Cv*FAP Wt and variants as lyophilized cell free extract

For cultivation of cells, heterologously expressing *Cv*FAP variants from overnight cultures were prepared by charging falcon tubes (50 mL) with LB-medium (15 mL), supplemented with a sterile filtrated kanamycin solution (15 µL of a 50 mg/mL stock in *d*H_2O) and inoculated with glycerol stocks

of *E. coli* BL21 (DE3) cells, containing a pET28a-His-Trx-FAP-plasmid that carries the respective point mutation (5 µL). The glycerol stock was kept on ice throughout the whole procedure and stored at -80 °C. The resulting cultures were shaken overnight at 37 °C and 120 rpm.

For the cultivation, baffled Erlenmeyer flasks (1 L) were used for a final volume of 300 mL. The TB-base for cell cultivation (1.0 L TB-Medium in total) was prepared by dissolving yeast extract (24 g), tryptone (12 g) and glycerol (4 mL) in *d*H_2O (0.9 L) in one flask while the 10 x KPi buffer was prepared in another flask by dissolving KH_2PO_4 (2.31 g) and K_2HPO_4 (12.54 g) in *d*H_2O (0.1 L). Cultivation flasks for the main cultures were heated to 200 °C for 2 h for dry sterilization and allowed to cool before adding the TB-base (270 mL/flask). The TB-base medium in the cultivation flasks and the 10 x KPi-buffer were autoclaved separately. Upon cooling, sterile filtrated kanamycin (0.1% v/v, 300 µL/flask; 50 mg/mL in *d*H_2O), the 10x KPi-buffer (30 mL/flask), and an aliquot of the ONCs (1% v/v, 3 mL) were added to the flasks containing the TB-base under sterile conditions. The main cultures were then incubated at 37 °C, 120 rpm until an OD_{600} of 1.0-1.2 was reached (~140 min). The flasks were then allowed to cool down at 4 °C for 1 h, upon which gene expression was induced by adding sterile filtrated IPTG (isopropyl-*β*-D-thiogalactoside; 300 µL, 1 mol/L stock in *d*H_2O). The cultures were covered with aluminum foil and then incubated overnight (18 h) at 17 °C and 120 rpm. From here on every step was performed in the absence of light with only dimmed white light in the background. Cells of the main cultures were harvested by centrifugation at 4 °C and 8000 rpm (12000 x g) for 10 min. After discarding the supernatant, cells were resuspended in lysis buffer (Tris·HCl buffer, 50 mM, 100 mM NaCl, pH 8.0; ~ 3 mL/g of wet cells). The cell suspensions were then transferred to falcon tubes (50 mL) and lysed on ice (0 °C) in fractions of 15 mL using a sonicator (Branson 250 Digital Sonifier) under the following parameters: Total Runtime: 2.30 min, Pulse ON: 2 sec, Pulse OFF: 4 sec, Amplitude: 30%. Cell debris were pelleted at 17000 rpm, 4 °C for 20 min and the supernatant was collected. An aliquot of the cell pellet (pipette tip) and the supernatant (50 µL), respectively, were stored in a microcentrifuge tube at -21 °C for SDS-PAGE analysis. The rest of the cell lysate was shock frozen in N_2(l), lyophilized overnight, and stored at -21 °C under protection from light.

2.9.2 SDS-PAGE

For SDS-PAGE analysis, volumes equivalent to 15 µg of total enzyme concentration (determined *via* Bradford Assay) were charged with Laemmli sample buffer (2x) in a ratio of 1:1, incubated for 5 minutes at 95 °C, and spun down (30 s, 14680 rpm). The respective samples were loaded onto a 10 % Bis-Tris commercial SDS-PAGE gel (160 V, MOPS-Tris buffer), together with a marker (PageRuler Prestained™: 7.5 µL). The gel was stained overnight in a solution of Coomassie Brilliant Blue Quick Stain at 35 rpm, and finally destained with *d*H_2O for 2 h.

2.9.3 Re-screening of *Cv*FAP hit variants at 1 mL scale

Biotransformations were carried out under the same conditions as described in **section 1.2**, but using 10 mg/mL of lyophilized CFE of the respective hit variants and either (*R*)-, or (*S*)-**10a** as substrate and an irradiation time of 4 h. The work-up was carried out as described in **section 1.2** using MTBE (500 µL, containing 10 mM, *n*-dodecane as internal standard) as extraction solvent. Samples were measured on a GC-FID with **method GC-3** as described in **section 7**.

2.10 Reaction engineering

When using lyophilized CFEs the required amounts (10 mg/mL) were weighed into screw cap glass vials (1.5 mL) and rehydrated in Tris·HCl buffer (100 mM, pH 8.5, 700 µL) for 20 min and gently flipped twice after 10 minutes to facilitate rehydration. When using lyophilized whole cells, the cells were rehydrated in Tris·HCl buffer (100 mM, pH 8.5, 700 µL) for 1 h (25 °C, 300 rpm). For the biotransformations with resuspended whole cells, the pellets (270 OD_{600} units) were resuspended in Tris·HCl buffer (100 mM, pH 8.5), creating stock solutions of 80 OD_{600} units/mL. 250–500 µL of resuspended whole cells were used to obtain a final OD_{600}/mL of 20-40 in the reaction, followed by the addition of Tris·HCl buffer (100 mM, pH 8.5) to a volume of 700 µL. This was followed by the addition of the substrate stock (**10a**, 300 µL, 33.3 mM in DMSO) for a final volume of 1.0 mL. The illumination and work-up was carried out as described in **section 1.2** using MTBE (500 µL, containing 10 mM, *n*-dodecane as internal standard) as extraction solvent. Samples were measured on a GC-FID with **method GC-3** as described in **section 7**. Furthermore, a cell dry weight (CDW) determination of the resuspended whole cells was conducted to compare the activity to the lyophilized whole cells. This was done by shock freezing respective volumes used in the biotransformations and lyophilization overnight, followed by its weighting.

3 Computational Details

3.1 System preparation and Molecular Dynamics (MD) simulations:

The crystal structure of *Chlorella variabilis* Fatty acid Photodecarboxylase (*Cv*FAP) in complex with flavin adenine dinucleotide (FAD) cofactor and palmitic acid (PDB ID: 5NCC) was used as the starting point for the preparation of system for Molecular Dynamics (MD) simulations of Wild-Type (WT) enzyme and the engineered *Cv*FAP M1, M2, and M4 variants. The FAD cofactor was retained in the same orientation as in PDB 5NCC, while the palmitic acid was removed from the active site. The mutations corresponding to each variant were introduced using the mutagenesis tool implemented in the PyMOL Molecular Graphics System, Version 3.0 Schrödinger, LLC. Finally, the substrate (*R*)-**10a** was manually docked to match the position of the palmitic acid in the X-ray crystal structure. This (*R*)-**10a** substrate- and cofactor-bound structure was used as starting point for MD simulations. The protonation states of protein residues were assigned using the H++ webserver.[3-5]

Molecular Dynamics (MD) simulations were performed using the GPU code (*pmemd*) of the AMBER 22 package.[6] The force field parameters for the (*R*)-**10a** molecule were obtained using the *antechamber* module of AMBER22, employing the general AMBER force field (GAFF)[7] for atom types and parameters. The partial atomic charges of (*R*)-**10a** were assigned according to the Restrained Electrostatic Potential (RESP)[8] model, which was calibrated to fit the electrostatic potential calculated at the B3LYP/6-31G(d) theoretical level. The calculation of these charges was performed using the Gaussian16 software,[9] based on the Merz-Singh-Kollman scheme.[10,11] Each system under study was subsequently immersed in a pre-equilibrated truncated cuboid box of TIP3P[12] water molecules with a 10 Å buffer using the leap module. The systems were neutralized by the addition of explicit counter ions (Na^+ and Cl^-). The force-field used to describe protein residues was the well-established Amber ff14SB force field, incorporating the Stony Brook modifications.

First, an energy minimization of 10,000 steps was performed on the full system. Then, the systems were gradually heated using six 20 ps steps, increasing the temperature by 50 K for each step (0–300 K) under constant-volume and periodic-boundary circumstances. Bonds involving hydrogen were constrained with the SHAKE[13,14] algorithm. The particle-mesh-Ewald (PME) approach[15-17] has been used to model long-range electrostatic effects. Lennard-Jones and electrostatic interactions were subjected to a 10 Å cutoff. The temperature was controlled and equalized using the Langevin equilibration scheme.[18] During the heating stages, the time step has been fixed at 2 fs to allow for possible inhomogeneities to self-adjust. Then, each system was equilibrated under the NPT ensemble for 12 ns at a constant pressure of 1 atm with a 2-fs time step using the Berendsen barostat. Once the systems

were equilibrated, conventional MD trajectories at constant volume and temperature (300 K) were performed.[19]

In this study, MD simulations were carried out for the WT *Cv*FAP and M1, M2, and M4 variants, each complexed with the (*R*)-**10a** molecule positioned within its active site in the presence of the FAD cofactor. Specifically, for the WT enzyme and the M1 variant, three independent replicas of 500 ns were performed. The M2 and M4 variants were investigated with a more extensive sampling, consisting of six independent 500 ns of MD replicas for each system.

The computational analysis and processing of all resulting MD trajectories were executed through the *cpptraj* module,[20] which is part of the AmberTools utilities.

3.2 Quantum Mechanics Density Functional Theory (DFT) calculations:

Computational studies based on Density Functional Theory (DFT) were conducted using the Gaussian16 software package.[9] Truncated computational models for the different steps of the reaction were employed to study the reaction pathways. This model (**M-(R)-10a**) includes full substrate (*R*)-**10a** (or related species along the reaction pathway), the guanidinium group of Arg[451] and the isoalloxazine ring of the FAD cofactor (**Figure S2**). Geometry optimizations and vibrational frequency analyses were executed using the (U)wB97XD functional[21] in conjunction with the 6-31+G(d,p) basis set[22,23] on all atoms. Transition states (TS) were verified to possess one imaginary frequency, which corresponded to the intended reaction coordinate. Furthermore, all stationary points, whether minima or first-order saddle points (TS), were confirmed by subsequent vibrational frequency analysis. Intrinsic Reaction Coordinate (IRC) calculations were also performed to ensure that each optimized transition state linked the expected reactant and product structures with the local quadratic approximation (LQA)[24,25] for the predictor step. Thermodynamic parameters were calculated under standard conditions of 1 atm and 298.15 K. To approximate the dielectric environment within the polar reaction site (composed by the flavin, Arg[451], and (*R*)-**10a** carboxylate functional group) of enzyme's active site during the optimization process, a solvent model was incorporated: the SMD (Solvation Model Density)[26] variant of the IEFPCM (Integral Equation Formalism Polarizable Continuum Model), using water as the solvent (ε = 78.3553).

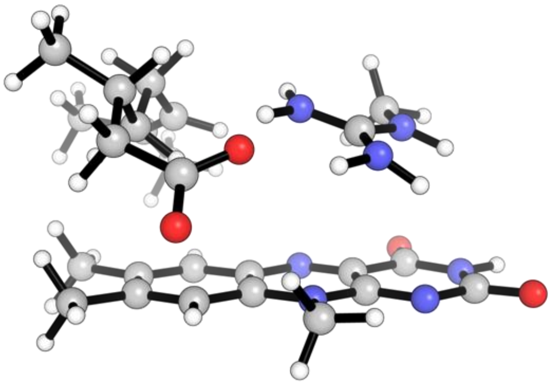

***Figure S2.** Representation of truncated model (**M-(R)-10a**) used for DFT calculations including the full substrate (R)-**10a**, the guanidinium group of Arg^{451} and the isoalloxazine ring of the FAD cofactor.*

Excited-state properties were computed using time-dependent density-functional theory (TD-DFT)[27] at the wB97XD/6-31+G(d,p) level, including the SMD solvation model and water as the solvent.

All figures were generated with PyMOL and Chemcraft.[28] All computational data have been deposited in the IOCHEM-BD platform (www.iochem-bd.org)[29] under the following DOI: https://doi.org/10.19061/iochem-bd-4-96,[30] to facilitate data exchange and dissemination, according to the FAIR principles of OpenData sharing.

4 Supplementary Computational Data

4.1 DFT calculations

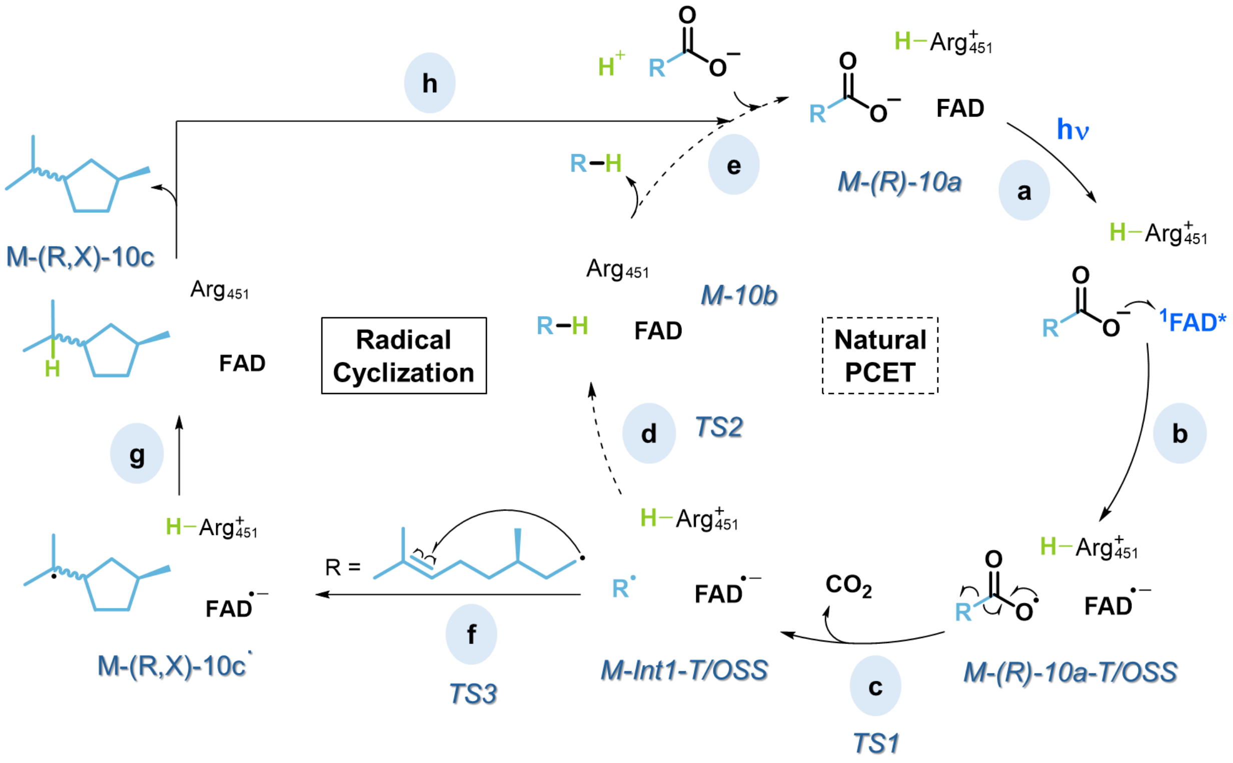

Figure S3. *Proposed photobiocatalytic decarboxylation and radical cyclisation mechanisms of substrate (R)-**10a** catalyzed by CvFAP and its engineered variants. Schematic illustration of the computationally investigated reaction pathways, including the native fatty acid decarboxylation mechanism leading to the linear alkane **10b** product (R-H in the figure, formed following steps a-b-c-d-e) and the intramolecular decarboxylative radical cyclization pathway (steps a-b-c-f-g-h). Truncated active site models (**M-(R)-10a** and related species) were used to computationally investigate the steps suggested for this mechanism. T denotes triplet states and OSS open-shell singlet states. The notation (R,X) indicates the possible formation of (1R,3R)- or (1S,3R)-**10c** cyclized products, whereas **M-10b** corresponds to the linear product. ^{1}FAD* denotes the photoexcited singlet state of flavin adenine dinucleotide (FAD) cofactor.*

Step a

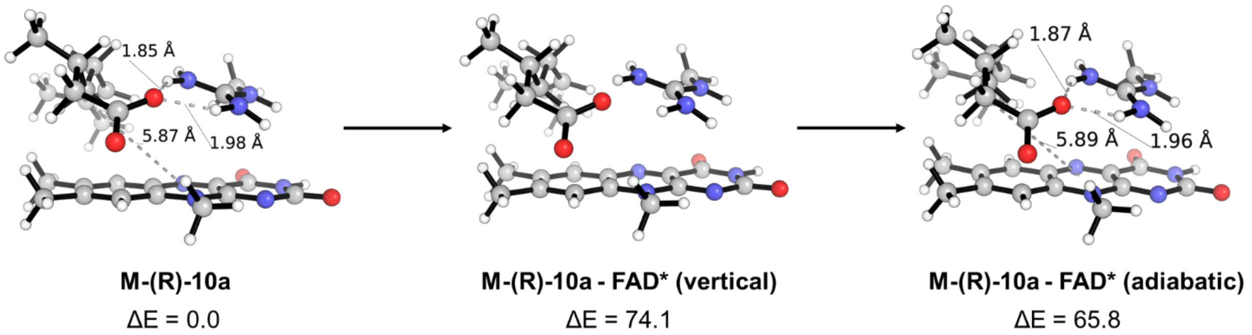

Figure S4. *Photoexcitation of FAD (step a). From left to right, (i) ground-state (S_0) optimized geometry, (ii) singlet S_1 excited-state vertical excitation energy from the ground-state geometry, and (iii) adiabatically optimized S_1 excited-state geometry. Electronic energies obtained at wB97XD/6-31+G(d,p) level using TD-DFT are given in kcal/mol. Key distances are given in Å. All geometries were optimized without any geometric constraint.*

To confirm that the computed excitation corresponds to the relevant photochemically active state, TD-DFT calculations were performed to analyze the electronic transitions involved in the vertical excitation. As shown in **Figure S5**, the dominant excitation corresponds to transitions from orbitals 133 (HOMO-1) and 134 (HOMO) to orbital 135 (LUMO), which is localized on the FAD cofactor. This confirms that the photoexcitation is correctly described as an FAD-centered electronic transition.

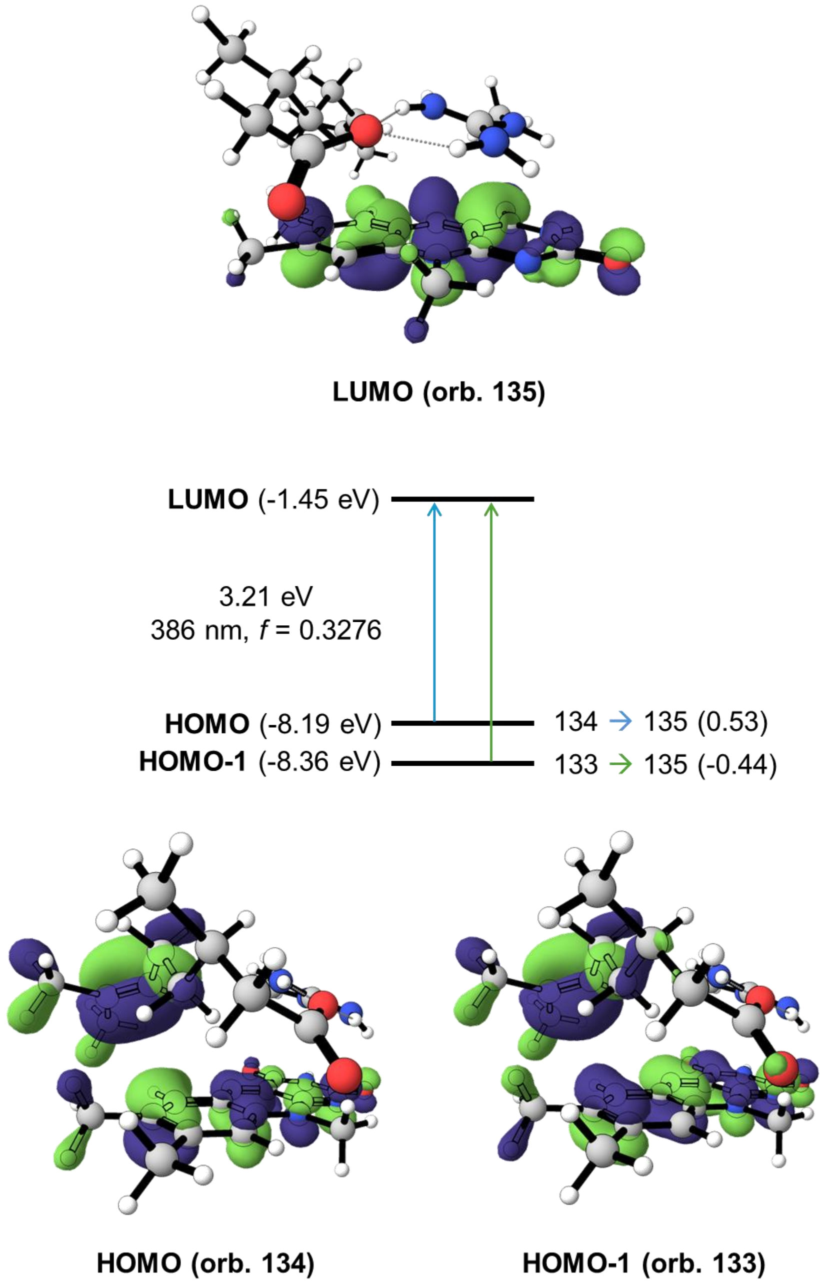

Figure S5. *Molecular orbitals and electronic transitions involved in the photoexcitation of FAD (step a), obtained from the vertical TD-DFT excitation of the* ***M-(R)-10a*** *- FAD system. The dominant excitation corresponds to transitions from orbitals 133 (HOMO−1) and 134 (HOMO) to orbital 135 (LUMO), which is localized on the FAD cofactor.*

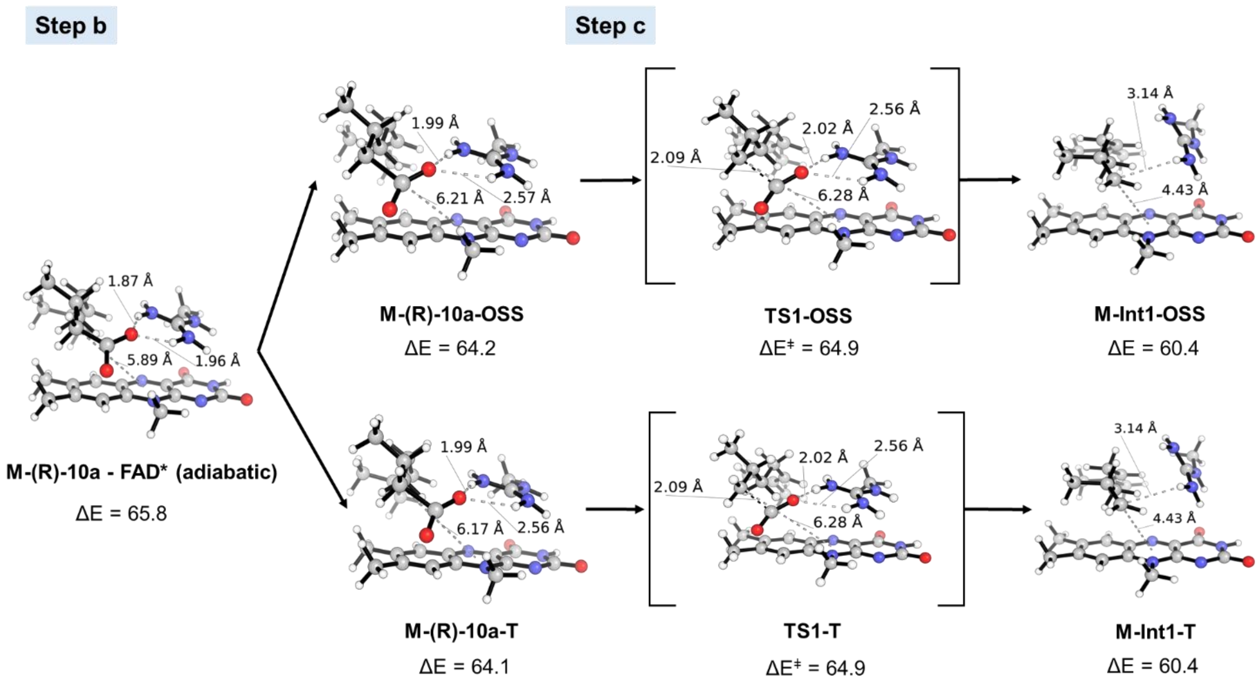

Figure S6. *Decarboxylation of **M-(R)-10a - FAD*** (step b and c). Electronic energies obtained at (u)wB97XD/6-31+G(d,p)/SMD(water) level are given in kcal/mol. Key distances are given in Å. All geometries were optimized without geometric constraints.*

As shown in **Figure S6**, following photoexcitation of the FAD cofactor, either a triplet state or an open-shell singlet (OSS) radical pair can be rapidly formed. Both electronic states lead to a highly favorable decarboxylation step (TS1-OSS = 0.7 kcal/mol and TS1-T = 0.8 kcal/mol), yielding the C-terminal primary radical reactive intermediate (**M-Int1**) as the product. The near energetic degeneracy of the OSS and triplet states suggests weak electronic coupling between the unpaired electrons, which is consistent with a spatially separated radical pair.

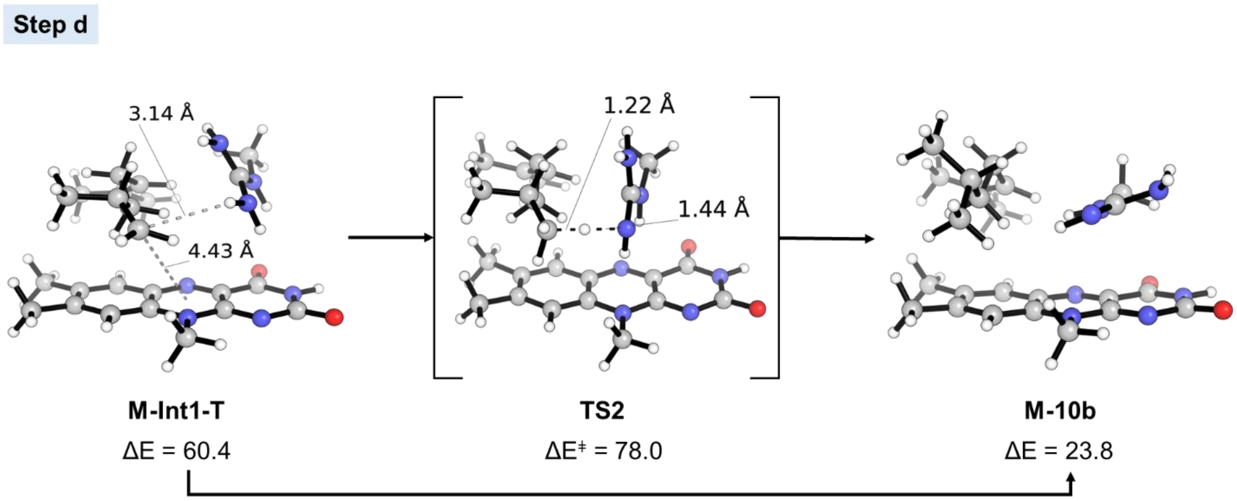

Figure S7. *Proton-coupled electron transfer (PCET) of M-Int1-T consistent with native CvFAP reaction to form linear product **10b** (step d). Electronic energies obtained at (u)wB97XD/6-31+G(d,p)/SMD(water) level are given in kcal/mol. Key distances are given in Å. All geometries were optimized without geometric constraints.*

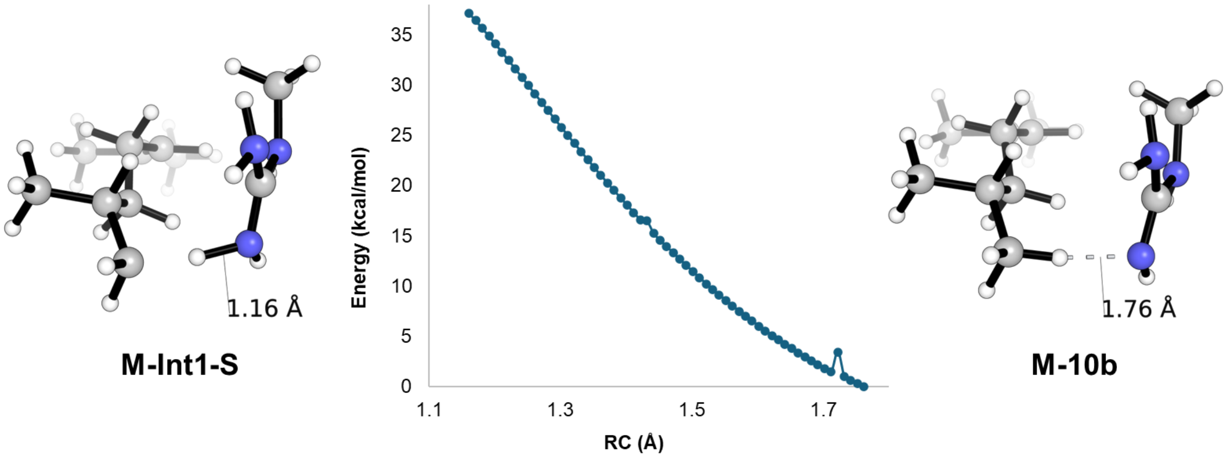

Figure S8. *Potential energy scan (electronic energy) along the hydrogen transfer coordinate from the residue Arg451 to the C terminal primary radical intermediate* ***M-Int1*** *in the singlet state, considering that electron transfer has already occurred. Electronic energies obtained at (u)wB97XD/6-31+G(d,p)/SMD(water) level are given in kcal/mol. Key distances are given in Å.*

This step (step d in **Figure S3**) corresponds to the native PCET pathway, which yields the formation of the thermodynamically favored linear product. This process can, in principle, proceed via either proton transfer followed by electron transfer (PT-ET) or, electron transfer followed by proton transfer (ET-PT). The transition state associated with initial proton-transfer (TS2) along the PT-ET pathway is significantly higher ($\Delta E^{\ddagger}$ =17.6 kcal/mol) than the corresponding barrier for the competing cyclization pathway ($\Delta E^{\ddagger}$ = 5 kcal/mol, **Figure S9**). In contrast, the potential energy scan shown in **Figure S8** demonstrates that when electron transfer occurs first, the subsequent hydrogen transfer becomes barrierless. These results suggest that the native PCET proceeds via ET-PT sequence. These results suggest that the native PCET proceeds either via an ET-PT sequence (**Figure S8**) or a concerted process (**Figure S7**). It is also worth mentioning that within the enzyme environment other species may act as proton-donor.

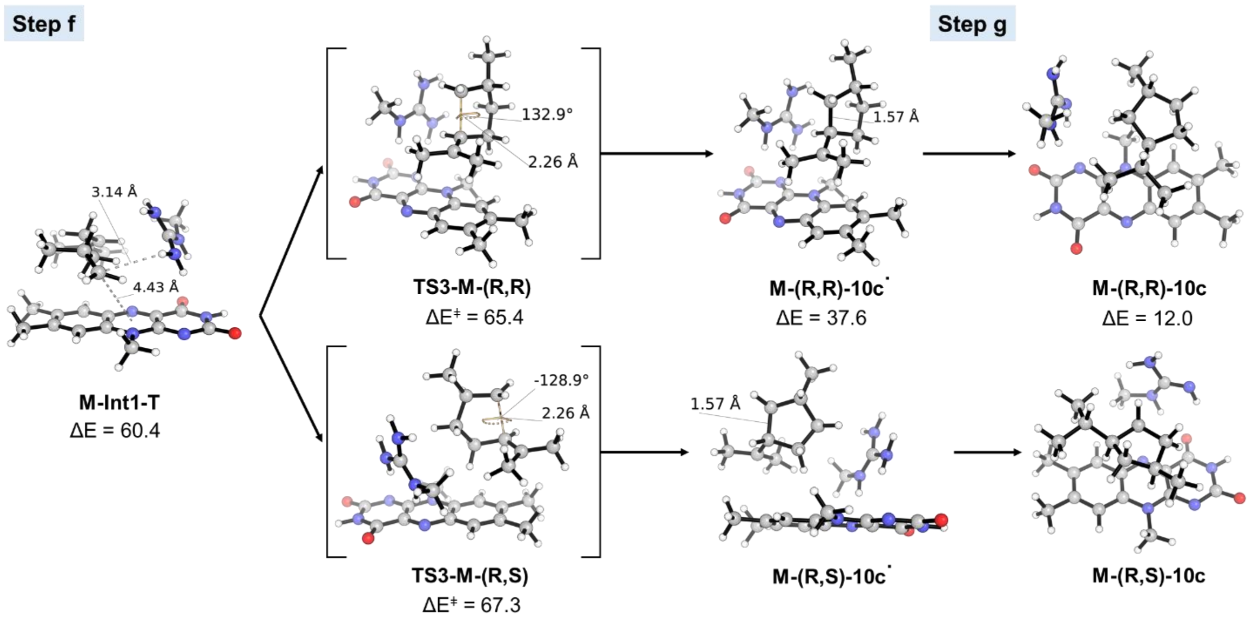

Figure S9. *Radical cyclization of **M-Int1-T** (steps f and g). Electronic energies obtained at (u)wB97XD/6-31+G(d,p)/SMD(water) level are given in kcal/mol. Key distances are given in Å. All geometries were optimized without geometric constraints. Products: (1R,3R)-**10c** /(1S,3R)-**10c**.*

Alternatively, the radical intermediate (**M-Int1**) can undergo an intramolecular *exo-trig* radical attack on the double bond leading to the formation of a five-membered cyclic radical (**M-(R,R)-10c**˙ and **M-(R,S)-10c**). This reactive intermediate subsequently undergoes a final spontaneous PCET to form the cyclic product (1*R*,3*R*)-**10c** /(1*S*,3*R*)-**10c**. The calculated activation barriers for this cyclization step ($\Delta E_{R,R}^{‡}$ = 5.0 kcal/mol; $\Delta E_{R,S}^{‡}$ = 6.9 kcal/mol) are low for both enantiomers, therefore, indicating that both pathways are energetically accessible, showing no clear enantiopreference in the absence of the protein active site environment.

4.2 Molecular dynamics simulations of (*R*)-**10a** substrate preorganization and reaction pathway preference in *Cv*FAP variants.

4.2.1 General comparison of *Cv*FAP WT, M1, M2 and M4 variants.

*4.2.1.1 Substrate preorganization: FAD - Substrate (R)-**10a** relative distance in CvFAP WT, M1, M2 and M4 variants.*

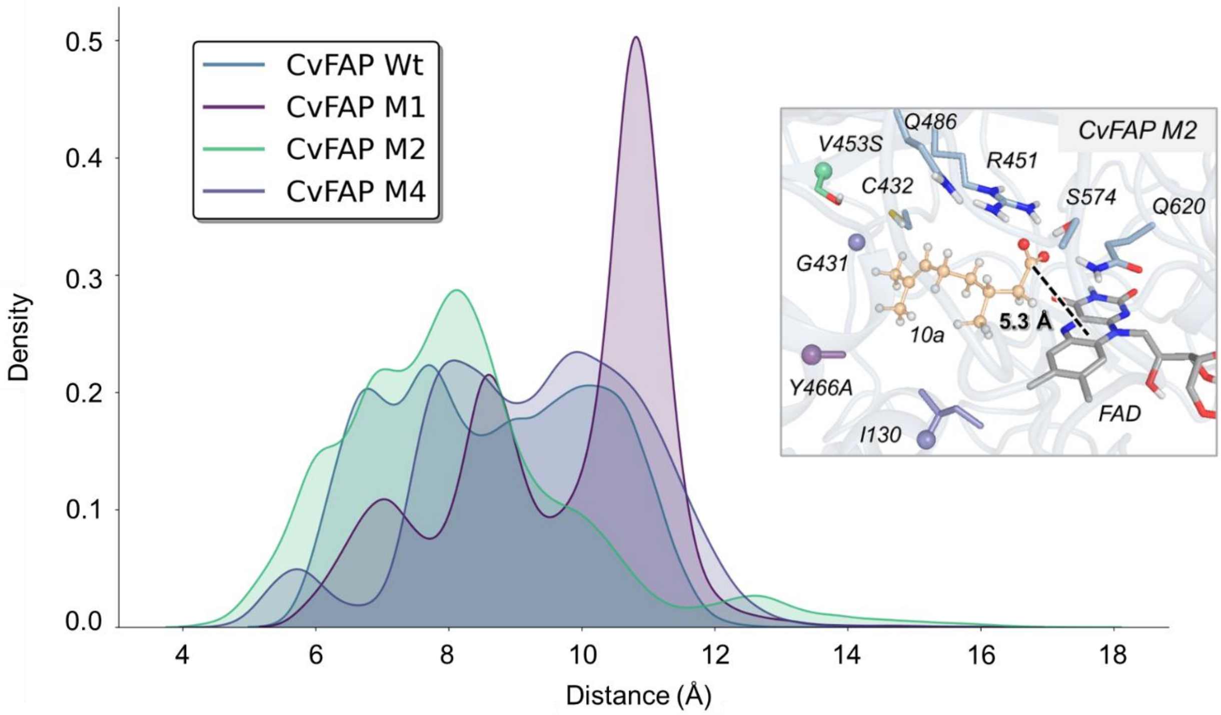

Figure S10. *Probability density distributions of the distance between the carboxylate group of substrate (R)-**10a** and the FAD cofactor obtained from MD simulations of CvFAP WT, M1, M2, and M4 variants. The distance was measured between the carbon atom of the substrate carboxylate group and the center of mass of the two nitrogen atoms of the central isoalloxazine ring of the FAD cofactor. A representative snapshot from the CvFAP M2 simulation illustrating the substrate preorganization within the active site is shown. The FAD cofactor is depicted in gray; conserved (non-mutated) residues are shown in blue; mutations introduced in M1 are shown in purple, in M2 in green, and in M4 in light purple. All distances are in Å.*

These results show that in the WT enzyme and in variant M1, substrate (*R*)-**10a** predominantly samples conformations located far from the FAD cofactor, indicating weak stabilization within the active site. In contrast, mutations introduced in variant M2 favour closer positioning of the substrate relative to the FAD cofactor, stabilizing catalytically relevant conformations that are competent for photoinduced decarboxylation and, as discuss below, subsequent radical cyclization. In variant M4, the introduction of I130K mutation alters the substrate binding mode by anchoring the carboxylate group in a mid-region of the active site, resulting in slightly increased average substrate-FAD distances compared to M2 while still maintaining values compatible with efficient photoactivation. As shown below, this shift

disfavours the native PCET pathway while preserving substrate folding and geometric preorganization for intramolecular C-C bond formation, driving the reaction toward the cyclization pathway.

*4.2.1.2 Substrate preorganization: R451 - Substrate (R)-**10a** relative distance in CvFAP WT, M1, M2 and M4 variants.*

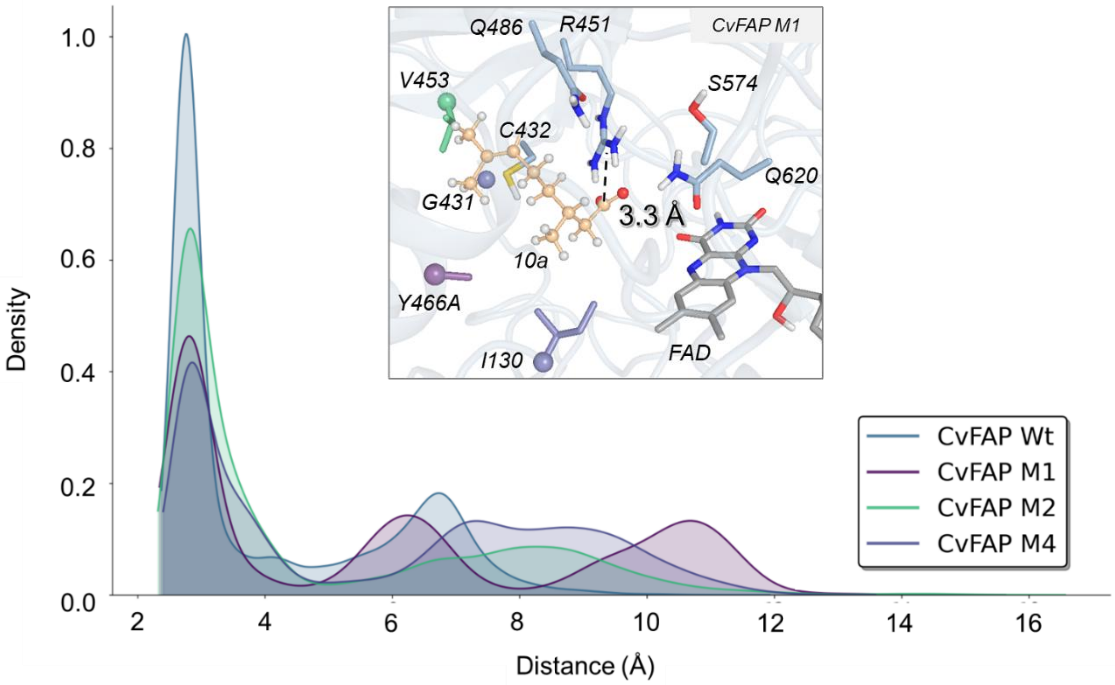

Figure S11. *Probability density distributions of the distance between the carboxylate group of substrate (R)-**10a** and residue Arg451 obtained from MD simulations of CvFAP WT, M1, M2, and M4 variants. The distance was measured between the carbon atom of the substrate carboxylate group and the carbon of the guanidinium group of Arg451. A representative snapshot from the CvFAP M1 simulation illustrating the analyzed interaction within the active site is shown. In the snapshot, the FAD cofactor is depicted in gray; conserved (non-mutated) residues are shown in blue; mutations introduced in M1 are shown in purple, in M2 in green, and in M4 in light purple. All distances are in Å.*

In all variants, the substrate frequently samples short distances to Arg451, indicating strong salt-bridge interactions between the carboxylate group and the guanidinium moiety. These close contacts are consistent with binding modes that favour the native PCET pathway, in which Arg451 can play a role as proton donor. The M4 variant exhibits a distribution shifted toward larger values, reflecting a reduced persistence of close Arg451-substrate interactions. As shown below, the introduction of I130K mutation contributes to this effect by establishing competing salt-bridge interactions with substrate (*R*)-**10a**, partially unbinding the substrate from Arg451 and, consequently, disfavouring the native PCET pathway. Overall, these results support a model in which the I130K mutation introduced in the M4 variant weakens Arg451-mediated stabilization of PCET-competent conformations while promoting substrate geometries favourable for radical cyclization.

4.2.1.3 Substrate preorganization: Stereoselectivity of (R,X)-**10c** formation in CvFAP WT, M1, M2 and M4 variants.

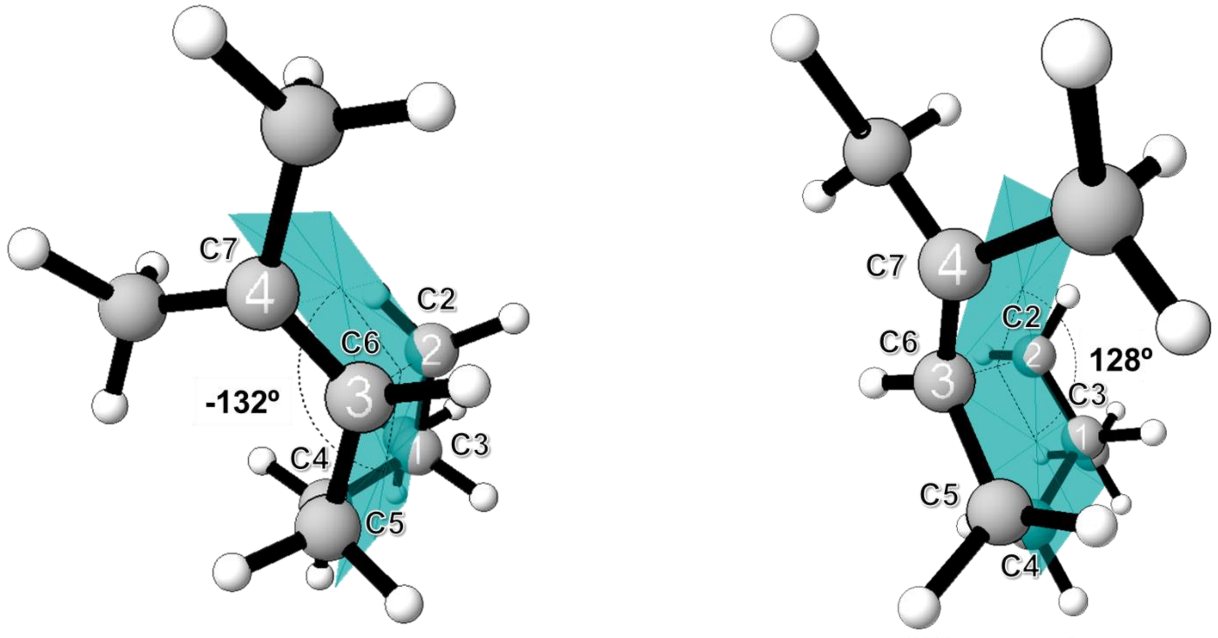

(R,S)-10c **(R,R)-10c**

Figure S12. *DFT optimized transition-state (TS) geometries for C-C bond formation leading to the two cyclized diastereomers, (1S,3R)-**10c** and (1R,1R)-**10c** (wB97XD/6-31+G(d,p)/SMD(water) level of theory). The highlighted dihedral angle (∠(1-2-3-4-5)) defining the relative orientation of the reacting carbon centers is shown for each TS and serves as a stereochemical descriptor for distinguishing between the two cyclization pathways. These TS dihedral values are used as reference points for comparison with dihedral angle distributions obtained from MD simulations, see below.*

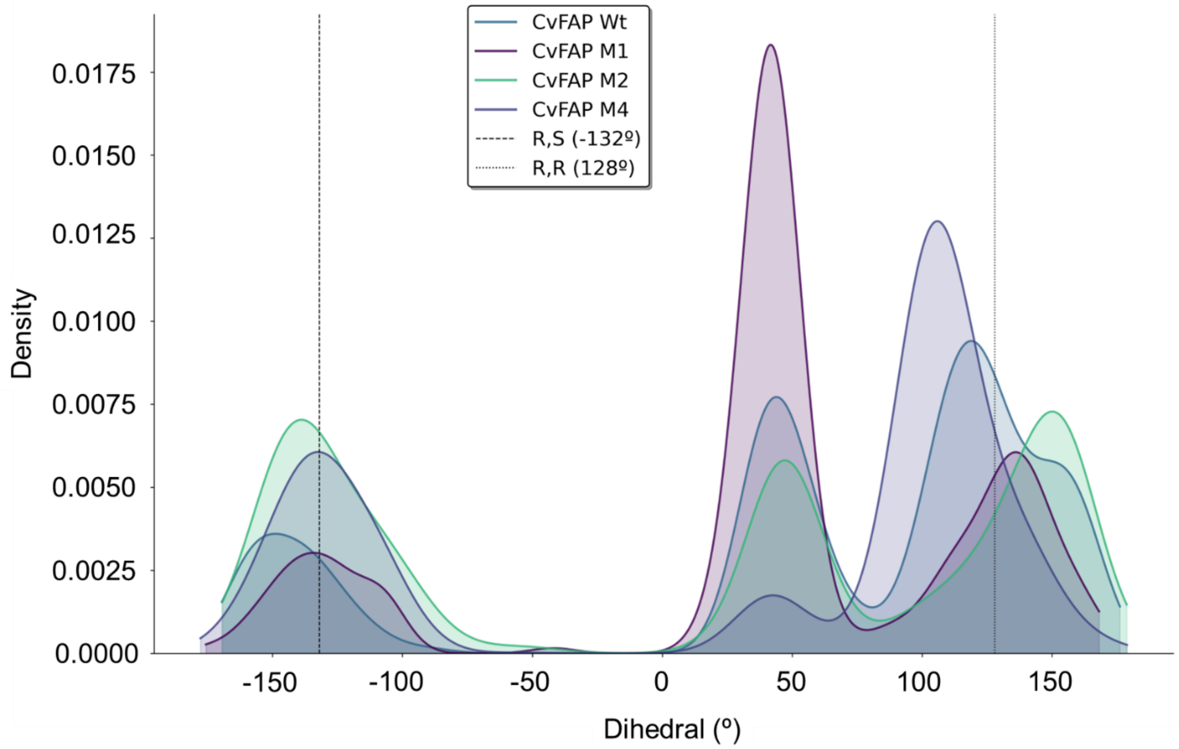

Figure S13. *Probability density distributions of the reactive dihedral angle describing the enantioselectivity during the cyclization and C-C bond formation for substrate (R)-**10a**, obtained from MD simulations of CvFAP WT, M1, M2, and M4 variants. Only conformations in which the distance between the reacting carbon atoms (C2 and C6, for atom numbering see **Figure S12**) is below 3.5 Å were considered. The optimal TS dihedral angles for formation of the (1S,3R)-**10c** and (1R,1R)-**10c** diastereomers, computed at the DFT level, are shown as dashed lines.*

The dihedral angle distributions obtained from accumulated MD trajectories provide a measure of substrate geometric preorganization for intramolecular C-C bond formation. These distributions indicate a preference toward dihedral values consistent with the formation of the (1*R*,1*R*)-**10c** product as most populated dihedral regions are found around the optimal TS value for formation of the (1*R*,1*R*)-**10c** diastereomer rather than the (1*S*,3*R*)-**10c** product.

4.2.2 Wild-type (WT) *Cv*FAP MD Simulations:

*4.2.2.1 Substrate preorganization: FAD – Substrate (R)-**10a** relative distance in CvFAP WT.*

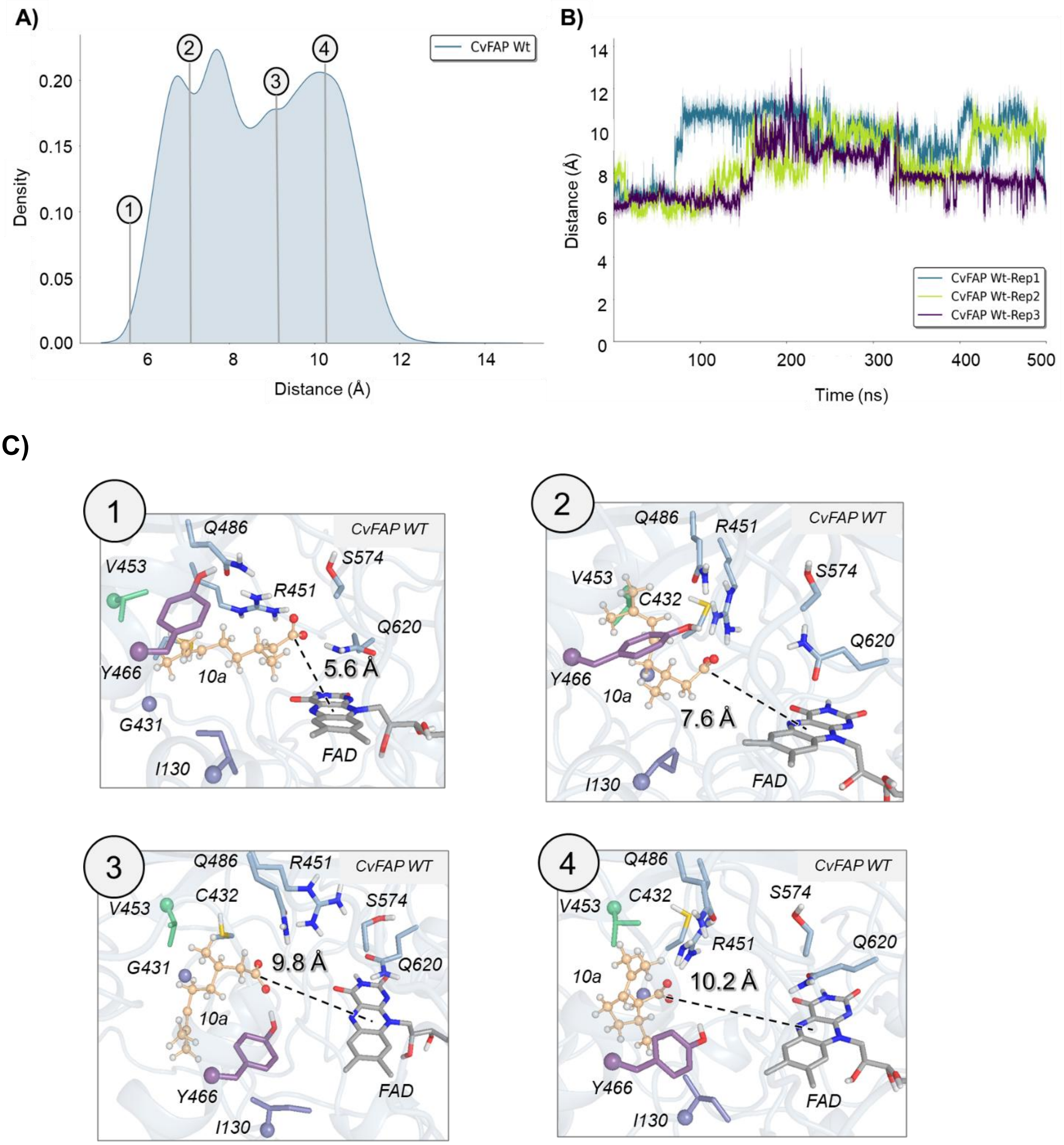

Figure S14. A) *Probability density distributions of the distance between the carboxylate group of substrate (R)-**10a** and the FAD cofactor obtained from MD simulations of CvFAP WT. The distance was measured between the carbon atom of the substrate carboxylate group and the center of mass of the two nitrogen atoms of the central isoalloxazine ring of the FAD cofactor.* ***B)*** *Distance vs. time plot describing the interaction between the carboxylate group of (R)-**10a** and the FAD central isoalloxazine ring obtained from MD simulations (3 replicas of 500 ns for each) of CvFAP WT.* ***C)*** *Four representative snapshots from the CvFAP WT simulations illustrating the substrate preorganization within the active site. The FAD cofactor is depicted in gray; conserved (non-mutated) residues are shown in blue; mutations introduced in M1 are shown in purple, in M2 in green, and in M4 in light purple. All distances are in Å and simulation times in ns.*

*4.2.2.2 R451 – Substrate (R)-**10a** relative distance in CvFAP WT.*

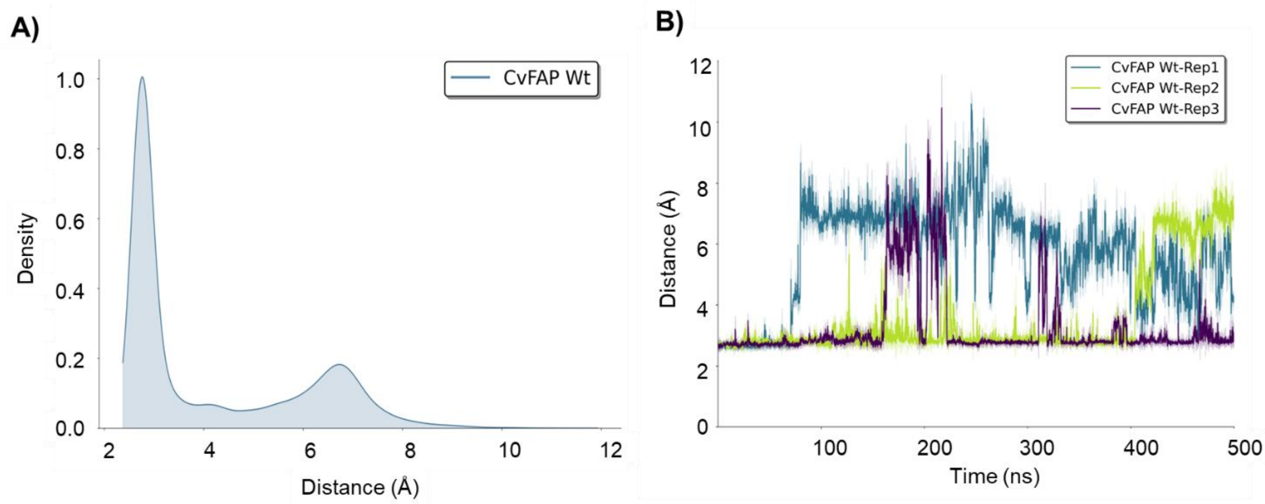

***Figure S15. A)** Probability density distributions of the distance between the carboxylate group of substrate (R)-**10a** and residue Arg451 obtained from MD simulations of CvFAP WT. The distance was measured between the carbon atom of the substrate carboxylate group and the carbon of the guanidinium group of Arg451. **B)** Distance vs. time plot describing the interaction between the carboxylate group of (R)-**10a** – R451 obtained from MD simulations (3 replicas of 500 ns for each) of CvFAP WT. All distances are in Å.*

*4.2.2.3 Substrate preorganization: Stereoselectivity of (R,X)-**10c** formation in CvFAP WT.*

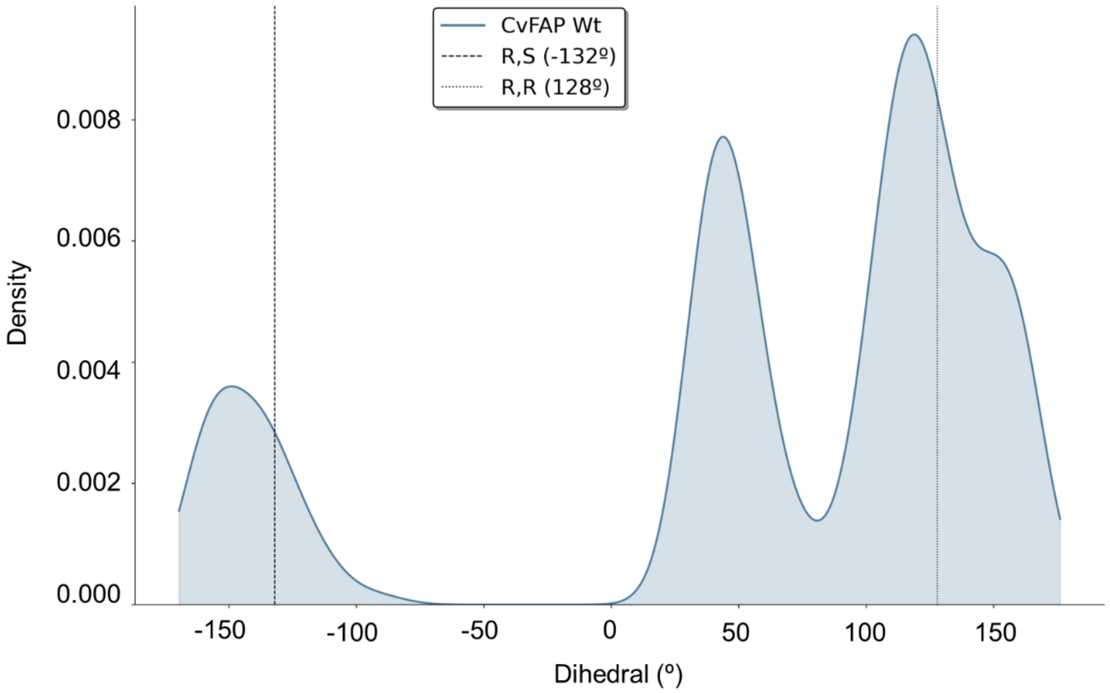

***Figure S16.** Probability density distributions of the reactive dihedral angle defining C-C bond formation for cyclization of substrate (R)-**10a**, obtained from MD simulations of CvFAP WT. Only conformations in which the distance between the reacting carbon atoms (C2 and C6, for atom numbering see **Figure S12**) is below 3.5 Å were considered. The optimal TS dihedral angles for formation of the (1S,3R)-**10c** and (1R,1R)-**10c** diastereomers, computed at the DFT level, are shown as dashed lines.*

These results show that in the WT enzyme, substrate (*R*)-**10a** tends to separate from the FAD cofactor, indicating weak stabilization within the active site. In contrast, the substrate carboxylate group establish more persistent interactions with the guanidinium group of Arg451, forming stable interactions that anchor the substrate away from the flavin. When substrate (*R*)-**10a** transiently samples folded, cyclization-competent conformations (C2–C6 distances below 3.5 Å, for atom numbering see **Figure S12**), the dihedral angle distribution shows some preference toward geometries leading to formation of the (1*R*,3*R*)-**10c** product.

4.2.3 M1 *Cv*FAP MD simulations:

*4.2.3.1 Substrate preorganization: FAD – Substrate (R)-**10a** relative distance in CvFAP M1.*

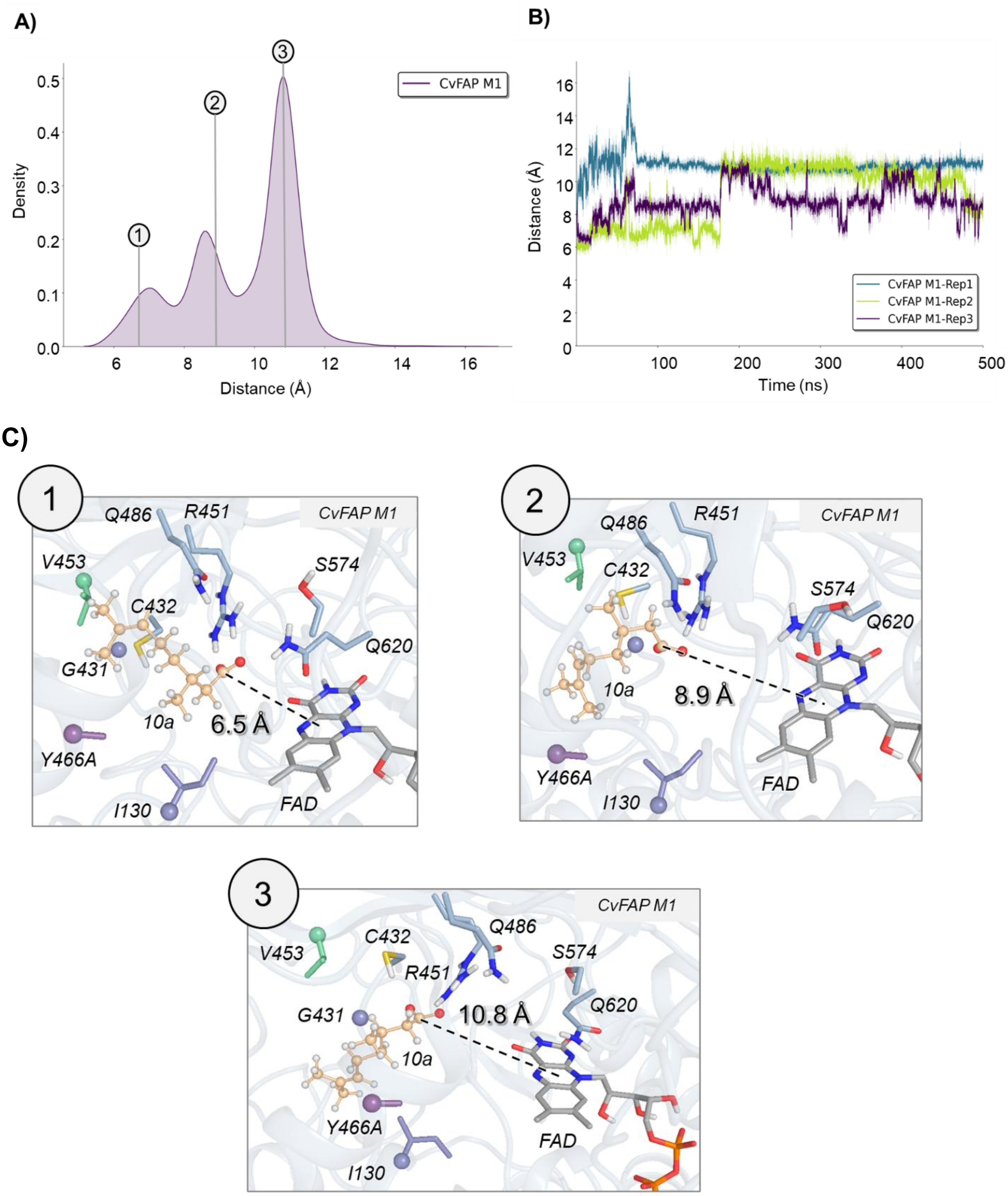

Figure S17. A) *Probability density distributions of the distance between the carboxylate group of substrate (R)-**10a** and the FAD cofactor obtained from MD simulations of CvFAP M1 variant. The distance was measured between the carbon atom of the substrate carboxylate group and the center of mass of the two nitrogen atoms of the central isoalloxazine ring of the FAD cofactor.* ***B)*** *Distance vs. time plot describing the interaction between the carboxylate group of (R)-**10a** and the FAD central isoalloxazine ring obtained from MD simulations (3 replicas of 500 ns for each) of CvFAP M1.* ***C)*** *Three representative snapshots from the CvFAP M1 simulations illustrating the substrate preorganization within the active site. The FAD cofactor is depicted in gray; conserved (non-mutated) residues are shown in blue; mutations introduced in M1 are shown in purple, in M2 in green, and in M4 in light purple. All distances are in Å and simulation times in ns.*

*4.2.3.2 Substrate preorganization: R451 – Substrate (R)-**10a** relative distance in CvFAP M1.*

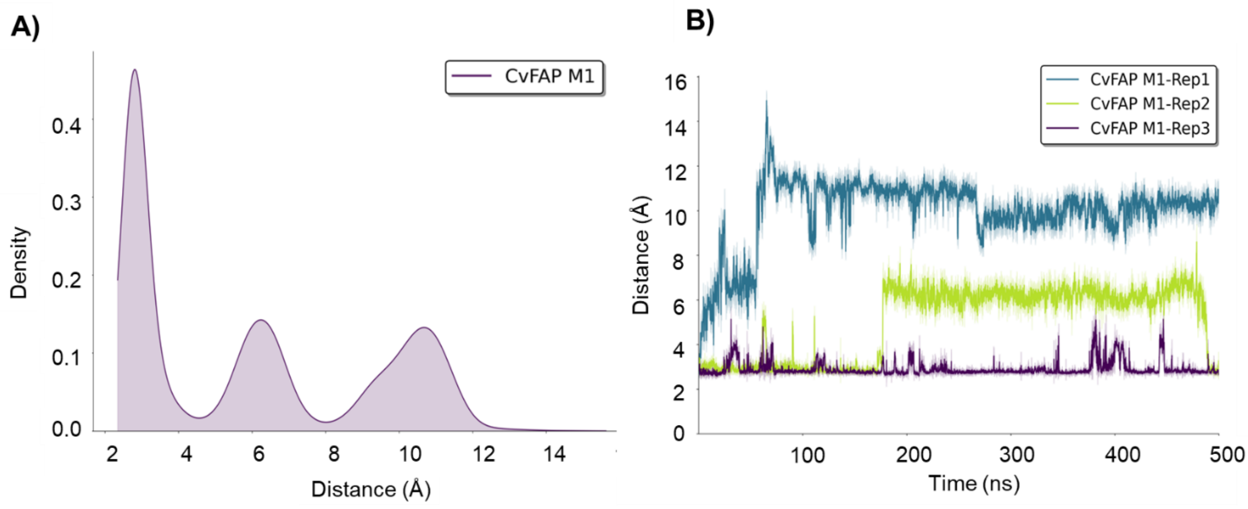

Figure S18. A) *Probability density distributions of the distance between the carboxylate group of substrate (R)-**10a** and residue Arg451 obtained from MD simulations of CvFAP M1. The distance was measured between the carbon atom of the substrate carboxylate group and the carbon of the guanidinium group of Arg451.* ***B)*** *Distance vs. time plot describing the interaction between the carboxylate group of (R)-**10a** – R451 obtained from MD simulations (3 replicas of 500 ns for each) of CvFAP M1. All distances are in Å.*

*4.2.3.3 Substrate preorganization: Stereoselectivity of (R,X)-**10c** in CvFAP M1.*

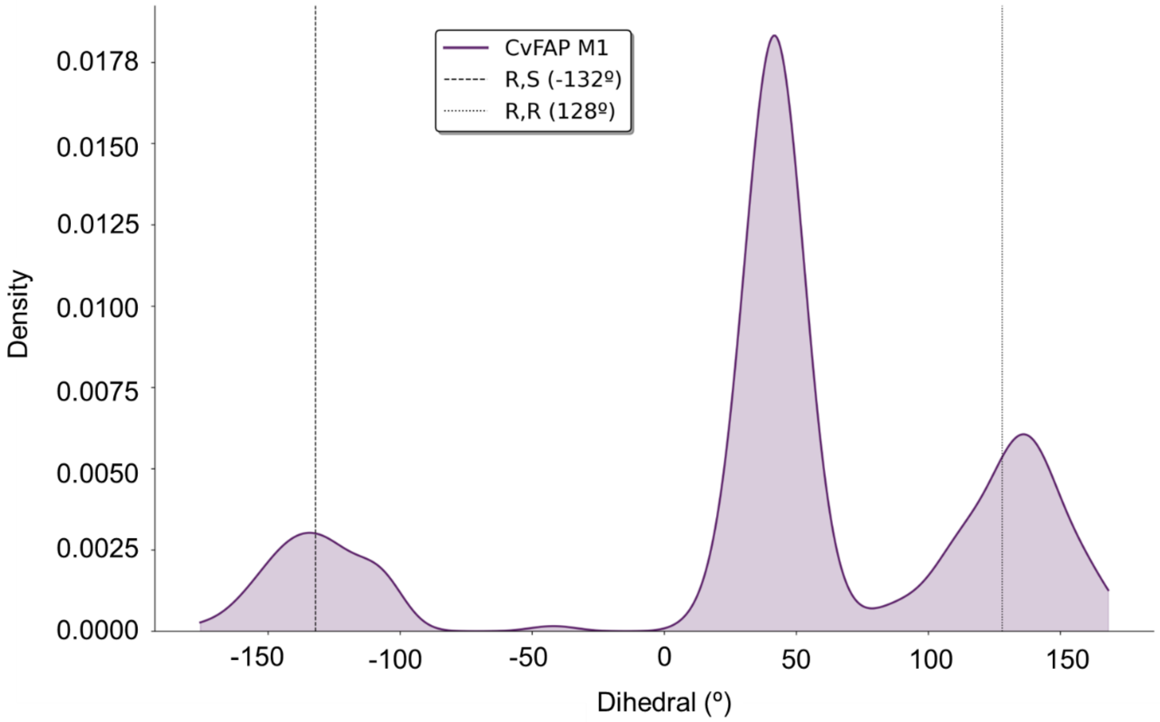

***Figure S19.** Probability density distributions of the reactive dihedral angle defining C-C bond formation for cyclization of substrate (R)-**10a**, obtained from MD simulations of CvFAP M1. Only conformations in which the distance between the reacting carbon atoms (C2 and C6, for atom numbering see **Figure S12**) is below 3.5 Å were considered. The optimal TS dihedral angles for formation of the (1S,3R)-**10c** and (1R,1R)-**10c** diastereomers, computed at the DFT level, are shown as dashed lines.*

These results indicate that in the M1 variant, substrate (*R*)-**10a** tends to separate from the FAD cofactor as observed for the WT enzyme, indicating weak stabilization within the active site. The substrate carboxylate group establish less persistent interactions with the guanidinium group of Arg451 than in the case of the WT enzyme. When substrate (*R*)-**10a** transiently samples folded, cyclization-competent conformations (C2–C6 distances below 3.5 Å, for atom numbering see **Figure S12**), the dihedral angle distribution shows some preference toward geometries leading to formation of the (1*R*,3*R*)-**10c** product.

4.2.4 M2 *Cv*FAP MD simulations:

*4.2.4.1 Substrate preorganization: FAD – Substrate (R)-**10a** relative distance in CvFAP M2.*

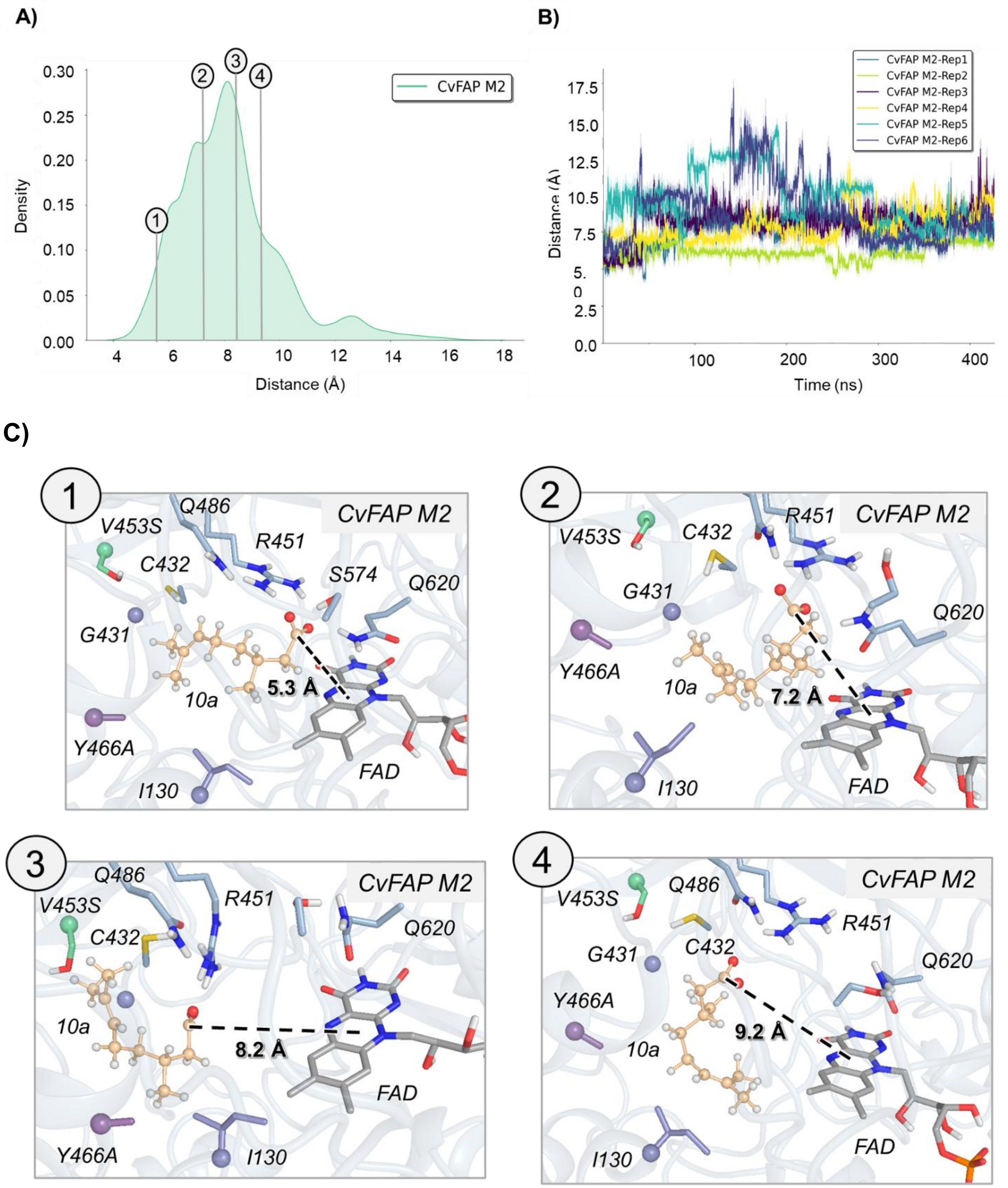

***Figure S20. A)** Probability density distributions of the distance between the carboxylate group of substrate (R)-**10a** and the FAD cofactor obtained from MD simulations of CvFAP M2 variant. The distance was measured between the carbon atom of the substrate carboxylate group and the center of mass of the two nitrogen atoms of the central isoalloxazine ring of the FAD cofactor. **B)** Distance vs. time plot describing the interaction between the carboxylate group of (R)-**10a** and the FAD central isoalloxazine ring obtained from MD simulations (6 replicas of 500 ns for each) of CvFAP M2. **C)** Four representative snapshots from the CvFAP M2 simulations illustrating the substrate preorganization within the active site. The FAD cofactor is depicted in gray; conserved (non-mutated) residues are shown in blue; mutations introduced in M1 are shown in purple, in M2 in green, and in M4 in light purple. All distances are in Å and simulation times in ns.*

*4.2.4.2 Substrate preorganization: R451 – Substrate (R)-**10a** relative distance in CvFAP M2.*

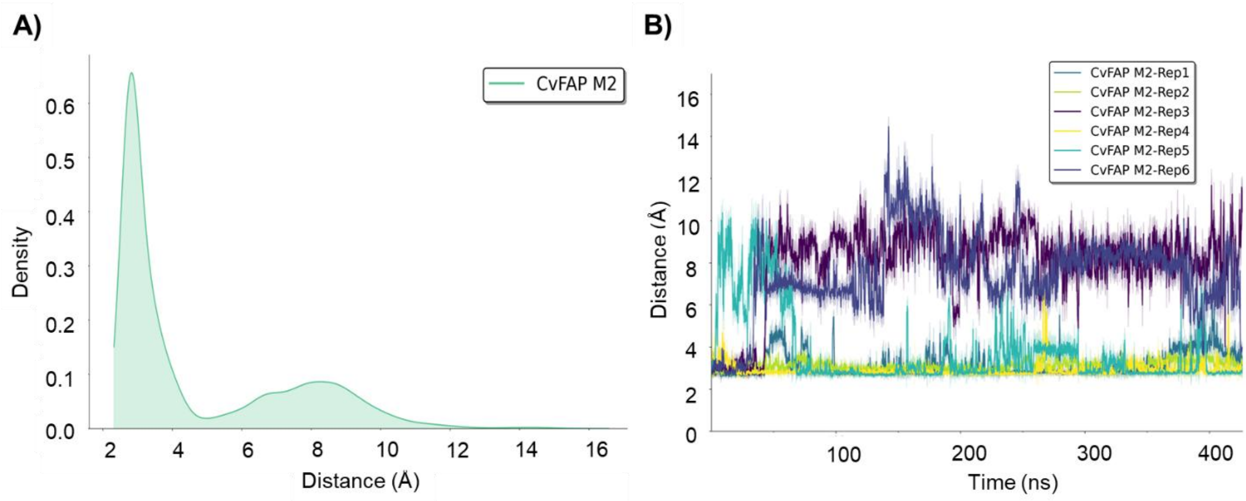

***Figure S21. A)** Probability density distributions of the distance between the carboxylate group of substrate (R)-**10a** and residue Arg451 obtained from MD simulations of CvFAP M2. The distance was measured between the carbon atom of the substrate carboxylate group and the carbon of the guanidinium group of Arg451. **B)** Distance vs. time plot describing the interaction between the carboxylate group of (R)-**10a** – R451 obtained from MD simulations (6 replicas of 500 ns for each) of CvFAP M2. All distances are in Å.*

*4.2.4.3 Stereoselectivity of (R,X)-**10c** in CvFAP M2.*

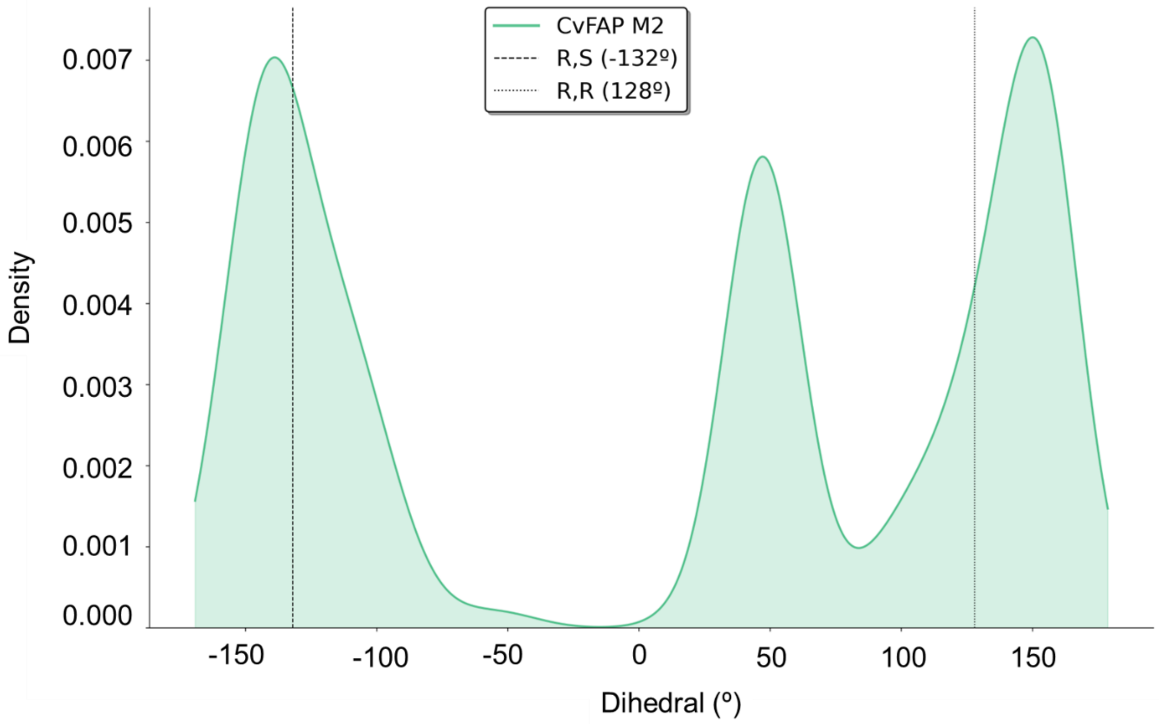

***Figure S22.** Probability density distributions of the reactive dihedral angle defining C-C bond formation for cyclization of substrate (R)-**10a**, obtained from MD simulations of CvFAP M2. Only conformations in which the distance between the reacting carbon atoms (C2 and C6, for atom numbering see **Figure S12**) is below 3.5 Å were considered. The optimal TS dihedral angles for formation of the (1S,3R)-**10c** and (1R,1R)-**10c** diastereomers, computed at the DFT level, are shown as dashed lines.*

*4.2.4.4 Substrate preorganization: Substrate (R)-**10a** prefolding CvFAP M2 and interactions with I130.*

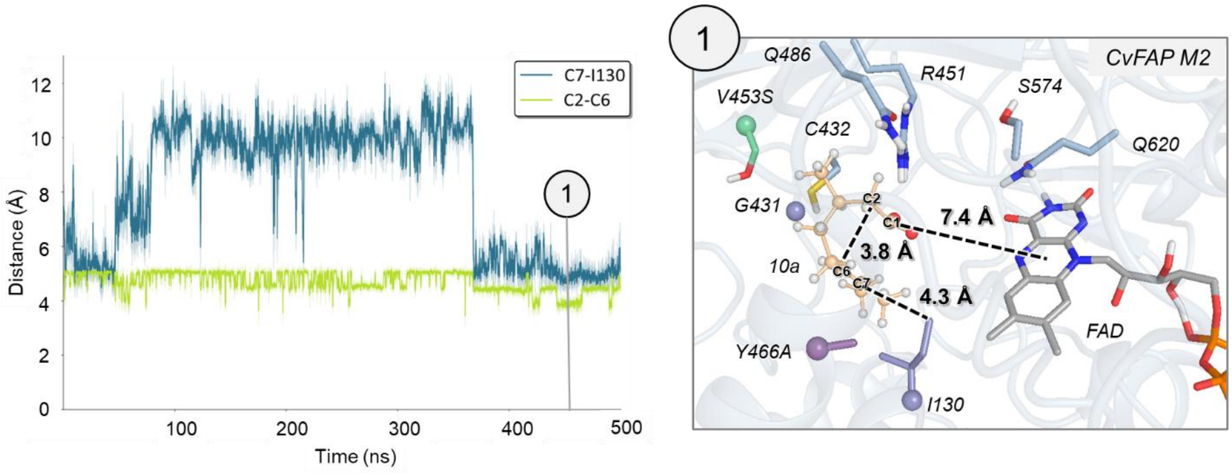

***Figure S23.** Distance vs. time plot describing the interaction between the C7 of (R)-**10a** and the delta carbon (C_δ) of I130 and between the C2 of (R)-**10a** and the C6 of (R)-**10a** (for atom numbering see **Figure S12**). Representative snapshot extracted from CvFAP M2 variant MD simulations showing the prefolding state of substrate (R)-**10a** and the interaction with I130. All distances are in Å and simulation time in ns.*

The mutations introduced in variant M2 favour closer positioning of the substrate relative to the FAD cofactor, stabilizing catalytically relevant conformations that are competent for photoinduced

decarboxylation and subsequent radical cyclization. The interaction with Arg451 is partially retained, allowing decarboxylation while not fully anchoring the substrate away from the flavin. Importantly, analysis of cyclization-competent pre-folded poses reveals a hydrophobic contact between the terminal region of the alkyl chain and residue I130, which stabilizes folded substrate geometries and enhances geometric preorganization for radical attack onto the C=C bond. Consistent with this preorganization, the dihedral angle distribution shows an increased bias toward geometries leading to formation of the (1*R*,3*R*)-**10c** product, although stereocontrol remains moderate compared to the more evolved M4 variant (see below).

4.2.5 M4 *Cv*FAP MD simulations:

*4.2.5.1 Substrate preorganization: FAD – Substrate (R)-**10a** relative distance in CvFAP M4.*

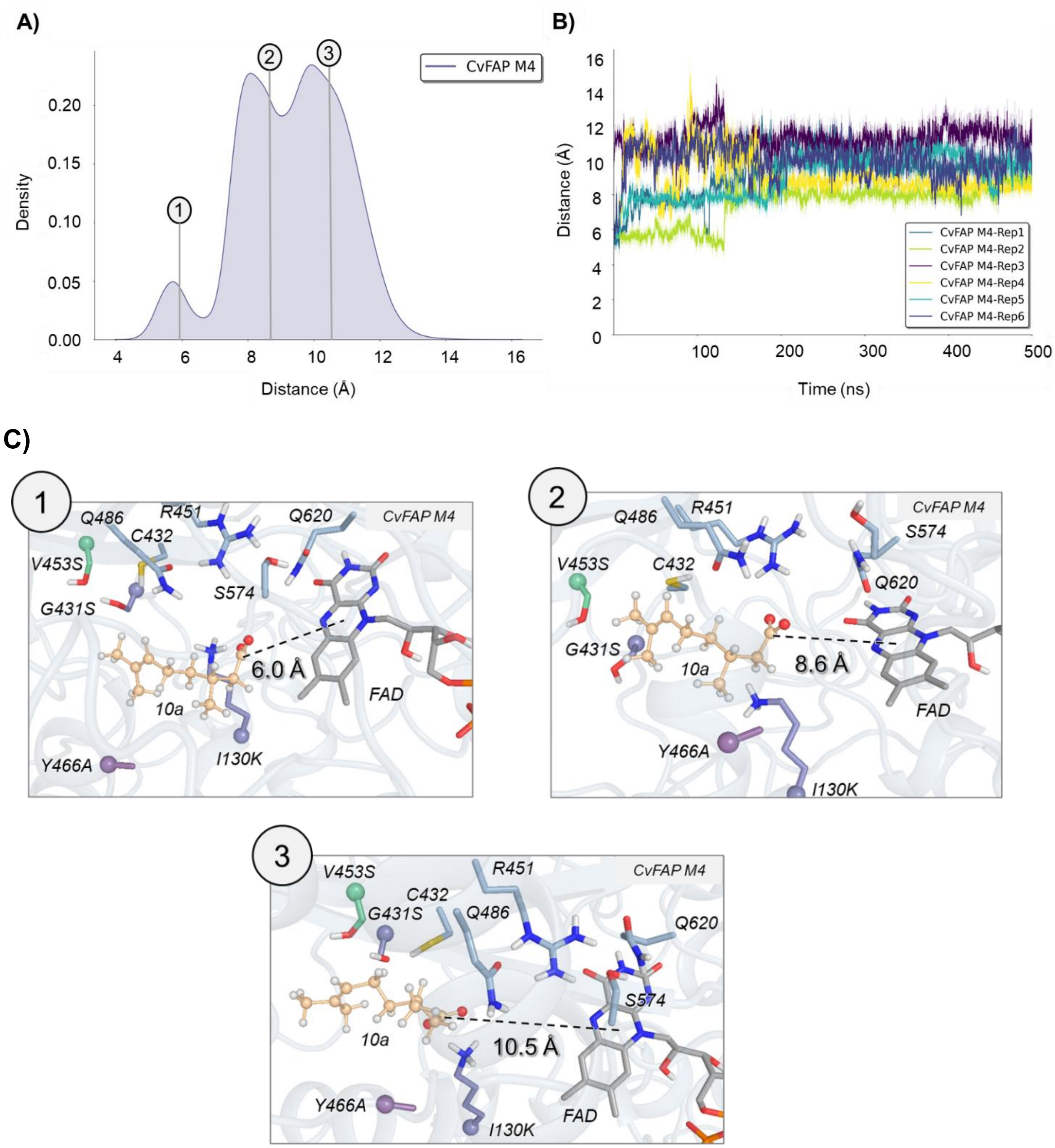

Figure S24. A) *Probability density distributions of the distance between the carboxylate group of substrate (R)-**10a** and the FAD cofactor obtained from MD simulations of CvFAP M4 variant. The distance was measured between the carbon atom of the substrate carboxylate group and the center of mass of the two nitrogen atoms of the central isoalloxazine ring of the FAD cofactor.* ***B)*** *Distance vs. time plot describing the interaction between the carboxylate group of (R)-**10a** and the FAD central isoalloxazine ring obtained from MD simulations (6 replicas of 500 ns for each) of CvFAP M4.* ***C)*** *Three representative snapshots from the CvFAP M4 simulations illustrating the substrate preorganization within the active site. The FAD cofactor is depicted in gray; conserved (non-mutated) residues are shown in blue; mutations introduced in M1 are shown in purple, in M2 in green, and in M4 in light purple. All distances are in Å and simulation times in ns.*

4.2.5.2 *Substrate preorganization: R451 – Substrate (R)-**10a** and I130K – Substrate (R)-**10a** relative distance in CvFAP M4.*

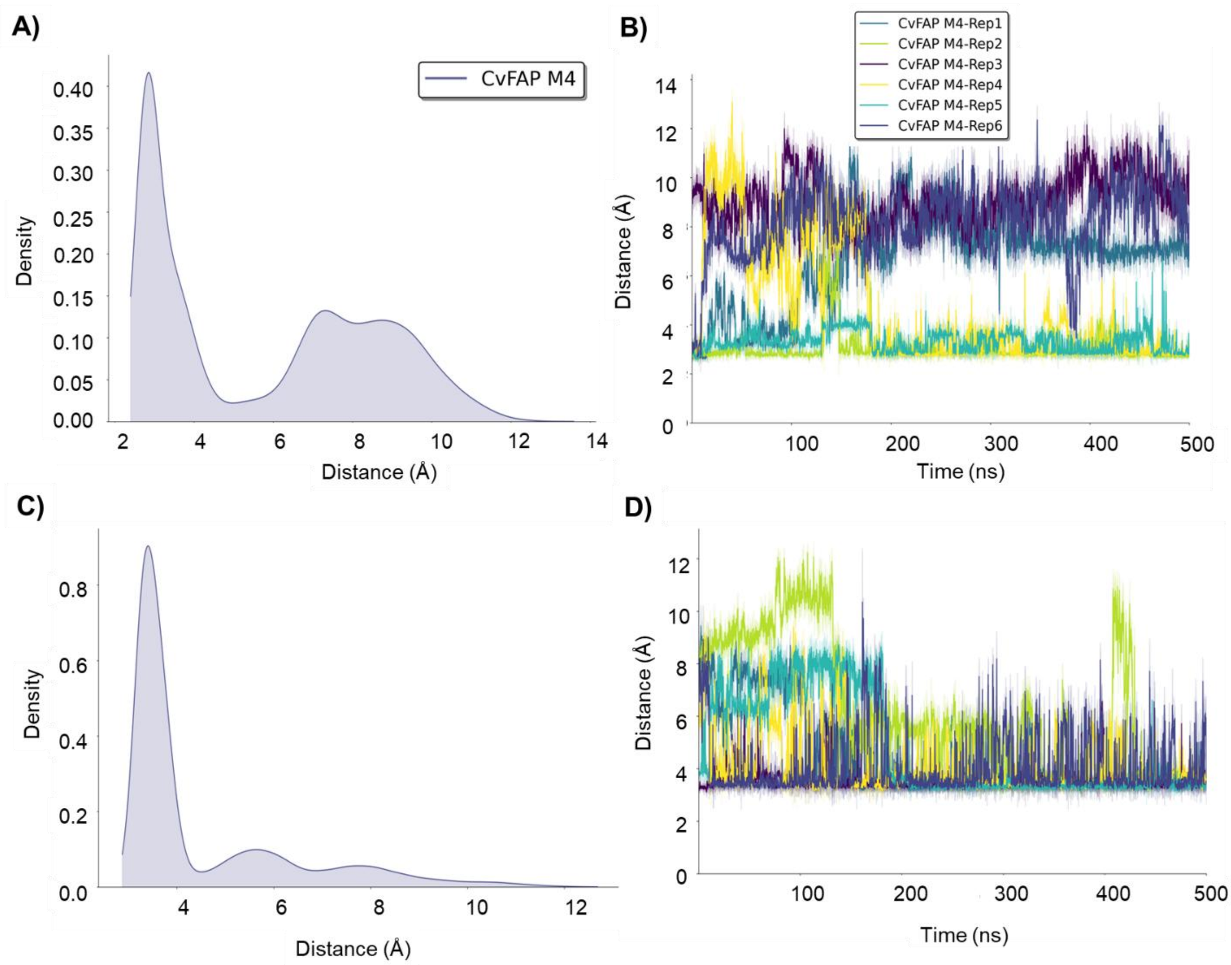

Figure S25. A) *Probability density distributions of the distance between the carboxylate group of substrate (R)-**10a** and residue Arg451 obtained from MD simulations of CvFAP M4. The distance was measured between the carbon atom of the substrate carboxylate group and the carbon of the guanidinium group of Arg451.* ***B)*** *Distance vs. time plot describing the interaction between the carboxylate group of (R)-**10a** – R451 obtained from MD simulations (6 replicas of 500 ns for each) of CvFAP M4. All distances are in Å.* ***C)*** *Probability density distributions of the distance between the carboxylate group of substrate (R)-**10a** and residue I130K obtained from MD simulations of CvFAP M4. The distance was measured between the carbon atom of the substrate carboxylate group and the zeta nitrogen (N_ζ) of the side chain of lysine residue (I130K).* ***D)*** *Distance vs. time plot describing the interaction between the carboxylate group of (R)-**10a** – I130K obtained from MD simulations (6 replicas of 500 ns for each) of CvFAP M4. All distances are in Å.*

*4.2.5.3 Stereoselectivity of (R,X)-**10c** in CvFAP M4.*

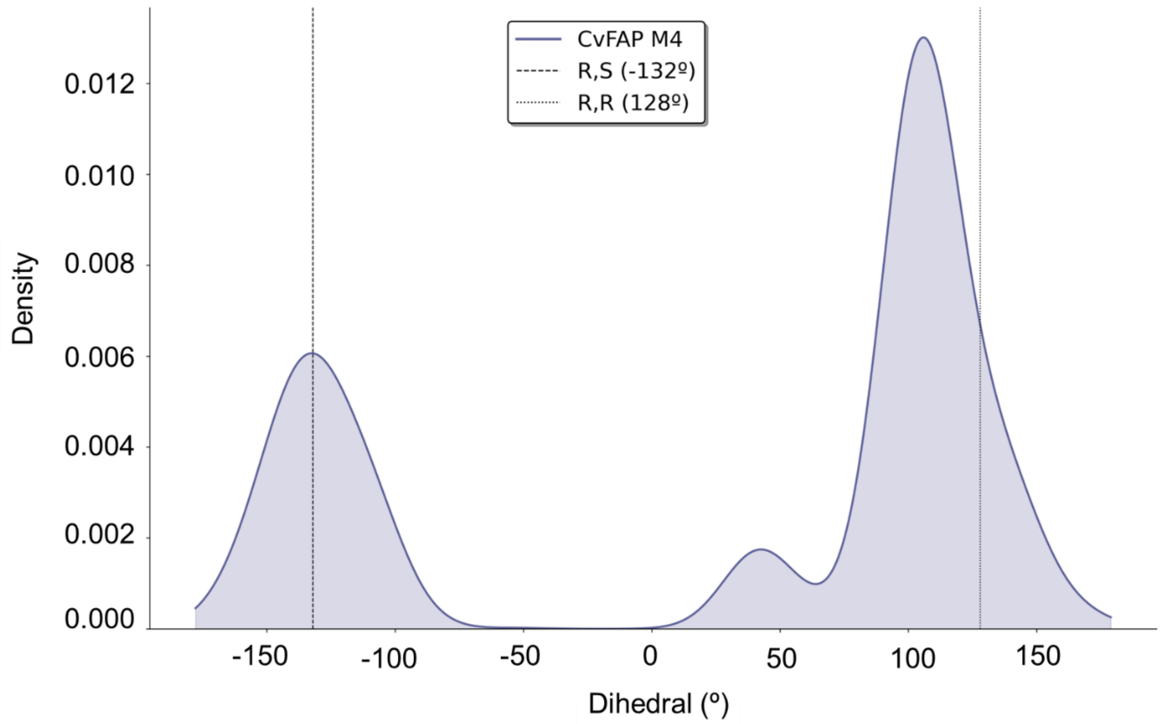

Figure S26. *Probability density distributions of the reactive dihedral angle defining C-C bond formation for cyclization of substrate (R)-**10a**, obtained from MD simulations of CvFAP M4. Only conformations in which the distance between the reacting carbon atoms (C2 and C6, for atom numbering see **Figure S12**) is below 3.5 Å were considered. The optimal TS dihedral angles for formation of the (1S,3R)-**10c** and (1R,1R)-**10c** diastereomers, computed at the DFT level, are shown as dashed lines.*

These results describe that in variant M4, the introduction of I130K mutation alters the substrate binding mode by anchoring the carboxylate group in a mid-region of the active site, resulting in slightly increased average substrate-FAD distances compared to M2 while still maintaining values compatible with efficient photoactivation. As shown below, this shift disfavours the native PCET pathway while preserving substrate folding and geometric preorganization for intramolecular C-C bond formation, driving the reaction toward the cyclization pathway. In addition, the M4 variant exhibits a distribution shifted toward larger values of the distance between Arg451 and (*R*)-**10a**, reflecting a reduced persistence of close Arg451-substrate interactions. The introduction of I130K mutation contributes to this effect by establishing competing salt-bridge interactions with substrate (*R*)-**10a**, partially unbinding the substrate from Arg451 and, consequently, disfavouring the native PCET pathway. Overall, these results support a model in which the I130K mutation introduced in the M4 variant weakens Arg451-mediated stabilization of PCET-competent conformations while promoting substrate geometries favourable for radical cyclization. The dihedral angle distribution shows an increased bias toward geometries leading to formation of the (1*R*,3*R*)-**10c** product in comparison to WT, M1 and M2 variants.

4.3 Rationalization of competing native and cyclization pathways from combined MD and DFT mechanistic analyses.

Based on the MD simulations and the DFT mechanistic studies, TD-DFT calculations were performed to elucidate why *Cv*FAP M4 enhances the cyclization pathway while disfavoring the native reaction pathway. The MD simulations indicate that the substrate (*R*)-**10a** is positioned closer to the FAD cofactor in *Cv*FAP M2, whereas in *Cv*FAP M4 the substrate is displaced from the FAD due to the additional interaction with the I130K mutation.

Representative structures extracted from MD simulations of *Cv*FAP M2 and M4, in which the substrate is either close to the FAD cofactor (obtained from *Cv*FAP M2 MD simulations) or more distant from it (extracted from *Cv*FAP M4 MD simulations), were selected to perform additional TD-DFT analyses. Active site models containing the flavin ring core, the arginine side chain and the substrate (*R*)-**10a** were generated from the selected snapshots, and the substrate (*R*)-**10a** was manually changed for radical intermediate generated after decarboxylation (**Int1**, see **Figure S27**). TD-DFT calculations describe that in both cases, the dominant electronic excitation describing the relevant electron transfer between FAD cofactor and the substrate involves orbitals 123B and 124B (**Figure S27**). The analysis of the excitation energies (**Table S2**) reveals a significant difference between the two variants: when the radical intermediate (**Int1**) is placed closer to the cofactor (*Cv*FAP M2), the excitation energy associated with the required ET from the flavin to the radical intermediate **Int1** is 3.44 eV, whereas the displacement of the **Int1** further from the FAD (*Cv*FAP M4) leads to higher ET excitation energy of 4.95 eV. These results suggest that the I130K mutation promotes interaction with the substrate that increases the substrate/Int1 - FAD distance, thereby raising the energetic cost of the photoinduced electron-transfer step associated with the initial photo-induced electron transfer and the native PCET pathway, respectively. As a consequence, the PCET leading to the native pathway is disfavored in *Cv*FAP M4 while the longer-lived radical intermediate formed upon decarboxylation becomes more likely to undergo intramolecular cyclization (see also discussions on substrate *preorganization in* **Figure S10***), consistent with the experimentally observed shift toward formation of the* cyclized product.

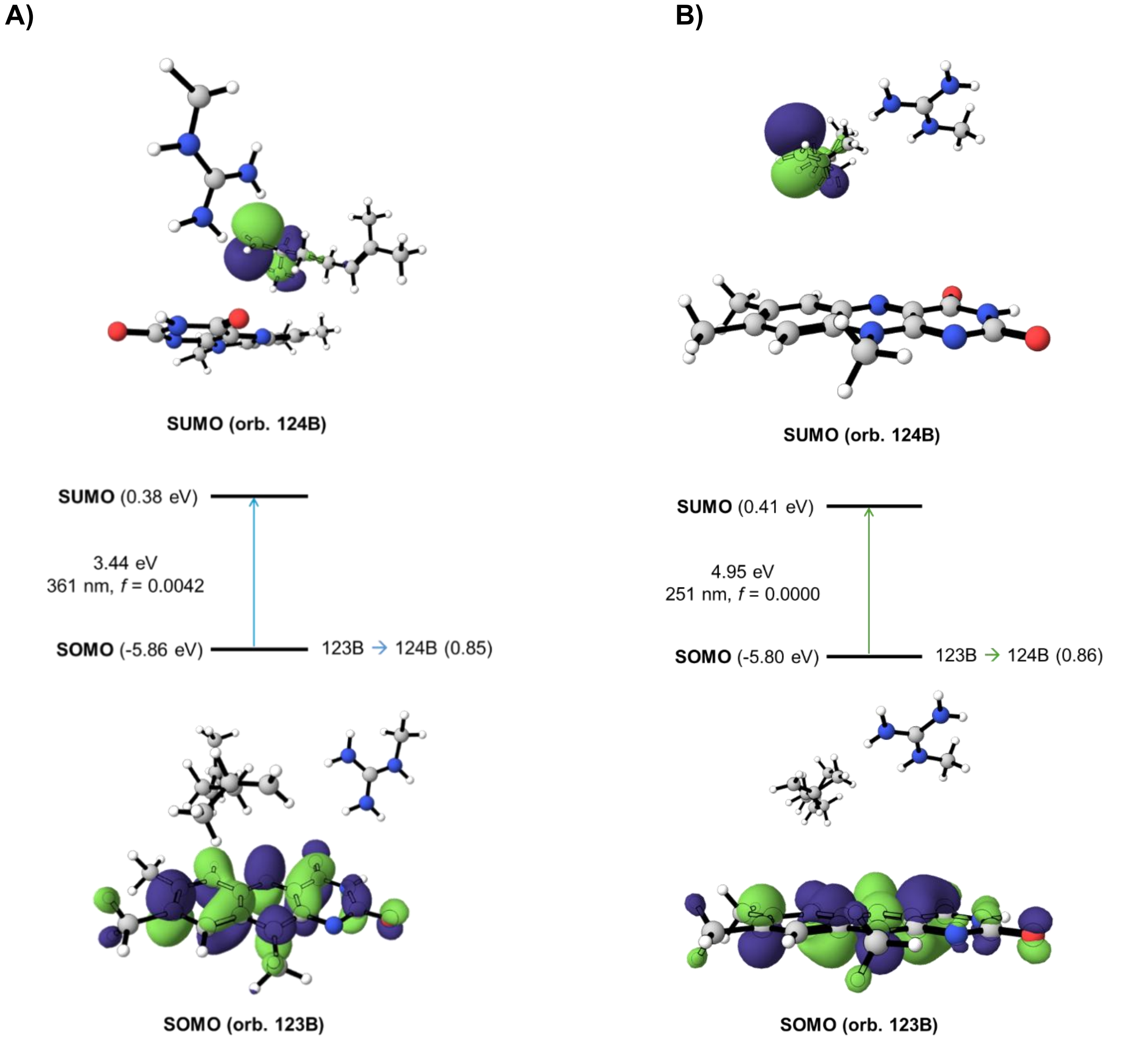

Figure S27. *Molecular orbitals and electronic transitions associated with the* $FAD^{\cdot-}$ *- radical intermediate* ***Int1*** *electron transfer studied for representative MD-derived geometries (see* ***Figure S26****) including* ***A)*** *CvFAP M2 and* ***B)*** *CvFAP M4. This excitation corresponds to a transition from orbital 123B (SOMO) to orbital 124B (SUMO), with the associated excitation energies indicated in eV.*

Table S2. *Summary of TD-DFT excited state properties for representative active site models for the FAD$^{\cdot -}$ - radical intermediate* ***Int1*** *MD-derived geometries of CvFAP M2 and CvFAP M4, including excited state, excitation energy in eV, associated wavelength in nm, oscillator strength (f), spin contamination ($\langle S^2 \rangle$) and dominant orbital transitions with corresponding contributions.*

**System**	**State**	**E (eV)**	**λ (nm)**	***f***	**S^2**	**Transitions**
*Cv*FAP M2	5	3.44	360.83	0.0042	0.460	119A → 124A (0.37)<br>121A → 124A (-0.12)<br>123B → 124B (0.85)<br>123B → 126B (-0.14)
*Cv*FAP M4	15	4.95	250.72	0.0000	0.380	123B → 124B (0.86)<br>123B → 128B (0.17)<br>123B → 130B (-0.14)<br>123B → 131B (-0.24)<br>123B → 132B (-0.11)<br>123B → 133B (0.15)<br>123B → 134B (0.14)<br>123B → 137B (0.13)<br>123B → 146B (0.11)

4.4 Role of C432 in the (*Z*)→(*E*) photoisomerization of isolated C=C bonds.

4.4.1 Mechanistic role of C432 in thiyl-radical catalyzed isomerization mechanism.

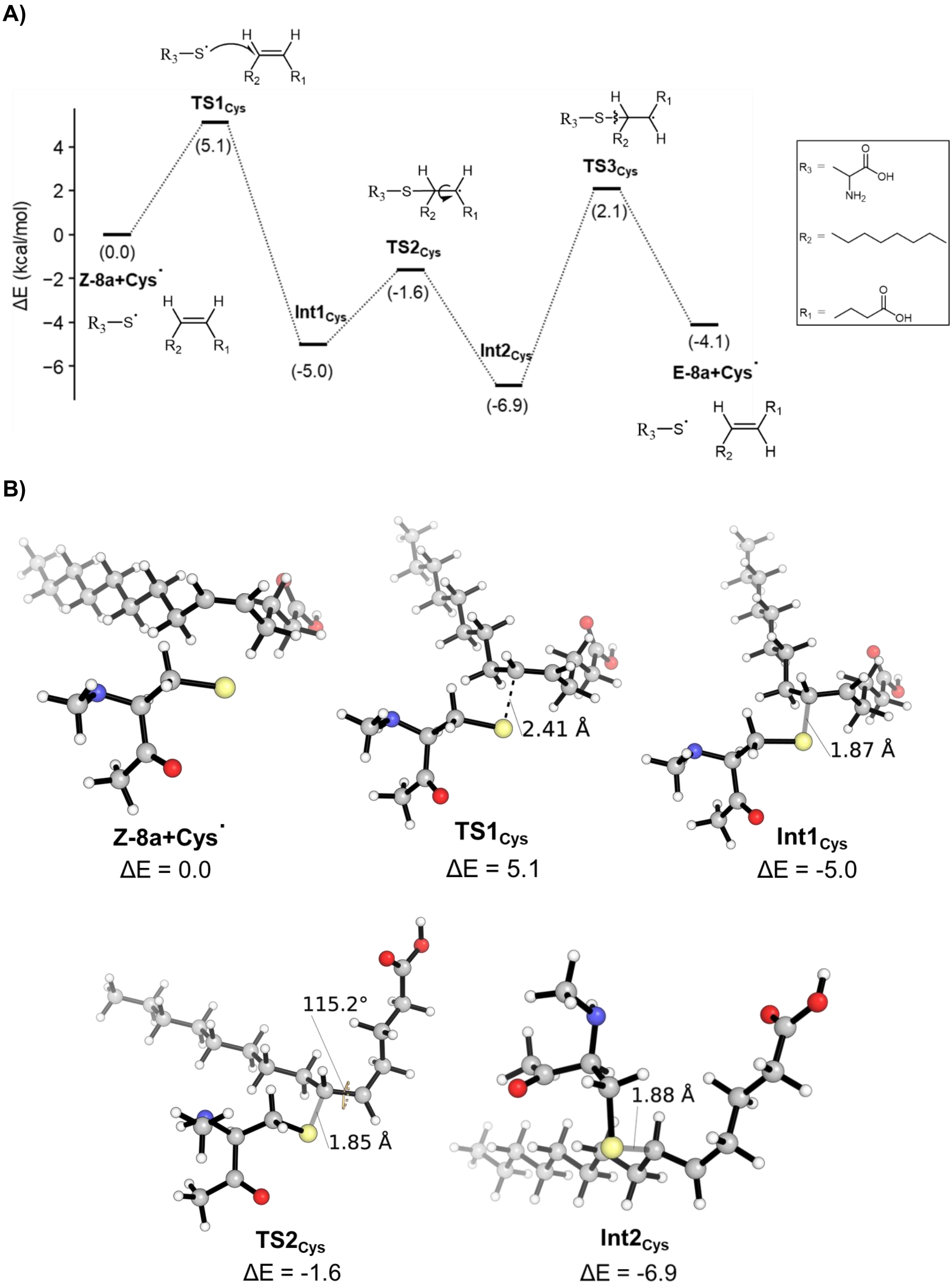

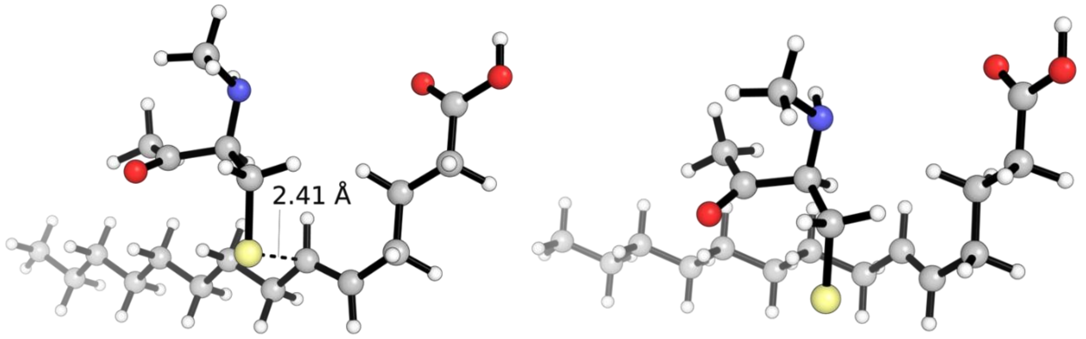

***Figure S28. A)** Reaction energy profile for the thiyl-radical-mediated (Z)→(E) isomerization of **8a** obtained from DFT calculations. Relative electronic energies (ΔE, kcal·mol⁻¹) are reported with respect to the **(Z)-8a+Cys˙** reactant complex. **B)** Optimized geometries of all reactants, transition states, intermediates, and products involved in the mechanism. All energy values were given in kcal/mol. Distances and angles in Angstrom (Å) and degrees (°), respectively.*

Based on the experimental observation that mutation of the highly conserved Cys432 residue abolishes the isomerization activity, DFT calculations performed on truncated models were used to evaluate thiyl-radical-mediated (*Z*)→(*E*) isomerization mechanism using **(Z)-8a+Cys˙** as a model system. The computed reaction energy profile indicates that the mechanism is energetically accessible. The initial thiyl-radical addition to the alkene proceeds *via* a low-energy transition state (**TS1$_{Cys}$**, $\Delta E^{\ddagger}$ = 5.1 kcal·mol^{-1}), forming a radical intermediate (**Int1$_{Cys}$**). This intermediate readily undergoes C–C bond rotation through a second transition state (**TS2$_{Cys}$**), enabling isomerization around the former double bond and yielding a slightly more stable radical intermediate (**Int2$_{Cys}$**). Subsequent thiyl-radical elimination via **TS3$_{Cys}$** leads to formation of the thermodynamically favored **(E)-8a+Cys˙** product.

4.4.2 Alternative mechanisms studied.

To support the conclusion that the isomerization proceeds via a thiyl-radical mechanism, we investigated two alternative pathways with DFT calculations. The first alternative mechanism explored (**Figure S29, A**) involves a hydrogen atom transfer (HAT) from S-H(Cys) to the double bond, generating a sulfur-centered radical on the Cys side-chain and a carbon-centered radical on the substrate, allowing for the rotation around the C-C single bond. The second alternative (**Figure S29, B**) is an anionic pathway, in which a deprotonated Cys performs a nucleophilic attack on the alkene, leading to an isomerization process that proceeds via a carbanion intermediate.

Regarding the HAT mechanism **A)**, this process is highly endergonic as illustrated by the potential energy scan in **Figure S29, C)**, indicating that this pathway is energetically inaccessible. A similar outcome was observed for anionic mechanism **B)** (**Figure S29, D**), which also proved to be highly endergonic. Consequently, based on these findings and the energy profile obtained for the thiyl-radical

isomerization mechanism, we conclude that the reaction proceeds *via* the thiyl-radical that can be generated under the photocatalytic conditions.

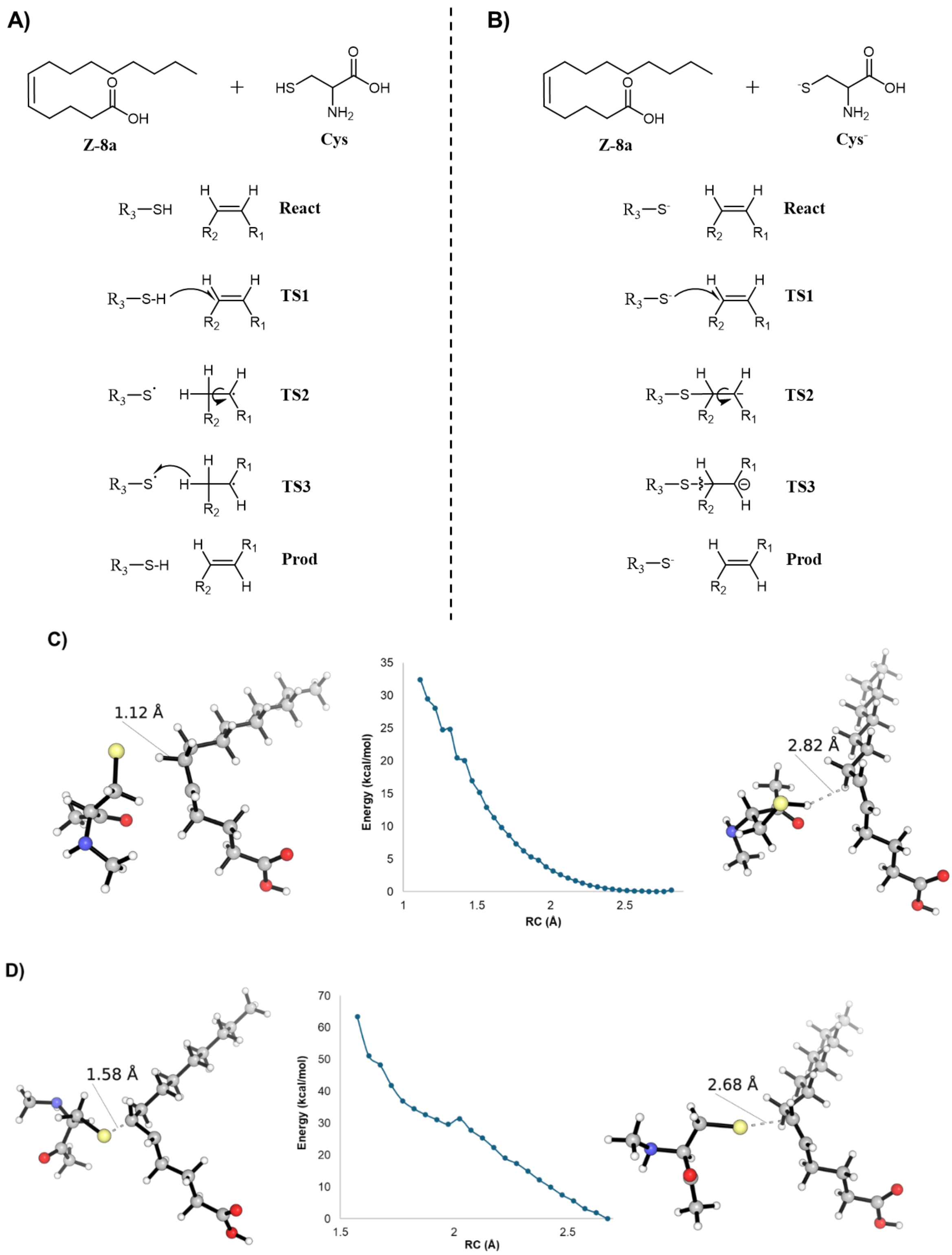

Figure S29. *Exploration of alternative mechanisms for the (Z)→(E) isomerization of* ***8a****.* ***A)*** *Homolytic S-H cleavage followed by hydrogen-atom transfer (HAT) mechanism.* ***B)*** *Anionic mechanism involving nucleophilic attack of a deprotonated cysteine.* ***C)*** *Potential energy scan of the S-H···C=C coordinate for the HAT mechanism, with two representative snapshots of the initial and the final structure.* ***D)*** *Potential energy scan of the S···C=C coordinate for the anionic mechanism, with two representative snapshots of the initial and the final structure. All distances are in Å.*

4.5 Radical Stability and Transition-State Barriers to Cyclization Efficiency.

DFT calculations rationalize the experimental reactivity trends for the decarboxylative radical cyclization onto nucleophilic C=C bonds, correlating cyclization efficiency with both the stability of the corresponding radical intermediates and the height of the cyclization transition-state barrier.

As shown in **Table S3**, substrates with high cyclization barriers ($\Delta G^{‡}$ = 15 kcal/mol) and poorly stabilized cyclized radicals ($\Delta\Delta H$ = 0–10 kcal/mol, *e.g.*, **7a** and (*Z*)-**8a**), showed no detectable cyclization, yielding only linear products. In these cases, the primary radicals are too short-lived to overcome the significant energy penalty for ring closure. Substrates **5a** and (*Z*)-**9a**, characterized by moderate cyclization barriers ($\Delta G^{‡}$ = 8–10 kcal/mol) and limited radical stabilization, afforded only trace amounts of cyclic products, indicating that the cyclization pathway begins to compete with the linear pathway. In contrast, the favorable outcome observed for substrate **10a** is explained by the formation of a highly stabilized tertiary radical upon *exo*-attack consistent with the higher experimental conversion to the cyclic product.

These findings support our working hypothesis: radical stability and lifetime directly correlate with cyclization activity. A sufficiently long-lived (stabilized) radical provides a wider kinetic window, allowing the cyclization process to occur before competing pathways take place. Overall, this establishes a clear link between radical stabilization, transition-state energetics, and the observed product distribution.

Table S3. *Substrates tested in CvFAP M1, their radical intermediates, cyclic product ratio, Gibbs energies of cyclizations (ΔG‡) and radical stabilization energies (ΔΔH), in kcal/mol.*

	Substrate	Radical Intermediate	Product ratio[a]	ΔG‡	ΔΔH[c]
**5a**	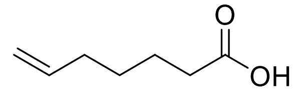	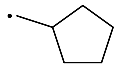	*traces*	9.8	-3.5
**6a**	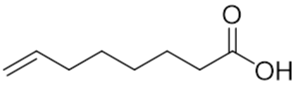	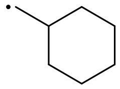	*nd*	7.3	-9.8
**7a**	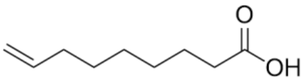	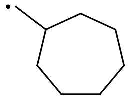	*nd*	15.0	0.0
(*Z*)-**8a**	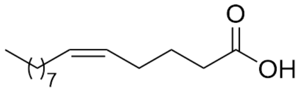	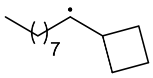	*nd*	17.1	12.0
(*Z*)-**9a**	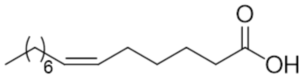	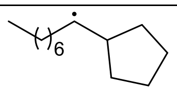	7%	8.8	-4.6
(*R*)-**10a**	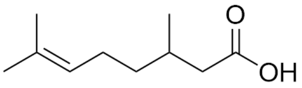	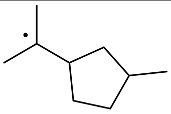	17%	8.0[b]	-6.3

nd = no product detected. [a] *Calculated based on the GC-FID area of formed product analytes: (cyclic product / (cyclic product + linear product)) *100.* [b] *(1S,3R)-**10c** product.* [c] *$\Delta\Delta H$ = FAD + R^- → $FAD^{\cdot-}$ + $R^\cdot$*

5 Supplementary Experimental Data

5.1 Quick Quality Control of the SSM libraries

5.1.1 Y466X

Table S4. *Distribution of nucleotide bases at the target codon Y466 after the 1st round of SSM.*

	**Base distribution of target codon Y466**		
	**1st**	**2nd**	**3rd**
Theoretical values	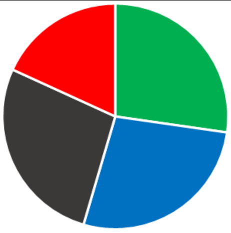	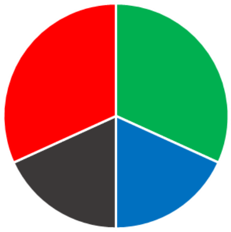	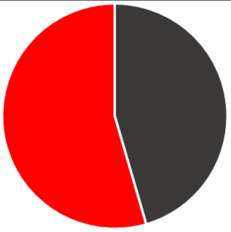
T_a= 62.7 °C	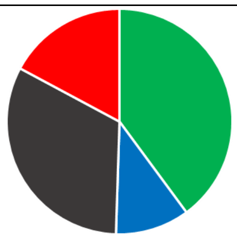	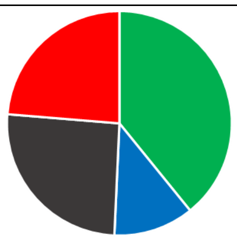	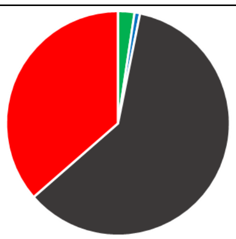
**T_a=65.8 °C**	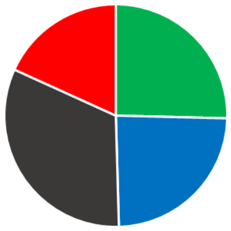	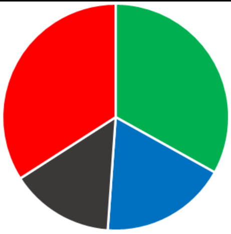	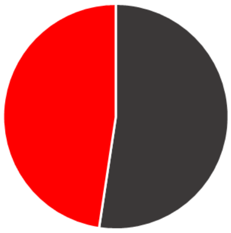
T_a=68 °C	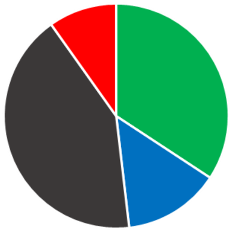	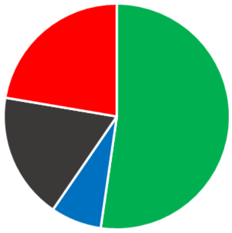	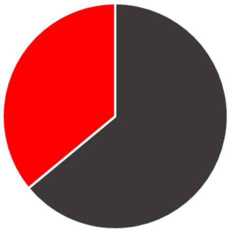
T_a=72 °C	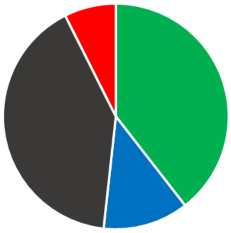	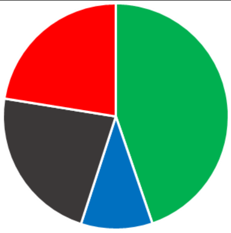	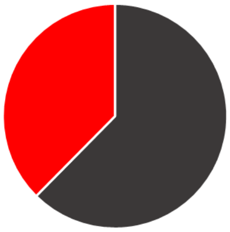

The percentage distribution from a pooled sample of mutants is presented as pie diagrams for QQC. The color code for nucleotide bases: Guanine (G, black), Adenine (A, green), Thymine (T, red) and Cytosine (C, blue). T_a = Annealing temperature of PCR reactions. PCR products from the annealing temperature in bold were chosen as library.

5.1.2 Y466A/L386X

Table S5. *Distribution of nucleotide bases at the target codon L386 after the 2nd round of SSM.*

	Base Distribution at target codon L386		
	**1st**	**2nd**	**3rd**
Theoretical values	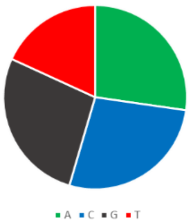	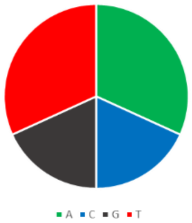	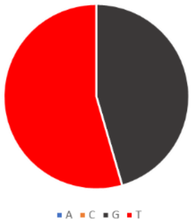
T_a = 68.8 °C	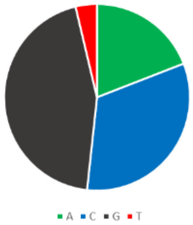	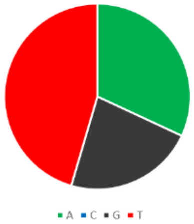	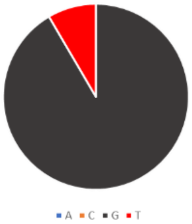
**T_a = 70.0 °C**	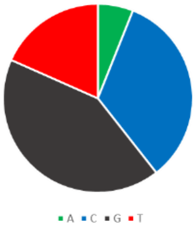	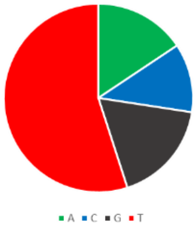	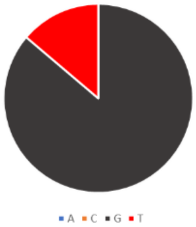
T_a = 71.0 °C	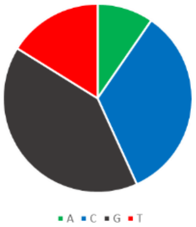	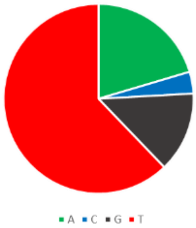	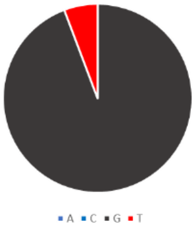

The percentage distribution from a pooled sample of mutants is presented as pie diagrams for QQC. The color code for nucleotide bases: Guanine (G, black), Adenine (A, green), Thymine (T, red) and Cytosine (C, blue). T_a = Annealing temperature of PCR reactions. PCR products from the annealing temperature in bold were chosen as library.

5.1.3 Y466A/I398X

Table S6. *Distribution of nucleotide bases at the target codon I398 after the 2nd round of SSM.*

	Base Distribution at target codon I398		
	**1st**	**2nd**	**3rd**
Theoretical values	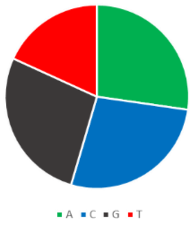	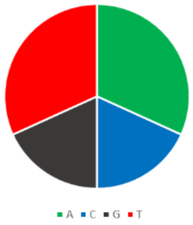	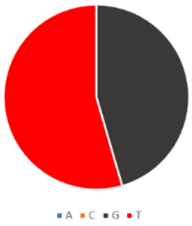
T_a = 61.0 °C	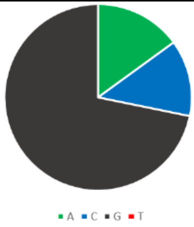	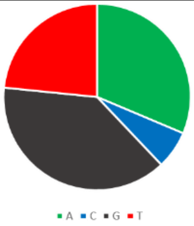	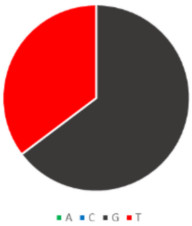
T_a = 61.9 °C	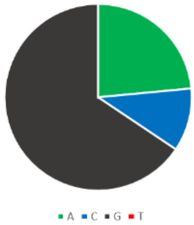	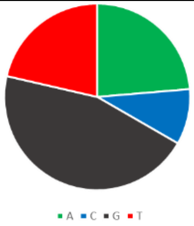	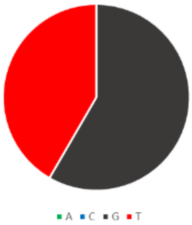
T_a = 64.1 °C	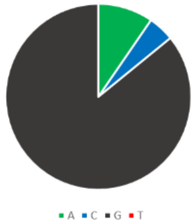	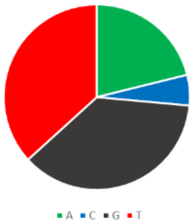	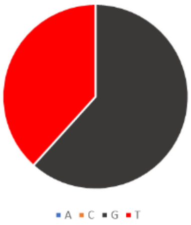
**T_a = 65.4 °C**	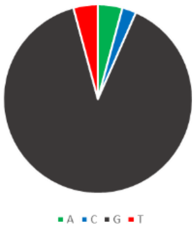	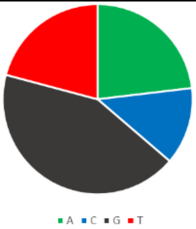	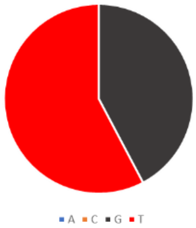

The percentage distribution from a pooled sample of mutants is presented as pie diagrams for QQC. The color code for nucleotide bases: Guanine (G, black), Adenine (A, green), Thymine (T, red) and Cytosine (C, blue). T_a = Annealing temperature of PCR reactions. PCR products from the annealing temperature in bold were chosen as library.

5.1.4 Y466A/G431X

Table S7. *Distribution of nucleotide bases at the target codon G431 after the 2nd round of SSM.*

	**Base Distribution at target codon G431**		
	**1st**	**2nd**	**3rd**
Theoretical values	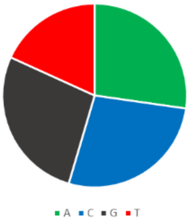	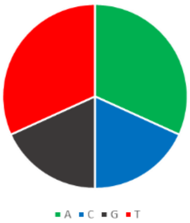	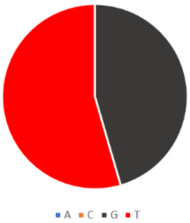
T_a = 61.0 °C	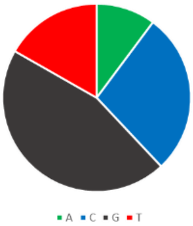	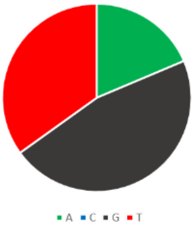	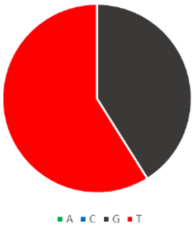
**T_a = 62.8 °C**	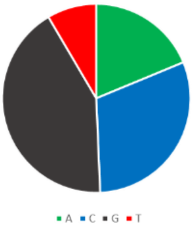	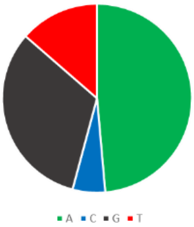	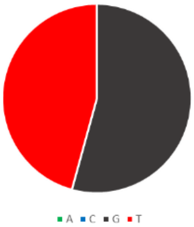
T_a = 65.4 °C	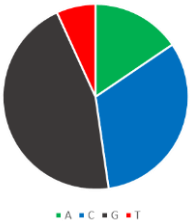	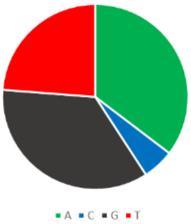	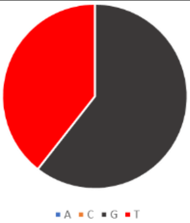

The percentage distribution from a pooled sample of mutants is presented as pie diagrams for QQC. The color code for nucleotide bases: Guanine (G, black), Adenine (A, green), Thymine (T, red) and Cytosine (C, blue). T_a = Annealing temperature of PCR reactions. PCR products from the annealing temperature in bold were chosen as library.

5.1.5 Y466A/V453X

Table S8. *Distribution of nucleotide bases at the target codon V453 after the 2nd round of SSM.*

	Base Distribution at target codon V453		
	**1st**	**2nd**	**3rd**
Theoretical values	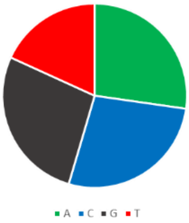	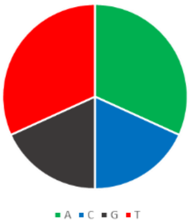	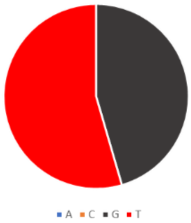
**T_a = 61.0 °C**	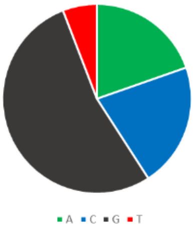	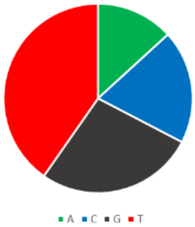	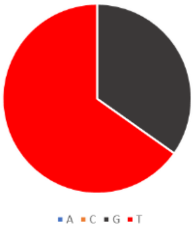
T_a = 62.8 °C	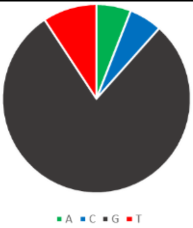	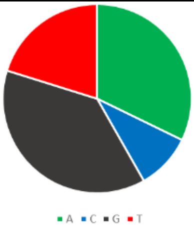	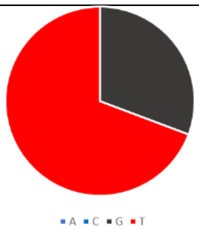
T_a = 65.4 °C	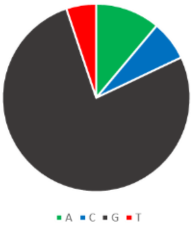	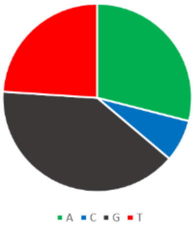	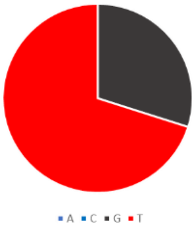
T_a = 67.9°C	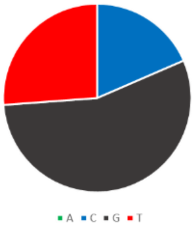	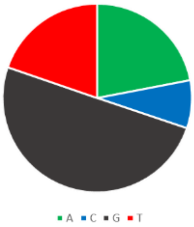	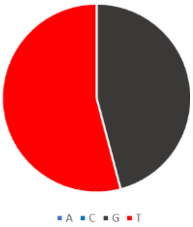

The percentage distribution from a pooled sample of mutants is presented as pie diagrams for QQC. The color code for nucleotide bases: Guanine (G, black), Adenine (A, green), Thymine (T, red) and Cytosine (C, blue). T_a = Annealing temperature of PCR reactions. PCR products from the annealing temperature in bold were chosen as library.

5.1.6 Y466A/G462X

Table S9. *Distribution of nucleotide bases at the target codon G462 after the 2nd round of SSM.*

	**Base Distribution at target codon G462**		
	**1st**	**2nd**	**3rd**
Theoretical values (VHG library)	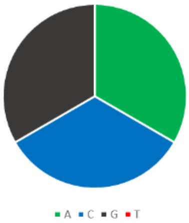	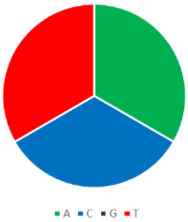	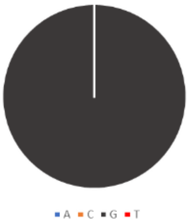
**T_a = 61.0 °C**	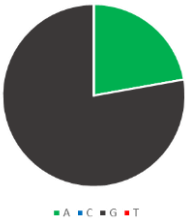	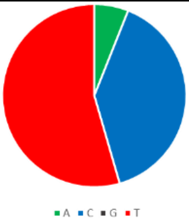	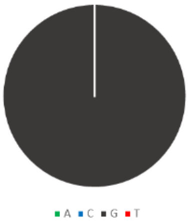

The percentage distribution from a pooled sample of mutants is presented as pie diagrams for QQC. The color code for nucleotide bases: Guanine (G, black), Adenine (A, green), Thymine (T, red) and Cytosine (C, blue). T_a = Annealing temperature of PCR reactions. PCR products from the annealing temperature in bold were chosen as library.

5.1.7 Y466A/S574X

Table S10. *Distribution of nucleotide bases at the target codon S574 after the 2nd round of SSM.*

	**Base Distribution at target codon S574**		
	**1st**	**2nd**	**3rd**
Theoretical values	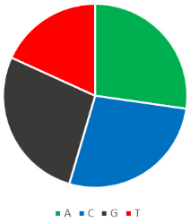	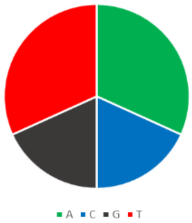	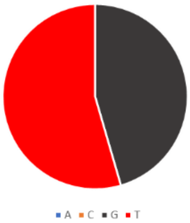
**T_a = 59.0 °C**	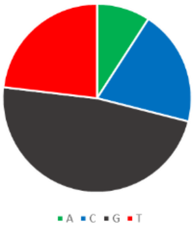	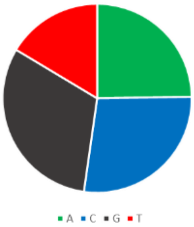	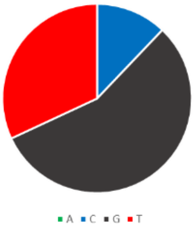

The percentage distribution from a pooled sample of mutants is presented as pie diagrams for QQC. The color code for nucleotide bases: Guanine (G, black), Adenine (A, green), Thymine (T, red) and Cytosine (C, blue). T_a = Annealing temperature of PCR reactions. PCR products from the annealing temperature in bold were chosen as library.

5.1.8 Y466A/V453S/F134X

Table S11. *Distribution of nucleotide bases at the target codon F134 after the 3rd round of SSM.*

	**Base Distribution at target codon F134**		
	**1st**	**2nd**	**3rd**
Theoretical values	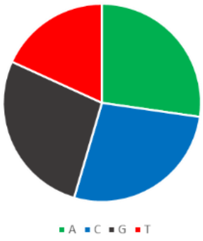	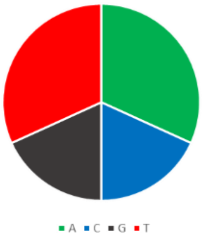	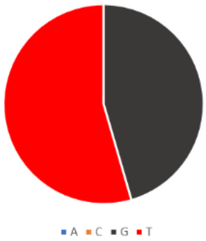
T_a = 64.9 °C	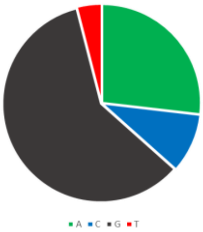	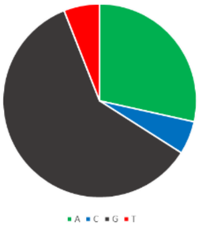	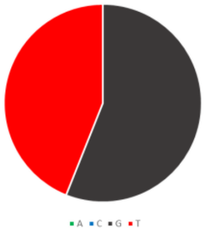
**T_a = 67.2 °C**	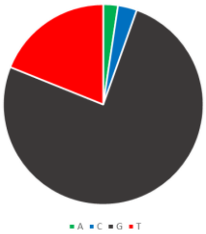	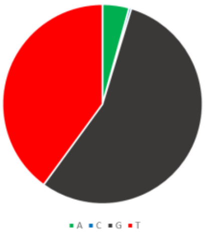	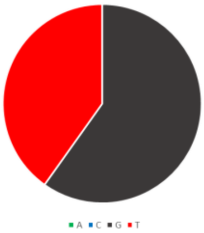
**T_a = 68.4 °C**	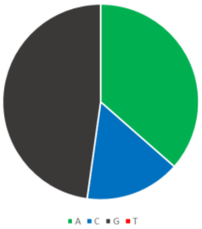	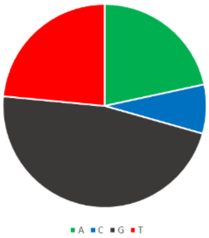	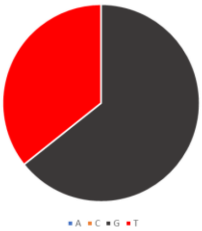
T_a = 70.0 °C	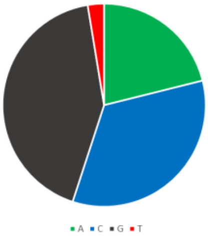	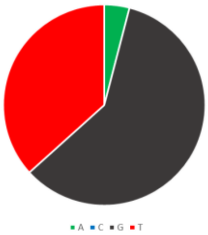	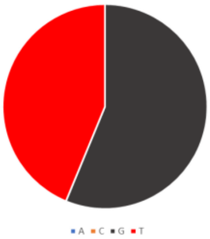

The percentage distribution from a pooled sample of mutants is presented as pie diagrams for QQC. The color code for nucleotide bases: Guanine (G, black), Adenine (A, green), Thymine (T, red) and Cytosine (C, blue). T_a = Annealing temperature of PCR reactions. ***PCR*** *products from the annealing temperature in bold were chosen as library in a 1:1 mixture.*

5.1.9 Y466A/V453S/G431X

Table S12. *Distribution of nucleotide bases at the target codon G431 after the 3rd round of SSM.*

	**Base Distribution at target codon G431**		
	**1st**	**2nd**	**3rd**
Theoretical values	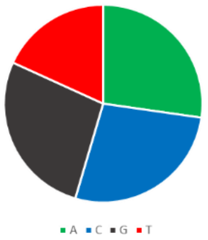	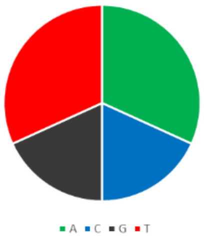	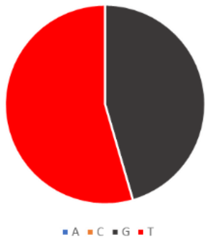
**T_a = 62.8 °C**	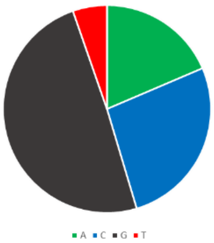	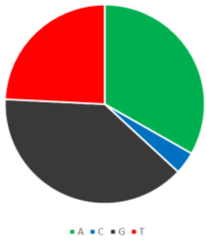	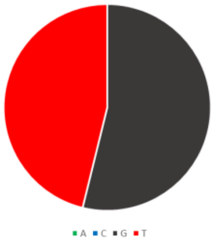

The percentage distribution from a pooled sample of mutants is presented as pie diagrams for QQC. The color code for nucleotide bases: Guanine (G, black), Adenine (A, green), Thymine (T, red) and Cytosine (C, blue). T_a = Annealing temperature of PCR reactions. PCR products from the annealing temperature in bold were chosen as library.

5.1.10 Y466A/V453S/A384X

Table S13. *Distribution of nucleotide bases at the target codon A384 after the 3rd round of SSM.*

	Base Distribution at target codon A384		
	**1st**	**2nd**	**3rd**
Theoretical values	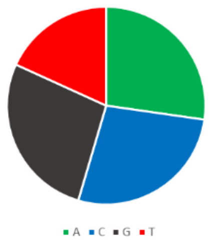	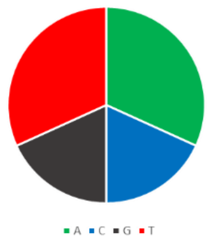	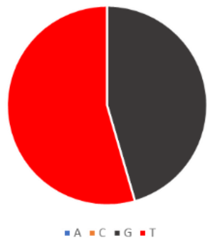
**T_a = 64.6 °C**	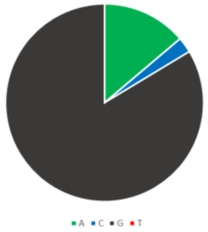	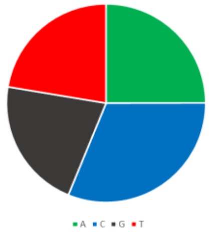	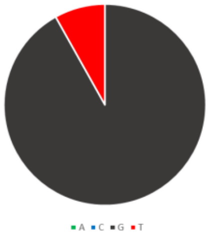
T_a = 66.9 °C	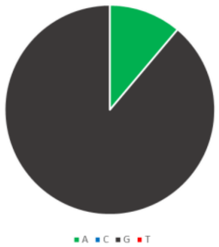	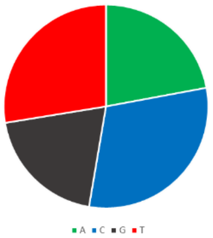	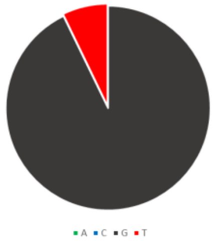
T_a = 68.1 °C	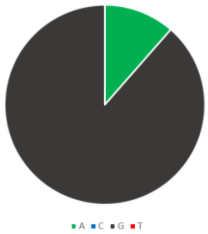	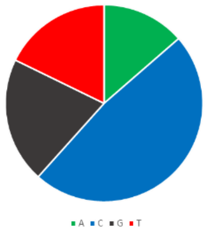	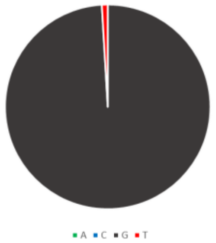
T_a = 70.4 °C	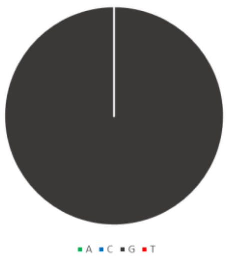	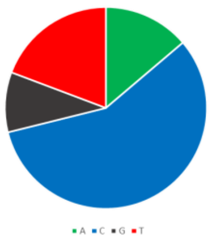	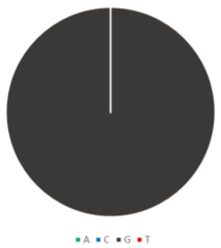

The percentage distribution from a pooled sample of mutants is presented as pie diagrams for QQC. The color code for nucleotide bases: Guanine (G, black), Adenine (A, green), Thymine (T, red) and Cytosine (C, blue). T_a = Annealing temperature of PCR reactions. PCR products from the annealing temperature in bold were chosen as library.

5.1.11 Y466A/V453S/L386X

Table S14. *Distribution of nucleotide bases at the target codon L386 after the 3rd round of SSM.*

	**Base Distribution at target codon L386**		
	**1st**	**2nd**	**3rd**
Theoretical values	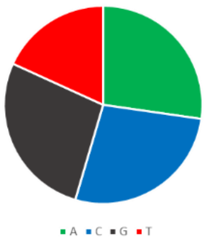	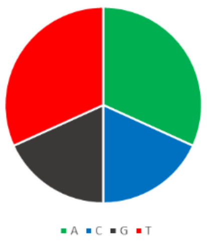	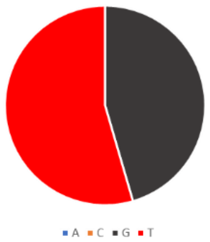
**T_a = 69.2 °C**	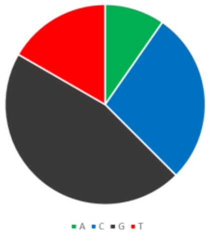	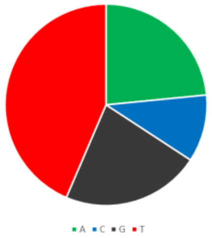	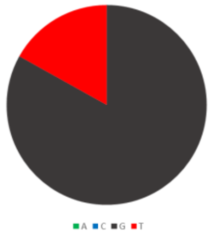
T_a = 70.4 °C	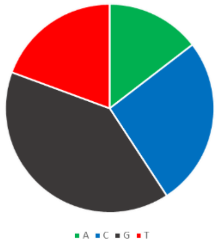	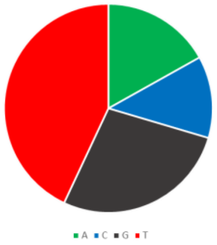	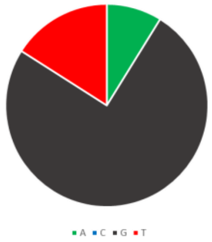

The percentage distribution from a pooled sample of mutants is presented as pie diagrams for QQC. The color code for nucleotide bases: Guanine (G, black), Adenine (A, green), Thymine (T, red) and Cytosine (C, blue). T_a = Annealing temperature of PCR reactions. PCR products from the annealing temperature in bold were chosen as library.

5.1.12 Y466A/V453S/G431S/C432X

Table S15. *Distribution of nucleotide bases at the target codon C432 after the 4th round of SSM using variant Y466A/V453S/G431S as template.*

	**Base Distribution at target codon C432**		
	**1st**	**2nd**	**3rd**
Theoretical values	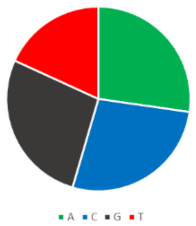	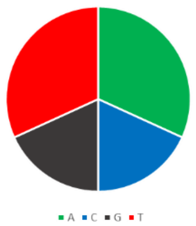	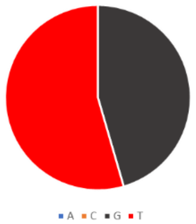
**T_a = 60.0 °C**	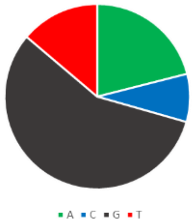	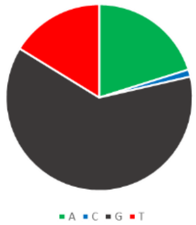	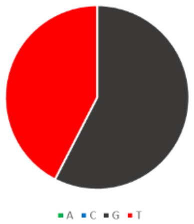
T_a = 61.0 °C	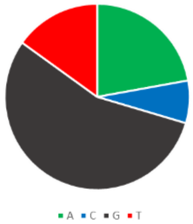	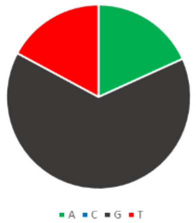	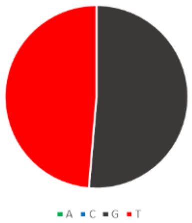
T_a = 62.2 °C	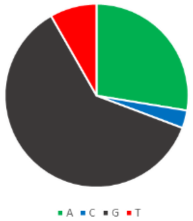	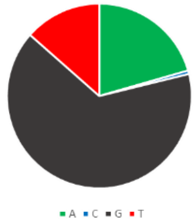	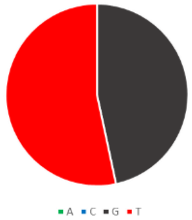
T_a = 65.3 °C	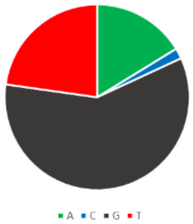	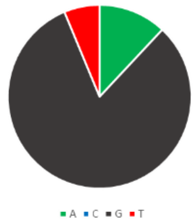	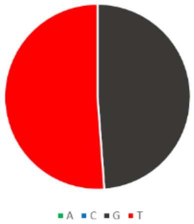

The percentage distribution from a pooled sample of mutants is presented as pie diagrams for QQC. The color code for nucleotide bases: Guanine (G, black), Adenine (A, green), Thymine (T, red) and Cytosine (C, blue). T_a = Annealing temperature of PCR reactions. PCR products from the annealing temperature in bold were chosen as library.

5.1.13 Y466A/V453S/G431S/I130X

Table S16. *Distribution of nucleotide bases at the target codon I130 after the 4th round of SSM using variant Y466A/V453S/G431S as template.*

	**Base Distribution at target codon I130**		
	**1st**	**2nd**	**3rd**
Theoretical values	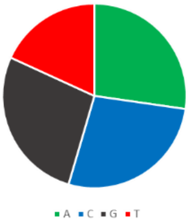	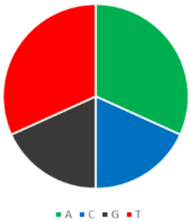	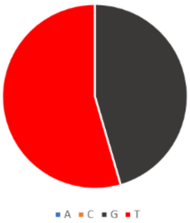
**T_a = 65.3 °C**	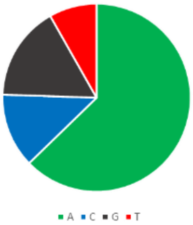	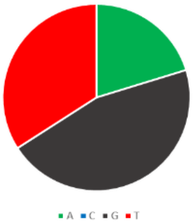	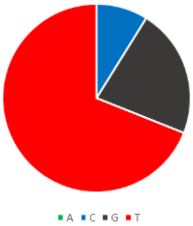
T_a = 66.7 °C	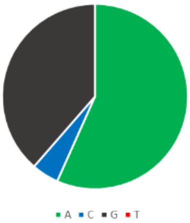	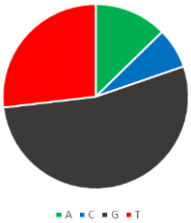	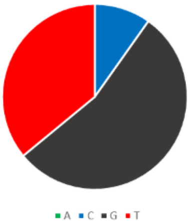
T_a = 68.3 °C	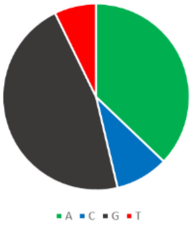	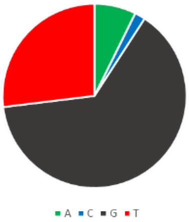	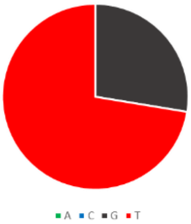

The percentage distribution from a pooled sample of mutants is presented as pie diagrams for QQC. The color code for nucleotide bases: Guanine (G, black), Adenine (A, green), Thymine (T, red) and Cytosine (C, blue). T_a = Annealing temperature of PCR reactions. PCR products from the annealing temperature in bold were chosen as library.

5.2 Control experiments

Table S17. *Control experiments for the intermolecular decarboxylative radical coupling with electrophilic C=C bonds.*

Conditions	Fatty acid	Enone	1a [mM]	1b [mM]	4d [mM]
***Cv*FAP Wt[a]**			9.1 ± 0.5	1.2 ± 0.1	nd
**Dark Control[b]**			9.5 ± 0.0	nd	nd
**FAD Control[c]**	**1a**	**3c**	10.6 ± 0.7	nd	nd
**Substrate Control[d]**			11.2 ± 0.6	nd	nd
**Empty *E. coli* Control[e]**			10.0 ± 0.4	nd	nd
**Y466A[a]**	**1a**	x	7.8 ± 0.2	1.2 ± 0.1	nd

*[a]Reaction conditions: CvFAP Wt or variant Y466A (lyophilized CFE, 20 mg/mL, corresponding to 3.4 nmol of CvFAP Y466A), fatty acid (**1a**, 10 mM), enone (**3c**, 20 mM), 30% v/v DMSO (degassed), in Tris·HCl buffer (100 mM, pH 8.5, degassed) with a final volume of 1 mL, anaerobic (nitrogen atmosphere); illumination in a custom photoreactor (blue LEDs, 455 nm, 36 µE/L), 16 h at 25 °C and 500 rpm. [b]dark control: in the absence of light. [c]FAD control: FAD (100 µM), with substrate and without enzyme under irradiation. [d]substrate control: with substrate and without enzyme under irradiation. [e]empty E. coli control: E. coli BL21 (DE3) (20 mg/mL, lyophilized CFE). nd = not detected; Wt = wild type. Triplicates.*

Table S18. *Control experiments for the decarboxylative radical cyclization with nucleophilic C=C bonds.*

Conditions	Carboxylic acid	(R)-**10a [mM]**	10b [mM]	10c [mM]
***Cv*FAP Wt[a]**		3.0 ± 0.1	4.8 ± 0.4	< 0.1
**Dark Control[b]**		9.0 ± 0.2	nd	nd
**FAD Control[c]**	(*R*)-**10a**	9.9 ± 0.1	nd	nd
**Substrate Control[d]**		11.1 ± 0.1	nd	nd
**Empty *E. coli* Control[e]**		10.2 ± 0.3	nd	nd

*[a]Reaction conditions: CvFAP Wt or variant Y466A (lyophilized CFE, 20 mg/mL, corresponding to 3.5 nmol of CvFAP Y466A), carboxylic acid ((R)-**10a**, 10 mM), 30% v/v DMSO, in Tris·HCl buffer (100 mM, pH 8.5) with a final volume of 1 mL, aerobic; illumination in a custom photoreactor (blue LEDs, 455 nm, 36 µE/L), 16 h at 25 °C and 500 rpm. [b]dark control: in the absence of light. [c]FAD control: FAD (100 µM), with substrate and without enzyme under irradiation. [d]substrate control: with substrate and without enzyme under irradiation. [e]empty E. coli control: E. coli BL21 (DE3) (20 mg/mL, lyophilized CFE). nd = not detected. Triplicates.*

Table S19. *Reaction and controls of the carbohydroxylation activity using CvFAP V453A and (E/Z)-**12a.***

Condition	12d [mM]
**anaerobic**	0.05 ± 0.04
**aerobic**	0.24 ± 0.01
***Cv*FAP Wt[a]**	0.05 ± 0.04
**Dark Controls[b]**	nd
**Substrate Control[c]**	nd
**Empty *E. coli* Control[d]**	nd
**FAD Control[e]**	traces
**$NADP^+$ Control[f]**	nd
**NADPH Control[g]**	nd

*Reaction conditions: CvFAP V453A (lyophilized CFE, 20 mg/mL, corresponding to 5.5 nmol of CvFAP V453A), substrate (**12a**: 10 mM), 30% v/v DMSO, in Tris·HCl buffer (100 mM, pH 8.5) with a final volume of 1.0 mL; illumination in a custom photoreactor (blue LEDs, 455 nm, 36 µE/L), 7 h at 25 °C and 500 rpm; aerobic: air atmosphere; anaerobic: under argon atmosphere (Schlenk line) using degassed DMSO and Tris·HCl buffer. Wt= wild type. [a]CvFAP Wt (lyophilized, 20 mg/mL), duplicates. [b]dark control: in the absence of light, duplicates. [c]substrate control: with substrate and without enzyme under irradiation. [d]empty E. coli control: E. coli BL21 (DE3) (20 mg/mL, lyophilized CFE). [e]FAD control: FAD (100 µM), with substrate and without enzyme under irradiation. [f]$NADP^+$ control: $NADP^+$ (100 µM), with substrate and without enzyme under irradiation. [g]NADPH control: NADPH (100 µM), with substrate and without enzyme under irradiation. nd = not detected. Triplicates, except stated otherwise.*

5.3 Characterization of hit variants at 1 mL scale

5.3.1 1st Round of ISM:

Table S20. *Quantified results of the biotransformation of (R)-**10a** towards intramolecular C-C bond formation using lyophilized CFE of the hit variants of CvFAP identified in the first round of evolution.*

Variant	(*R*)-10a [mM]	10b [mM]	10c [mM]	Recovery [mM]	*d.e.* (*R,R*)/(*S,R*)	%cyclised species[a]
***Cv*FAP Wt**	4.0 ± 0.2	4.2 ± 0.1	0.2 ± 0.0	8.3	16	3.5
**I398L**	4.9 ± 0.3	3.5 ± 0.2	0.1 ± 0.0	8.5	16	4.0
**V453A**	2.4 ± 0.4	5.1 ± 0.3	0.2 ± 0.0	7.7	12	4.4
**Y466V**	6.6 ± 0.2	2.0 ± 0.1	0.3 ± 0.0	8.9	43	11.8
**Y466S**	6.8 ± 0.2	1.6 ± 0.0	0.3 ± 0.0	8.6	44	14.1
**Y466A**	2.8 ± 0.1	4.9 ± 0.1	0.9 ± 0.0	8.7	15	16.1

*Reaction conditions: CvFAP Wt or variants (lyophilized CFE, 10 mg/mL), carboxylic acid ((R)-**10a**, 10 mM), 30% v/v DMSO, in Tris·HCl buffer (100 mM, pH 8.5) with a final volume of 1 mL, aerobic; illumination in a custom photoreactor (blue LEDs, 455 nm, dutyrange: 100, dutycycle: 1% corresponding to 36 µE/L), 4 h at 25 °C and 500 rpm; triplicates. [a]calculated based on the quantified values of **10b** and **10c**.*

Table S21. *Quantified results of the biotransformation of (S)-**10a** towards intramolecular C-C bond formation using lyophilized CFE of the hit variants of CvFAP identified in the first round of evolution.*

Variant	(*S*)-10a [mM]	10b [mM]	10c [mM]	Recovery [mM]	*d.e.* (*S,S*)/(*R,S*)	%cyclised species[a]
***Cv*FAP Wt**	5.0 ± 0.3	2.3 ± 0.1	0.2 ± 0.0	7.5	64	6.6
**I398L**	5.9 ± 0.1	2.2 ± 0.1	0.2 ± 0.0	8.3	61	6.8
**V453A**	5.7 ± 0.2	1.8 ± 0.1	0.2 ± 0.0	7.8	47	10.8
**Y466V**	7.2 ± 0.1	1.0 ± 0.0	0.2 ± 0.0	8.4	5	15.8
**Y466S**	7.2 ± 0.1	0.6 ± 0.1	0.2 ± 0.0	8.0	5	21.4
**Y466A**	4.8 ± 0.3	2.7 ± 0.2	0.6 ± 0.0	8.1	10	18.8

*Reaction conditions: CvFAP Wt or variants (lyophilized CFE, 10 mg/mL), carboxylic acid ((S)-**10a**, 10 mM), 30% v/v DMSO, in Tris·HCl buffer (100 mM, pH 8.5) with a final volume of 1 mL, aerobic; illumination in a custom photoreactor (blue LEDs, 455 nm, dutyrange: 100, dutycycle: 1% corresponding to 36 µE/L), 4 h at 25 °C and 500 rpm; triplicates. [a]calculated based on the quantified values of **10b** and **10c**.*

5.3.2 2nd Round of ISM

Table S22. *Quantified results of the biotransformation of (R)-**10a** towards intramolecular C-C bond formation using lyophilized CFE of the hit variants of CvFAP identified in the second round of evolution.*

Variant	(*R*)-10a [mM]	10b [mM]	10c [mM]	Recovery [mM]	*d.e.* (*R,R*)/(*S,R*) [%]	%cyclised species
**Y466A/G462A**	2.6 ± 0.1	4.4 ± 0.1	1.0 ± 0.1	8.1	-21	19
**Y466A/I398C**	4.0 ± 0.4	3.1 ± 0.2	0.7 ± 0.1	7.7	10	18
**Y466A/I398V**	4.6 ± 0.1	2.8 ± 0.1	0.6 ± 0.1	8.0	11	18
**Y466A/G431V**	9.0 ± 0.2	traces	traces	9.1	n.a.	n.a
**Y466A/G431N**	8.9 ± 0.0	nd	nd	9.0	n.a.	n.a
**Y466A/V453S**	6.9 ± 0.2	0.8 ± 0.1	0.4 ± 0.0	8.1	12	36
**Y466A/V453G**	8.1 ± 0.5	0.2 ± 0.1	0.1 ± 0.0	8.5	10	42
**Y466A/V453A**	7.1 ± 0.1	0.9 ± 0.0	0.5 ± 0.0	8.5	10	34

*Reaction conditions: CvFAP variants (lyophilized CFE, 10 mg/mL), carboxylic acid ((R)-**10a**, 10 mM), 30% v/v DMSO, in Tris·HCl buffer (100 mM, pH 8.5) with a final volume of 1 mL, aerobic; illumination in a custom photoreactor (blue LEDs, 455 nm, dutyrange: 100, dutycycle: 1% corresponding to 36 µE/L), 4 h at 25 °C and 500 rpm; triplicates. [a]calculated based on the quantified values of **10b** and **10c**. nd=not detected; n.a.=not analysed.*

Table S23. *Quantified results of the biotransformation of (S)-**10a** towards intramolecular C-C bond formation using lyophilized CFE of the hit variants of CvFAP identified in the second round of evolution.*

Variant	(*S*)-10a [mM]	10b [mM]	10c [mM]	Recovery [mM]	*d.e.* (*S,S*)/(*R,S*) [%]	%cyclised species[a]
**Y466A/G462A**	4.0 ± 0.0	2.0 ± 0.0	0.6 ± 0.0	6.7	6.1	22
**Y466A/I398C**	4.4 ± 0.1	1.8 ± 0.0	0.4 ± 0.0	6.7	8.6	19
**Y466A/I398V**	4.8 ± 0.2	1.4 ± 0.2	0.3 ± 0.0	6.6	11.8	19
**Y466A/G431V**	7.0 ± 0.1	traces	traces	7.0	n.a.	n.a.
**Y466A/G431N**	7.2 ± 0.1	nd	nd	7.2	n.a.	n.a.
**Y466A/V453S**	6.9 ± 0.1	0.1 ± 0.0	0.1 ± 0.0	7.1	12.0	38
**Y466A/V453G**	6.6 ± 0.7	0.0 ± 0.0	0.0 ± 0.0	6.7	11.5	33
**Y466A/V453A**	6.2 ± 0.2	0.4 ± 0.0	0.2 ± 0.0	6.8	10.7	38

*Reaction conditions: CvFAP variants (lyophilized CFE, 10 mg/mL), carboxylic acid ((S)-**10a**, 10 mM), 30% v/v DMSO, in Tris·HCl buffer (100 mM, pH 8.5) with a final volume of 1 mL, aerobic; illumination in a custom photoreactor (blue LEDs, 455 nm, dutyrange: 100, dutycycle: 1% corresponding to 36 µE/L), 4 h at 25 °C and 500 rpm; triplicates. [a]calculated based on the quantified values of **10b** and **10c**. nd=not detected; n.a.=not analysed.*

5.3.3 3rd Round of ISM

Table S24. *Quantified results of the biotransformation of (R)-**10a** towards intramolecular C-C bond formation using lyophilized CFE of the hit variants of CvFAP identified in the third round of evolution.*

Variant	(*R*)-10a [mM]	10b [mM]	10c [mM]	Recovery [mM]	*d.e.* (*R,R*)/(*S,R*)	%cyclised species[a]
**Y466A/V453S/G431W**	6.2 ± 0.5	0.4 ± 0.0	0.1 ± 0.0	6.7	40	17
**Y466A/V453S/G431D[b]**	5.2	0.6	0.4	6.1	17	38
**Y466A/V453S/G431S**	6.6 ± 0.2	0.4 ± 0.1	0.3 ± 0.1	7.3	12	43
**Y466A/V453S/A384W**	7.9 ± 0.1	nd	nd	7.9	n.a.	n.a.

*Reaction conditions: CvFAP variants (lyophilized CFE, 10 mg/mL), carboxylic acid ((R)-**10a**, 10 mM), 30% v/v DMSO, in Tris·HCl buffer (100 mM, pH 8.5) with a final volume of 1 mL, aerobic; illumination in a custom photoreactor (blue LEDs, 455 nm, dutyrange: 100, dutycycle: 1% corresponding to 36 µE/L), 4 h at 25 °C and 500 rpm; triplicates, except stated otherwise. [a]calculated based on the quantified values of **10b** and **10c**. nd=not detected; n.a.=not analysed. [b]duplicates.*

Table S25. *Quantified results of the biotransformation of (S)-**10a** towards intramolecular C-C bond formation using lyophilized CFE of the hit variants of CvFAP identified in the third round of evolution.*

Variant	(*S*)-10a [mM]	10b [mM]	10c [mM]	Recovery [mM]	*d.e.* (*S,S*)/(*R,S*)	%cyclised species[a]
**Y466A/V453S/G431W**	6.5 ± 0.1	0.2 ± 0.0	traces	6.8	45	n.a.
**Y466A/V453S/G431D**	4.8 ± 0.5	0.2 + 0.0	0.1 ± 0.0	5.0	14	31
**Y466A/V453S/G431S**	4.9 ± 0.9	0.1 ± 0.0	traces	5.0	11	n.a.
**Y466A/V453S/A384W**	6.0 ± 0.6	0.1 ± 0.0	nd	6.0	n.a.	n.a.

*Reaction conditions: CvFAP variants (lyophilized CFE, 10 mg/mL), carboxylic acid ((S)-**10a**, 10 mM), 30% v/v DMSO, in Tris·HCl buffer (100 mM, pH 8.5) with a final volume of 1 mL, aerobic; illumination in a custom photoreactor (blue LEDs, 455 nm, dutyrange: 100, dutycycle: 1% corresponding to 36 µE/L), 4 h at 25 °C and 500 rpm; triplicates, except stated otherwise. [a]calculated based on the quantified values of **10b** and **10c**. nd=not detected; n.a.=not analysed.*

5.3.4 4th Round of ISM

***Table S26.** Quantified results of the biotransformation of (R)-**10a** towards intramolecular C-C bond formation using lyophilized CFE of the hit variants of the directed evolution.*

Variant	c. (*R*)-10a [mM]	c. 10b [mM]	c. 10c [mM]	%cyclised species[b]
**Y466A/V453S/G431S/I130K**	9.3 ± 0.3	< 0.1	0.3 ± 0.0	82[b]
**Y466A/V453S/G431S/I130K[a]**	6.9 ± 0.3	< 0.1	0.5 ± 0.0	81[b]

*Reaction conditions: CvFAP variants (lyophilized CFE, 10 mg/mL, except stated otherwise), carboxylic acid ((R)-**10a**, 10 mM), 30% v/v DMSO, in Tris·HCl buffer (100 mM, pH 8.5) with a final volume of 1 mL, aerobic; illumination in a custom photoreactor (blue LEDs, 455 nm, dutyrange: 100, dutycycle: 1% corresponding to 36 µE/L), 4 h at 25 °C and 500 rpm; triplicates. [a]CFE: 30 mg/mL. [b]calculated based on the GC-FID areas of formed product analytes: (**10c**/(**10c**+**10b**)) *100.*

5.4 Determination of enzyme concentration and TONs

5.4.1 BCA assay of hit variants (CFE) and purified *Cv*FAP wt

To determine the TONs of respective *Cv*FAP hit variants, equal amounts of total protein needed to be loaded into each well in the SDS PAGE. For this reason, a BCA micro-scale assay in 96 well MTPs was carried out as described in the user's manual (Thermofisher, TB380). First the respective lyophilized CFEs and the purified *Cv*FAP Wt stock were thawed to room temperature. Then CFE (20 mg) was rehydrated in Tris·HCl buffer (1 mL; 100 mM, pH 8.5) for 15 min. During half time of the rehydration samples were gently flicked several times to facilitate homogenization. CFEs and the purified *Cv*FAP Wt stock were diluted 1:20 and 1:100, respectively, using Tris·HCl buffer (100 mM, pH 8.5). In parallel BSA standards were prepared in a range from 0-1000 µg/mL as stated in the user manual's standard assay. The BCA working reagent was prepared by adding 50 parts BCA solution to 1 part of 4% cupric acid (aqueous solution) and used immediately.

BSA standards (25 µL) were pipetted into individual wells of a 96-well plate. The CFE samples (1:20 dilution) and the purified *Cv*FAP Wt stock (1:100) were measured in triplicates. Next the BCA working reagent (200 µL) was added *via* a multi-channel micropipette. The plate was covered with parafilm and incubated at 37 °C for 30 min for color development. The plate was then cooled to ambient temperature for 5 min, after which immediately the absorbance was measured at 562 nm at 25 °C after 5 s initial shaking on a UV/VIS plate reader (SpectraMax2, Molecular Devices).

The total protein concentration of different batches of *Cv*FAP Y466A, comprising Y466A-1 until Y466A-4 alongside Y466A-2* were measured. Within this work batch Y466A-4 was used for decarboxylative radical cyclization with nucleophilic C=C bonds, batch Y466A-1 was used for intermolecular decarboxylative radical coupling with electrophilic C=C bonds, batch Y466A-2 was used for (*Z*)→(*E*) photoisomerization of isolated C=C bonds, while batch Y466A-2* was used for the characterization of the first *Cv*FAP hit variant identified in the directed evolution campaign at 1 mL scale. The batch Y466A-3 was not used at all.

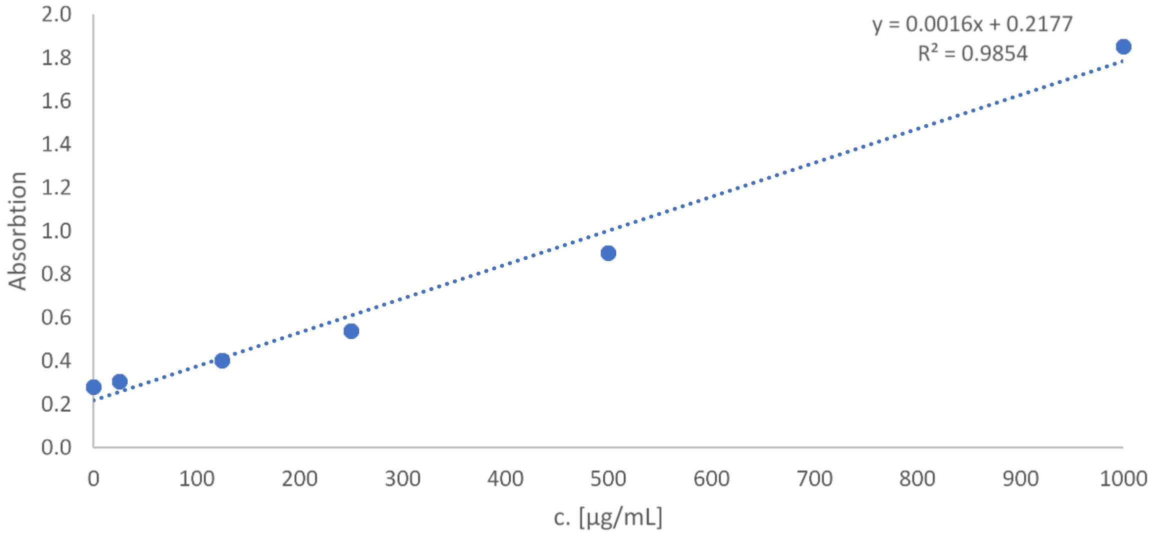

Figure S30. *BSA calibration curve for the BCA assay.*

Table S27. *Measured total protein concentrations of the lyophilized CFE CvFAP samples (20 mg/ml) and the purified CvFAP Wt Stock.*

Variant	Total protein[c] [mg/mL]
***Cv*FAP Wt (purified)[a]**	16.08
***Cv*FAP Wt[b]**	4.49
**Y466A-4[b]**	5.81
**Y466A-1[b]**	5.43
**Y466A-2[b]**	5.22
**Y466A-3[b]**	5.14
***Cv*FAP M2[b]**	5.60
***Cv*FAP M3[b]**	5.58
***Cv*FAP M4[b]**	5.05
**Y466A/C432A[b]**	4.99
**Y466A/C432S[b]**	4.75
***Cv*FAP M3/C432A[b]**	5.67
**V453A[b]**	4.82
**Y466A/G462A[b]**	5.07
**Y466A-2*[b]**	5.63

[a]purified CvFAP Wt, dilution (1:100); [b]lyophilized CFEs, dilution (1:20); [c]recaculated for 20 mg of lyophilized CFE; Wt = wild type.

5.4.2 Densitometric analysis of SDS-PAGE

For the SDS-PAGE sample preparation of the CFEs equal amounts of protein were used (45 µg) filling up to a total volume of 30 µL with dH_2O. Standards of purified *Cv*FAP were prepared in a range of 0.1-1.6 µg/well. Samples were then mixed with Laemmli buffer (30 µL, 2x concentrated). Additionally,

*Cv*FAP M2 whole cells (40 OD_{600}/mL) were diluted 1:20, and mixed 1:1 with Laemmli buffer. The samples were heated for 5 min to 95 °C and spun down for 30 s at 14680 rpm. Protein Ladder (PL) = 5 µL, lyophilized CFEs = 5 µL, *Cv*FAP M2 whole cells = 20 µL, *Cv*FAP Wt calibration = 12 µL were loaded onto a commercially available 4-12% Bis-Tris gel using MOPS-Tris buffer and was run at 160 V for 1 h. Noteworthy, the SDS-PAGE was overrun to increase the resolution, which led to elution of two bands of the protein ladder with the lowest molecular weight.

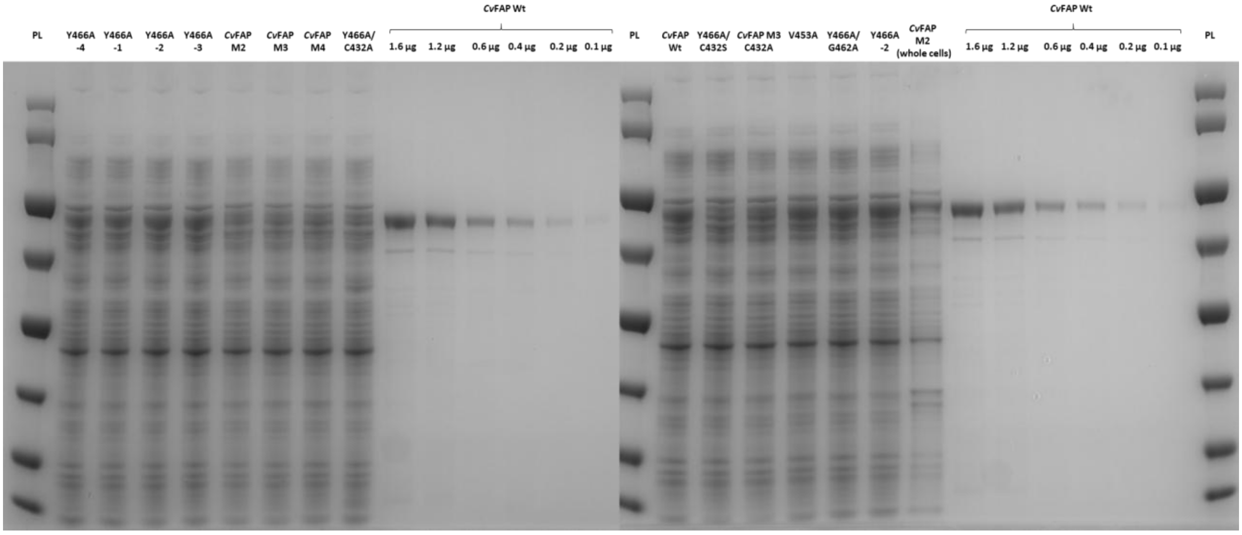

Figure S31. *SDS-PAGE for the densitometric determination of the protein concentration of CvFAP variants in the different enzyme preparations, alongside purified CvFAP Wt calibration standards. Y466A-4 (used for decarboxylative radical cyclization with nucleophilic C=C bonds); Y466A-1 (used for intermolecular decarboxylative radical coupling with electrophilic C=C bonds); Y466A-2 (used for (Z)→(E) photoisomerization of isolated C=C bonds; CvFAP M2 = Y466A/V453S; CvFAP M3 = Y466A/V453S/G431S; CvFAP M4 = Y466A/V453S/G431S/I130K; CvFAP M3/C432A = Y466A/V453S/G431S/C432A; Wt = wild type; PL = protein ladder; CvFAP Wt calibration curve (from 0.1-1.6 µg/well).*

This SDS-PAGE was analyzed with the software GeneTools from Syngene. The baseline of the individual band of each track was set by using a Rolling Disk of 40 pixels. For each PAGE a *Cv*FAP Wt calibration curve was established as illustrated in **Figure S32 and Figure S33**, respectively.

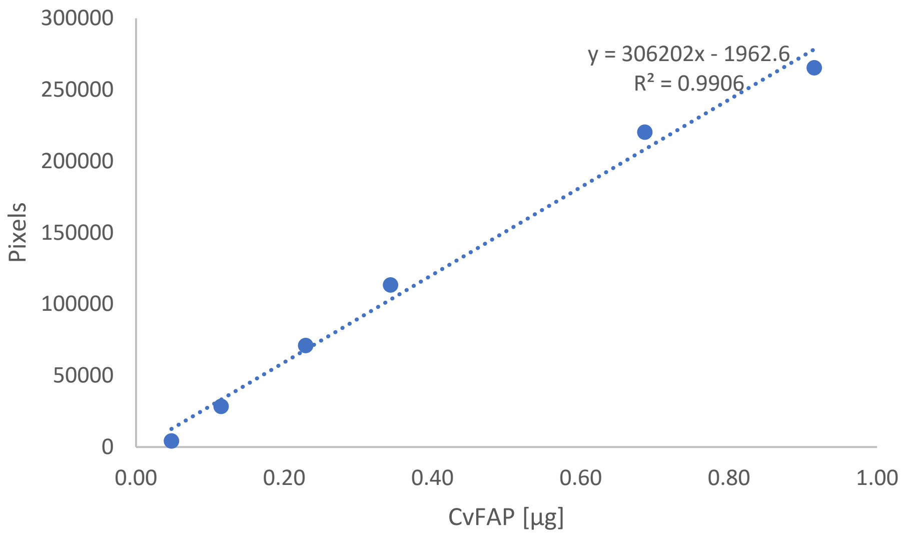

Figure S32. *CvFAP Wt calibration for the densitometric determination of the protein concentration of selected CvFAP variants of the first SDS PAGE.*

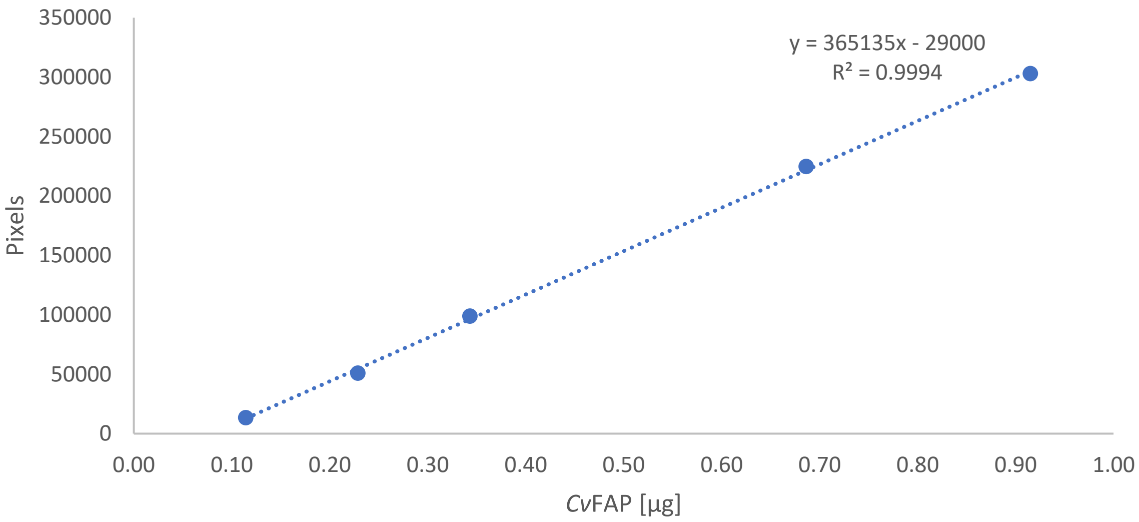

Figure S33. *CvFAP Wt calibration for the densitometric determination of the protein concentration of selected CvFAP variants on the second SDS PAGE.*

The protein concentration of each variant and enzyme preparation was determined based on the raw volume (pixels) of the *Cv*FAP band of each track and the respective *Cv*FAP Wt calibration of the corresponding gel.

Table S28. *Concentration of CvFAP Wt and variants n_{cat} in a 20 mg sample of either lyophilized CFE or lyophilized whole cells.*

Enzyme	Raw volume (Pixels)	n(catalyst) [µmol in 20 mg]
**Y466A-4**	56021	0.0037
**Y466A-1**	59340	0.0037
**Y466A-2**	76227	0.0045
**Y466A-3**	67649	0.0039
**Y466A/V453S**	12438	0.0009
**Y466A/V453S/G431S**	9064	0.0007
**Y466A/V453S/G431S/I130K**	10020	0.0007
**Y466A/C432A**	15162	0.0009
***Cv*FAP Wt**	86833	0.0048
**Y466A/C432S**	13691	0.0019
***Cv*FAP M3/C432A**	22496	0.0027
**V453A**	94192	0.0055
**Y466A/G462A**	92801	0.0057
**Y466A-2***	131027	0.0083
***Cv*FAP M2 whole cells**	133814	0.0111

Y466A-4 (used for decarboxylative radical cyclization with nucleophilic C=C bonds); Y466A-1 (used for intermolecular decarboxylative radical coupling with electrophilic C=C bonds); Y466A-2 (used for (Z)→(E) photoisomerization of isolated C=C bonds; Y466A-2 (used for the characterization at 1 mL scale in the first round of directed evolution); Wt=wild type.*

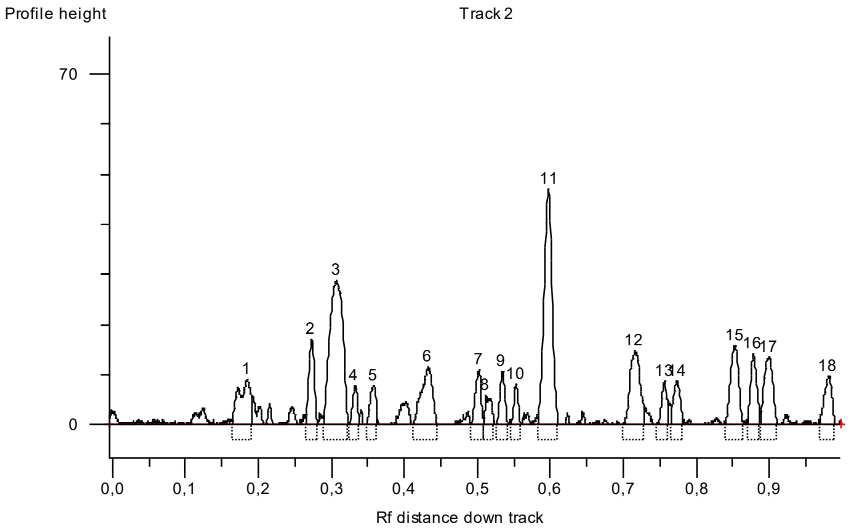

Figure S34. *Exemplary histogram of the protein bands of track 2 (Y466A-4), including the band corresponding to CvFAP and its variants (Peak 3).*

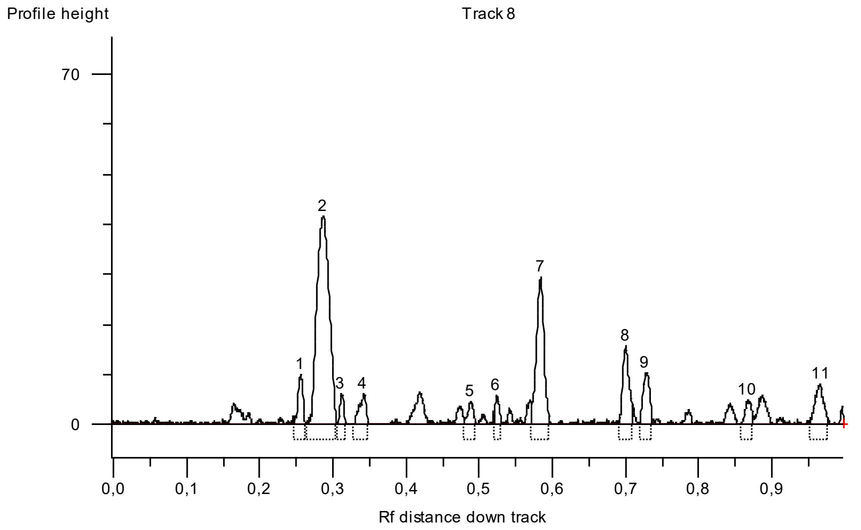

Figure S35. *Exemplary histogram of the protein bands of track 8 (CvFAP M2 Whole cells), including the band corresponding to CvFAP and its variants (Peak 2)*

6 Synthesis of Substrates and Reference Material

For all reactions under inert conditions, the reaction setup was heated, evacuated and flushed with the respective inert gas (N_2 or Ar) in three cycles.

6.1 (*Z*)-5-tetradecenoic acid (*Z*-**8a**)

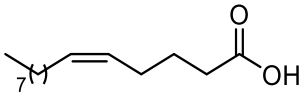

The literature procedure[31] was slightly adapted and performed under inert conditions. To a 250 mL 3-neck round-bottom flask, equipped with a stirring bar and a gas inlet, 4-(carboxybutyl)-triphenylphosphonium bromide (3.3 g, 7.5 mmol, 1.5 eq.) was added as solution in anhydrous THF (50 mL), followed by the addition of NaHMDS (1.0 M in THF, 15 mL, 15 mmol, 3 eq.) at 0 °C under argon flow. The mixture was stirred at 0 °C for 1 h and the resulting reddish-orange mixture was cooled to -95 °C to -90 °C in a N_2/acetone cooling bath. A solution of nonanal (860 µL, 5 mmol, 1 eq.) in anhydrous THF (15 mL) was added dropwise within 20 min, followed by stirring at -95 °C for 1 h. After this time, the mixture was allowed to warm up to 0 °C within 3 h. The reaction was then quenched with sat. aqueous NH_4Cl (80 mL) at 0 °C, followed by filtration through celite. After separating the organic phase, the aqueous layer was extracted with Et_2O (4 x 60 mL), and the combined organic phases were washed with dH_2O (1 x 50 mL) and brine (2 x 100 mL). The solution was dried over Na_2SO_4, filtered, and concentrated *in vacuo* to yield a pale-yellow oil as crude (1.81g). The crude product was purified *via* flash column chromatography (silica; cyclohexane/EtOAc/TFA= 80:19.5:0.5) to yield (*Z*)-**8a** as a pale-yellow oil (726 mg; 64% yield). TLC (cyclohexane/EtOAc = 4:1 + 2 drops AcOH; detection: UV and $KMnO_4$, R_f = 0.26). **^{1}H-NMR** (300 MHz, $CDCl_3$) δ 10.31 (bs, 1H), 5.76 – 4.99 (m, 2H), 2.36 (t, *J* = 7.6 Hz, 2H), 2.08 (q, *J* = 7.2 Hz, 2H), 2.00 (q, *J* = 6.6 Hz, 2H), 1.70 (p, *J* = 7.2 Hz, 2H), 1.27 (m, 12H), 0.88 (t, *J* = 7.0 Hz, 3H). **^{13}C-NMR** (75 MHz, $CDCl_3$) δ 180.14, 131.55, 128.24, 33.54, 32.05, 29.84, 29.66, 29.46, 27.40, 26.57, 24.75, 22.83, 14.26.

6.2 (*Z*)-6-tetradecenoic acid (*Z*-**9a**)

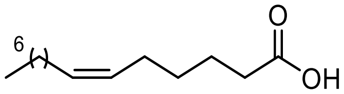

The literature procedure[31] was slightly adapted and performed under inert conditions. To a 250 mL 3-neck round-bottom flask, equipped with a stirring bar and a gas inlet 5-(carboxypentyl)-triphenylphosphonium bromide (3.43 g, 7.5 mmol, 1.5 eq.) was added as solution in anhydrous THF (50 mL), followed by the addition of NaHMDS (1.0 M in THF, 15 mL, 15 mmol, 3 eq.) at 0 °C under argon flow. The mixture was stirred at 0 °C for 1 h and the resulting reddish-orange mixture was cooled to -95 °C to -90 °C in a N_2/acetone cooling bath. A solution of octanal (782 µL, 5 mmol, 1 eq.) in anhydrous THF (15 mL) was added dropwise to the mixture within 20 min, followed by stirring at -95 °C for 1 h. After this time the mixture was allowed to warm up to 0 °C within 3 h. The reaction

was then quenched with sat. aqueous NH_4Cl (80 mL) at 0 °C, followed by filtration through celite. After separating the organic phase, the aqueous layer was extracted with Et_2O (4 x 60 mL), and the combined organic phases were washed with dH_2O (1 x 50 mL) and brine (2 x 100 mL). The solution was dried over Na_2SO_4, filtered, and concentrated *in vacuo* to yield a pale-yellow oil. The crude product was purified *via* flash column chromatography (silica; cyclohexane/EtOAc/TFA = 80:19.5:0.5, detection: $KMnO_4$ and UV) to yield (*Z*)-**9a** as a pale-yellow oil (632 mg; 56% yield). **^{1}H NMR** (300 MHz, CDCl3) δ 11.17 (bs, 1H), 5.35 (m, 2H), 2.36 (t, *J* = 7.3 Hz, 2H), 2.02 (m, 4H), 1.65 (p, *J* = 7.4 Hz, 2H), 1.34 (m, 12H), 0.88 (t, *J* = 6.3 Hz, 3H). **^{13}C-NMR** (75 MHz, $CDCl_3$) δ 179.46, 130.19, 128.56, 33.53, 31.48, 29.34, 28.89, 28.83, 28.71, 26.86, 26.39, 23.90, 22.29, 13.72.

6.3 Methyl-6-hydroxy-hexanoate (**13**)

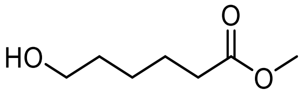

The synthesis was performed as previously reported.[32] To a dry and inert two-neck round-bottom flask (250 mL), equipped with a stirring bar, reflux condenser, and septum, was added ε-caprolactone (9.7 mL, 87.6 mmol, 1 eq.), sulfuric acid (96%, 0.53 mL, 9.86 mmol, 0.11 eq.) and anhydrous MeOH (140 mL). The mixture was stirred under reflux for 3.5 h. After this time, TLC (silica, cyclohexane/EtOAc 2:1, detection: $KMnO_4$, R_f (product) = 0.18), confirmed complete conversion of the starting material. The mixture was allowed to cool to ambient temperature and worked up by adding solid sodium bicarbonate (2.03 g, 24.13 mmol) and stirring for 10 min. Solids were removed by filtration and the filtrate was concentrated *in vacuo* to remove MeOH. The remaining solid residue was then dissolved in EtOAc (150 mL) and washed with dH_2O (100 mL). The organic phase was separated, and the aqueous phase was extracted with EtOAc (3 x 75 mL). The combined organic phases were washed with dH_2O (2 x 100 mL), dried over anhydrous Na_2SO_4, and concentrated to dryness under reduced pressure furnishing compound **13** as colorless oil (10.92 g, 85% yield), which was stored at -21 °C. Compound **13** was used without further purification. The NMR signals obtained agrees with literature.[33] **^{1}H NMR** (300 MHz, $CDCl_3$) δ 3.69 – 3.58 (m, 4H), 2.33 (t, *J* = 7.4 Hz, 2H), 1.87 – 1.50 (m, 5H), 1.48 – 1.29 (m, 2H). **^{13}C NMR** (75 MHz, $CDCl_3$) δ 174.34, 62.73, 51.66, 34.09, 32.38, 25.39, 24.73.

6.4 Methyl-6-oxo-hexanoate (**14**)

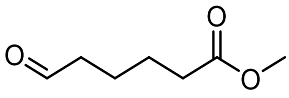

The synthesis was performed as previously reported.[32] A solution of anhydrous oxalylchloride (2 M in DCM; 22.6 mL, 45.15 mmol, 1.3 eq.) in dry DCM (190 mL) was added to a dry and inert three-neck round bottom flask (500 mL), equipped with a stirring bar, gas inlet and septum. Next, anhydrous DMSO (6.51 mL, 2.7 mmol, 2.7 eq.) was added dropwise over 30 min at -78 °C. The resulting mixture was stirred for 20 min until gas evolution ceased, after which a solution

of **13** (5.0 g, 34.2 mmol, 1.0 eq.) in DCM (32 mL) was added dropwise over 20 min at -78 °C. The mixture was stirred for 20 min, followed by the dropwise addition of anhydrous Et_3N (19.6 mL, 140.9 mmol, 4.1 eq.) over 15 min. After stirring for 10 min, the reaction mixture was cooled on an ice bath and stirred at 0 °C until complete conversion of the starting material was observed on TLC (silica, cyclohexane/EtOAc 1:2, detection: $KMnO_4$, R_f (product) = 0.76). After 1 h, the reaction mixture was poured into *d*H_2O (210 mL) and vigorously stirred for 5 min. The product was isolated *via* extraction of the aqueous phase with DCM (4 x 140 mL). The combined organic phases were washed with *d*H_2O (2 x 280 mL) and brine (2 x 280 mL), filtered over celite, dried over anhydrous Na_2SO_4 and concentrated *in vacuo* to yield a yellow-orange oily crude product (4.94 g; 100.3% crude yield). The crude was purified *via* flash column chromatography (silica; cyclohexane/EtOAc 1:2; detection: $KMnO_4$, R_f = 0.76) yielding compound **14** as orange oil (4.29 g, 87% yield). The NMR agrees with literature.[34] **^{1}H-NMR** (300 MHz, $CDCl_3$) δ 9.76 (s, 1H), 3.66 (s, 3H), 2.52 – 2.40 (m, 2H), 2.39 – 2.25 (m, 2H), 1.73 – 1.57 (m, 4H). **^{13}C-NMR** (75 MHz, $CDCl_3$) δ 202.21, 173.82, 51.72, 43.62, 33.83, 24.46, 21.61.

6.5 Methyl-(*E*/*Z*)-7-cyanooct-6-enoate ((*E*/*Z*)-**15**)

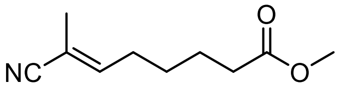

The synthesis was performed as previously reported[35] and under inert conditions. A solution of diethyl-(1-cyano-ethyl)-phosphonate (2.3 mL, 13.1 mmol, 1.27 eq.) in anhydrous THF (14 mL) was added dropwise under stirring to a solution of *n*-BuLi (2.5 M in hexanes, 5.32 mL, 13.3 mmol, 1.29 eq.) in anhydrous THF (4.7 mL) at 0 °C. After complete addition, the cooling bath was removed and the mixture was stirred for 1 h. The resulting dark yellowish mixture was charged with a solution of **14** (1.49 g, 10.3 mmol, 1 eq.) in anhydrous THF (4.6 mL) *via* dropwise addition. Stirring was continued and whenever the reaction mixture warmed up it was cooled with an ice bath. After 90 min, TLC (silica, cyclohexane/EtOAc 7:3, detection: $KMnO_4$, R_f (product) = 0.33 & 0.40) confirmed complete conversion of the starting material. The reaction was then treated with sat. aqueous NH_4Cl (70 mL) at 0 °C. After separation of the layers, the organic phase was washed with *d*H_2O (2 x 25 mL). The combined aqueous layers were extracted with Et_2O (4 x 50 mL), and the combined organic phases were dried over anhydrous Na_2SO_4, filtered, and concentrated *in vacuo* to yield a dark-yellowish oil as crude product (2.36 g, 127%). The crude product was purified by flash column chromatography (silica; cyclohexane/EtOAc = 7:3, detection: $KMnO_4$) to yield **15** (1.65 g; 88% yield) as a pale-yellow oil and as a 60:40 mixture of the *E* and *Z*-isomers as determined by ^{1}H-NMR spectroscopy (based on integrals of the signals at δ 6.1 and 6.3). **^{1}H-NMR** (300 MHz, $CDCl_3$) δ 6.32 (td, *J* = 7.5, 1.6 Hz, 1H, minor isomer), 6.12 (td, *J* = 7.6, 1.6 Hz, 1H, major isomer), 3.66 (s, 3H), 2.46 – 2.27 (m, 4H), 2.25 – 2.11 (m, 1H), 1.92 (d, *J* = 1.4 Hz, 3H, major isomer), 1.85 (d, *J* = 1.4 Hz, 3H, minor isomer), 1.73 – 1.56 (m, 2H), 1.54 – 1.38 (m, 2H). **^{13}C NMR** (75 MHz, CDCl3) δ 173.94, 173.82, 147.87, 147.79, 120.74,

118.22, 109.78, 109.73, 51.70, 34.06, 33.79, 31.30, 28.31, 28.11, 27.78, 24.53, 24.38, 20.19, 14.97, 14.36.

6.6 (*E*/*Z*)-7-cyanooct-6-enoic acid ((*E*/*Z*)-**12a**)

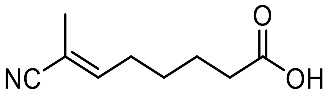

A round bottom flask (100 mL) was charged with KPi-buffer (1 M, pH 8, 40 mL), in which compound **15** (185.5 mg, 1.02 mmol) was dissolved. After addition of Novozyme 435 (500 mg) the flask was closed with a stopper and parafilm and incubated in a bioshaker at 30 °C (50 mm shaking throw, 110 rpm) for 5 h. After this time TLC (silica; cyclohexane/EtOAc 3:2, detection: $KMnO_4$, R_f (product) = 0.67 & 0.72) confirmed complete consumption of the substrate and the reaction was worked up as follows. Novozyme 435 was filtered off and the paper filter rinsed with dH_2O (10 mL). The filtrate was then acidified to pH 2 with HCl (aq.; 6 M). The solution was then extracted with EtOAc (3 x 80 mL). The combined organic layers were dried over anhydrous Na_2SO_4, filtered and the solvent was evaporated under reduced pressure yielding a pale-yellowish oil of compound (*E*/*Z*)-**12a** (160 mg), alongside colorless precipitate. The crude was purified *via* flash column chromatography (silica; cyclohexane/EtOAc 1:1 + 0.5% v/v TFA; detection: $KMnO_4$, R_f = 0.67 & 0.72) yielding compound (*E*/*Z*)-**12a** as a pale yellowish oil solid (150 mg, 88% yield), and as a 60:40 mixture of the *E*- and *Z*-isomers as determined by ^{1}H-NMR spectroscopy (signals at δ 6.1 and 6.3). **^{1}H-NMR** (300 MHz, $CDCl_3$) δ 9.83 (bs, 1H), 6.34 (td, *J* = 7.5, 1.6 Hz, 1H, minor isomer), 6.14 (td, *J* = 7.6, 1.6 Hz, 1H, major isomer), 2.49 – 2.10 (m, 4H), 1.93 (d, *J* = 1.4 Hz, 3H, major isomer), 1.87 (d, *J* = 1.6 Hz, 3H, minor isomer), 1.75 – 1.59 (m, 2H), 1.59 – 1.40 (m, 2H). **^{13}C-NMR** (75 MHz, $CDCl_3$) δ 179.80, 179.65, 147.94, 147.83, 120.57, 118.12, 109.88, 109.78, 33.73, 31.29, 28.29, 27.99, 27.67, 24.22, 24.06, 20.17, 14.96.

6.7 General procedure for the synthesis of 1,4-adducts (Intermolecular)

All the glassware was dried overnight before the synthesis in an oven at 90 °C. A two-step synthesis was required to obtain the intermolecular 1,4-adducts. The first step, the synthesis of the required Grignard reagents from the corresponding bromoalkanes, is directly followed by the conjugate addition to the cyclic alkenones.

Synthesis of the Grignard reagents: A 100 mL 3-neck round-bottom flask, equipped with a stirring bar, a gas inlet and a reflux condenser were purged with argon, and evacuated three times to accomplish dry and inert conditions. Magnesium turnings (2.2 eq) and a small crystal of I_2 were added to the flask under argon flow. The flask was heated with a heat gun for a few seconds to sublimate I_2. Using a syringe, dry diethyl ether (8 mL) was added to the flask under argon flow. The mixture was stirred for 2 minutes giving a yellowish solution. A small amount of bromoalkane (300 µL) was added dropwise and then the flask was heated slightly to start the reaction (discoloration of the solution and gas

evolution). Once the reaction started, the remaining bromoalkane (in total 2 eq.) was added dropwise over a period of 20 minutes. From time to time the flask was heated to keep the reaction under reflux. The reaction was allowed to stir for 30 min at room temperature with occasional heating to maintain reflux. After this time, the solution turned grey with some remaining pieces of Mg. Finally, dry diethyl ether (5 mL) was added and stirring was continued for 2 min.

Conjugate addition: A 100 mL 3-neck round-bottom flask was equipped with a stirring bar and a gas inlet. The flask was flushed with argon and evacuated three times to accomplish dry and inert conditions. Using a syringe, the solution of the Grignard reagent from the first step was added (without any remaining magnesium pieces). CuI (15% mol) was added under agon flow at 0 °C and the solution was stirred for 15 minutes. The color of the solution gradually changed from gray to brown and finally black. A solution of the cyclic enone (1 eq.) in dry diethyl ether (10 mL) was added dropwise over 30 minutes on an ice bath. The resulting solution was stirred at 25 °C for 1 hour until TLC confirmed complete conversion of the cyclic enone. The workup and purification are reported at the individual reactions.

6.7.1 3-tridecylcyclopentan-1-one (**4a**)

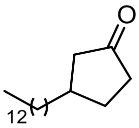

Reagents: Bromoalkane: 1-bromotridecane (1.7 mL, 6.6 mmol, 2 eq.); Enone: 2-cyclopen-1-one (276 µL, 3.3 mmol, 1 eq.); Magnesium turnings (0.1774 g, 7.3 mmol, 2.2 eq); CuI (0.0943 g, 15% mol).

For the workup of this reaction, methanol (20 mL) was added and the reaction mixture was stirred for 10 minutes. The solution was filtered through silica and the silica was washed with pentane (30 mL). The solution was concentrated *in vacuo* to yield a yellow solid (1.81 g). The crude product was purified *via* flash column chromatography (silica; diethyl ether/pentane = 5:95) to yield the 3-tridecylcyclopentan-1-one **4a** as a yellow solid (594.5 mg; 67% yield). **^{1}H-NMR** (300 MHz, $CDCl_3$) δ 2.51 – 1.93 (m, 4H), 1.91 – 1.65 (m, 1H), 1.62 – 0.95 (m, 24H), 0.87 (t, *J* = 6.4 Hz, 3H). **^{13}C-NMR** (75 MHz, $CDCl_3$) δ 220.28, 45.46, 38.67, 37.35, 35.84, 32.05, 29.84, 29.82, 29.79, 29.76, 29.72, 29.68, 29.49, 28.01, 22.82, 14.25.

6.7.2 3-undecylcyclopentan-1-one (**4b**)

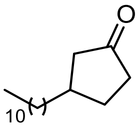

Reagents: Bromoalkane: 1-bromoundecane (1.34 mL, 6 mmol, 2 eq.); Enone: 2-cyclopenten-1-one (251 µL, 3 mmol, 1 eq.); Magnesium turnings (0.1604 g, 6.6 mmol, 2.2 eq.); CuI (0.0857 g, 15% mol)

For the workup of the reaction, methanol (40 mL) was added and the reaction mixture was stirred for 10 minutes. The solution was transferred to a separation funnel and brine (20 mL) was added. The organic layer was separated and the aqueous phase was extracted with diethyl ether (3 x 30 mL). The combined organic layers were washed with brine, dried over Na_2SO_4, filtered, and concentrated *in vacuo* to yield a pale-yellow waxy solid (0.82 g). The crude product was purified *via* flash column chromatography (silica; diethyl ether/pentane = 5:95) to yield the 3-undecylcyclopentan-1-one **4b** as a yellow liquid (163.6 mg; 23% yield). **^{1}H-NMR** (300 MHz, $CDCl_3$) δ 2.48 – 2.00 (m, 4H), 1.88 – 1.69 (m, 1H), 1.63 – 0.99 (m, 22H), 0.87 (t, *J* = 6.8 Hz, 3H). **^{13}C-NMR** (75 MHz, $CDCl_3$) δ 220.41, 45.45, 38.67, 37.33, 35.82, 32.04, 29.83, 29.78, 29.75, 29.71, 29.67, 29.47, 28.00, 22.81, 19.84, 14.23.

6.7.3 3-tridecylcyclohexan-1-one (**4c**)

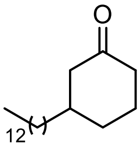

Reagents: Bromoalkane: 1-bromotridecane (1.7 mL, 6.6 mmol, 2 eq.); Enone: 2-cyclohexen-1-one (319 µL, 3.3 mmol, 1 eq.); Magnesium turnings: (0.1764 g, 7.26 mmol, 2.2 eq); CuI: (0.0951 g, 15% mol)

For the workup of this reaction, aqueous NH_4Cl (1M, 40 mL) was added to the final reaction mixture followed by stirring for 10 minutes. The organic layer was separated, and the aqueous phase was extracted with diethyl ether (3 x 30 mL). The combined organic layers were washed with brine, dried over Na_2SO_4, filtered, and concentrated *in vacuo* to yield a yellow oil (1.75 g). The crude product was purified by flash column chromatography (silica; diethyl ether/pentane = 5:95) to yield 3-tridecylcyclohexan-1-one **4c** as a brown solid (723.1 mg; 78% yield). **^{1}H-NMR** (300 MHz, $CDCl_3$) δ 2.48 – 2.13 (m, 4H), 2.10 – 1.51 (m, 5H), 1.25 (s, 24H), 0.87 (t, *J* = 6.4 Hz, 3H). **^{13}C-NMR** (75 MHz, $CDCl_3$) δ 212.32, 48.39, 41.68, 39.23, 36.77, 32.06, 31.48, 29.82, 29.79, 29.76, 29.73, 29.50, 26.79, 25.47, 22.83, 14.26.

6.7.4 *Rac*-2-methyl-3-tridecylcyclopentan-1-one (**4d**)

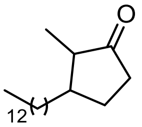

Reagents: Bromoalkane: 1-bromotridecane (1.7 mL, 6.6 mmol, 2 eq.); Enone: 2-methylcyclopent-2-en-1-one (324 µL, 3.3 mmol, 1 eq.); Magnesium turnings: (0.1775 g, 7.3 mmol, 2.2 eq); CuI: (0.0943 g, 15% mol)

For the workup of this reaction, methanol (20 mL) was added to the reaction and the mixture was stirred for 10 minutes. The solution was filtered through celite and the celite was washed with diethylether (30 mL). The combined organic layers were concentrated *in vacuo* to yield a yellow solid alongside an oily residue as crude product (1.34 g). This was purified *via* flash column chromatography (silica; cyclohexane/ethyl acetate = 95:5) to yield *rac*-**4d** as a yellow oil in a mixture of diastereomers (101.1 mg; 11% yield; de (*trans*/*cis*) = 73%). **^{1}H-NMR** (300 MHz, $CDCl_3$) δ 2.52 – 1.88 (m, 3H), 1.78 – 1.54 (m, 3H), 1.26 (m, 24H), 1.06 (d, J = 6.5 Hz, 3H, major isomer), 0.96 (d, J = 7.4 Hz, 3H, minor isomer), 0.87 (t, J = 6.5 Hz, 3H). **^{13}C-NMR** (75 MHz, $CDCl_3$) δ 221.79, 50.67, 47.08, 44.93, 40.20, 37.58, 36.38, 34.67, 32.07, 30.03, 29.99, 29.84, 29.82, 29.79, 29.76, 29.51, 29.34, 27.94, 27.35, 27.26, 25.67, 22.84, 14.27, 12.78, 9.85.

6.8 (2*S*, 3*R*)-2-methyl-3-tridecylcyclopentan-1-one (2*S*, 3*R*)-**4d**

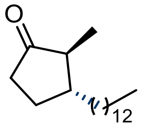

Reagents: Bromoalkane: 1-bromotridecane (0.421 mL, 1.65 mmol, 2 eq.); Enone: 2-methylcyclopent-2-en-1-one (81 µL, 0.825 mmol, 1 eq.); Magnesium turnings: (0.0441 g, 1.815 mmol, 2.2 eq); $CuBr{\cdot}SMe_2$: (0.0084 g, 0.041 mmol, 0.05 eq.), (*S*, R_{Fe})-Rev-Josiphos (0.0294 mg, 0.05 mmol, 0.06 eq., BLDpharm; CAS: 162291-01-2)

The synthesis of the Grignard reagent starting from 1-bromotridecane was carried out as described in **section 6.7**. The enantioselective 1,4-addition was carried out according to literature using (*S*, R_{Fe})-Rev-Josiphos as chiral catalyst, as follows.[36] A 3-neck round-bottom flask, equipped with a stirring bar and a gas inlet, was heated, evacuated and flushed with argon for 3 times to accomplish dry and inert conditions. Then, CuBr·SMe2, Rev-Josiphos and *t*-BuOMe anhydrous (11.5 mL) were added and allowed to stir for 15 min, followed by the addition of the cyclic enone, dissolved in *t*-BuOMe (5 mL). The solution was cooled down to -78°C with an acetone/N_2 (l) bath and then stirred for 30 min. The Grignard reagent was then added dropwise over 15 min and the reaction was left to stir at -78°C for 4h. The reaction was stopped by the addition of MeOH (10 mL) and allowed to warm to ambient temperature. After diluting the mixture with Et_2O (10 mL), sat. aqueous NH_4Cl (20 mL) was added while stirring and then the layers were separated. The aqueous phase was extracted with Et_2O (3x30 mL) and

the combined organic layers were dried with anhydrous Na_2SO_4, filtered and concentrated *in vacuo*, yielding a yellowish oil, alongside a red-orange solid residue (351.2 mg). The crude product was purified *via* flash column chromatography (silica; cyclohexane/ethyl acetate = 95:5) to yield (2*S*, 3*R*)-2-methyl-3-tridecylcyclopentan-1-one **4d** as a yellow oil in a mixture of diastereomers (24.1 mg; 10% yield; de (*trans*/*cis*) = 75%; ee (*trans*) = 75%; ee (*cis*) = 68%). **^{1}H-NMR** (300 MHz, $CDCl_3$) δ 2.51 – 1.78 (m, 3H), 1.78 – 1.59 (m, 3H), 1.40 – 1.13 (m, 24H), 1.07 (d, *J* = 7.0 Hz, 3H, major isomer), 0.97 (d, *J* = 7.4 Hz, 3H, minor isomer), 0.88 (t, *J* = 6.5 Hz, 3H). **^{13}C-NMR** (75 MHz, $CDCl_3$) δ 221.79, 50.67, 44.95, 41.76, 37.59, 34.69, 32.07, 30.04, 29.82, 29.80, 29.77, 29.51, 27.37, 27.27, 27.06, 23.66, 22.84, 14.27, 12.81.

6.9 (*Z*)-tridec-4-ene (8b)

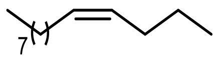

To a three-neck round-bottom flask (100 mL), equipped with a stirring bar and a gas inlet butyltriphenylphosphonium bromide (1.50 g, 3.75 mmol, 1.5 eq.) was added as solution in anhydrous THF (25 mL), followed by the addition of NaHMDS (1.0 M in THF, 3.75 mL, 3.75 mmol, 1.5 eq.) at 0 °C under an argon atmosphere. The mixture was stirred at 0 °C for 1 h and the resulting reddish-orange mixture was cooled to -90 °C, using a N_2/acetone cooling bath. A solution of nonanal (430 µL, 2.50 mmol, 1 eq.) in anhydrous THF (7.5 mL) was added to the mixture dropwise within 10 min, followed by stirring for 1 h at -90 °C. After this time the mixture was allowed to warm to 0 °C within 2 h. TLC (silica, cyclohexane/EtOAc = 9:1 detection: UV and $KMnO_4$, Rf (product) = 0.94) confirmed complete conversion of the starting material and the reaction was quenched by adding sat. aqueous NH_4Cl (40 mL) at 0 °C. After separating the organic phase, the aqueous layer was extracted with Et_2O (3 x 50 mL), and the combined organic phases were washed with brine (2 x 50 mL). The solution was dried over anhydrous Na_2SO_4, filtered, and concentrated *in vacuo* to yield a pale-yellow waxy solid (1.47 g). The crude product was purified by flash column chromatography (silica; cyclohexane/EtOAc = 9:1) to yield (*Z*)-**8b** as a colorless liquid (313 mg; 69% yield). **^{1}H-NMR** (500 MHz, $CDCl_3$) δ 5.40-5.31 (m, 2H), 2.04 – 1.98 (m, 4H), 1.41 – 1.12 (m, 14H), 0.96 – 0.81 (m, 6H). **^{13}C-NMR** (125 MHz, $CDCl_3$) δ 130.28, 129.77, 32.07, 29.94, 29.68, 29,49, 29.48, 29.45, 27.39, 23.06, 22.84, 14.26, 13.96. The NMR signals for **8b** agree with literature.[37]

6.10 (*Z*)-tridec-5-ene (9b)

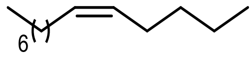

To a three-neck round-bottom flask (100 mL), equipped with a stirring bar and a gas inlet pentyltriphenylphosphonium bromide (1.55 g, 3.75 mmol, 1.5 eq.) was added as solution in anhydrous THF (25 mL), followed by the addition of NaHMDS (1.0 M in THF, 3.75 mL, 3.75 mmol, 1.5 eq.) at 0 °C under argon atmosphere. The mixture was stirred at 0 °C for 1 h and the resulting reddish-orange mixture was cooled to -90 °C with a N_2/acetone cooling bath. A solution of octanal (391 µL, 2.50 mmol, 1 eq.) in anhydrous THF (7.5 mL) was added dropwise within 10 min to the mixture,

followed by stirring for 1 h at -90 °C. After this time the mixture was allowed to warm up to 0 °C within 2 h. TLC (silica, cyclohexane/EtOAc = 9:1 detection: UV and $KMnO_4$, R_f (product) = 0.91) confirmed complete conversion of the substrate and the reaction was quenched by adding sat. aqueous NH_4Cl (40 mL) at 0 °C. After separating the organic phase, the aqueous layer was extracted with Et_2O (3 x 50 mL), and the combined organic phases were washed with brine (2 x 50 mL). The solution was dried over anhydrous Na_2SO_4, filtered, and concentrated *in vacuo* to yield a pale-yellow liquid (2.40 g). The crude product was purified by flash column chromatography (silica; cyclohexane/EtOAc = 9:1) to yield (*Z*)-**9b** as a colorless liquid (303 mg; 66% yield). **^{1}H-NMR** (500 MHz, $CDCl_3$) δ 5.38 – 5.32 (m, 2H), 2.06 – 1.95 (m, 4H), 1.47 – 1.13 (m, 14H), 0.94 – 0.83 (m, 6H). **^{13}C-NMR** (125 MHz, $CDCl_3$) δ 130.07, 130.00, 32.14, 32.04, 29.94, 29.44, 29.38, 27.36, 27.08, 22.83, 22.51, 14.26, 14.15. The NMR signals for **9b** agree with literature.[38]

6.11 1-octylcyclopentan-1-ol (16)

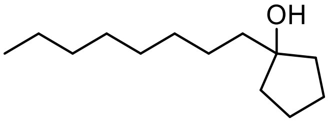

Magnesium turnings (201 mg, 8.25 mmol, 1.65 eq.), and a small crystal of iodine were added to a three-neck round-bottom flask, equipped with a stirring bar, a reflux condenser, a dropping funnel, and a septum. Next, a portion of a solution 1-bromo-octane (1.30 mL, 7.50 mmol, 1.50 Eq.) in anhydrous THF (6.2 mL) was added to just cover the Mg-turnings. The reaction was started by gentle heating, upon which the brownish color of the iodine vanished. The rest of the halogenalkane solution was added dropwise at a rate that the exothermic reaction was maintained while gentle stirring until nearly all the Mg-turnings were dissolved and the solution turned grey. After cooling to 0 °C, a solution of cyclopentanone (442 µL, 5.0 mmol, 1.0 eq.) in anhydrous THF (24.56 mL) was added dropwise to the *in-situ* prepared Grignard reagent, upon which the reaction was allowed to warm to RT and stirred for 3 h. At this time TLC (silica, cyclohexane/EtOAc = 6:4, detection: UV & $KMnO_4$, R_f (product) = 0.86) confirmed almost complete conversion of the starting material. The reaction was quenched by addition of saturated NH_4Cl (aqueous, 20 mL) followed by a dilution with EtOAc (20 mL). The layers were separated, and the aqueous layer was extracted with EtOAc (2 x 20 mL). The combined organic phases were washed with dH_2O (50 mL) and brine (50 mL), dried over anhydrous Na_2SO_4 and filtered. The solvent was removed *in vacuo*, yielding a colorless oil (882 mg), which was purified *via* flash column chromatography (silica; cyclohexane/EtOAc = 6:4, detection: $KMnO_4$) obtaining compound **16** as colorless oil (455 mg, 46%). **^{1}H-NMR** (500 MHz, $CDCl_3$) δ 1.83 – 1.75 (m, 2H), 1.67 – 1.51 (m, 8H), 1.43 – 1.35 (m, 3H), 1.33 – 1.20 (m, 10H), 0.88 (t, *J* = 7.2 Hz, 3H). **^{13}C-NMR** (125 MHz, $CDCl_3$) δ 82.77, 41.69, 39.83, 32.04, 30.46, 29.77, 29.44, 24.85, 23.98, 22.82, 14.25.

6.12 Octylidenecyclopentane (17)

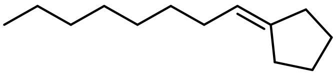
A round bottom flask (500 mL), equipped with a stirring bar, was charged with compound **16** (455 mg, 2.29 mmol, 1.0 eq.) and toluene (184 mL). Next, p-TsOH*H_2O (236 mg, 1.24 mmol, 0.54 eq.) was added. The reaction mixture was heated to reflux for 1 h, which was accompanied by a color change from yellow to orange. After this time, TLC (silica; cyclohexane/EtOAc = 9:1, detection: $KMnO_4$, R_f (product) = 0.88) confirmed complete conversion of the starting material and the reaction was worked up by the addition of sat. $NaHCO_3$ (100 mL). The organic layer was separated and washed with brine (2 x 50 mL), dried over anhydrous Na_2SO_4, filtered, and concentrated *in vacuo* to yield a yellow-orange oil (562 mg). The crude was purified *via* flash column chromatography (silica; cyclohexane/EtOAc 9:1; detection: UV and $KMnO_4$) yielding an isomeric mixture of **17** as yellowish oil (367 mg, 89% yield). **^{1}H-NMR** (500 MHz, $CDCl_3$) δ 5.32-5.29 (m, 1H), 2.31 – 2.27 (m, 2H), 2.24 – 2.19 (m, 2H), 2.07 – 2.02 (m, 2H), 1.84 (m, 2H), 1.43 (m, 2H, + cyclohexane impurity), 1.32 – 1.22 (m, 10H), 0.88 (t, *J* = 7.2 Hz, 3H). **^{13}C-NMR** (125 MHz, $CDCl_3$) δ 145.32, 123.06, 35.22, 32.57, 32.07, 31.37, 29.72, 29.68, 29.46, 28.00, 23.60, 22.84, 14.27.

6.13 Octylcyclopentane (9c)

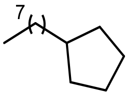

A three-necked round-bottom flask (100 mL), equipped with a stirring bar, stopper and 3-way valve adapter was charged with Pd/C (10% Pd, 106.2 mg; corresponding to 10.62 mg Pd, 0.1 mmol, 0.05 eq.). The catalyst was quickly covered with EtOAc (5 mL) upon which a solution of **17** (366.7 mg, 2.03 mmol, 1.0 eq.) in EtOAc (5 mL) was added. Immediately, a balloon filled with H_2 was connected under Ar counterflow to the three-way adapter. The system was then evacuated three times with a water jet pump until the solvent just started to evaporate and then reconnected to the H_2 from the balloon. After this, the reaction mixture was stirred vigorously (700 rpm) at room temperature. Reaction progress was monitored on GC-MS, which confirmed complete consumption of the substrate after 3 1/2 h. The reaction mixture was filtered through celite, the celite rinsed two more times with EtOAc (2 x 20 mL) and the solvent concentrated under reduced pressure affording compound **9c** (365 mg, 99 %) in pure form as a colorless liquid. **^{1}H-NMR** (500 MHz, $CDCl_3$) δ 1.77 – 1.69 (m, 3H), 1.62 – 1.55 (m, 2H), 1.53 – 1.45 (m, 2H), 1.33 – 1.20 (m, 14H), 1.09 – 1.00 (m, 2H), 0.88 (t, *J* = 7.2 Hz, 3H). **^{13}C-NMR** (125 MHz, $CDCl_3$) δ 40.35, 36.44, 32.90, 32.10, 30.14, 29.85, 29.53, 28.98, 25.35, 22.86, 14.28.

6.14 2,6-dimethylhept-2-ene (10b)

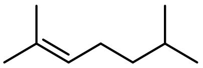
A pear-shaped flask, equipped with a stirring bar was charged with 2,6-dimethylheptane-2-ol (6.1 g, 42.3 mmol, 1 eq.) and connected to a fractionate distillation setup

comprising a Vigreux column. After the addition of 1 drop of H_2SO_4 conc., the neat mixture was heated to 130 °C and distilled at atmospheric pressure. The distilled product was a mixture of 2,6-dimethylhept-2-ene and 2,6-dimethylhept-1-ene and water, which resulted in the generation of two phases. After the distillation was complete, the apparatus was allowed to cool to room temperature and the water was separated. The content of all four receiver flasks was analyzed on GC-MS, to determine the product distribution between the two isomers. The fraction with the highest rel. GC area of compound **10b** was transferred to a microcentrifuge tube (1.5 mL), dried over anhydrous Na_2SO_4, vortexed for 1 min, and centrifuged (14680 rpm, 2 min), and transferred to a glass screw cap vial, obtaining a colorless solution of a mixture of **10b** and the terminal regio-isomer of **10b** in a ratio of 82:18 as determined by **^{1}H-NMR** spectroscopy (signals at δ 5.1 and 4.68). Compound **10b** was stored in the -21 °C freezer and used without further purification. **^{1}H NMR** (300 MHz, CDCl3) δ 5.10 (tt, J = 7.1, 1.4 Hz, 1H, major regioisomer), 4.69–4.66 (d, *J* = 6.5 Hz, 1H, minor regioisomer), 1.97 (q, *J* = 7.4 Hz, 2H), 1.68 (s, 3H), 1.60 (s, 3H), 1.58 – 1.46 (m, 1H), 1.27 – 1.14 (m, 2H), 0.88 (d, *J* = 6.6 Hz, 6H). **^{13}C NMR** (75 MHz, CDCl3) δ 131.13, 125.16, 39.24, 27.77, 26.04, 25.88, 22.78, 22.71, 17.76.

6.15 1-isopropyl-3-methylcyclopentane (10c)

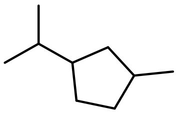

A two-neck round-bottom flask (10 mL), equipped with a stirring bar, alongside a stopper was dried in the 80 °C oven for 30 min. After assembling, a three-way valve and the stopper were attached to the apparatus and inert and dry conditions were accomplished *via* 2 cycles of heating, evacuation and flushing with argon. Afterwards, the round bottom flask was charged with Pd/C (10% w/w Pd; 35 mg) under Ar flow. The stopper was exchanged for a septum, the three-way valve was closed and connected to a balloon filled with H_2, upon which 1-isopropyl-3-methylenecyclopentane was added (300 µL). The three-way valve was opened to the balloon filled with hydrogen and the system was flushed for 30 seconds. The septum was then covered with parafilm and the reaction was vigorously stirred for 5 h at 1250 rpm without the addition of solvent. Reaction progress was followed on GC-MS by diluting a sample of the reaction (1 µL) in EtOAc (1 mL). The reaction was worked up by transferring the whole reaction mixture to a microcentrifuge tube (1.5 mL) and centrifugation at 14680 rpm for 2 min at 4 °C. The supernatant was then transferred to a glass vial, protected with parafilm and immediately stored in the -20 °C freezer without analyzing the yield. GC-MS analysis confirmed a purity of 96%, while ^{1}H-, and ^{13}C-NMR confirms the presence of a diastereomeric mixture (ratio(*cis*/*trans*) = 2:1) of 1-isopropyl-3-methyl cyclopentane (**10c**), as colorless liquid without the presence of any side products. **^{1}H-NMR** (700 MHz, $CDCl_3$) δ 2.04 – 0.99 (m, 8H), 0.95 (dd, *J* = 15.7, 6.5 Hz, 3H), 0.86 (dd, *J* = 6.6, 2.0 Hz, 6H). **^{13}C-NMR** (176 MHz, $CDCl_3$) δ 48.46, 46.61, 40.86, 38.70, 35.37, 34.81, 34.14, 34.00, 33.88, 33.67, 31.48, 29.97, 21.74, 21.65, 21.48, 21.13.

6.16 1-bromo-3-methylcyclopentane (**18**)

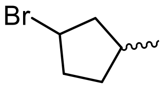

The synthesis was carried out according to literature reports.[39] A two-neck round-bottom flask (100 mL) was charged with a stirring bar, DCM (13 mL), and 3-methylcyclopentane-1-ol (550 µL, 5 mmol, 1 eq., mixture of isomers). The solution was cooled to 0 °C followed by the dropwise addition of PBr_3 (586 µL, 6.25 mmol, 1.25 eq.), upon which the ice bath was removed. The reaction was allowed to stir for 2 h at room temperature, until TLC (silica, cyclohexane/EtOAc = 7:3, detection: $KMnO_4$, R_f (product) = 0.95) confirmed complete consumption of the alcohol. The reaction was worked up by the addition of crushed ice (15 g), followed by extraction of the aqueous phase with DCM (2x25 mL). The combined organic layers were washed with sat. solution of $NaHCO_3$ (25mL), water (40 mL) and brine (40 mL), then dried over anhydrous Na_2SO_4 and concentrated under reduced pressure, yielding **18** as pale-yellow liquid (514 mg). The crude product was purified *via* flash column chromatography (silica; DCM, detection: $KMnO_4$) to yield **18** as a colorless oil in a mixture of diastereomers (314 mg; 38% yield). The observed NMR shifts agree with literature.[39] **^{1}H-NMR** (300 MHz, $CDCl_3$) δ 4.58 – 4.44 (m, 0.7H), 4.35 – 4.23 (m, 0.2H), 2.54 – 1.75 (m, 5H), 1.73 – 1.60 (m, 1H), 1.56 – 1.40 (m, 1H), 1.06 (dd, J = 23.5, 6.7 Hz, 3H). **^{13}C-NMR** (75 MHz, $CDCl_3$) δ 53.81, 50.94, 46.59, 46.42, 38.05, 37.87, 33.75, 32.88, 32.58, 32.08, 21.28, 20.58.

6.17 2-(3-methylcyclopentyl)propane-2-ol (**10d**)

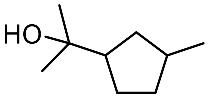

Magnesium turnings (86 mg, 3.55 mmol, 1.65 eq.), a small crystal of iodine and anhydrous THF (1 mL) were added to a three-neck round-bottom flask, equipped with a stirring bar, a reflux condenser, a dropping funnel, and a septum, followed by the dropwise addition of a solution of **18** (350 mg, 2.15 mmol, 1.50 Eq.) in anhydrous THF (2.2 mL). The generation of the Grignard reagent was started by gentle heating, upon which the brown color of the iodine vanished. The rest of the alkyl halide solution was added dropwise at a rate that maintained the exothermic reaction while stirring until nearly all the Mg-turnings were dissolved and the solution turned grey. After cooling to 0 °C, a solution of acetone (distilled, 110 µL, 1.43 mmol, 1.0 eq.) in anhydrous THF (7.2 mL) was added dropwise to the *in-situ* prepared Grignard reagent, upon which the reaction was allowed to warm to RT and stirred for 3 h. At this time TLC (silica, cyclohexane/EtOAc = 1:1, detection: $KMnO_4$, R_f (product) = 0.71) confirmed almost complete conversion of the starting material. The reaction was quenched by addition of saturated NH_4Cl (aqueous, 6 mL) followed by a dilution with EtOAc (20 mL). The layers were separated, and the aqueous layer was extracted with EtOAc (2 x 20 mL). The combined organic phases were washed with dH_2O (25 mL) and brine (25 mL), dried over anhydrous Na_2SO_4 and filtered. The solvent was removed *in vacuo*, yielding a yellowish residue (73 mg), which was purified

via flash column chromatography (silica; cyclohexane/EtOAc = 1:1, detection: $KMnO_4$) obtaining compound **10d** as colorless liquid as a mixture of diastereomers (20.1 mg, 10%, ratio(*cis*/*trans*) = 2:1). **^{1}H-NMR** (700 MHz, $CDCl_3$) δ 2.13 – 1.20 (m, 7H), 1.17 (d, *J* = 3.1 Hz, 6H), 1.14 – 1.06 (m, 1H), 0.98 (dd, *J* = 19.4, 1.7 Hz, 3H), 0.92 (q, *J* = 11.4 Hz, 1H). **^{13}C-NMR** (176 MHz, $CDCl_3$) δ 72.70, 72.41, 51.40, 49.90, 37.18, 35.55, 35.34, 35.08, 34.38, 34.35, 28.19, 28.10, 27.91, 27.81, 26.37, 21.20, 21.13, 20.59.

7 Analytical Methods

7.1 Achiral GC-FID analysis

Achiral GC analysis was carried out using an Agilent 7890A GC-FID System [7693 Autosampler], equipped with an HP-5 capillary column (dimensions: 30 m x 0.25 mm x 0.25 µm; stationary phase: bonded & cross-linked 14 %-cyanopropylphenyl-methylpolysiloxane) using helium as carrier gas. The corresponding retention times of the analytes of given methods are reported in the captions alongside the GC-FID chromatograms in **section 8**. Injection volumes were set to 5 µL.

Table S29. *Achiral GC-FID methods.*

Substrate		
	**Method GC-1**	
**1a**-**2a** + **3a**-**3c**	column:	HP-5
	injector temperature:	300 °C
	detector temperature:	300 °C
	split ratio:	50:1
	flow rate:	1 ml/min
	temperature program:	40 °C (5 min); 12 °C/min to 300 °C (2 min)
	total runtime:	28.7 min
	**Method GC-2**	
**5a**-**6a**	column:	HP-5
	injector temperature:	200 °C
	detector temperature:	300 °C
	split ratio:	50:1
	flow rate:	1 mL/min
	temperature program:	40 °C (4 min); 4 °C/min to 80 °C (0 min); 15 °C/min to 230 °C (1 min)
	total runtime:	25 min
	**Method GC-3**	
**7a**, **10a**, **12a**	column:	HP-5
	injector temperature:	200 °C
	detector temperature:	300 °C
	split ratio:	50:1
	flow rate:	1.1 mL/min
	temperature program:	35 °C (3 min); 2 °C/min to 44 °C (8 min); 10 °C/min to 70 (0 min); 30 °C/min to 220 °C (2 min)
	total runtime:	25.1 min
	**Method GC-4**	
(*Z*)-**8a**, (*Z*)-**9a**, (*E*)-**11a**, (*Z*)-**11a**	column:	HP-5
	injector temperature:	300 °C
	detector temperature:	300 °C
	split ratio:	50:1
	flow rate:	1 mL/min
	temperature program:	60 °C (0.5 min); 10 °C/min to 300 °C (0 min)
	total runtime:	24.5 min
	**Method GC-5 (Protein Engineering)**	
rac-**10a**	column:	HP-5
	injector temperature:	200 °C
	detector temperature:	300 °C
	split ratio:	50:1
	flow rate:	1.1 mL/min
	temperature program:	40 °C (3 min); 2 °C/min to 44 °C (7 min); 60 °C/min to 250 (3 min)
	total runtime:	18.4 min

7.2 Chiral GC-FID analysis

Chiral GC analysis was carried out using an Agilent 7890A GC-FID system and H_2 as carrier gas. Product **4a** and **4c** were analyzed using the Hydrodex-β-TBDAc column (dimensions: 25 m x 0.25 mm ID x 0.4 mm OD), and Hydrodex- β-6TBDM (dimensions: 25 m x 0.25 mm ID) with **method GC-6** and **GC-7** respectively. Product **4d** was analyzed with a CP-Chirasil-Dex-CB (dimensions: 25 m x 0.32 ID) using **method GC-8**, as reported with analogues displaying a shorter alkyl chain.[36] The assignment of the corresponding diastereomers/enantiomers of **4d** are based on the synthesis of an enantioenriched authentic reference as reported in literature.[36] For both analysis, hydrogen was used as carrier gas. Methods used for the determination of enantiomeric excess are summarized in **Table S30**. The corresponding retention times of the analytes of given methods are reported in the captions alongside the GC-FID chromatograms in **section 8**.

Table S30. *Chiral GC-FID methods.*

**Products**	**Method GC-6**	
**4a**	column:	Hydrodex-β-TBDAc
	injector temperature:	300°C
	detector temperature:	300°C
	split ratio:	1:1
	flow rate:	2 ml/min
	temperature program:	80 °C (2 min); 80 °C – 170 °C (10 °C/min); 170 ° C (45 min); 170 °C - 230 °C (5 °C/min)
	total runtime:	68 min
	**Method GC-7**	
**4c**	column:	Hydrodex- β-6TBDM
	injector temperature:	300°C
	detector temperature:	300°C
	split ratio:	1:1
	flow rate:	2 mL/min
	temperature program:	80 °C (2 min); 80 °C – 180 °C (10 °C/min); 180 °C - 190 °C (4°C/min); 190 °C (35 min); 190 °C – 200 °C (1 °C/min); 200 °C (10 min); 200 °C – 220 °C (15 °C/min)
	total runtime:	70.8 min
	**Method GC-8**	
**4d**	column:	CP-Chirasil-Dex-CB
	injector temperature:	300°C
	detector temperature:	300°C
	split ratio:	50:1
	flow rate:	1.6 mL/min
	temperature program:	130°C (8 min); 130°C – 140°C (10°C/min); 140° – 150°C (0.1°C/min); 150°C (77 min); 150 – 200 (10°C/min); 200°C (2 min)
	total runtime:	193 min

7.3 Determination of absolute configuration

For the NMR spectroscopic assignment of distinct signals corresponding to each diastereomer in the diastereomeric mixtures of both, the cyclic alkane and cyclic alcohol, 2D HSQC and HMBC spectra were used. The assignments of the cycloalkane **10c** are shown in **Figure S36**. The two isomers can be distinguished through large differences in the proton chemical shifts of the two diasteroemeric protons on C-2. In the *trans*-isomer the chemical shifts of both protons on C-2 are affected to similar amounts by chemical shift anisotropies of methyl or isopropyl groups at the nearby carbons C-1 and C-3. In contrast in the *cis*-form one proton on C-2 has both substituents of C-1 and C-3 at the same side of the ring, while the other one is on the opposite side of both substituents. This leads to larger chemical shift differences for the protons on C-2 for the *cis*-isomer relative to the *trans* counterpart.

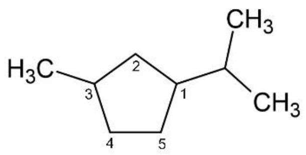

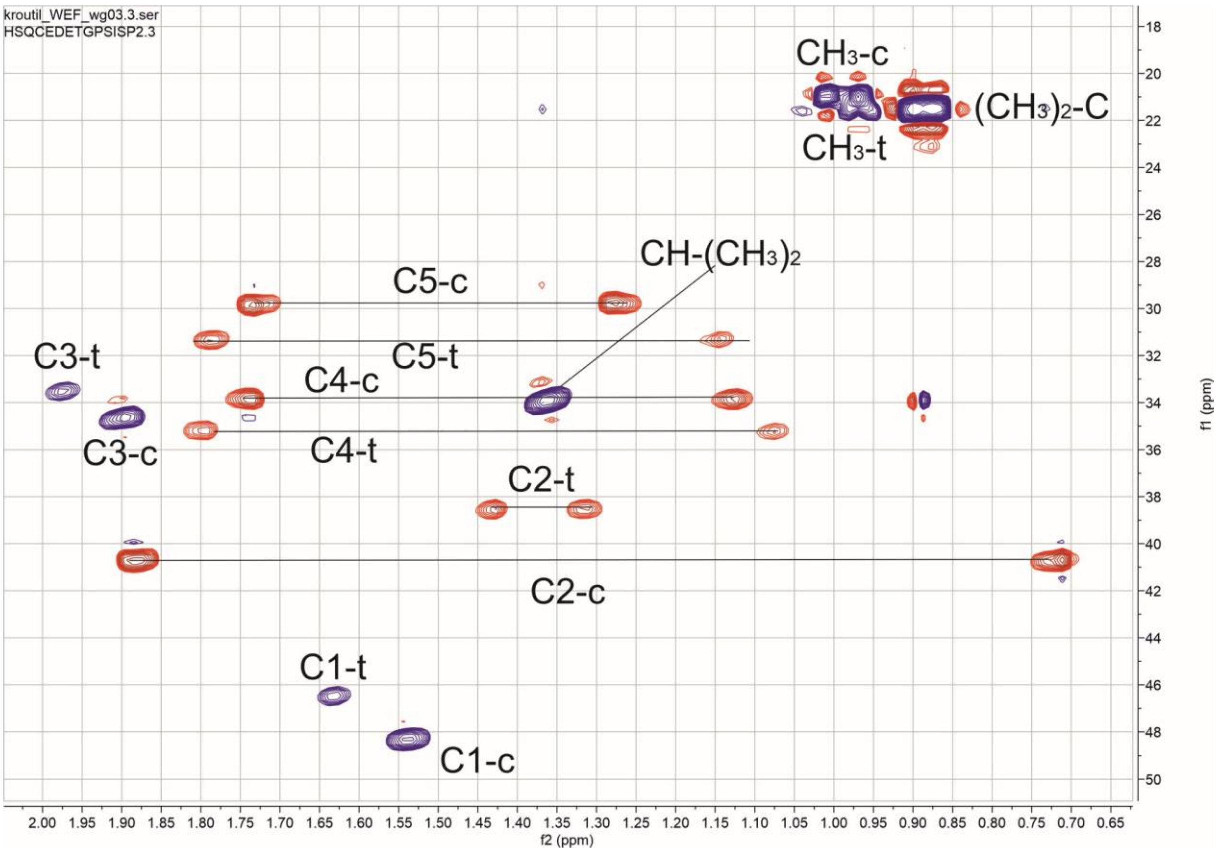

Figure S36. *HSQC of the authentical product reference of the cycloalkane* ***10c****, and its corresponding couplings and chemical assignments.*

The same trend was observed for the alcohol analogue **10d**, albeit the differences in the C-2-protons chemical shift of the *cis*-, and *trans*-isomer were smaller (**Figure S37**).

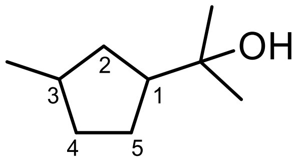

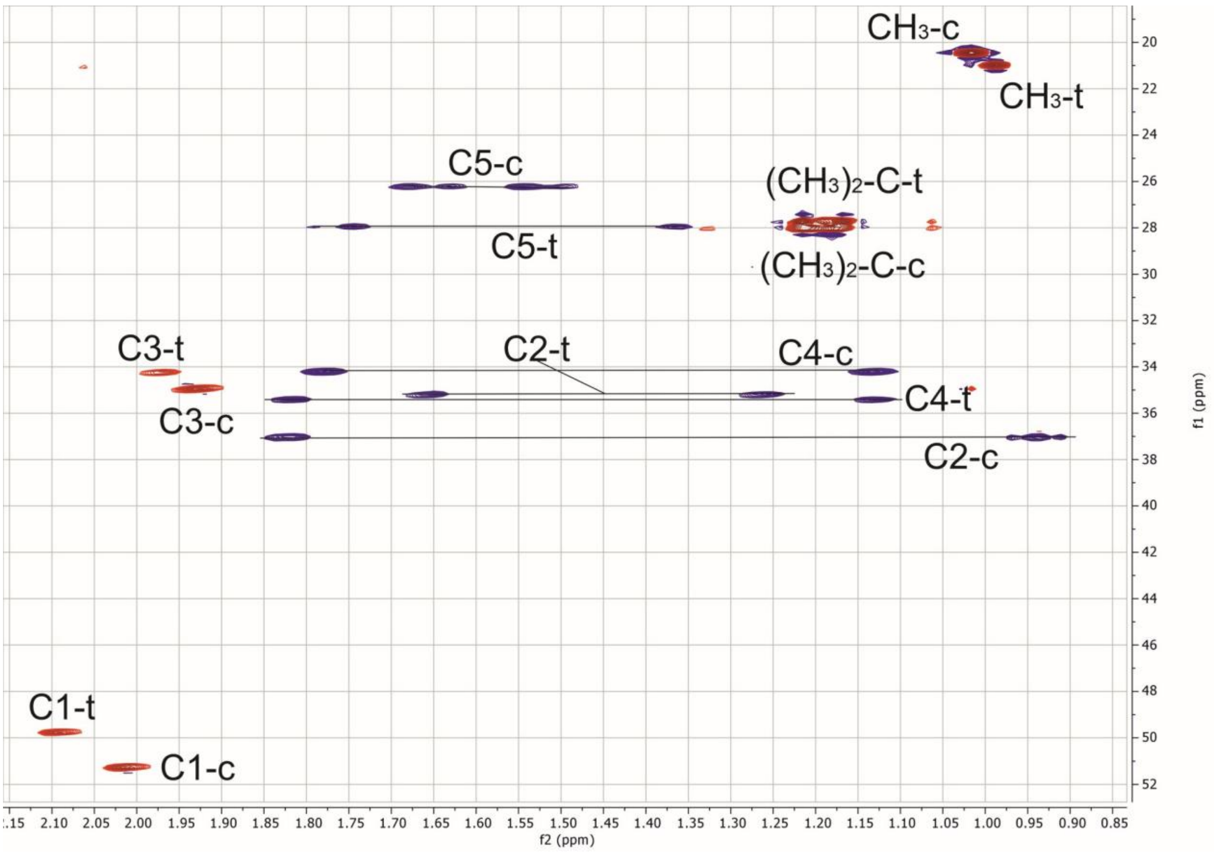

Figure S37. *HSQC of the authentical product reference of the cyclic alcohol* ***10d****, and its corresponding couplings and chemical assignments.*

For both product references of the cyclic alkane **10c** and the cyclic alcohol **10d** the *cis*-isomer is the major form, in a ratio of about 2:1.

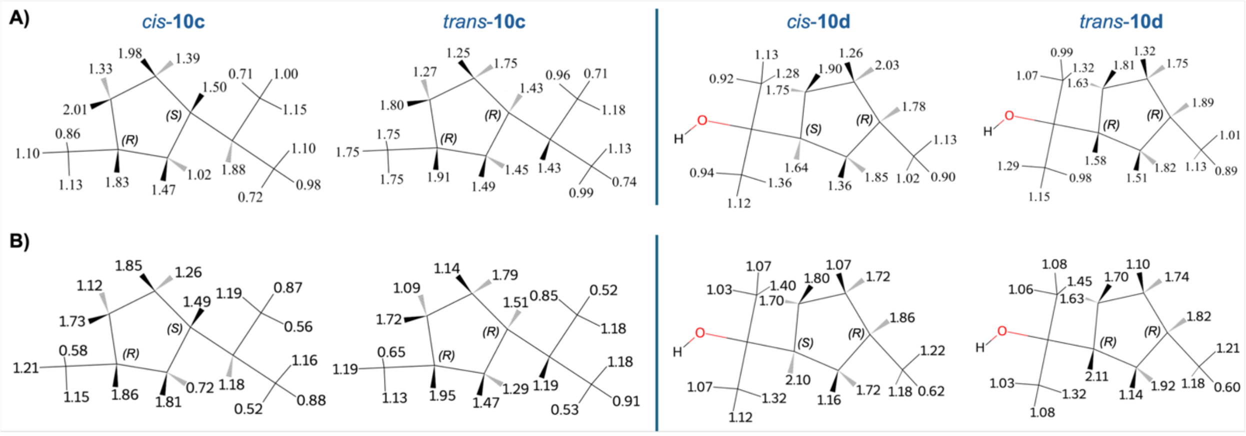

*Figure S38. Computationally predicted ^{1}H-NMR chemical shifts (ppm) for the cis and trans diastereoisomers of compounds **10c** and **10d**. **A)** Chemical shifts predicted using the ML-based CASCADE platform.[40] **B)** Chemical shifts calculated using DFT at the mPW1PW91/6-311+G(d,p)//wB97X-D/6-31+G(d,p) level of theory. All DFT values were referenced to tetramethylsilane (TMS) calculated at the same level of theory.*

Further confirmation for this result could be obtained from computational calculation of the chemical shifts. The chemical shifts for the different diastereoisomers of the cyclic alkane (**10c**) and the cyclic alcohol (**10d**) were evaluated using two complementary computational approaches. First (**Figure S38**, **A**), predictions were obtained from the CASCADE platform developed by the Paton group,[40] through its webserver.[41] This method utilizes a Graph Neural Network (GNN) trained on extensive DFT datasets to provide rapid ML-based NMR predictions. Second (**Figure S38**, **B**), DFT calculations were carried out to estimate chemical shifts. Starting from the optimized structures previously described (wB97X-D/6-31+G(d,p) with the SMD solvation model for water), single-point calculations were performed at the mPW1PW91/6-311+G(d,p) level of theory in Gaussian16.[42] This specific functional and basis set combination was selected to align with the training dataset used in the CASCADE program. ^{1}H-NMR chemical shifts were then estimated using the nmr=(giao,spinspin,mixed) keywords and referenced to the absolute shielding constant of tetramethylsilane (TMS) computed at the same level of theory.

The results obtained from both approaches show high consistency for the cyclic alkane derivatives (**10c**). However, some differences were observed in the case of the cyclic alcohol (**10d**). We attribute these differences to the presence of the hydroxyl group, which appears to interfere with the neighboring stereocenter. Upon integration with the experimental data, the ML-based results (CASCADE) proved to be more reliable, effectively capturing the stereochemical influence of the alcohol group and enabling the correct assignment of the experimentally obtained spectra to each enantiomer.

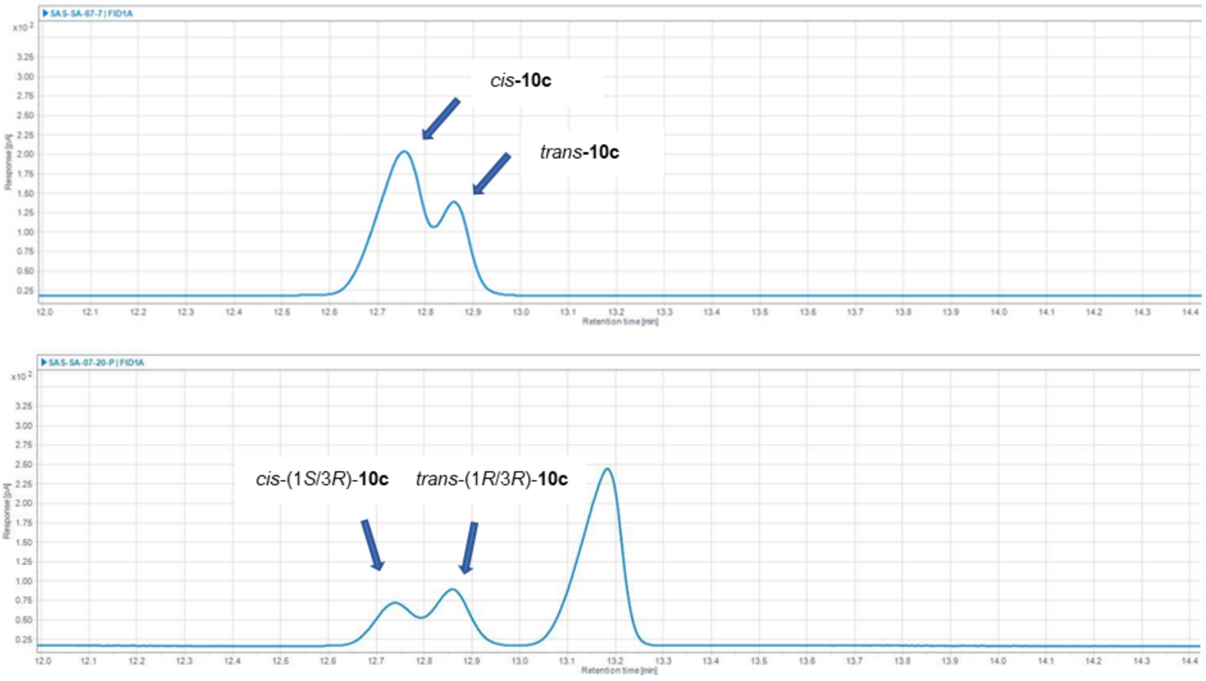

Figure S39. *GC- FID chromatograms of the authentical product reference of* ***10c*** *and the biotransformation using (R)-**10a**. (**top**): authentical product reference of the cyclic alkane **10c**. (**bottom**) Biotransformation of Y466A/V453S in form of resuspended whole cells (OD_{600}/mL = 40, corresponding to a CDW of 20.0 ± 0.2 mg) using (R)-**10a**.*

Concluding, according to the elution profile of the analytes in the GC-FID analysis (***Figure S39***) the slight de observed throughout the directed evolution can be assigned towards the *trans*-(*R*/*R*) configured cyclic product of **10c** when using (*rac*)-, or (*R*)-**10a** as starting material.

Analogous the alcohol **10d** generated in the biotransformations of (*R*)-**10a** must be the *trans*-configured (*R*/*R*) product as well (***Figure S40***).

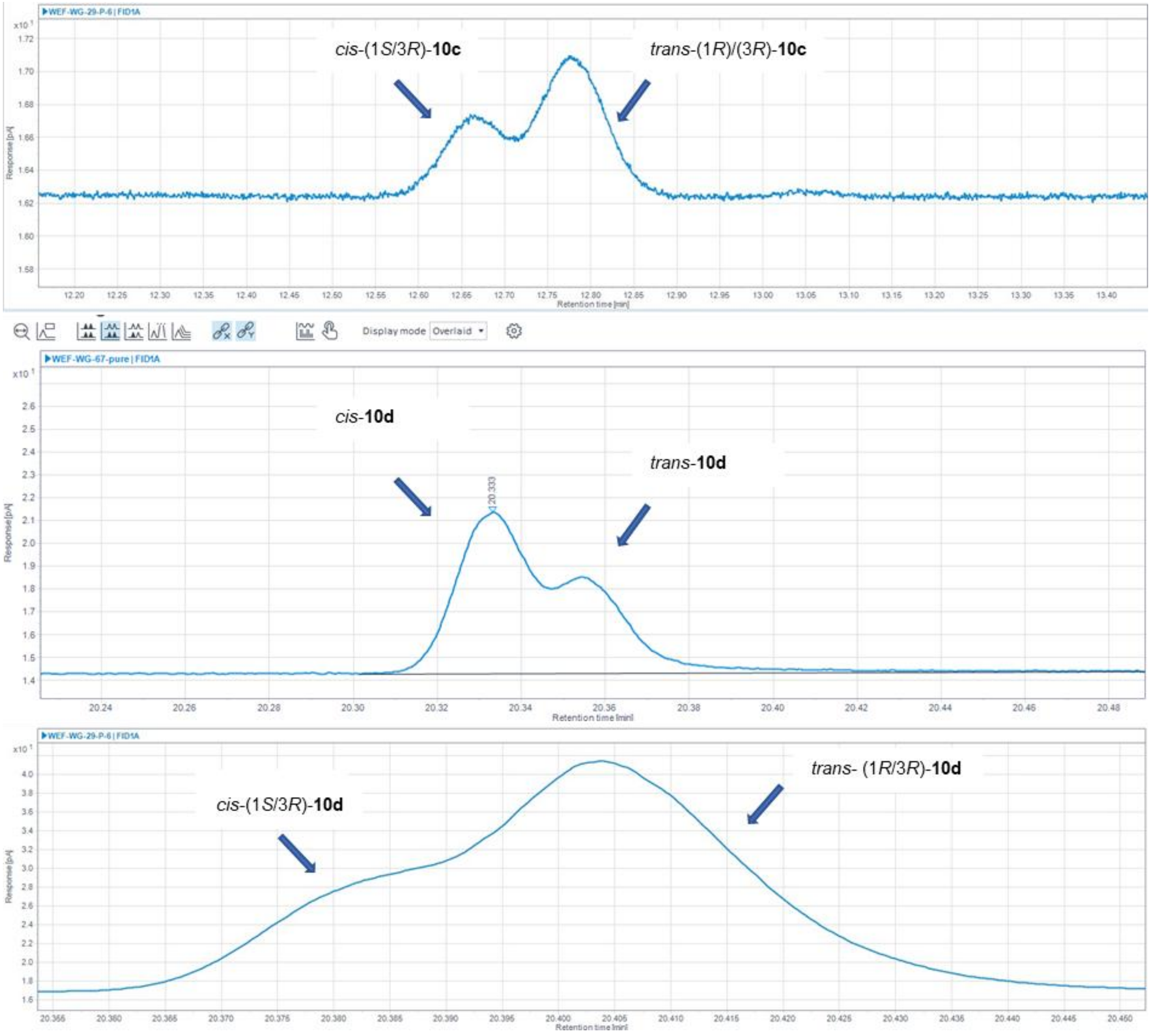

Figure S40. *GC- FID chromatograms of the authentical product reference of* ***10d*** *and biotransformation of (R)-**10a**. (**top**): Biotransformation of CvFAP M3 C432A in form of resuspended whole cells using (R)-**10a**, showcasing traces of formed **10c**. (**middle**): authentical product reference of the cyclic alcohol **10d** and its assignments. (**bottom**): Biotransformation of CvFAP M3 C432A in form of resuspended cells using (R)-**10a**, showcasing peaks corresponding to the cyclic alcohol **10d**.*

7.4 Calibrations

7.4.1 Intermolecular decarboxylative radical coupling with electrophilic C=C bonds:

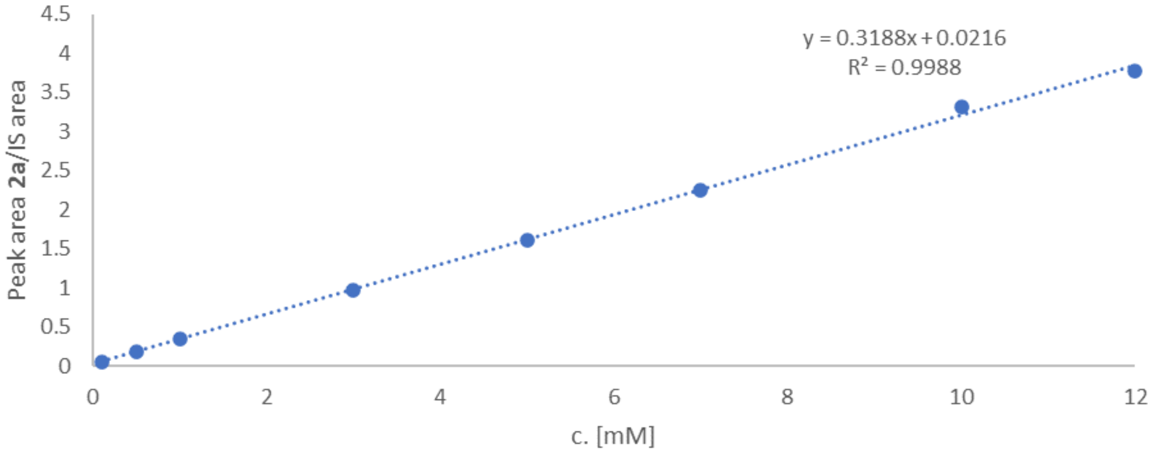

Figure S41. *Calibration curve for the quantification of myristic acid (**1a**)*

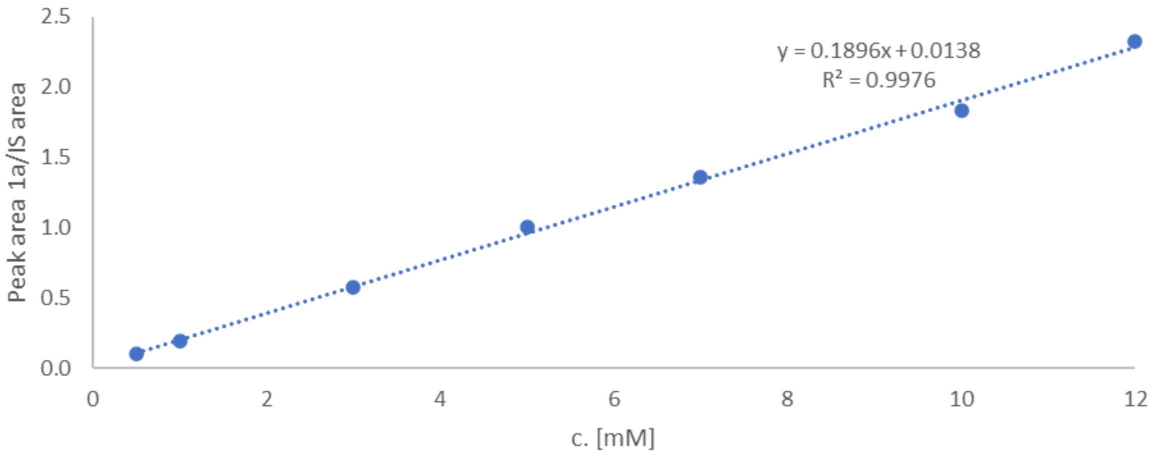

Figure S42. *Calibration curve for the quantification of lauric acid (**2a**).*

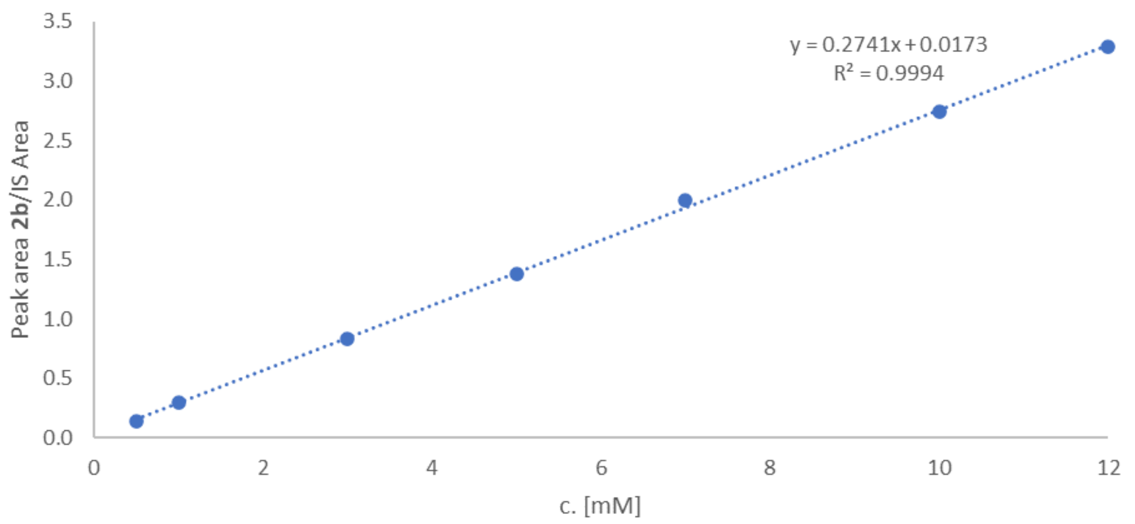

Figure S43. *Calibration curve for the quantification of tridecane (**1b**).*

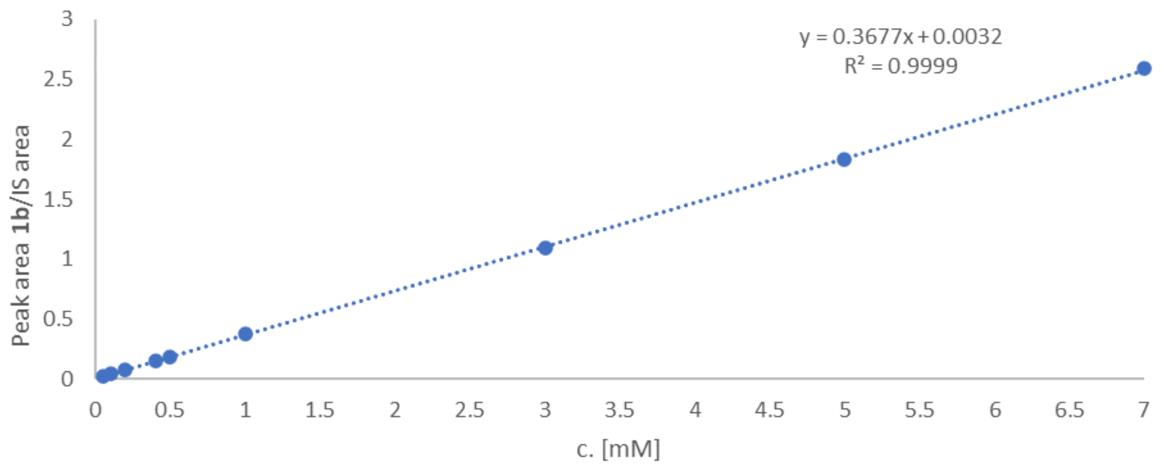

Figure S44. *Calibration curve for the quantification of undecane (**2b**).*

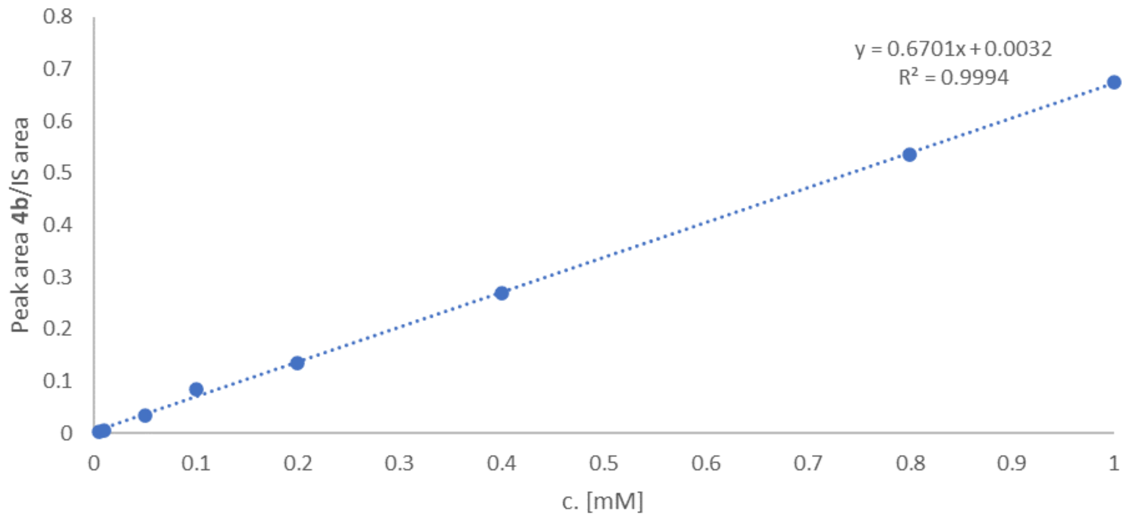

Figure S45. *Calibration curve for the quantification of 3-tridecylcyclopentan-1-one (**4a**).*

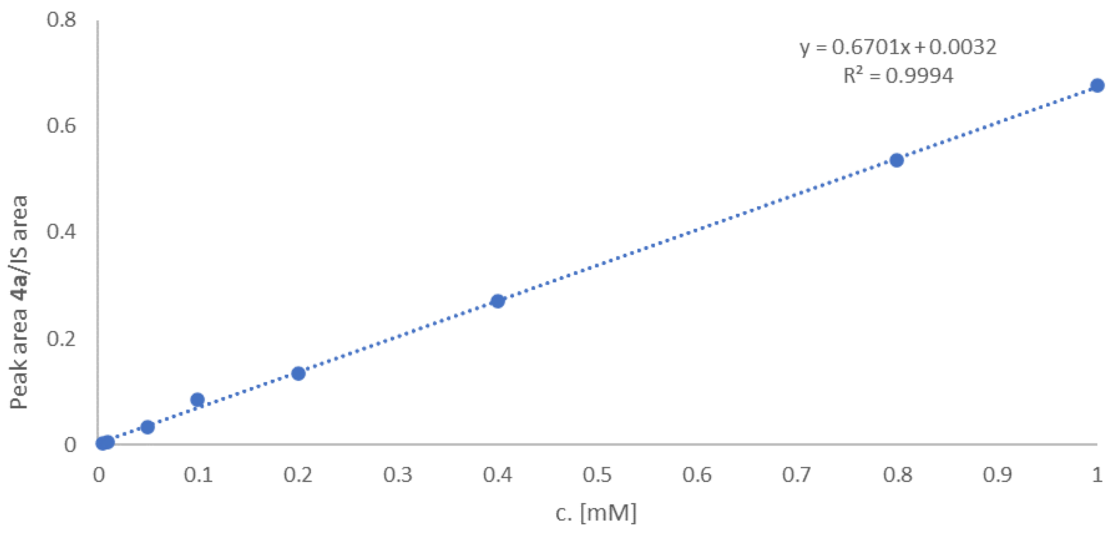

Figure S46. *Calibration curve for the quantification of 3-undecylcyclopentan-1-one (**4b**).*

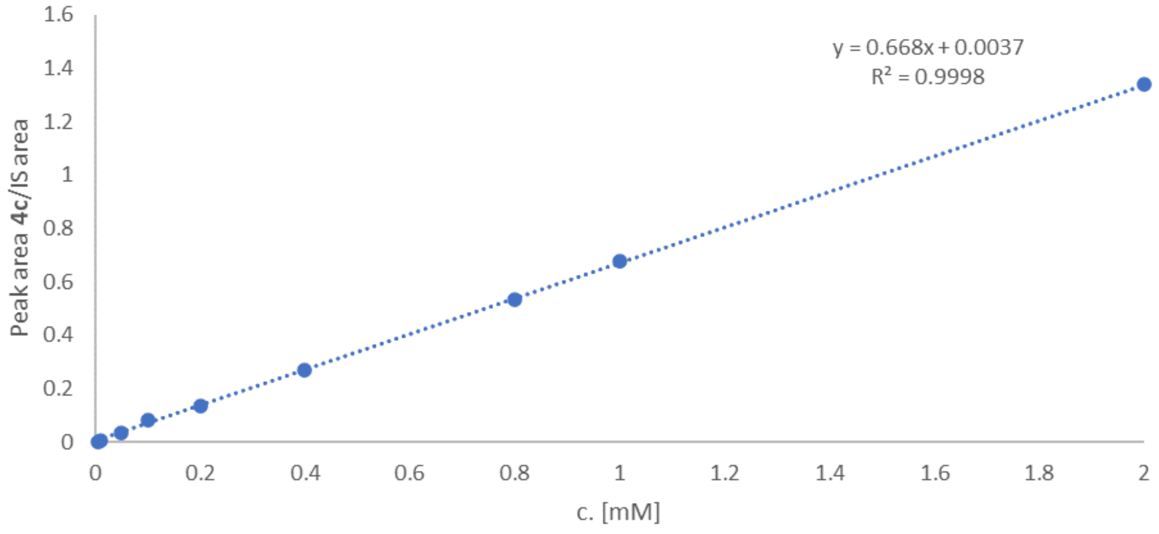

Figure S47. *Calibration curve for the quantification of 3-tridecylcyclohexan-1-one (**4c**).*

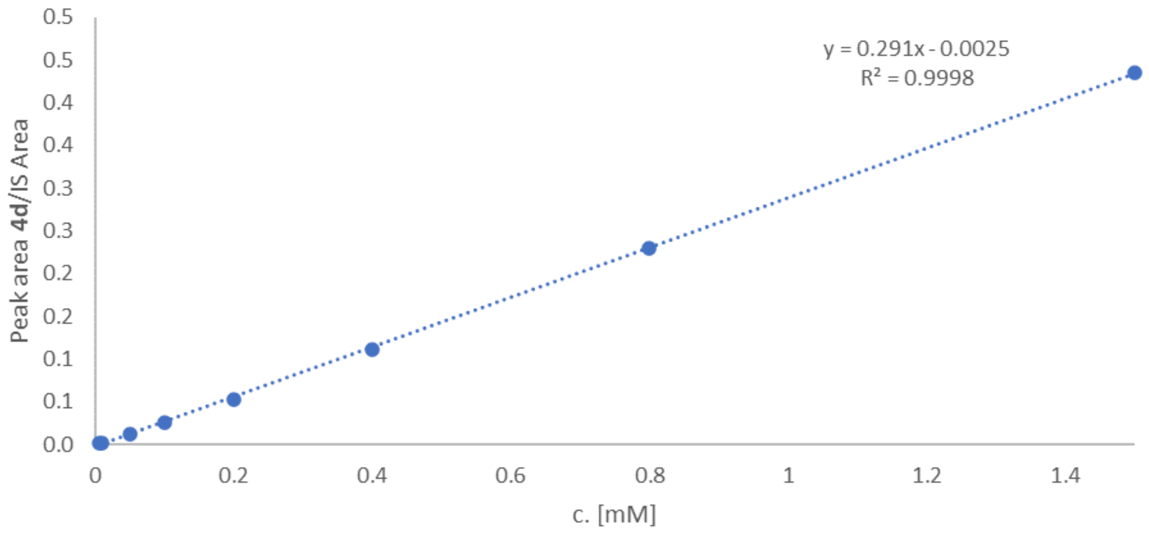

Figure S48. *Calibration curve for the quantification of rac-2-methyl-3-tridecylcyclopentan-1-one (**4d**).*

7.4.2 Decarboxylative radical cyclization with nucleophilic C=C bonds

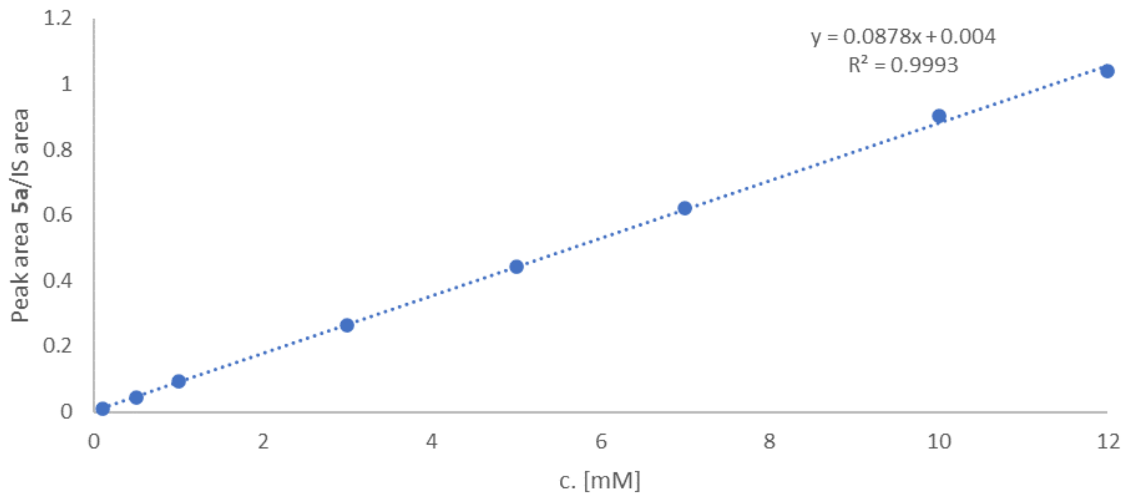

Figure S49. *Calibration curve for the quantification of hept-6-enoic acid (**5a**).*

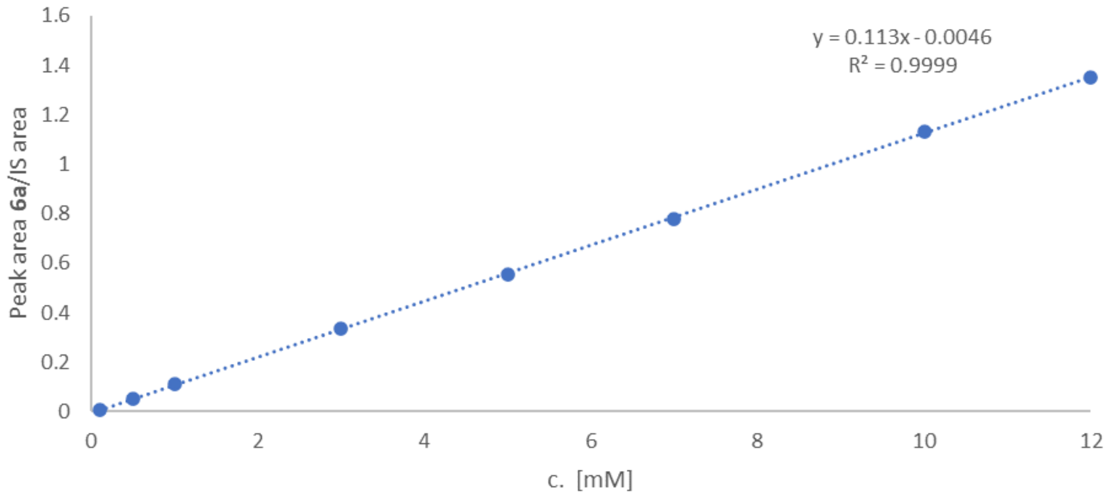

Figure S50. *Calibration curve for the quantification of oct-7-enoic acid (**6a**).*

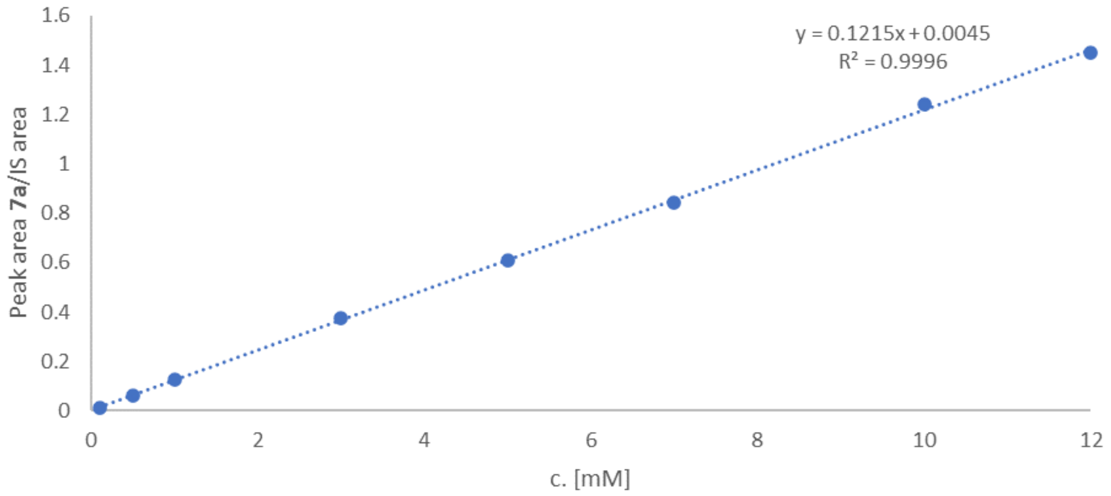

Figure S51. *Calibration curve for the quantification of non-8-enoic acid (**7a**).*

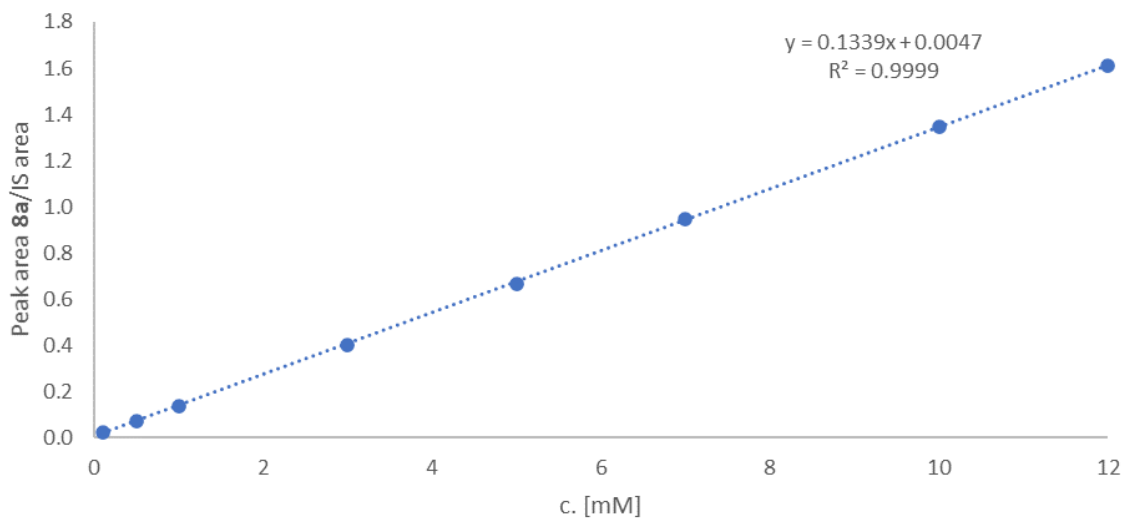

Figure S52. *Calibration curve for the quantification of (Z)-5-tetradecenoic acid ((Z)-**8a**).*

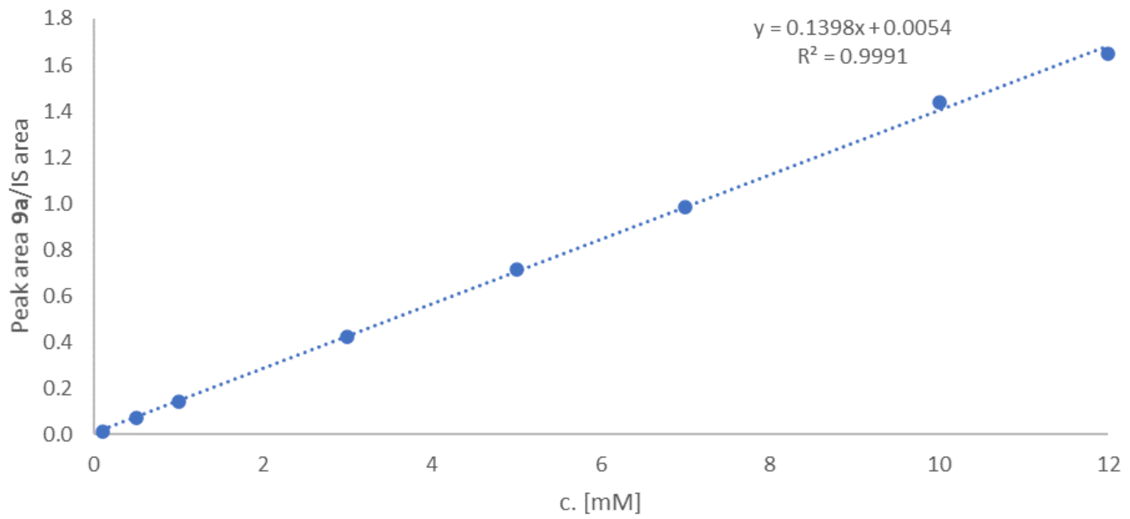

Figure S53. *Calibration curve for the quantification of (Z)-6-tetradecenoic acid ((Z)-**9a**).*

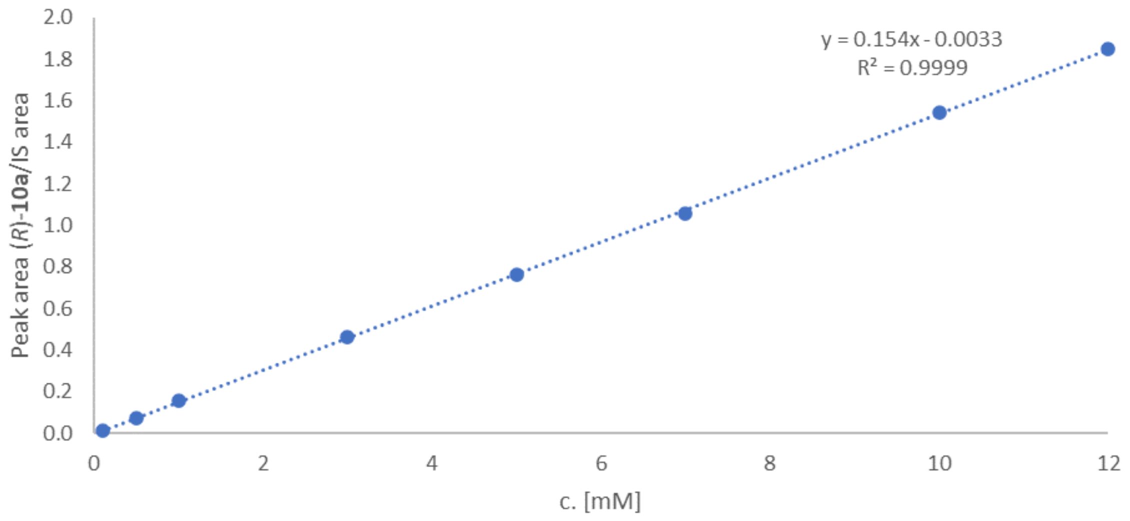

Figure S54. *Calibration curve for the quantification of (R)-citronellic acid ((R)-**10a**).*

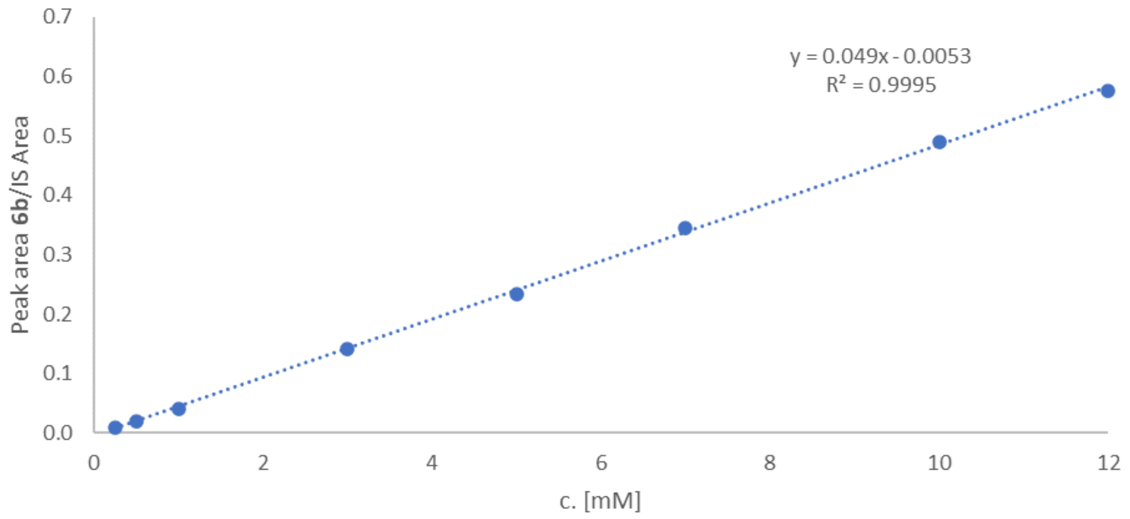

Figure S55. *Calibration curve for the quantification of hept-1-ene (**6b**).*

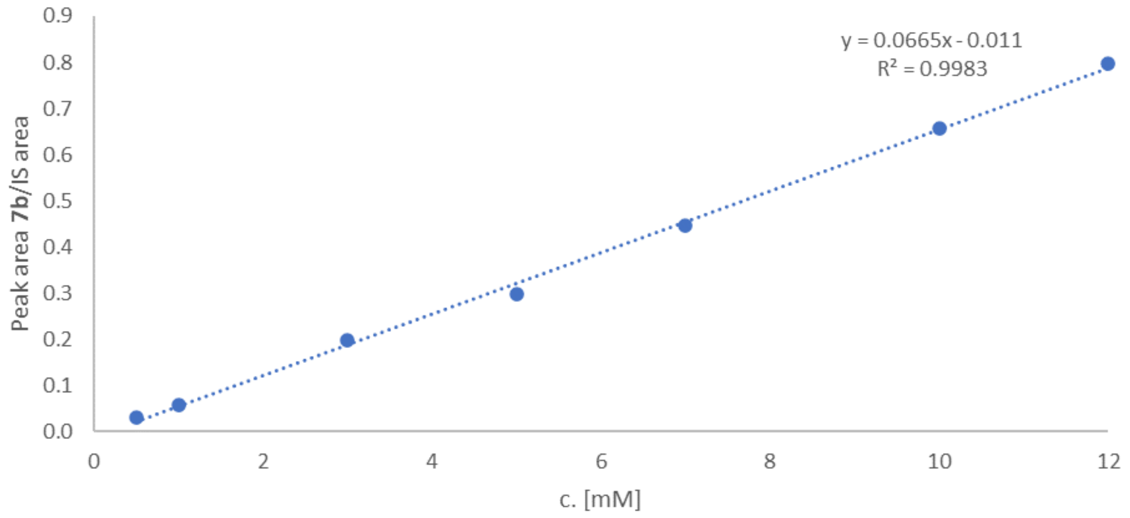

Figure S56. *Calibration curve for the quantification of oct-1-ene (**7b**).*

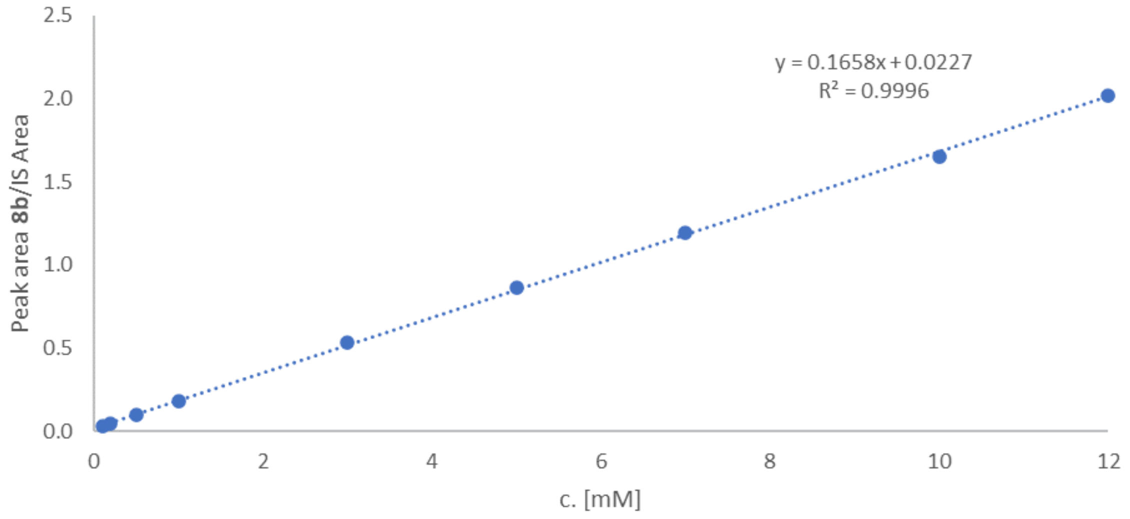

Figure S57. *Calibration curve for the quantification of (Z)-tridec-4-ene (**8b**).*

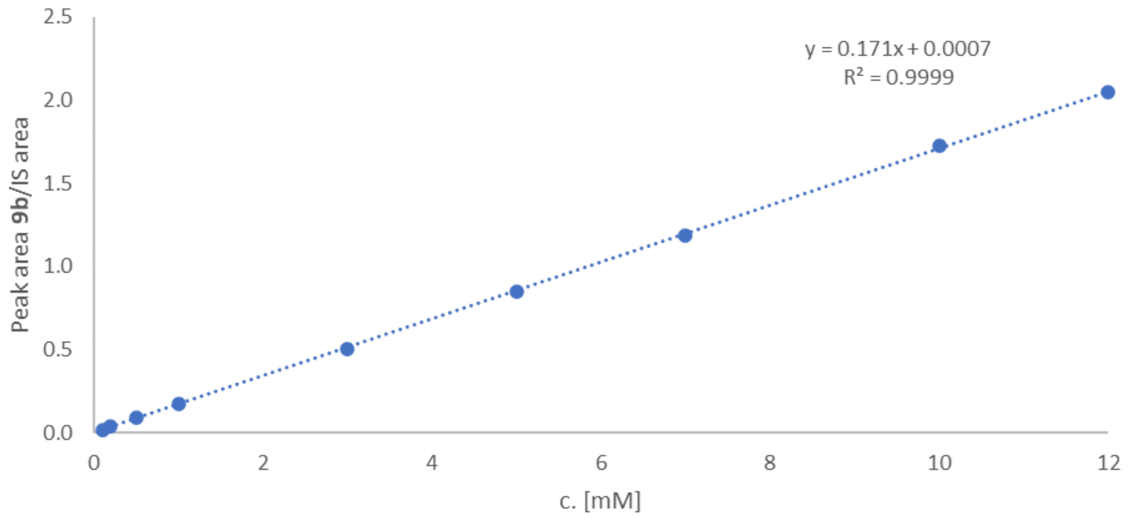

Figure S58. *Calibration curve for the quantification of (Z)-tridec-5-ene (**9b**).*

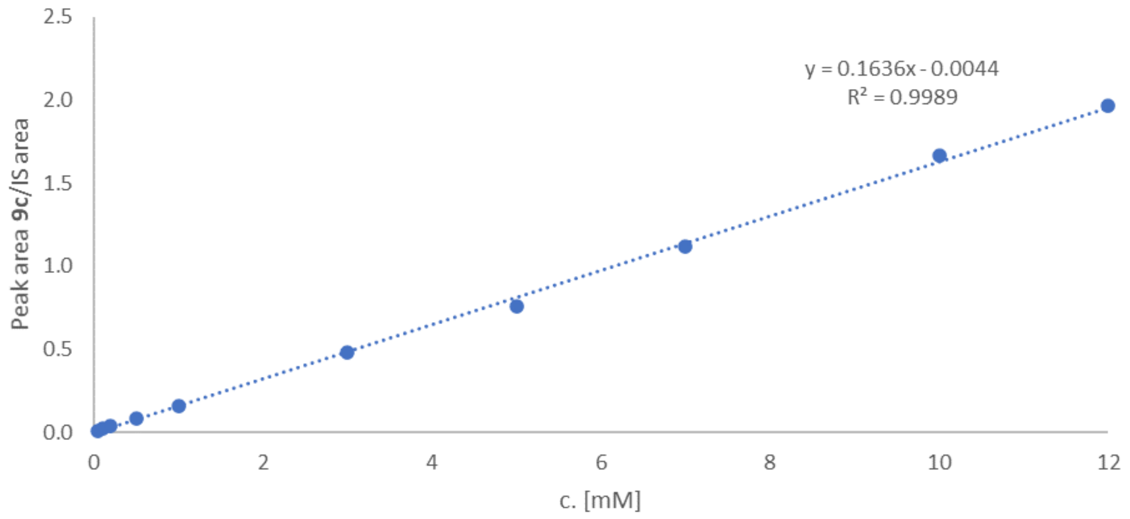

Figure S59. *Calibration curve for the quantification of octylcyclopentane (**9c**).*

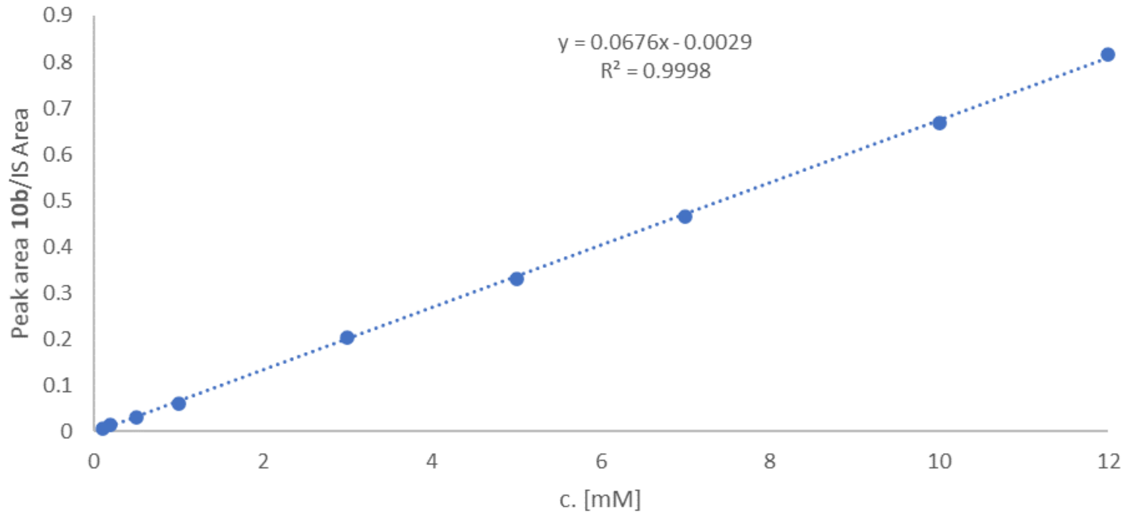

Figure S60. *Calibration curve for the quantification of 2,6-dimethylhept-2-ene (**10b**).*

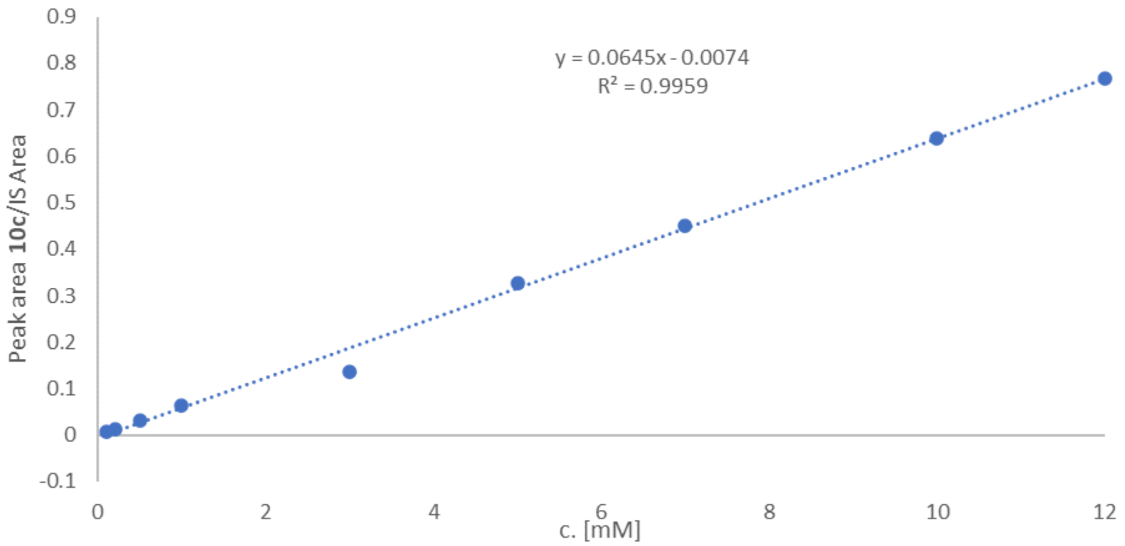

Figure S61. *Calibration curve for the quantification of 1-isopropyl-3-methylcyclopentane (**10c**).*

7.4.3 (*Z*)- to (*E*)-Photoisomerization

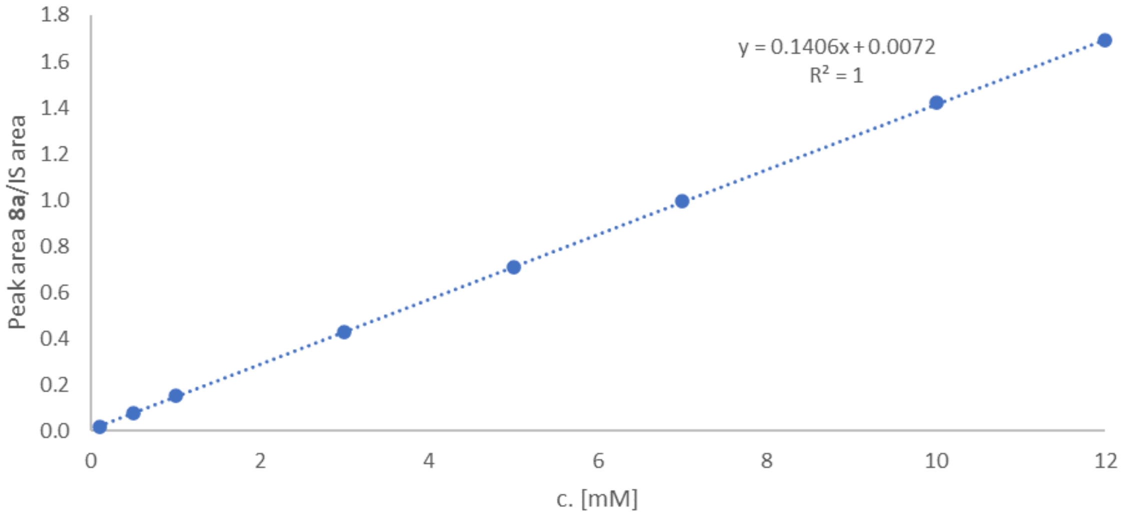

Figure S62. *Calibration curve for the quantification of (Z)-5-tetradecenoic acid ((Z)-**8a**).*

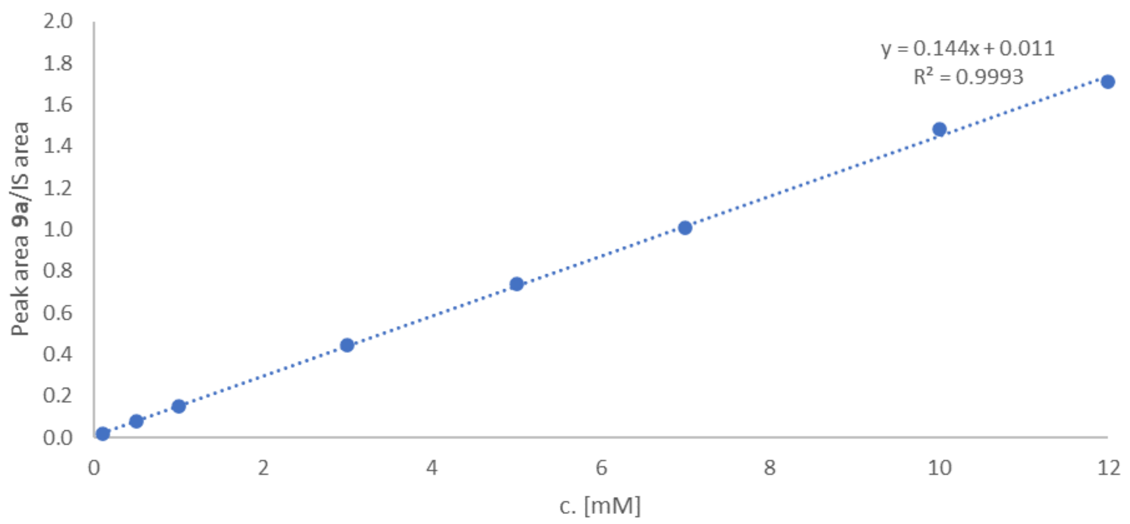

Figure S63. *Calibration curve for the quantification of (Z)-6-tetradecenoic acid ((Z)-**9a**).*

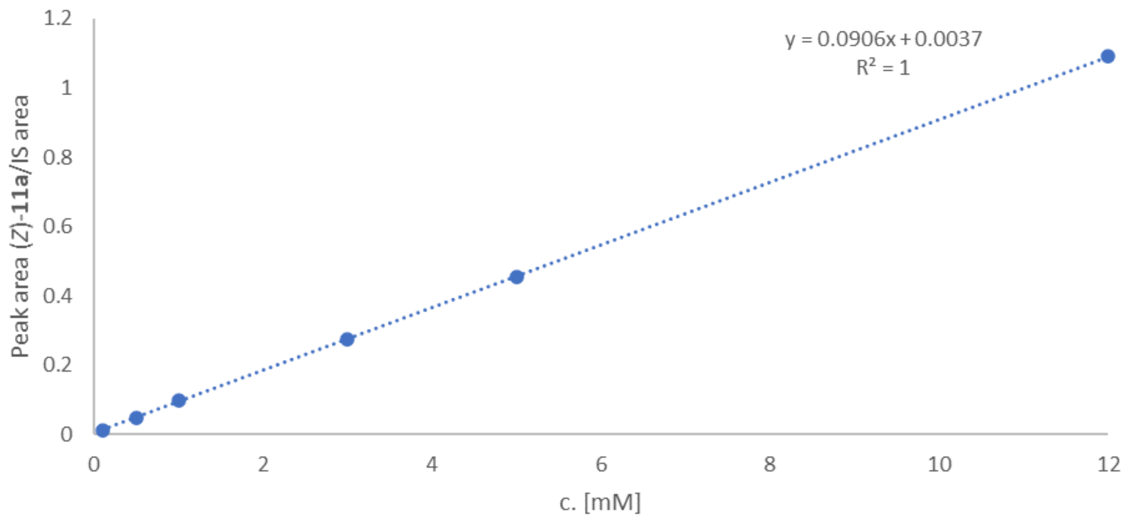

Figure S64. *Calibration curve for the quantification of (6Z)-8-methyl-6-nonenoic acid ((Z)-**11a**).*

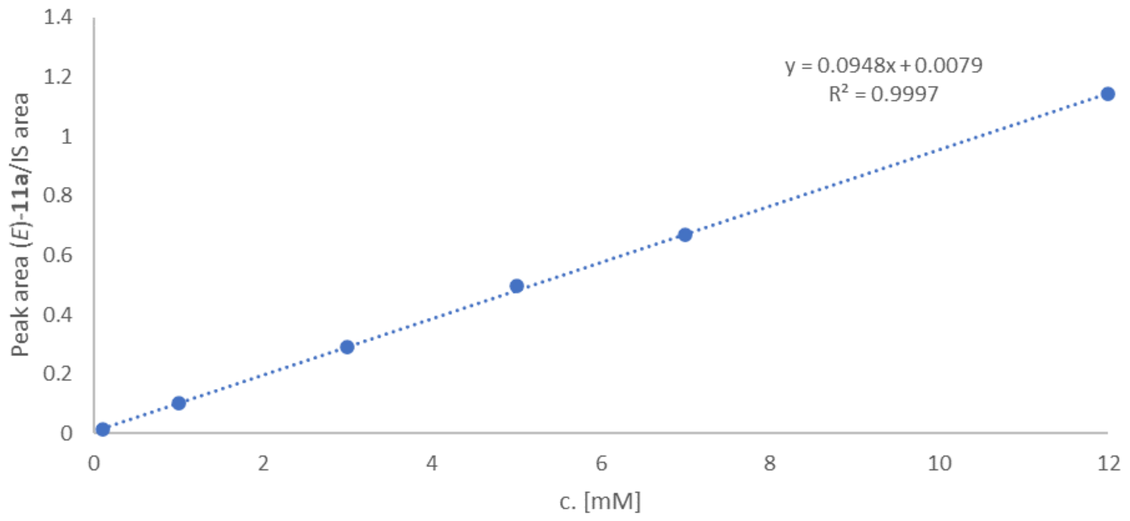

Figure S65. *Calibration curve for the quantification of (6E)-8-methyl-6-nonenoic acid ((E)-**11a**).*

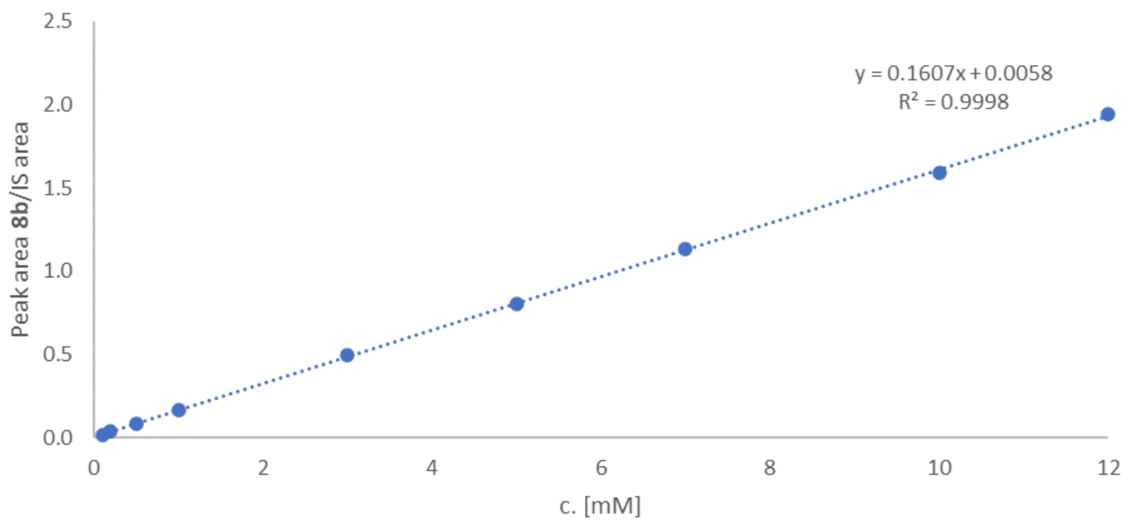

Figure S66. *Calibration curve for the quantification of (Z)-tridec-4-ene (**8b**).*

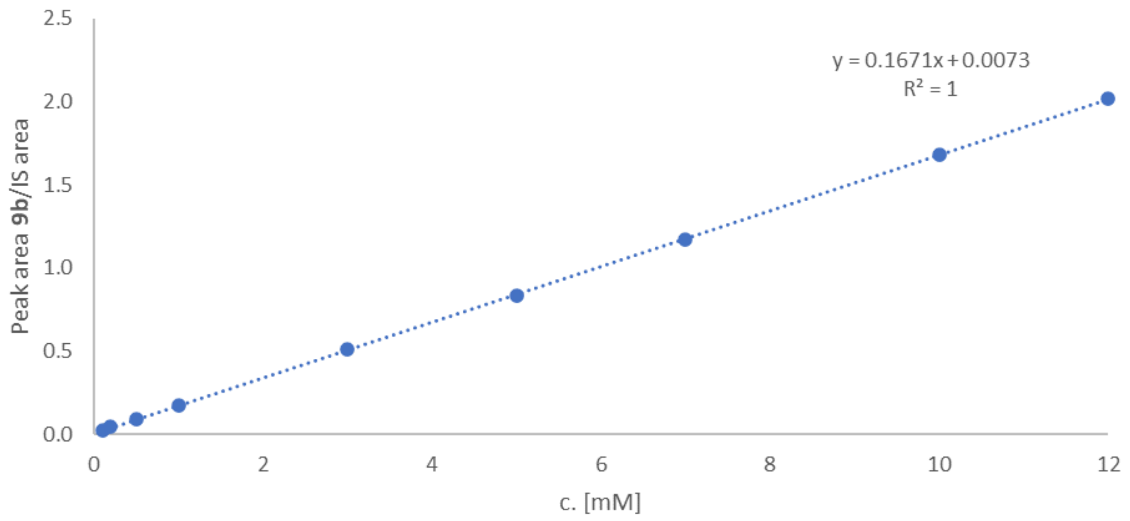

Figure S67. *Calibration curve for the quantification of (Z)-tridec-5-ene (**9b**).*

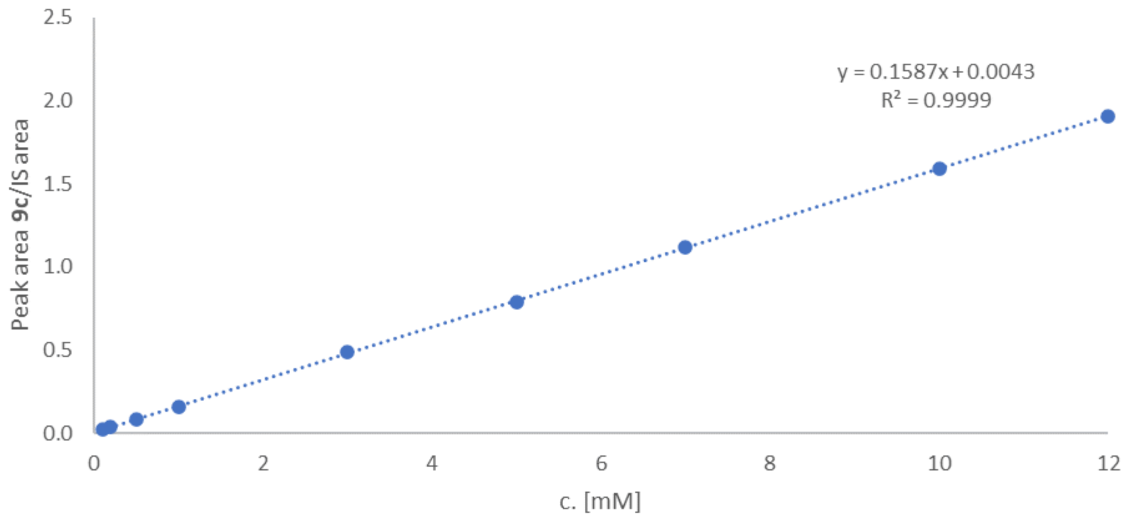

Figure S68. *Calibration curve for the quantification of octylcyclopentane (**9c**).*

7.4.4 Carbohydroxylation of C=C bonds

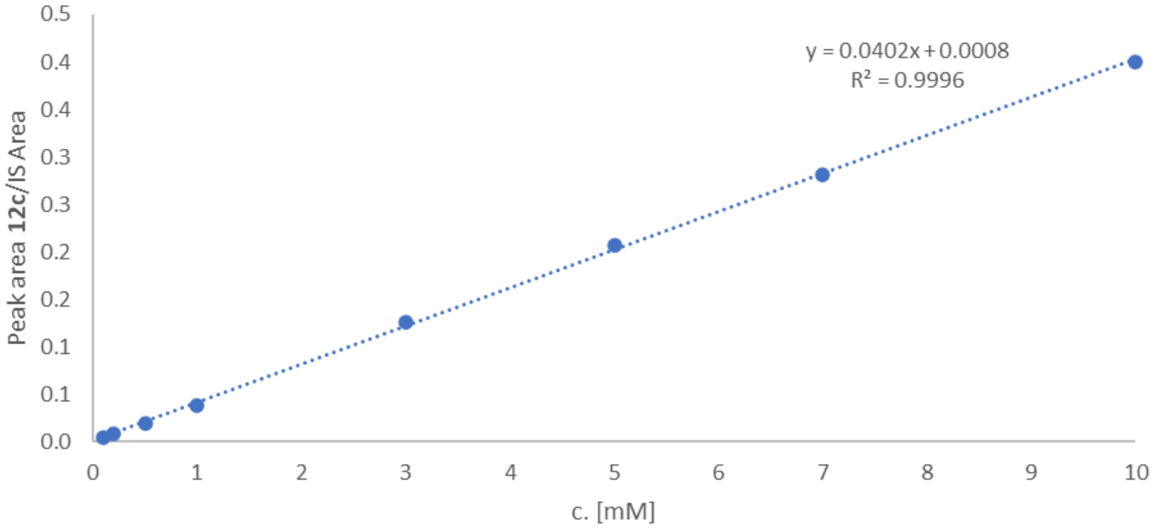

Figure S69. *Calibration curve for the quantification of cyclopentylmethylketone (**12c**).*

8 Chromatograms and Spectra

8.1 GC-FID chromatograms

8.1.1 Intermolecular decarboxylative radical coupling with electrophilic C=C bonds

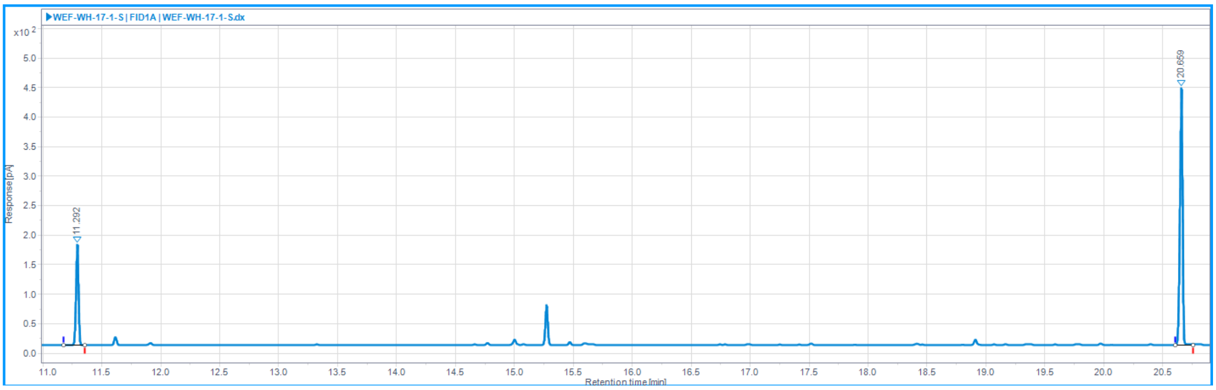

Figure S70. *GC-FID chromatogram of the biotransformation of myristic acid (**1a**, R_t = 20.66 min) without additional enone, catalyzed by CvFAP Y466A. IS = n-decane (R_t = 11.29 min). (achiral, derivatized)*

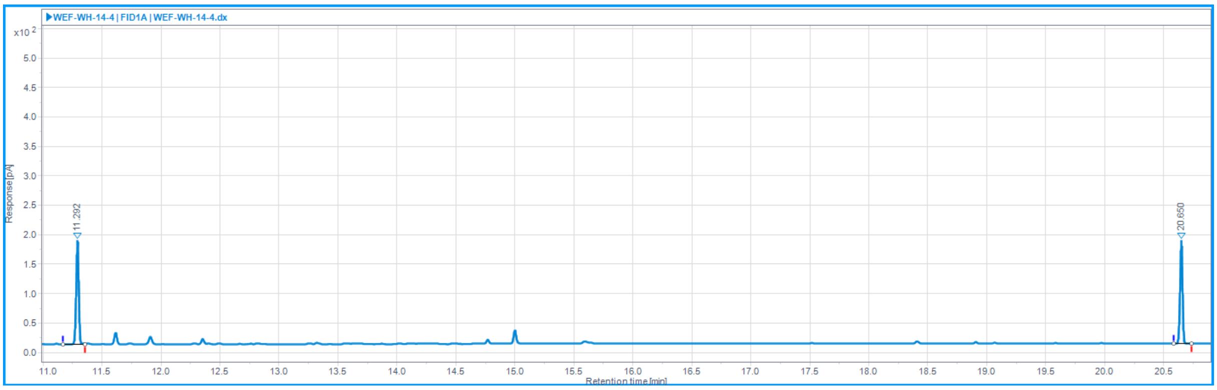

Figure S71. *GC-FID chromatogram of the substrate reference of myristic acid (**1a**, R_t = 20.65 min). IS = n-decane (R_t = 11.29 min). (achiral, derivatized)*

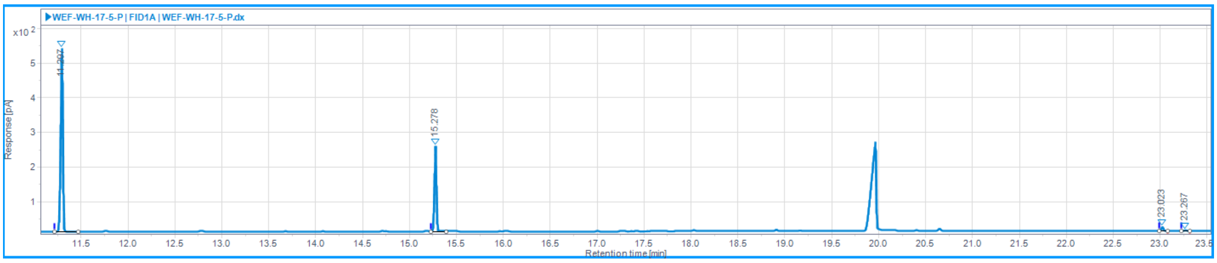

Figure S72. *GC-FID chromatogram of the biotransformation of myristic acid (**1a**) and 2-methyl-2-cyclopentenone (**3c**), catalyzed by CvFAP Y466A. Linear product (**1b,** R_t = 15.28 min). 1,4-adduct (**4d**, R_t = 23.02 min and 23.27 min) IS = n-decane (R_t = 11.29 min). (achiral, underivatized)*

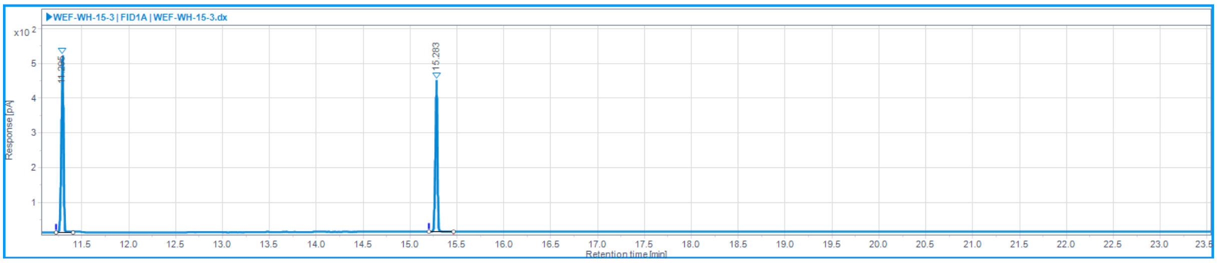

Figure S73. *GC-FID chromatogram of the product reference tridecane (**1b**, R_t = 15.28 min). IS = n-decane (R_t = 11.29 min). (achiral, underivatized)*

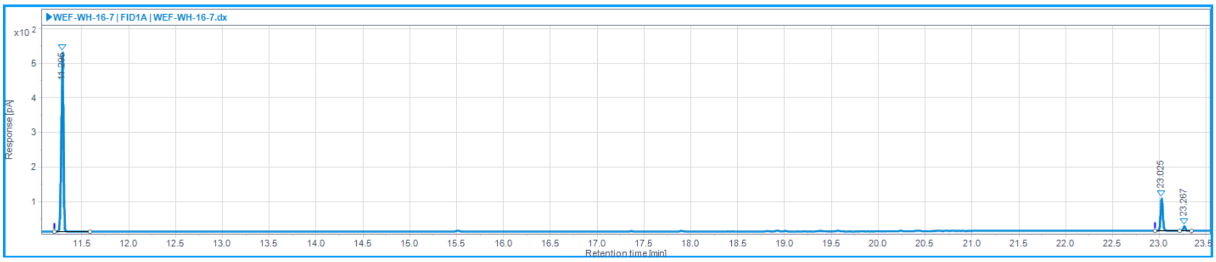

Figure S74. *GC-FID chromatogram of the product reference 2-methyl-3-tridecylcyclopentan-1-one (**4d**, R_t = 23.03 min and 23.37 min). IS = n-decane (R_t = 11.29 min). (achiral, underivatized)*

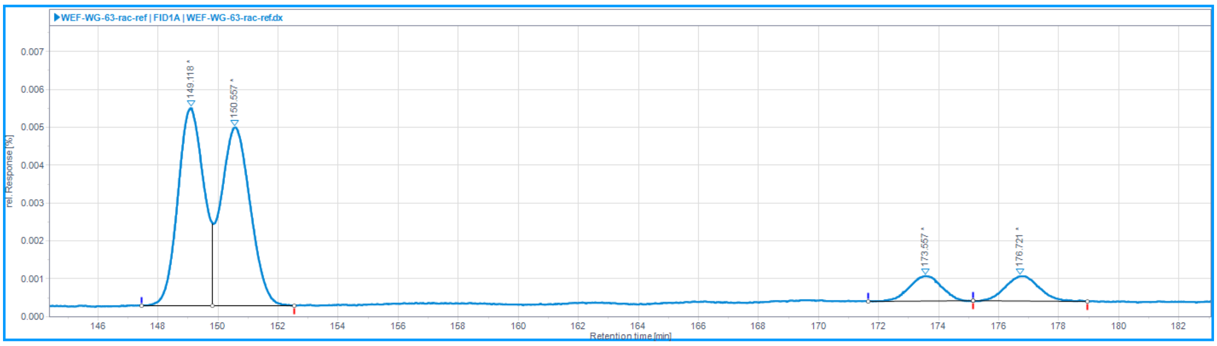

Figure S75. *GC-FID chromatogram of the chiral analysis of the racemic product reference 2-methyl-3-tridecylcyclopentan-1-one (rac-**4d**) (underivatized).*

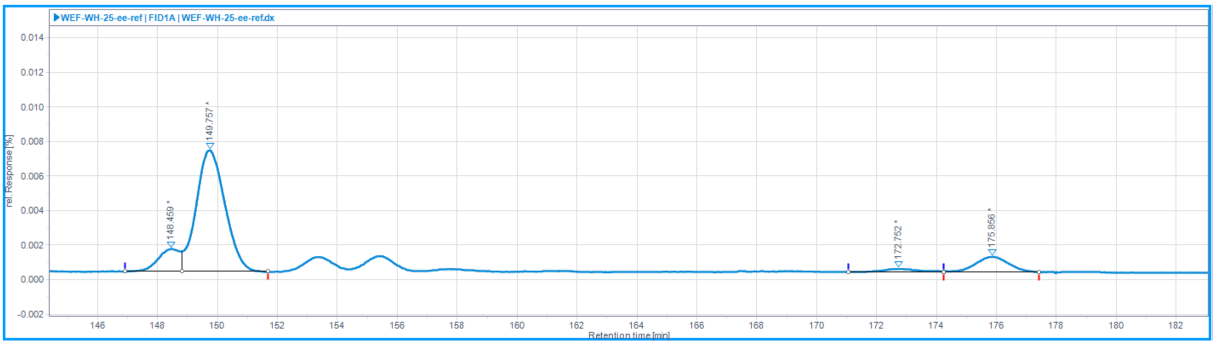

Figure S76. *GC-FID chromatogram of the chiral analysis of the enantioenriched product reference (2S/3R)-2-methyl-3-tridecylcyclopentan-1-one (2S/3R-**4d**) (underivatized).*

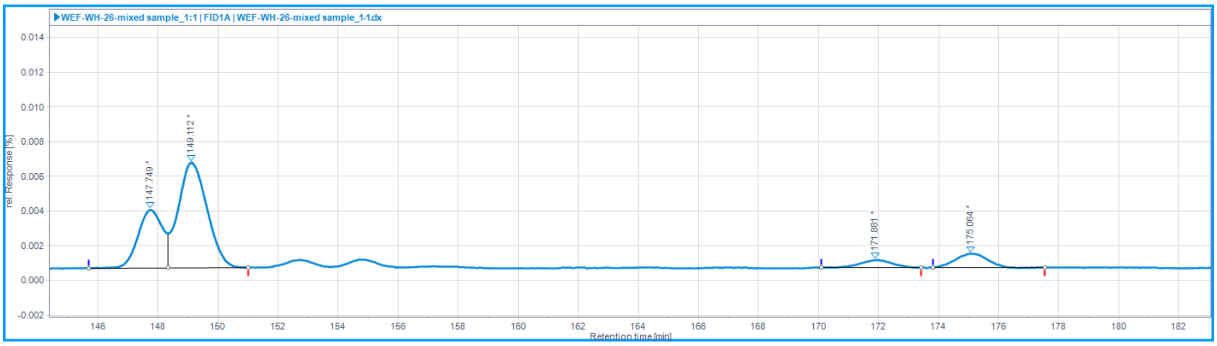

Figure S77. *GC-FID chromatogram of the chiral analysis of a 1:1 mixture spiked of rac-**4d** and (2S/3R)-**4d** (underivatized).*

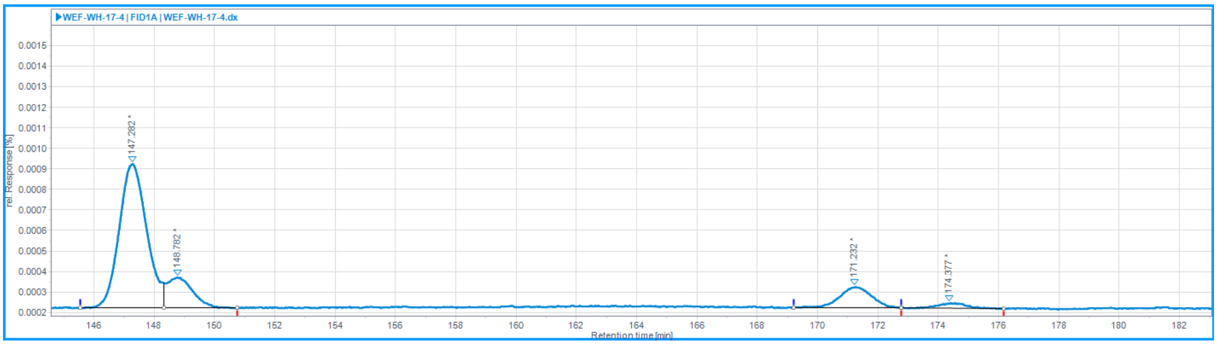

Figure S78. *GC-FID chromatogram of the chiral analysis of the biotransformation of myristic acid (**1a**) and 2-methylcyclopent-2-en-1-one (**3c**), catalyzed by CvFAP Y466A (underivatized).*

8.1.2 Decarboxylative radical cyclization with nucleophilic C=C bonds

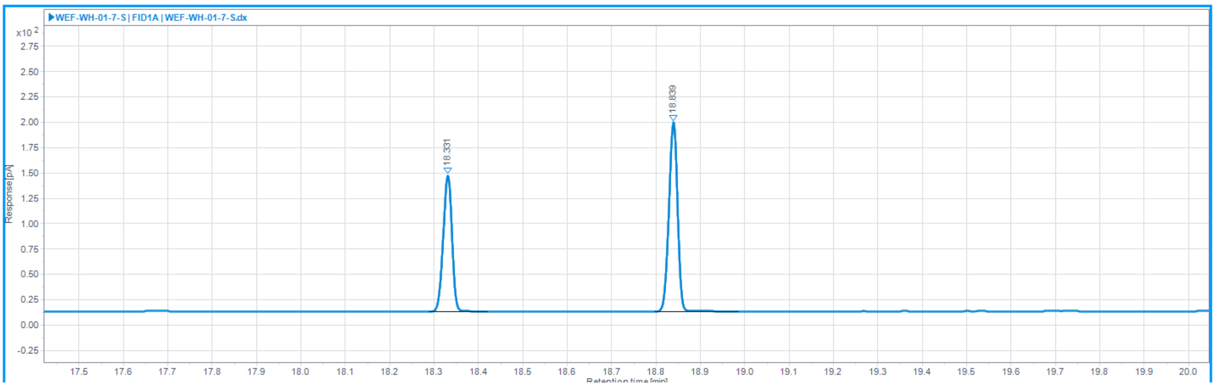

Figure S79. *GC-FID chromatogram of the biotransformation of hept-6-enoic acid (**5a**, R_t = 18.33 min), catalyzed by CvFAP Y466A. IS = n-dodecane (R_t = 18.84 min). (achiral, derivatized)*

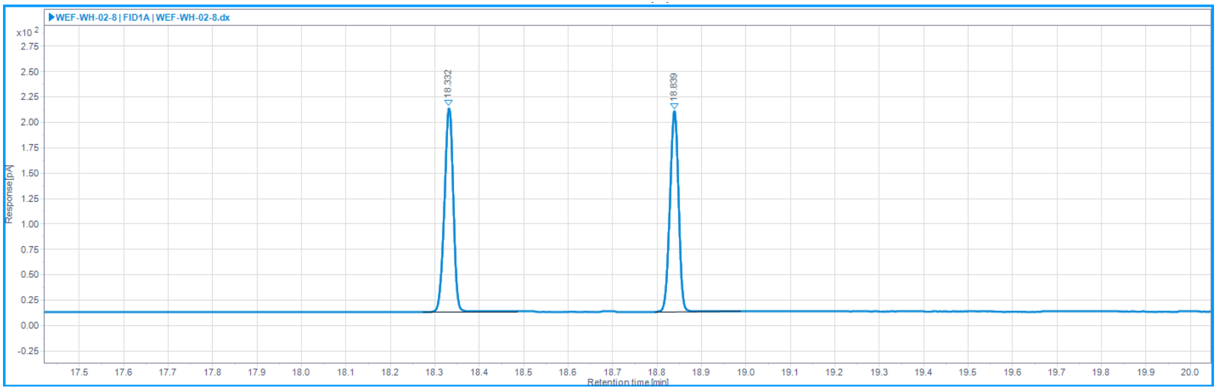

Figure S80. *GC-FID chromatogram of the substrate reference hept-6-enoic acid (**5a**, R_t = 18.33 min). IS = n-dodecane (R_t = 18.84 min). (achiral, derivatized)*

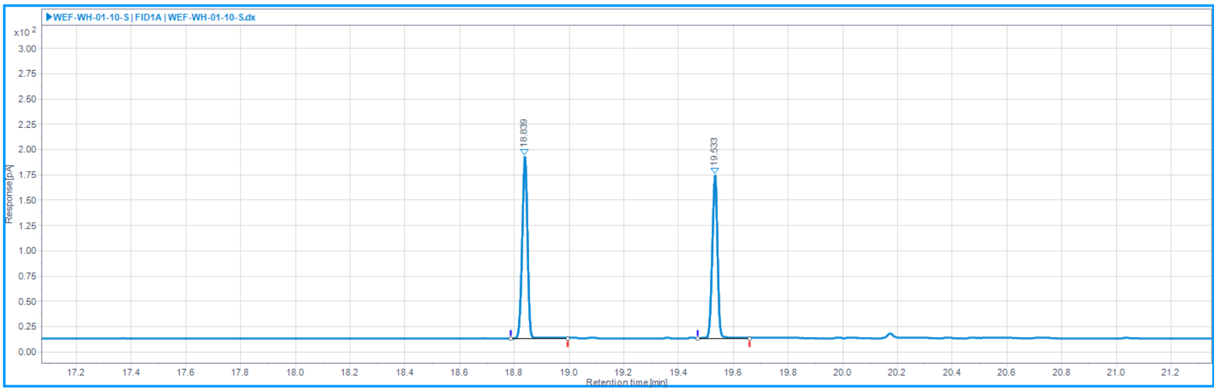

Figure S81. *GC-FID chromatogram of the biotransformation of oct-7-enoic acid (**6a,** R_t = 19.53 min), catalyzed by CvFAP Y466A. IS = n-dodecane (R_t = 18.84 min). (achiral, derivatized)*

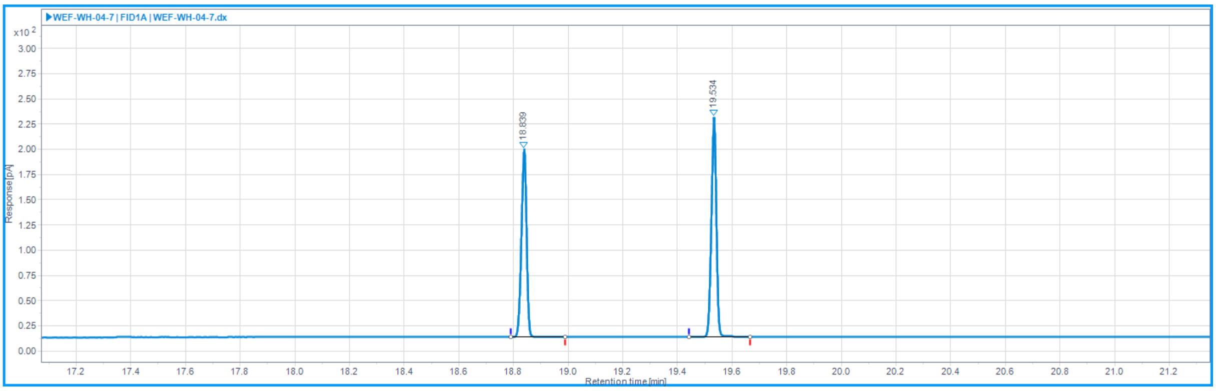

Figure S82. *GC-FID chromatogram of the substrate reference oct-7-enoic acid (**6a,** R_t = 19.53 min). IS = n-dodecane (R_t = 18.84 min). (achiral, derivatized)*

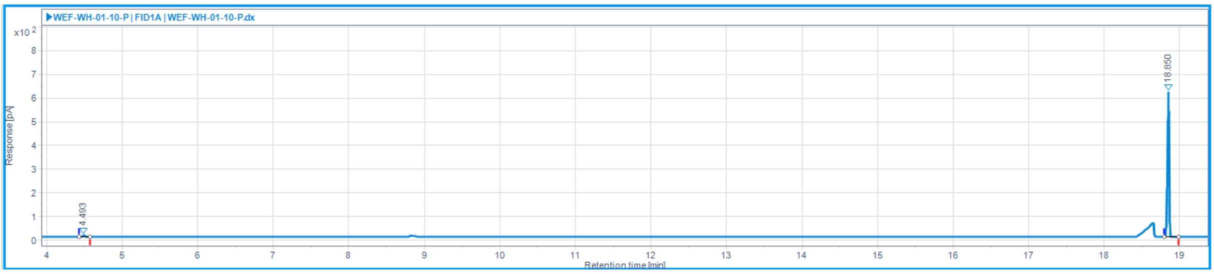

Figure S83. *GC-FID chromatogram of the biotransformation of oct-7-enoic acid (**6a**), catalyzed by CvFAP Y466A. IS = n-dodecane (R_t = 18.85 min). Linear product (**6b**, R_t = 4.49 min). (achiral, underivatized)*

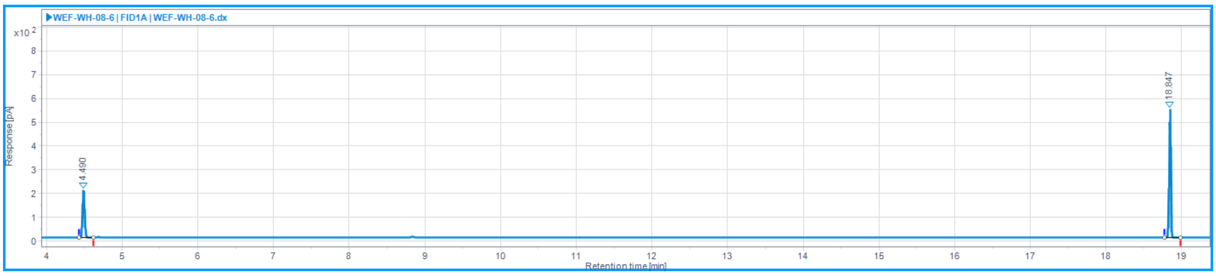

Figure S84. *GC-FID chromatogram of the product reference 1-heptene (**6b**, R_t = 4.49 min). IS = n-dodecane (R_t = 18.85 min). (achiral, underivatized)*

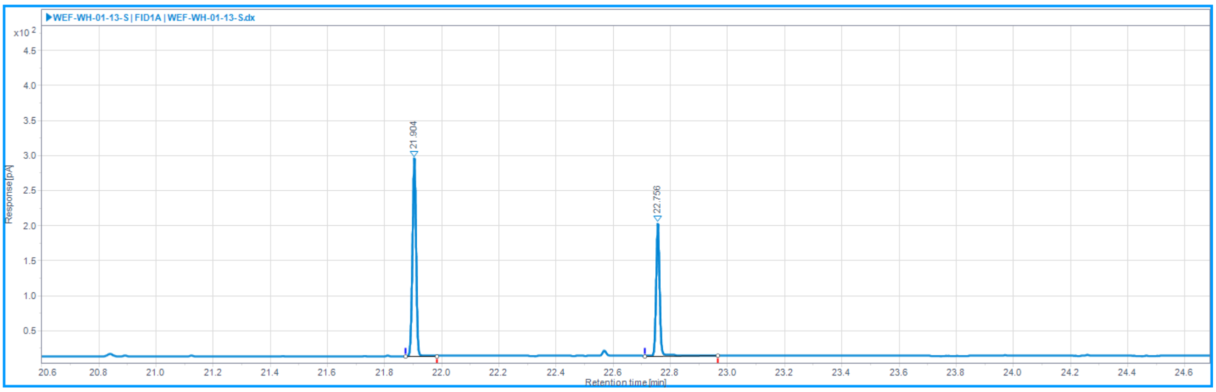

Figure S85. *GC-FID chromatogram of the biotransformation of non-8-enoic acid (**7a**, R_t = 22.76 min), catalyzed by CvFAP Y466A. IS = n-dodecane (R_t = 21.90 min). (achiral, derivatized)*

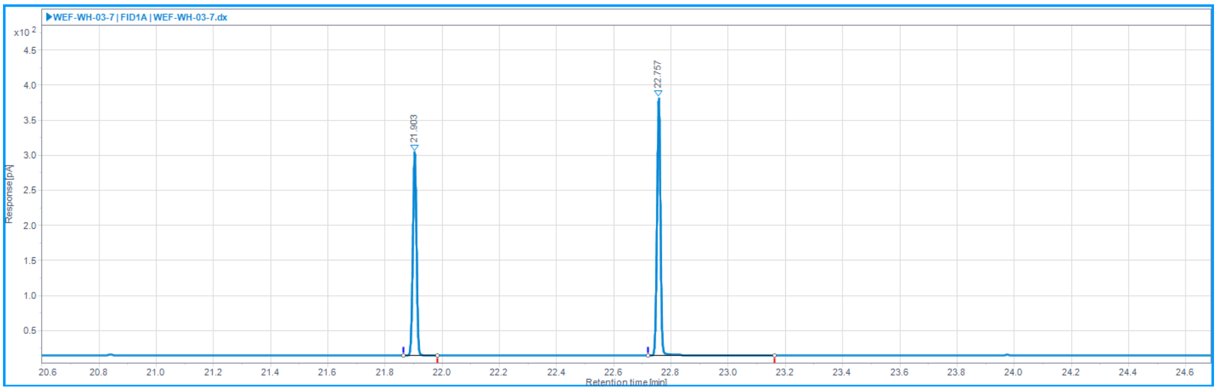

Figure S86. *GC-FID chromatogram of the substrate reference non-8-enoic acid (**7a**, R_t = 22.76 min). IS = n-dodecane (R_t = 21.90 min). (achiral, derivatized)*

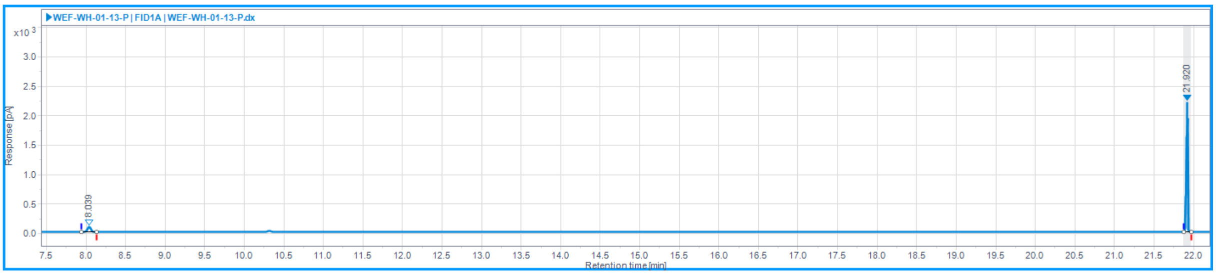

Figure S87. *GC-FID chromatogram of the biotransformation of non-8-enoic acid (**7a**), catalyzed by CvFAP Y466A. 1-octene (R_t = 8.04 min). IS = n-dodecane (R_t = 21.90 min). (achiral, underivatized)*

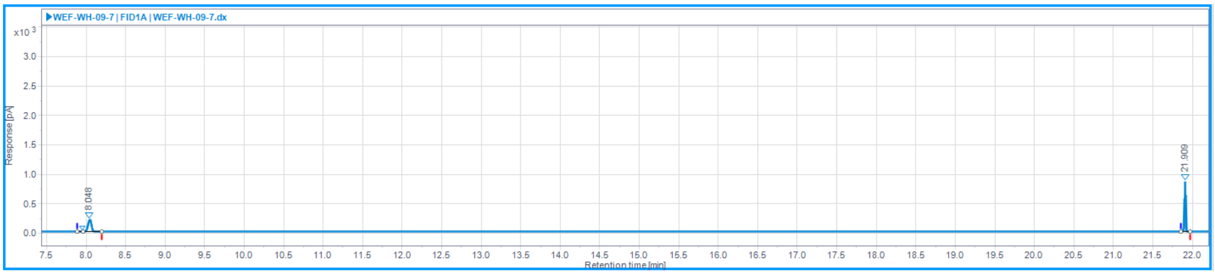

Figure S88. *GC-FID chromatogram of the product reference 1-octene (**7b**, R_t = 8.05 min). IS = n-dodecane (R_t = 21.90 min). (achiral, underivatized)*

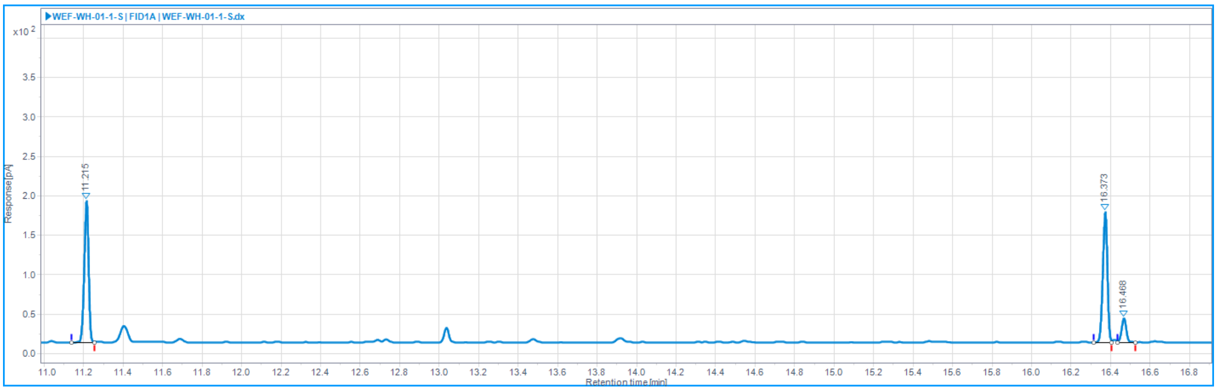

Figure S89. *GC-FID chromatogram of the biotransformation of (Z)-5-tetradecenoic acid ((Z)-**8a**, R_t = 16.37 min), catalyzed by CvFAP Y466A. E-isomer ((E)-**8a**, R_t = 16.47 min). IS = 1-decanol (R_t = 11.21 min). (achiral, derivatized)*

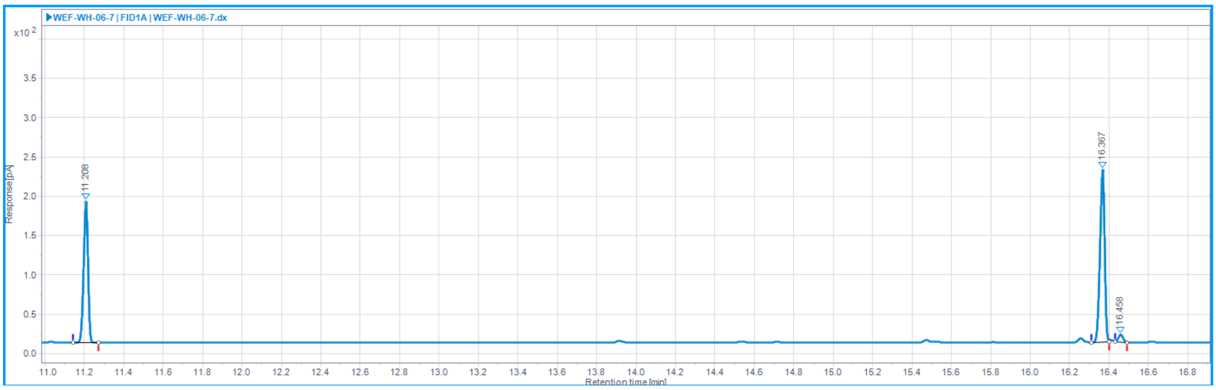

Figure S90. *GC-FID chromatogram of the substrate reference (Z)-5-tetradecenoic acid ((Z)-**8a**, R_t = 16.37 min). E-isomer ((E)-**8a**, R_t = 16.46 min). IS = 1-decanol (R_t = 11.21 min). (achiral, derivatized)*

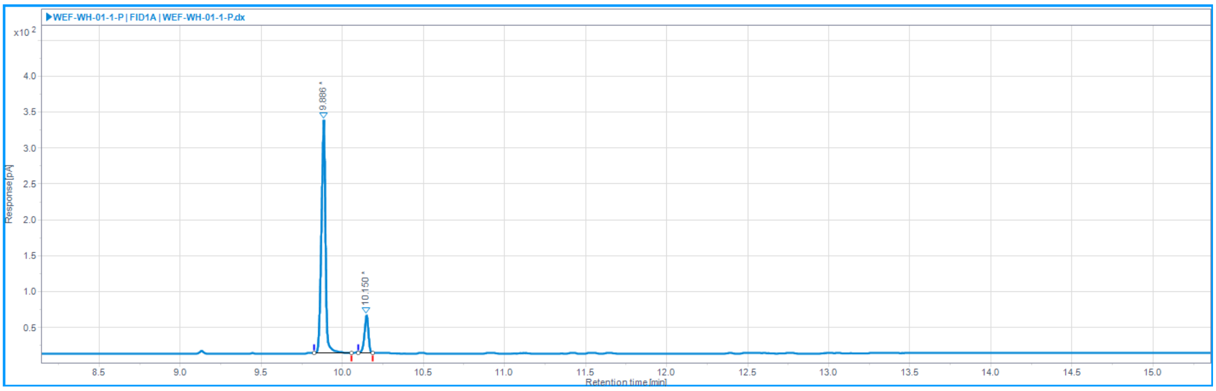

Figure S91. *GC-FID chromatogram of the biotransformation of (Z)-5-tetradecenoic acid ((Z)-**8a**), catalyzed by CvFAP Y466A. Linear product (**8b**, R_t = 10.15 min). IS = 1-decanol (R_t = 9.89 min). (achiral, underivatized)*

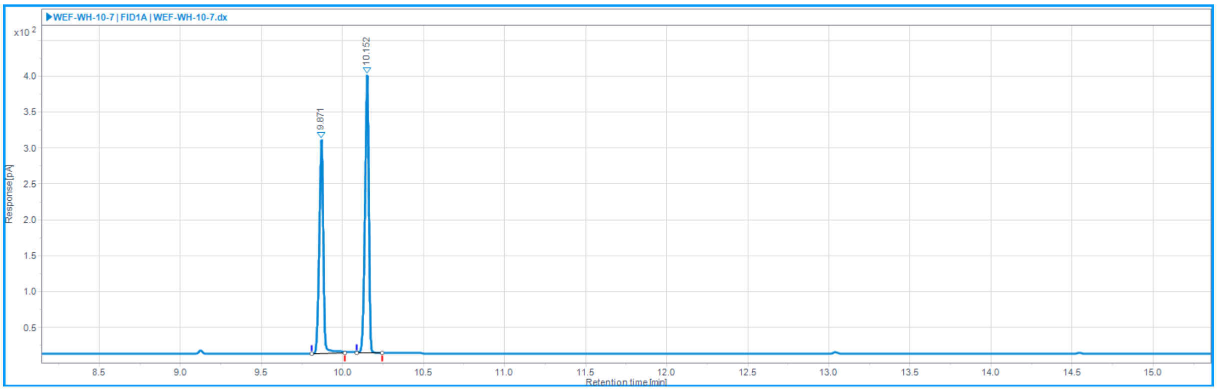

Figure S92. *GC-FID chromatogram of the product reference (Z)-4-tridecene (**8b**, R_t = 10.15 min). IS = 1-decanol (R_t = 9.87 min). (achiral, underivatized)*

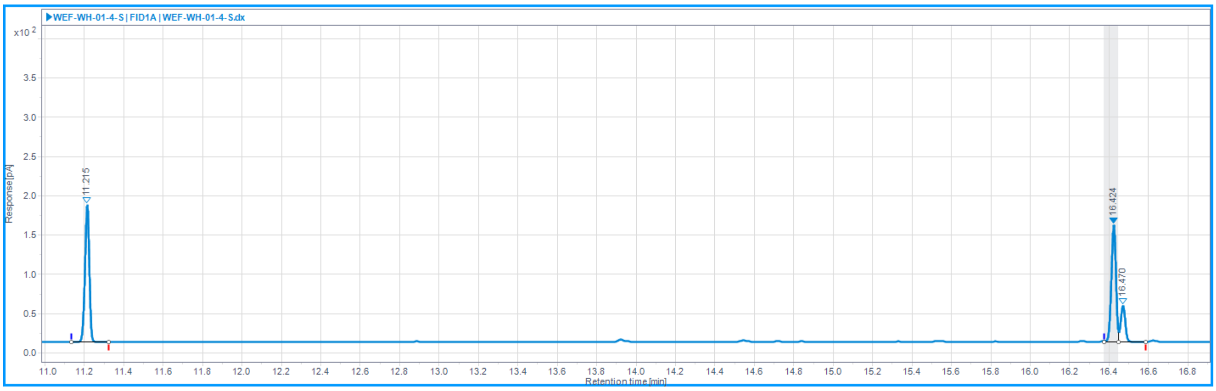

Figure S93. *GC-FID chromatogram of the biotransformation of (Z)-6-tetradecenoic acid ((Z)-**9a**, R_t = 16.42 min), catalyzed by CvFAP Y466A. E-isomer ((E)-**9a**, R_t = 16.47 min). IS = 1-decanol (R_t = 11.21 min). (achiral, derivatized)*

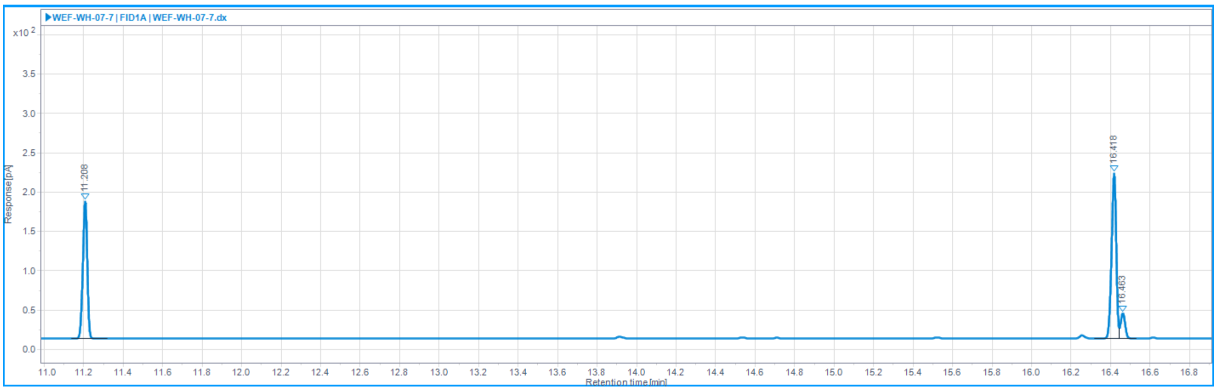

Figure S94. *GC-FID chromatogram of the substrate reference (Z)-6-tetradecenoic acid ((Z)-**9a**, R_t = 16.42 min). E-isomer ((E)-**9a**, R_t = 16.46 min). IS = 1-decanol (R_t = 11.21 min). (achiral, derivatized)*

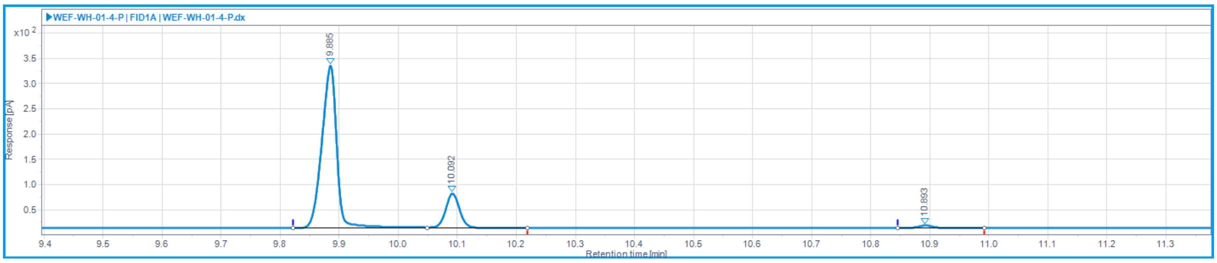

Figure S95. *GC-FID chromatogram of the biotransformation of (Z)-6-tetradecenoic acid ((Z)-**9a**), catalyzed by CvFAP Y466A. Linear product (**9b**, R_t = 10.09 min). Cyclic product (**9c**, R_t = 10.89 min). IS = 1-decanol (R_t = 9.89 min). (achiral, underivatized)*

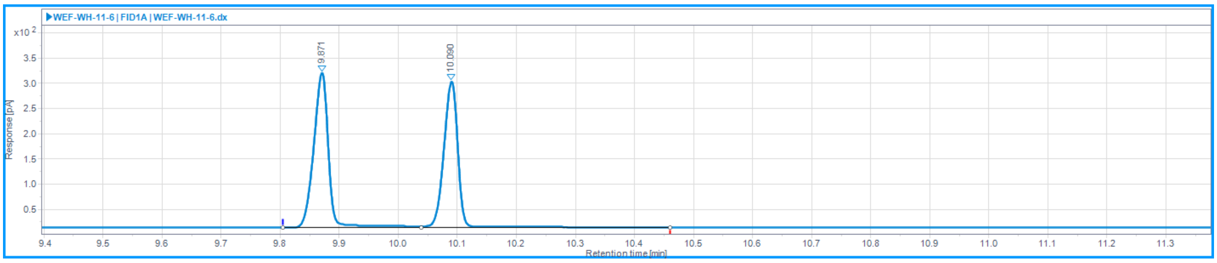

Figure S96. *GC-FID chromatogram of the product reference (Z)-5-tridecene (**9b**, R_t = 10.09 min). IS = 1-decanol (R_t = 9.87 min). (achiral, underivatized)*

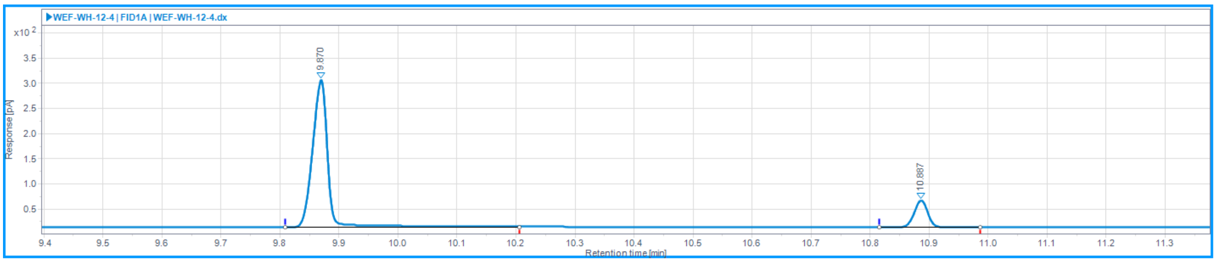

Figure S97. *GC-FID chromatogram of the product reference octylcyclopentane (**9c**, R_t = 10.89 min). IS = 1-decanol (R_t = 9.89 min). (achiral, underivatized)*

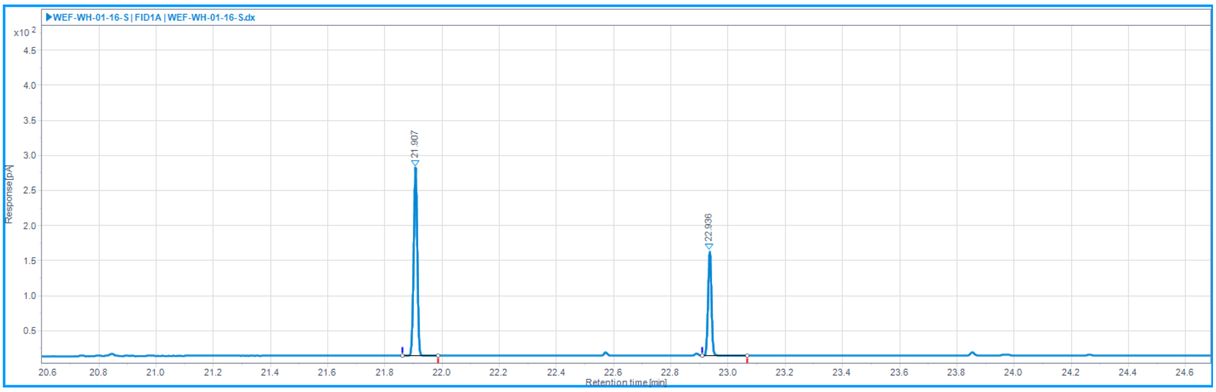

Figure S98. *GC-FID chromatogram of the biotransformation of rac-citronellic (rac-**10a,** R_t = 22.94 min), catalyzed by CvFAP Y466A. IS = n-dodecane (R_t = 21.90 min). (achiral, derivatized)*

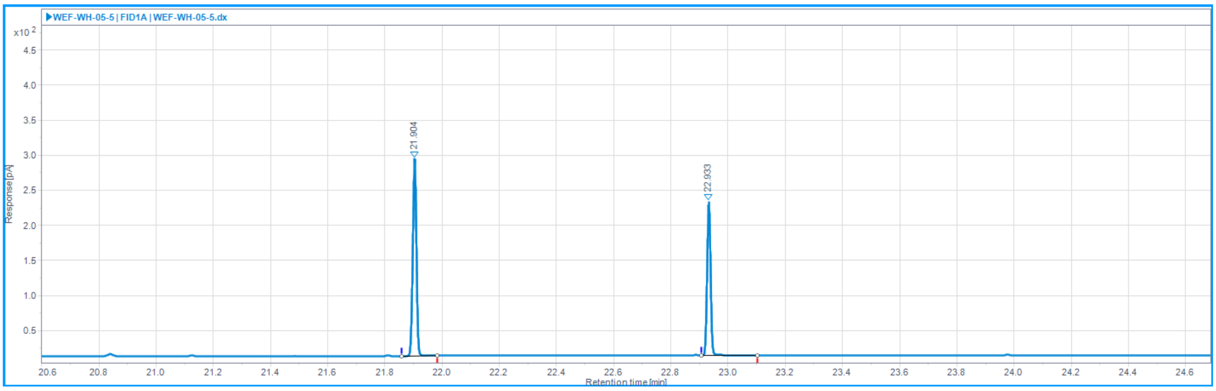

Figure S99. *GC-FID chromatogram of the substrate reference (R)-citronellic acid ((R)-**10a**, R_t = 22.93 min). IS = n-dodecane (R_t = 21.90 min). (achiral, derivatized)*

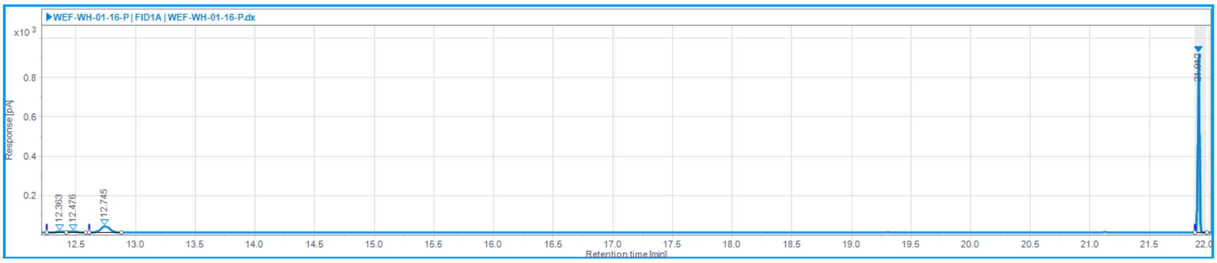

Figure S100. *GC-FID chromatogram of the biotransformation of rac-citronellic acid (rac-**10a**), catalyzed by CvFAP Y466A. IS = n-dodecane (R_t = 21.91 min). Linear product (**10b**, R_t = 12.75 min). Cyclic product (**10c**, R_t = 12.36 min and R_t = 12.48 min). (achiral, underivatized)*

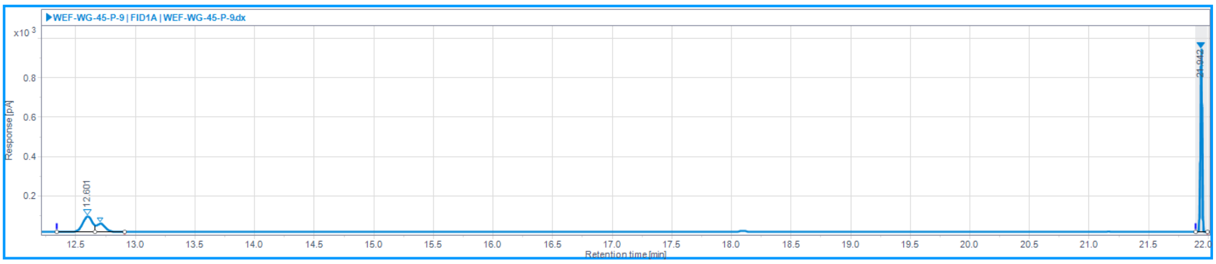

Figure S101. *GC-FID chromatogram of the product reference 1-isopropyl-3-methylcyclopentane (**10c,** R_t = 12.60 min and R_t = 12.70 min). IS = n-dodecane (R_t = 21.94 min). (achiral, underivatized)*

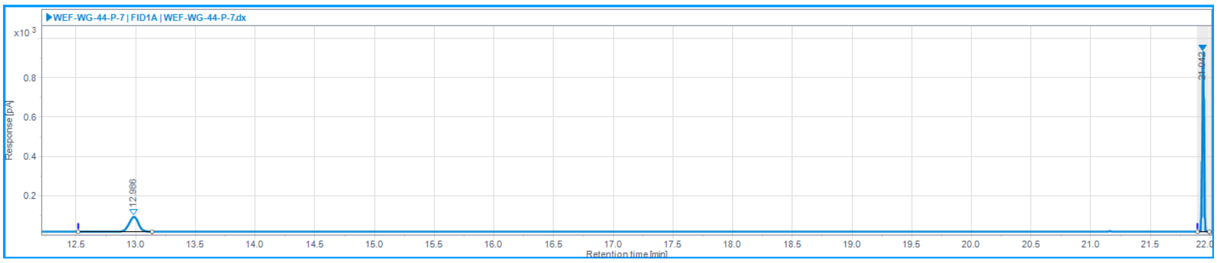

Figure S102. *GC-FID chromatogram of the product reference 2,6-dimethylheptene (**10b**, R_t = 12.99 min). IS = n-dodecane (R_t = 21.94 min). (achiral, underivatized)*

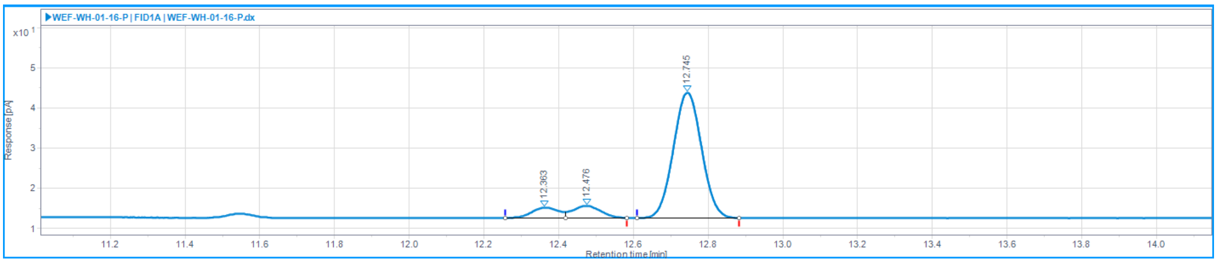

Figure S103. *GC-FID chromatogram of the biotransformation of rac-citronellic acid (rac-**10a**), catalyzed by CvFAP Y466A. Linear product (**10b**, R_t = 12.75 min). Cyclic product (**10c**, R_t = 12.36 min and R_t = 12.48 min). (Close up, achiral, underivatized)*

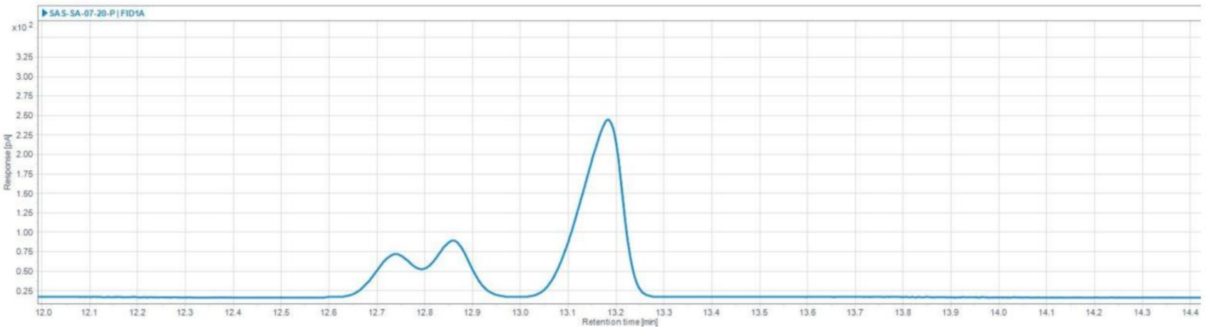

Figure S104: *GC-FID chromatogram of the biotransformation of (R)-citronellic acid ((R)-**10a**), catalyzed by CvFAP Y466A/V453S in form of resuspended whole cells (OD_{600}/mL = 40, corresponding to a CDW of 20.0 ± 0.2 mg). Linear product (**10b**, R_t = 13.2 min). Cyclic product (**10c**, R_t = 12.75 min and R_t = 12.85 min). (Close up, achiral, underivatized)*

8.1.3 (*Z*)→(*E*) Photoisomerisation of isolated C=C bonds

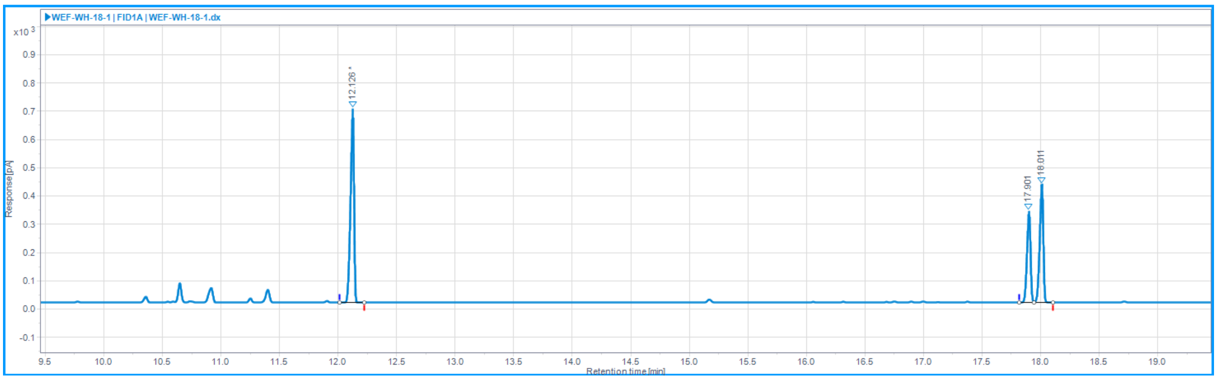

Figure S105. *GC-FID chromatogram of the biotransformation of (Z)-5-tetradecenoic acid ((Z)-**8a**, R_t = 17.90 min), catalyzed by CvFAP Y466A. E-isomer ((E)-**8a**, R_t = 18.01 min). IS = 1-decanol (R_t = 12.13 min). (achiral, derivatized)*

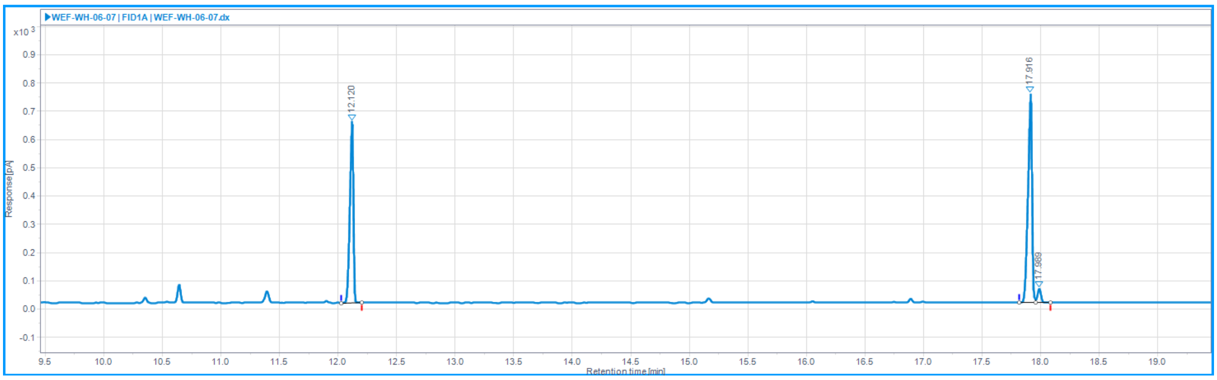

Figure S106. *GC-FID chromatogram of the substrate reference (Z)-5-tetradecenoic acid ((Z)-**8a**, R_t = 17.91 min). E-isomer ((E)-**8a**, R_t = 17.99 min). IS = 1-decanol (R_t = 12.12 min). (achiral, derivatized)*

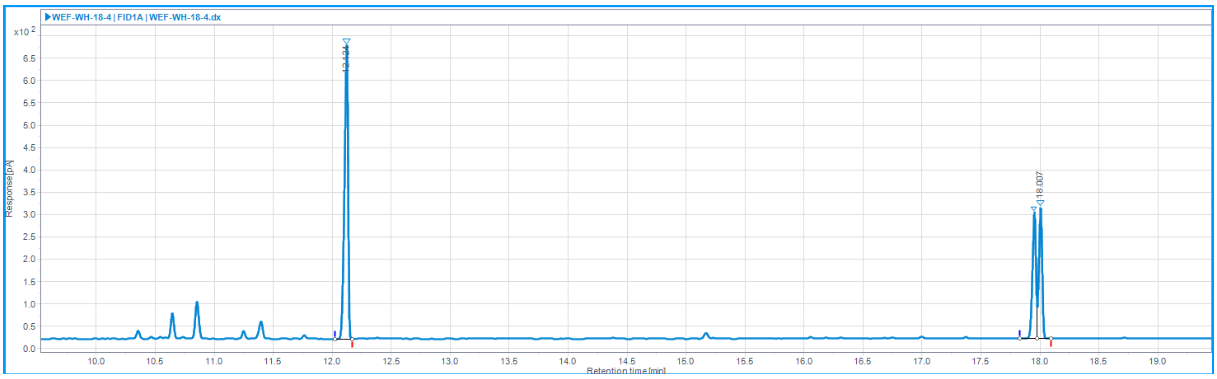

Figure S107. *GC-FID chromatogram of the biotransformation of (Z)-6-tetradecenoic acid ((Z)-**9a,** R_t = 17.92 min), catalyzed by CvFAP Y466A. E-isomer ((E)-**9a**, R_t = 18.00 min). IS = 1-decanol (R_t = 12.12 min). (achiral, derivatized)*

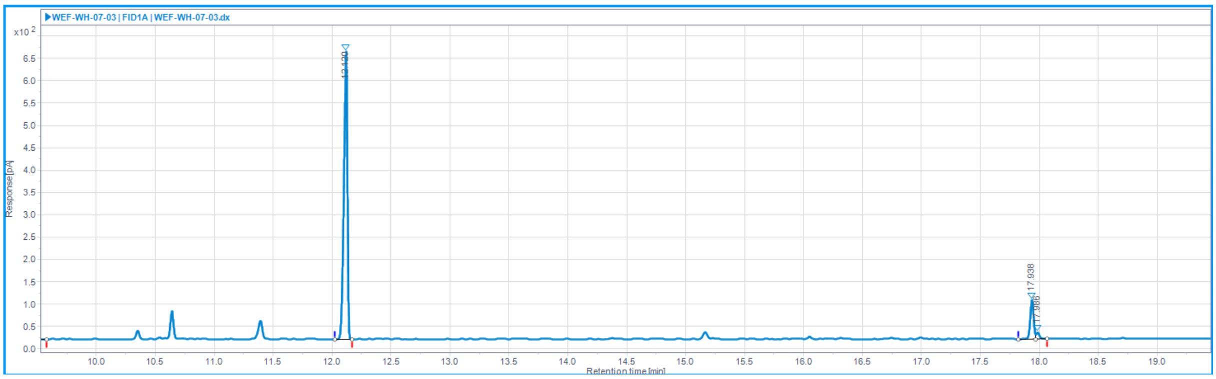

Figure S108. *GC-FID chromatogram of the substrate reference (Z)-6-tetradecenoic acid ((Z)-**9a**, R_t = 17.94 min). E-isomer ((E)-**9a**, R_t = 17.99 min). IS = 1-decanol (R_t = 12.12 min). (achiral, derivatized)*

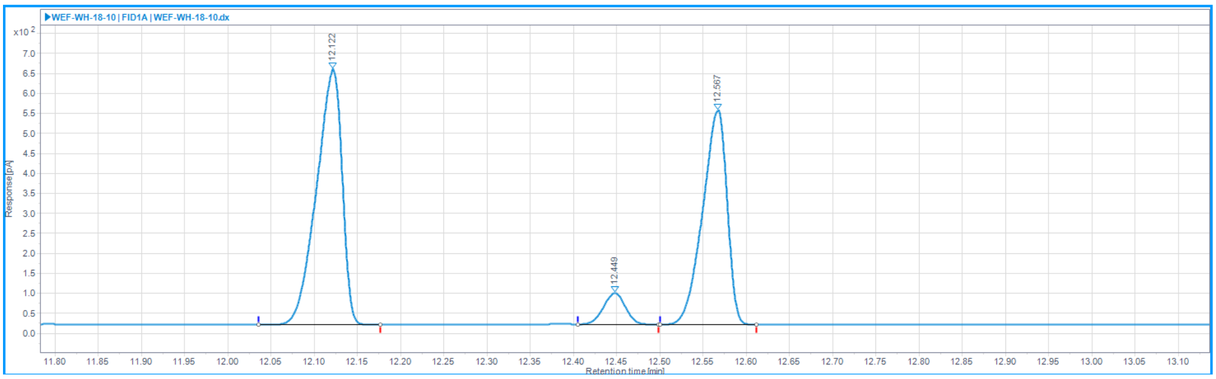

Figure S109. *GC-FID chromatogram of the biotransformation of (6E)-8-methyl-6-nonenoic acid ((E)-**11a**, R_t = 12.57 min), catalyzed by CvFAP Y466A. Z-isomer ((Z)-**11a**, R_t = 12.45 min). IS = 1-decanol (R_t = 12.12 min). (achiral, derivatized)*

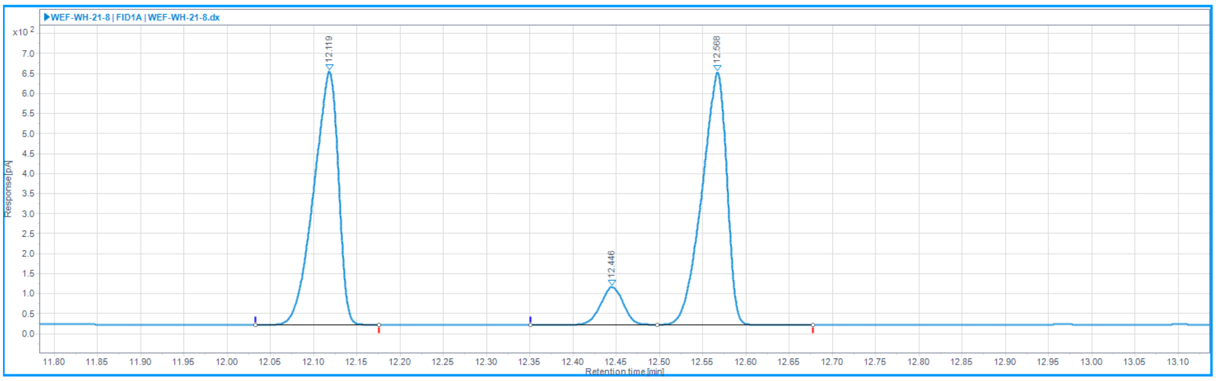

Figure S110. *GC-FID chromatogram of the substrate reference (6E)-8-methyl-6-nonenoic acid ((E)-**11a,** R_t = 12.57 min). Z-isomer ((Z)-**11a**, R_t = 12.45 min). IS = 1-decanol (R_t = 12.12 min). (achiral, derivatized)*

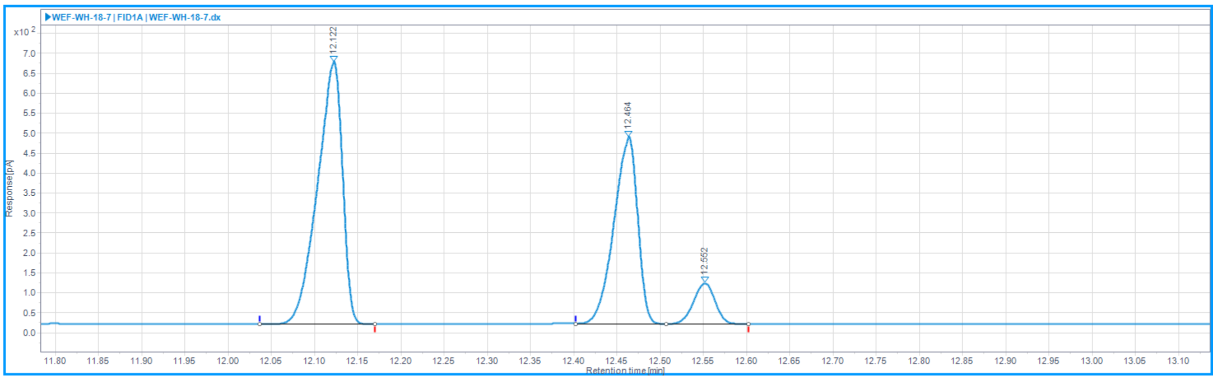

Figure S111. *GC-FID chromatogram of the biotransformation of (6Z)-8-methyl-6-nonenoic acid ((Z)-**11a**, 12.46 min), catalyzed by CvFAP Y466A. E-isomer ((E)-**11a**, R_t = 12.55 min). IS = 1-decanol (R_t = 12.12 min). (achiral, derivatized)*

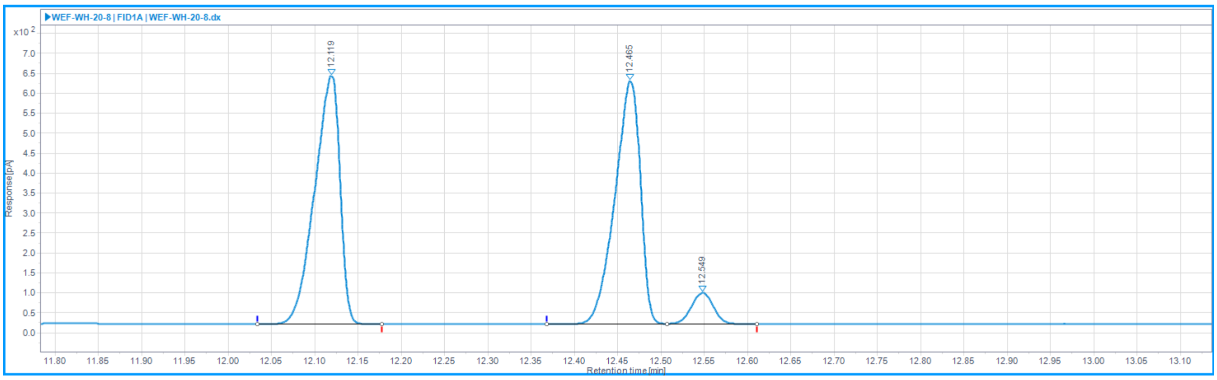

Figure S112. *GC-FID chromatogram of the substrate reference (6Z)-8-methyl-6-nonenoic acid ((Z)-**11a,** R_t = 12.47 min). E-isomer ((E)-**11a**, R_t = 12.55 min). IS = 1-decanol (R_t = 12.12 min). (achiral, derivatized)*

8.1.4 Carbohydroxylation of C=C bonds

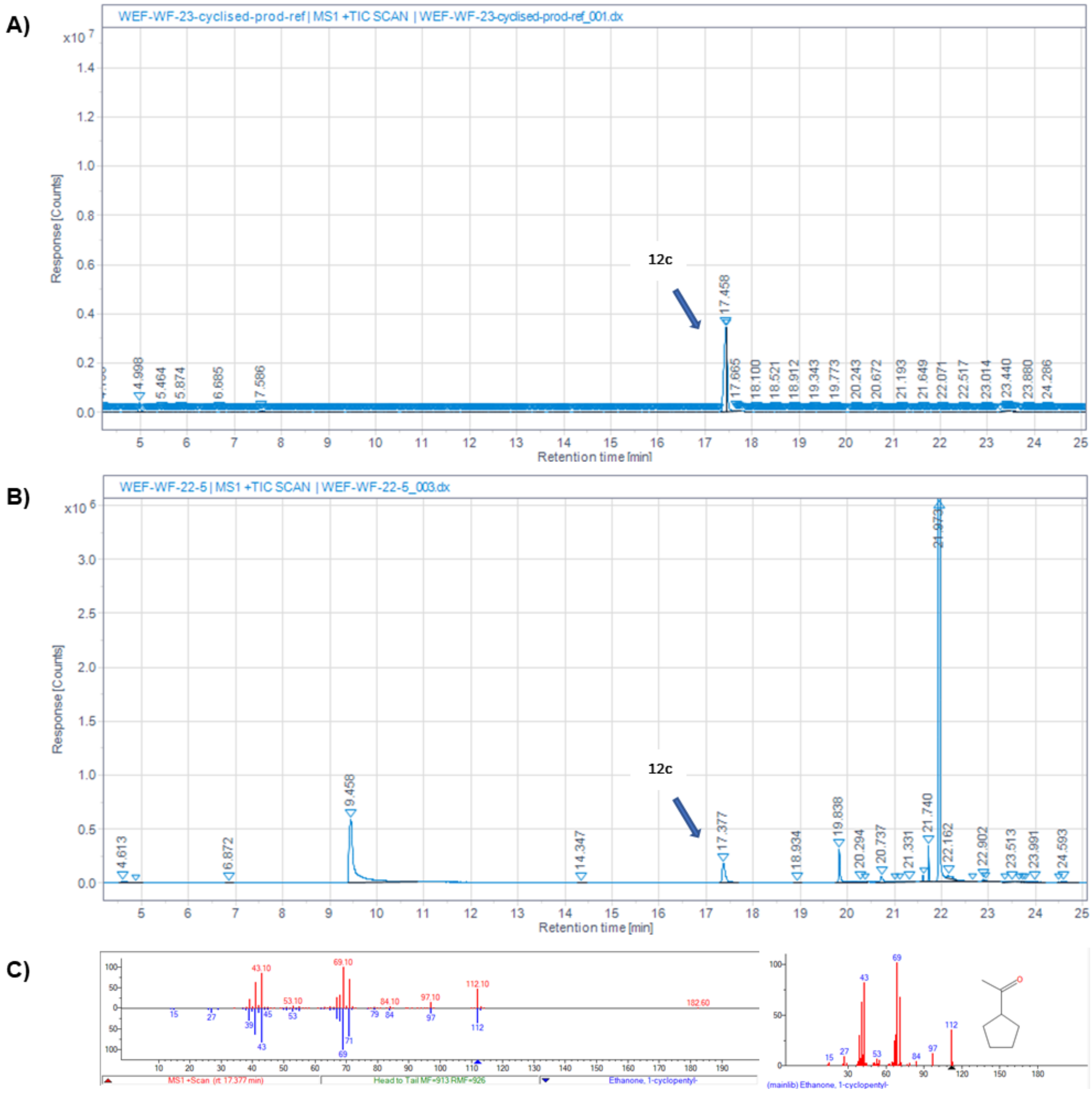

Figure S113. *GC-EI-MS chromatograms of the authentical product reference of **12c** and biotransformation of **12a** using CvFAP V453A. **A**) Authentical product reference of **12c** at R_t = 17.5 min. **B**) Biotransformation of CvFAP V453A using **12a** showcasing the peak corresponding to the cyclic ketone **12c**. **C**) Fragmentation pattern of the analyte of the biotransformation corresponding to the peak at R_t = 17.4 min (red) alongside the most fitting fragmentation suggested by the NIST library (blue) and its predicted structure.*

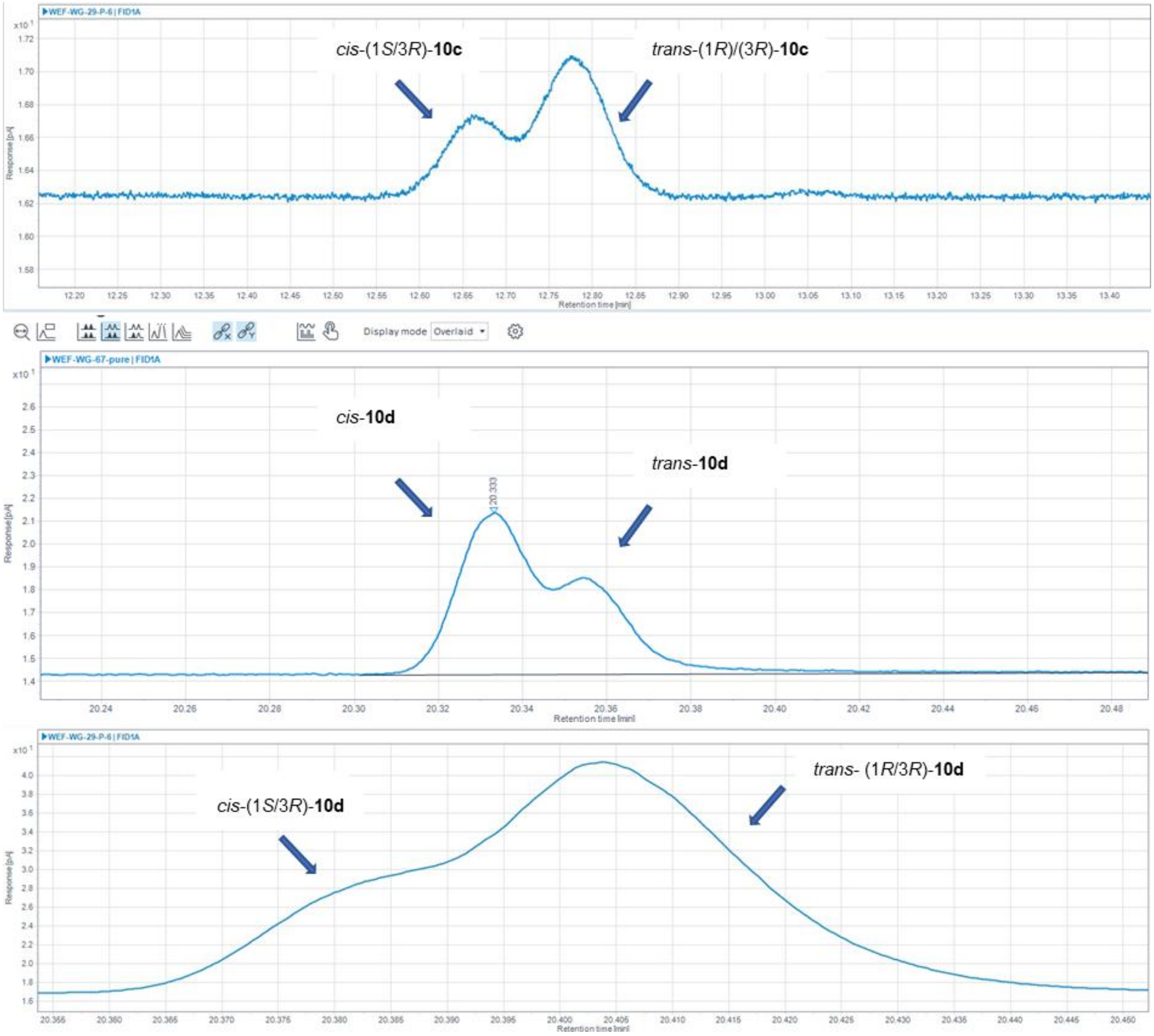

Figure S114. *GC- FID chromatograms of the authentical product reference of **10d** and biotransformation of (R)-**10a**. (**top**): Biotransformation of CvFAP M3 C432A in form of resuspended whole cells using (R)-**10a**, showcasing the two peaks corresponding to traces of the diastereomers of **10c**. (**middle**): authentical product reference of the cyclic alcohol **10d**. (**bottom**): peaks of the biotransformation shown in (top), corresponding to the cyclic alcohol **10d**.*

8.2 NMR spectra

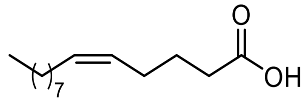

(*Z*)-**8a**

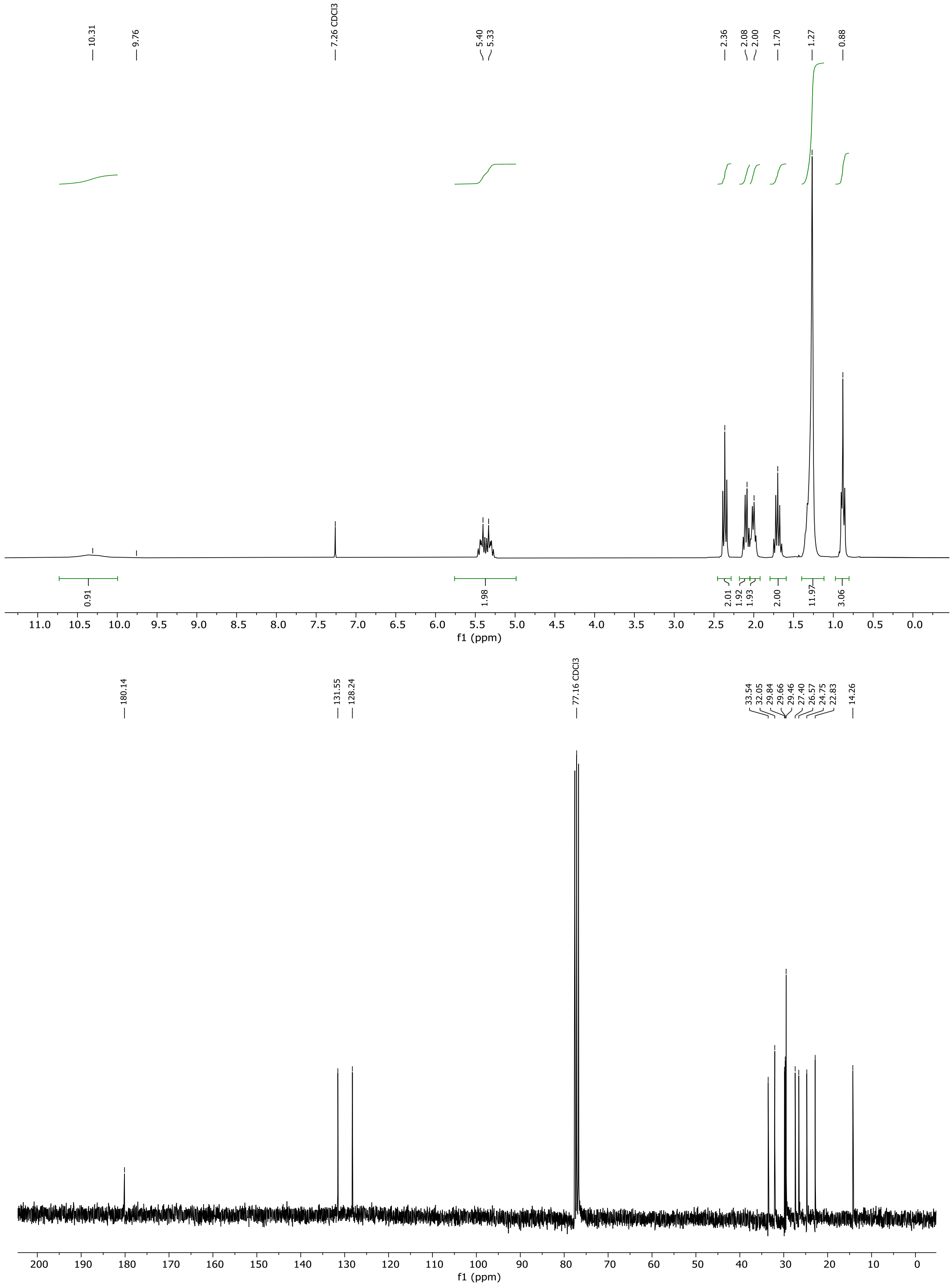

Figure S115. *^{1}H-and ^{13}C-NMR of (Z)-**8a** [300 MHz/75 MHz, $CDCl_3$].*

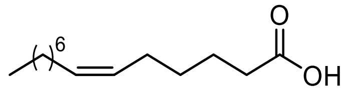

(Z)-**9a**

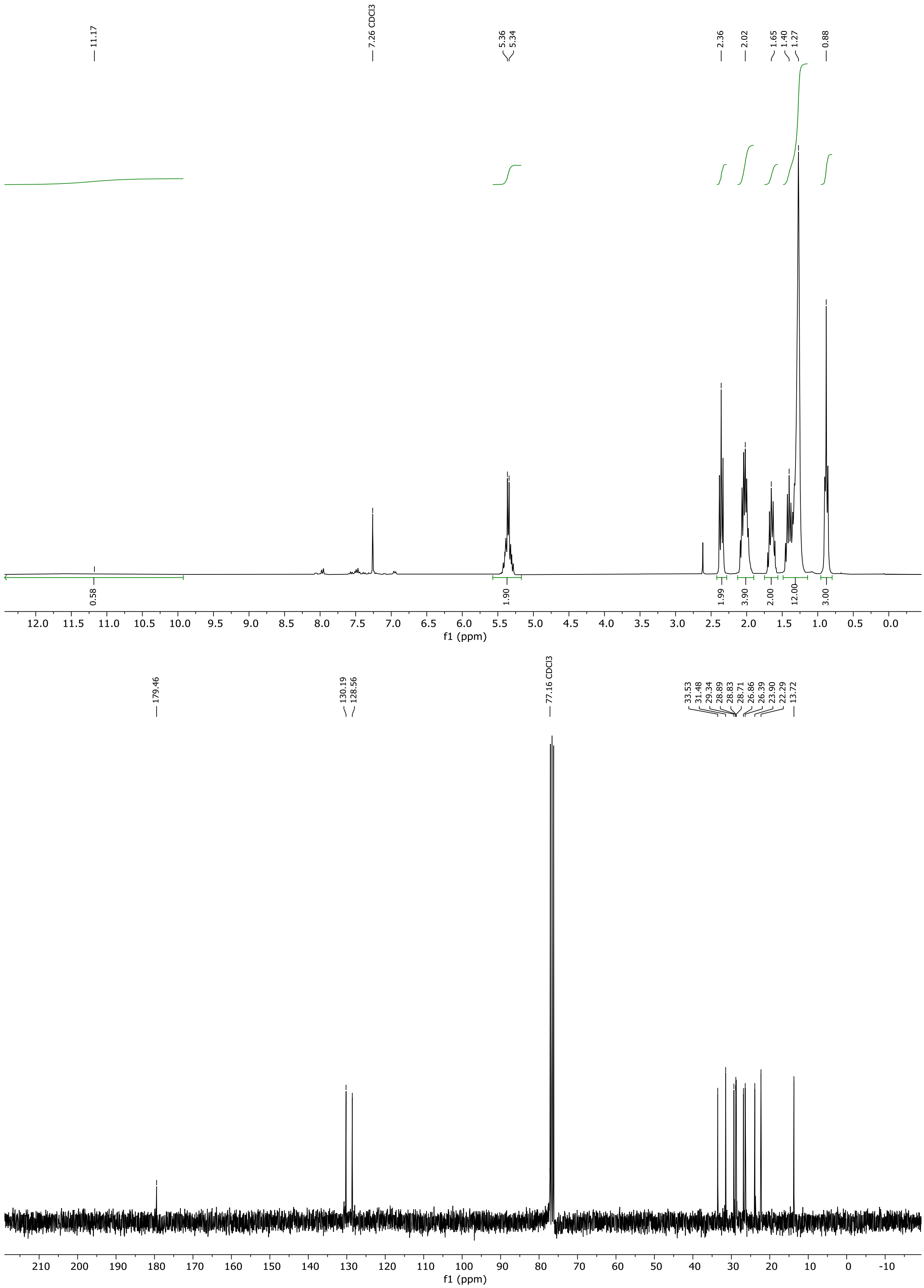

Figure S116. *^{1}H-and ^{13}C-NMR of (Z)-**9a** [300 MHz/75 MHz, $CDCl_3$].*

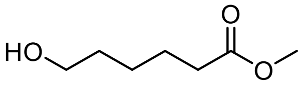

13

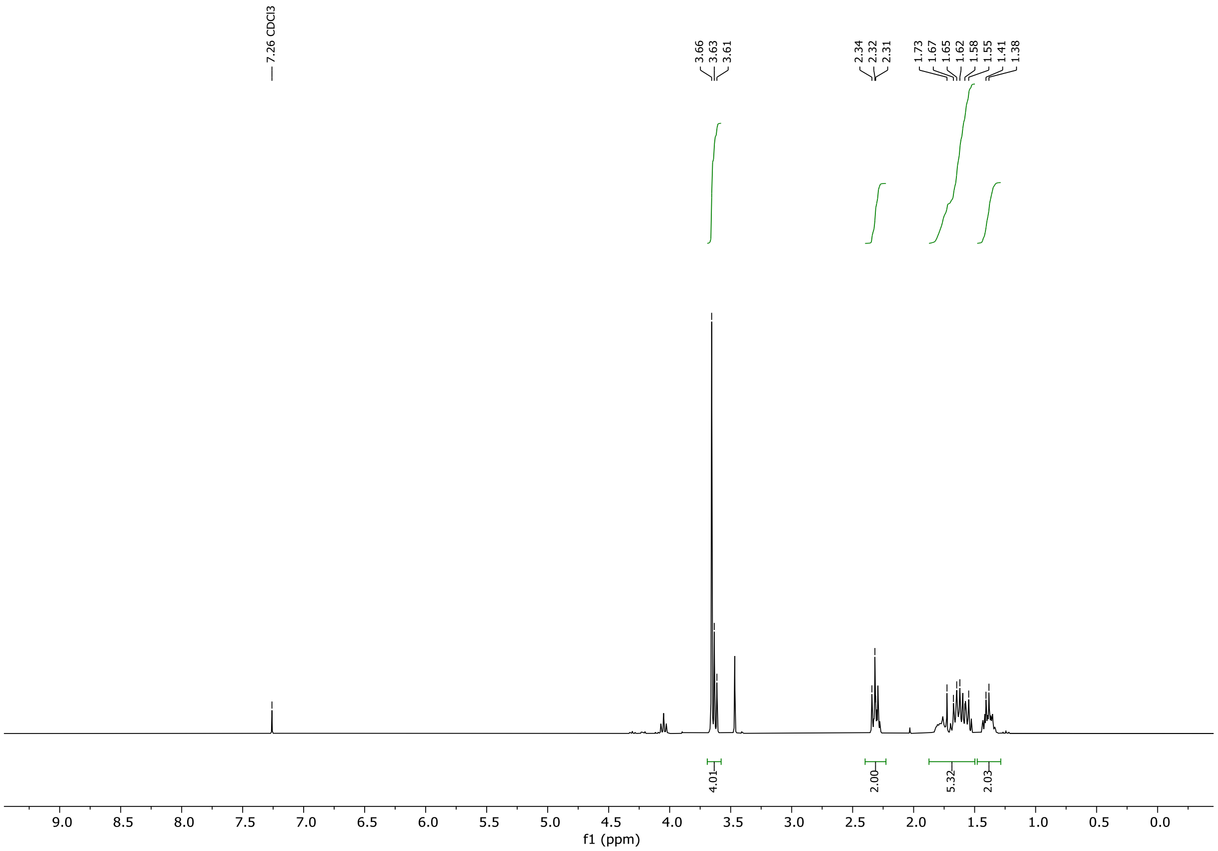

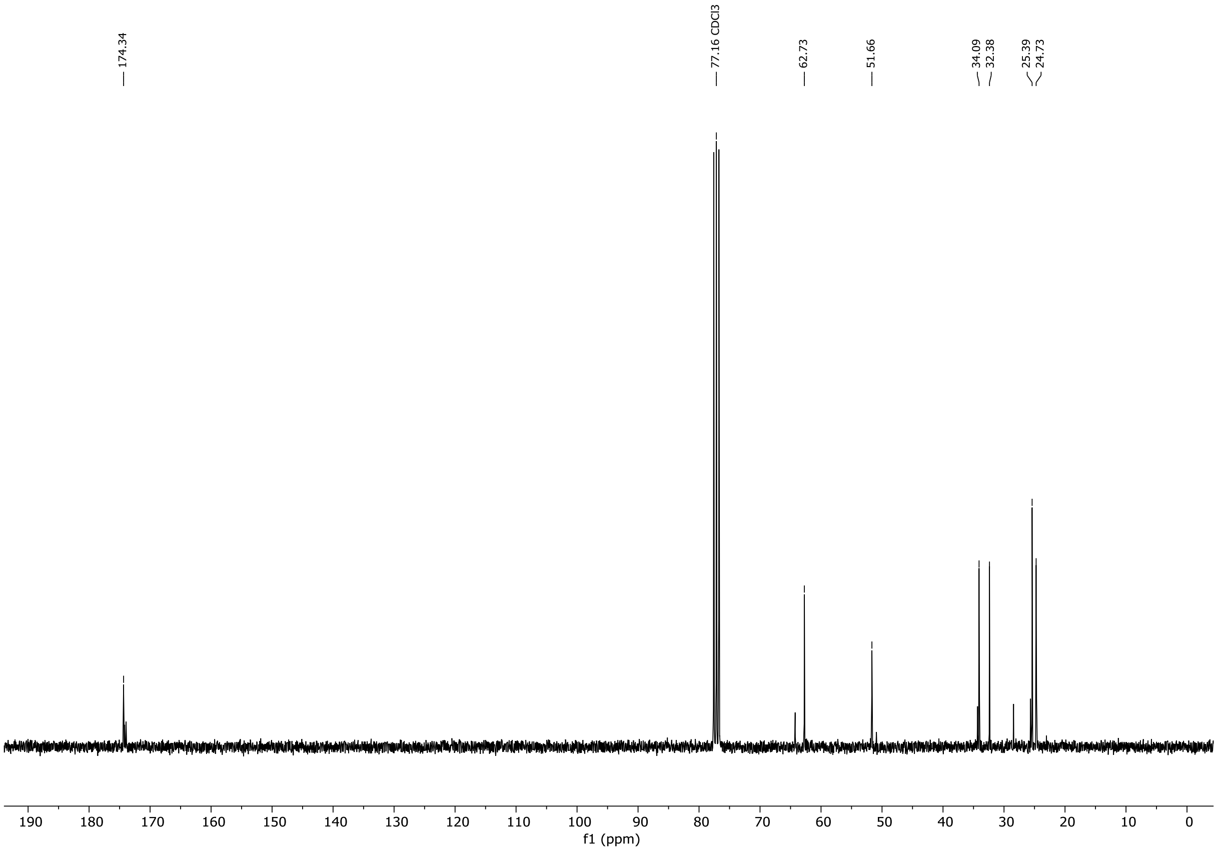

Figure S117. *^{1}H-and ^{13}C-NMR of **13** [300 MHz/75 MHz, $CDCl_3$].*

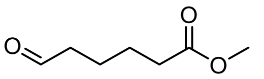

14

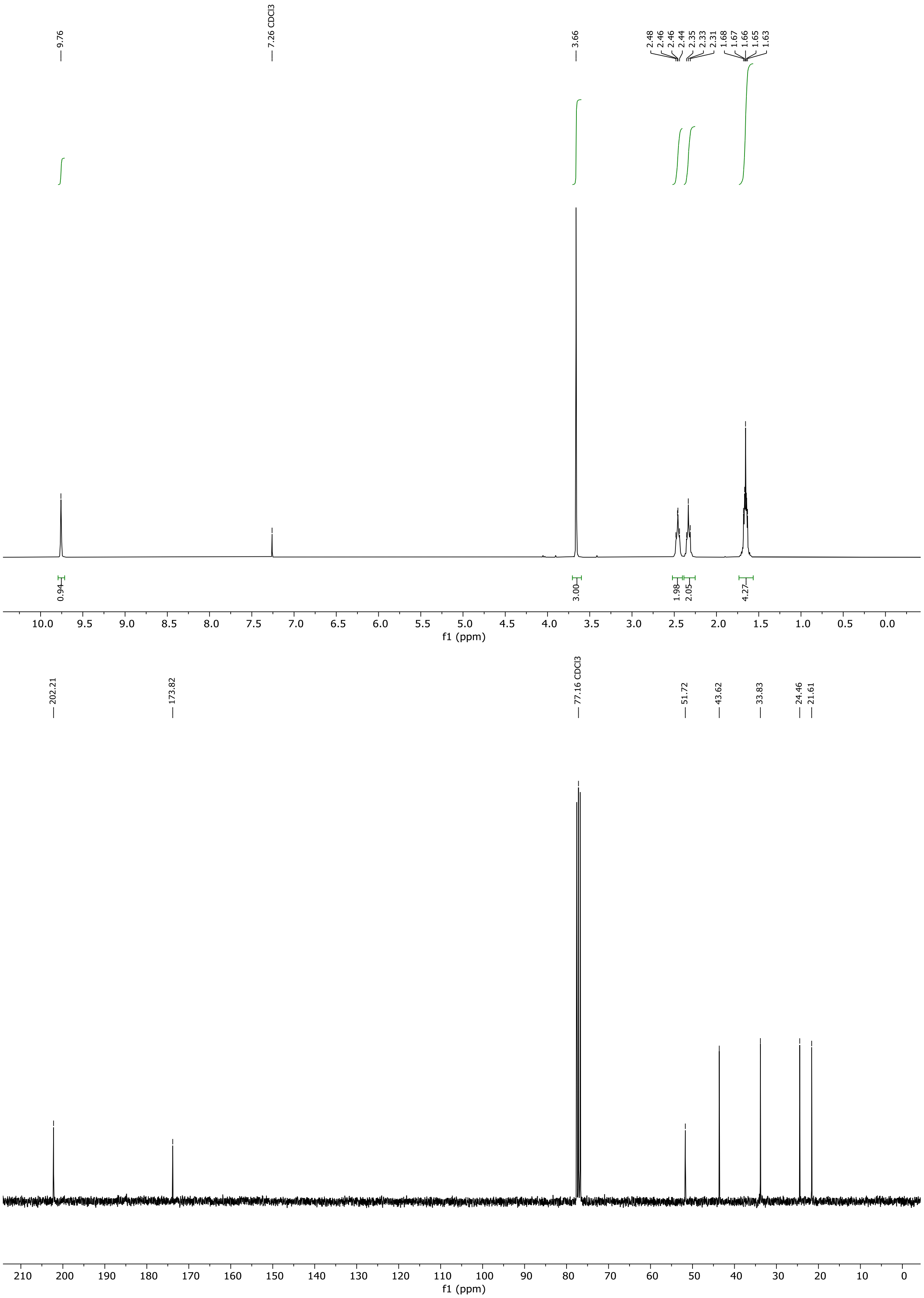

Figure S118. *^{1}H-and ^{13}C-NMR of* ***14*** *[300 MHz/75 MHz, $CDCl_3$].*

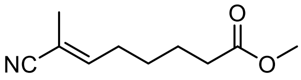

15

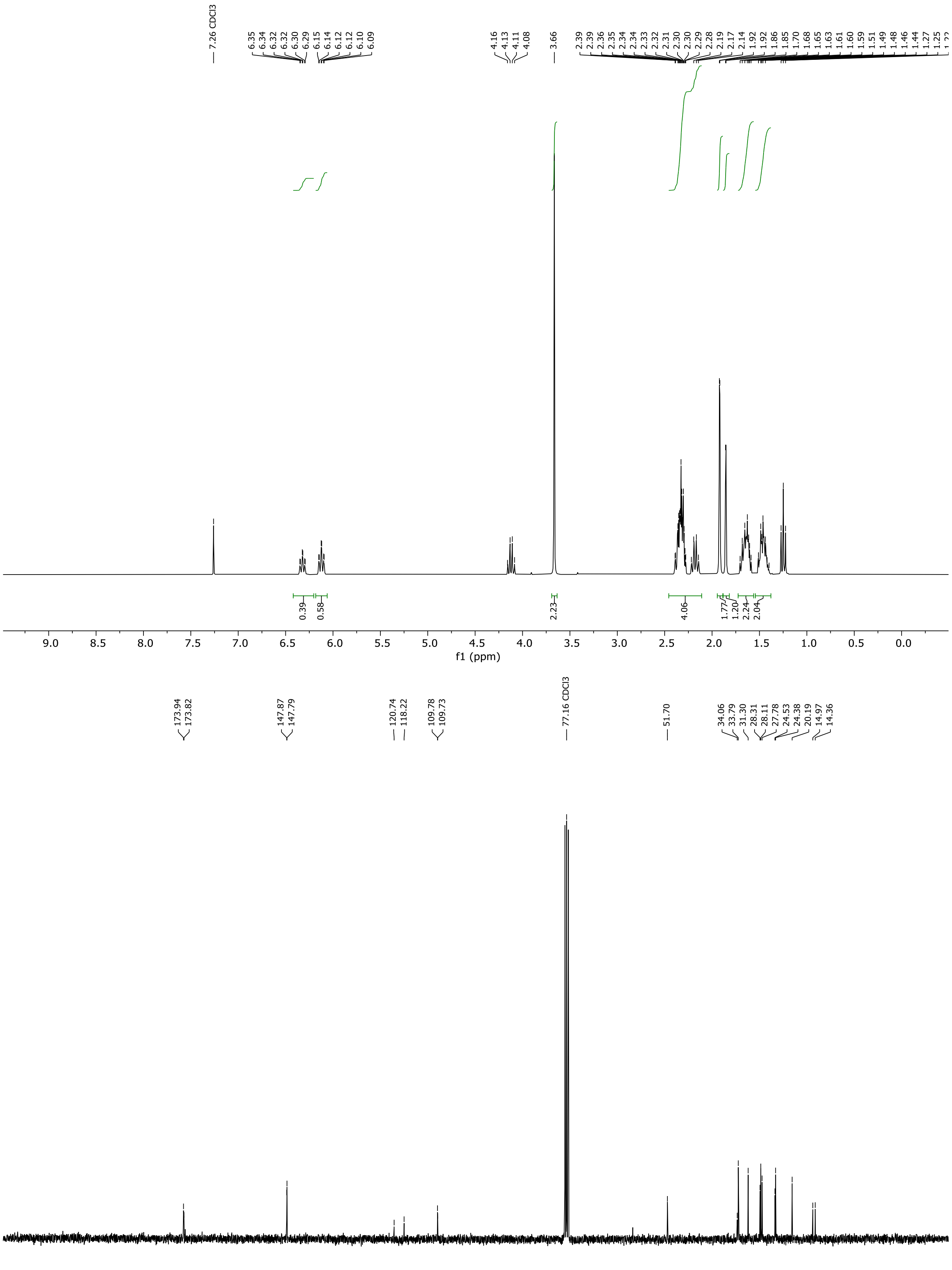

Figure S119. *^{1}H-and ^{13}C-NMR of (E/Z)-**15** [300 MHz/75 MHz, $CDCl_3$].*

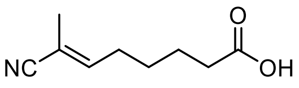

12a

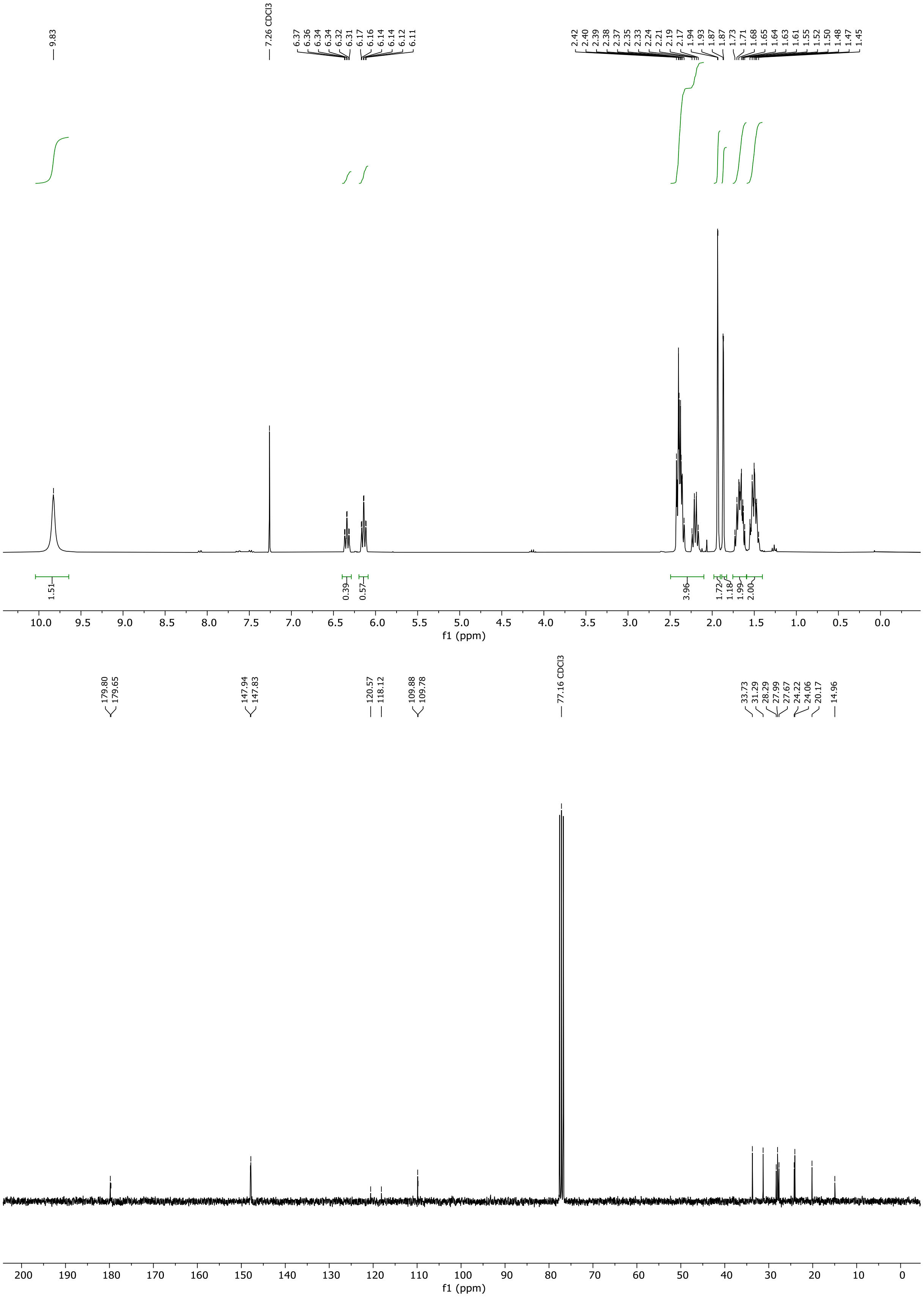

Figure S120. *^{1}H-and ^{13}C-NMR of (E/Z)-**12a** [300 MHz/75 MHz, $CDCl_3$].*

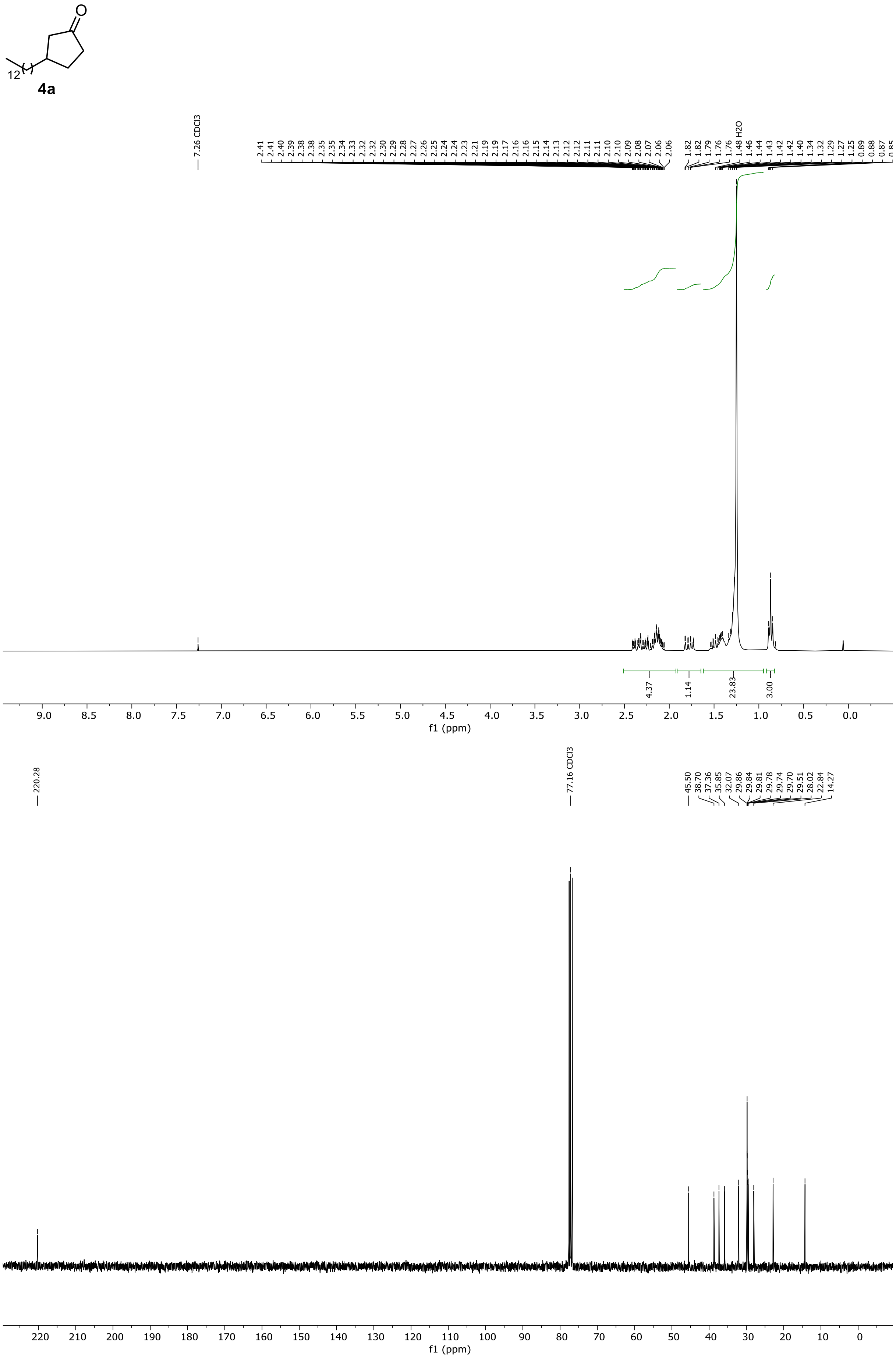

Figure S121. *^{1}H-and ^{13}C-NMR of **4a** [300 MHz/75 MHz, $CDCl_3$].*

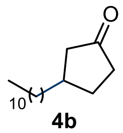

4b

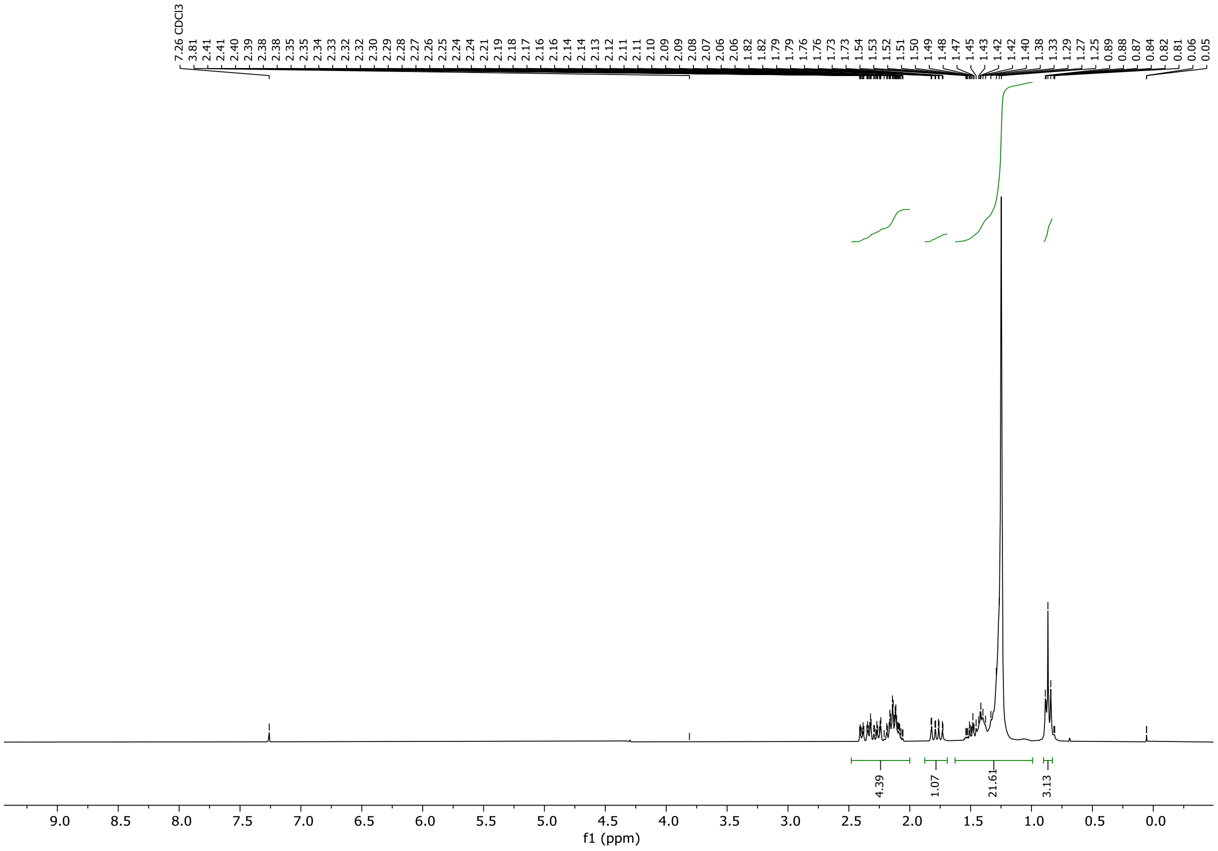

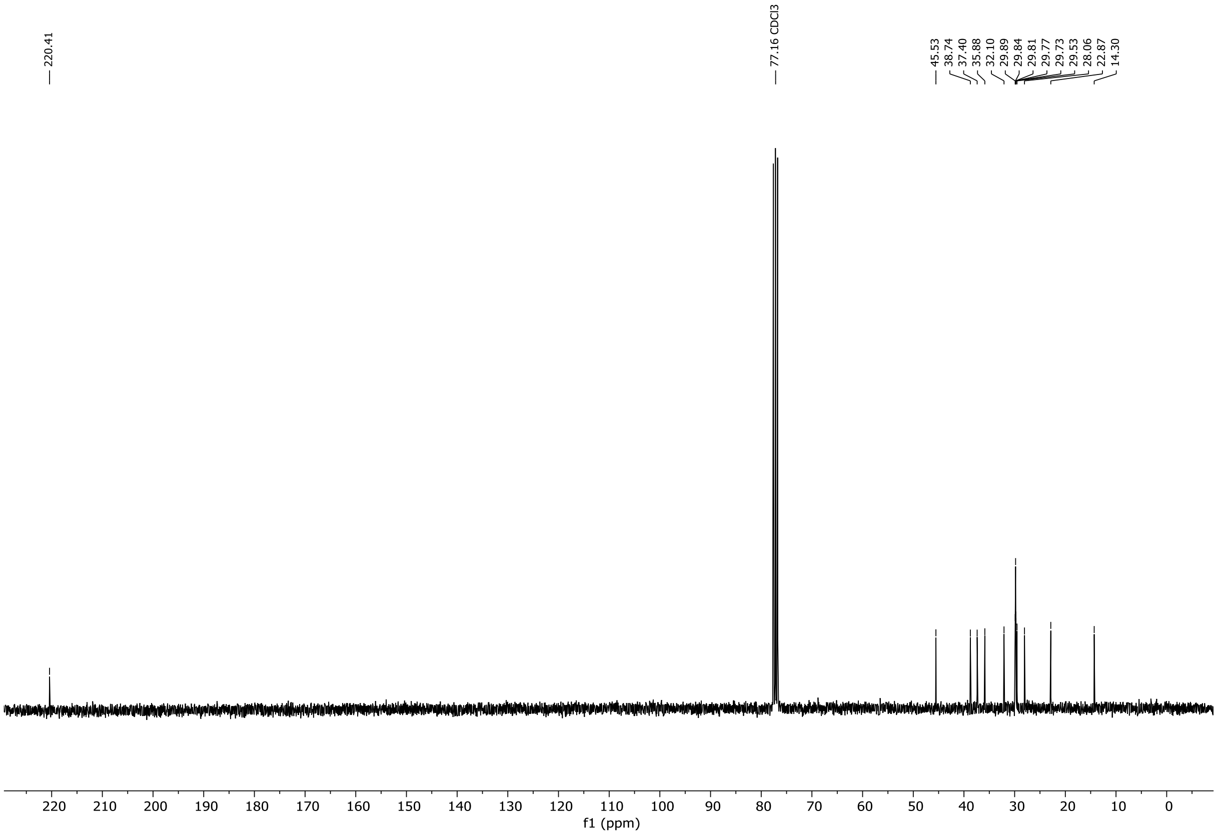

Figure S122. *^{1}H-and ^{13}C-NMR of **4b** [300 MHz/75 MHz, $CDCl_3$].*

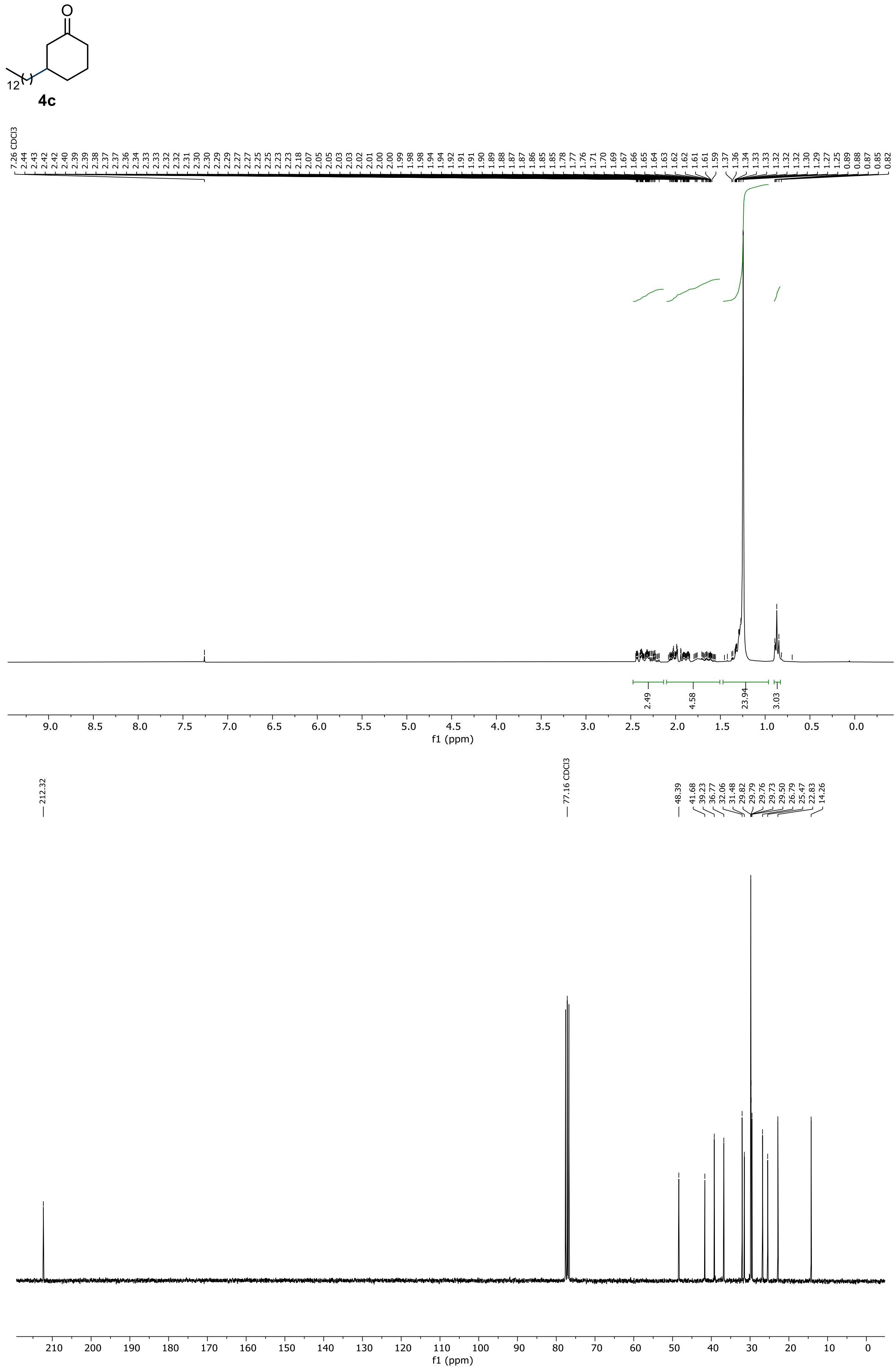

Figure S123. ^{1}*H-and* ^{13}*C-NMR of* ***4c*** *[300 MHz/75 MHz, CDCl$_3$].*

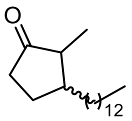
O
12

rac-**4d**

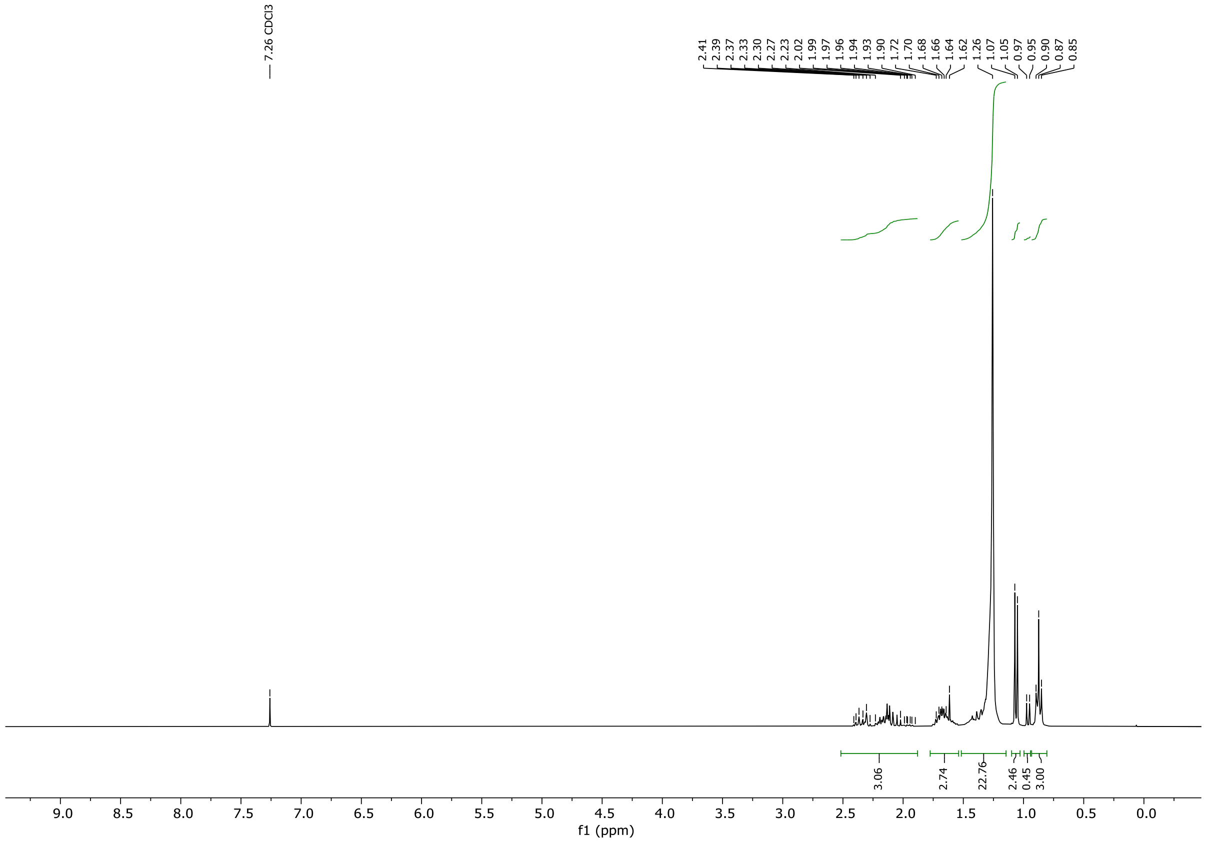
7.26 CDCl3
2.41
2.39
2.37
2.33
2.30
2.27
2.23
2.02
1.99
1.97
1.96
1.94
1.93
1.90
1.72
1.70
1.68
1.66
1.64
1.62
1.26
1.07
1.05
0.97
0.95
0.90
0.87
0.85
3.06
2.74
22.76
2.46
0.45
3.00
9.0 8.5 8.0 7.5 7.0 6.5 6.0 5.5 5.0 4.5 4.0 3.5 3.0 2.5 2.0 1.5 1.0 0.5 0.0
f1 (ppm)

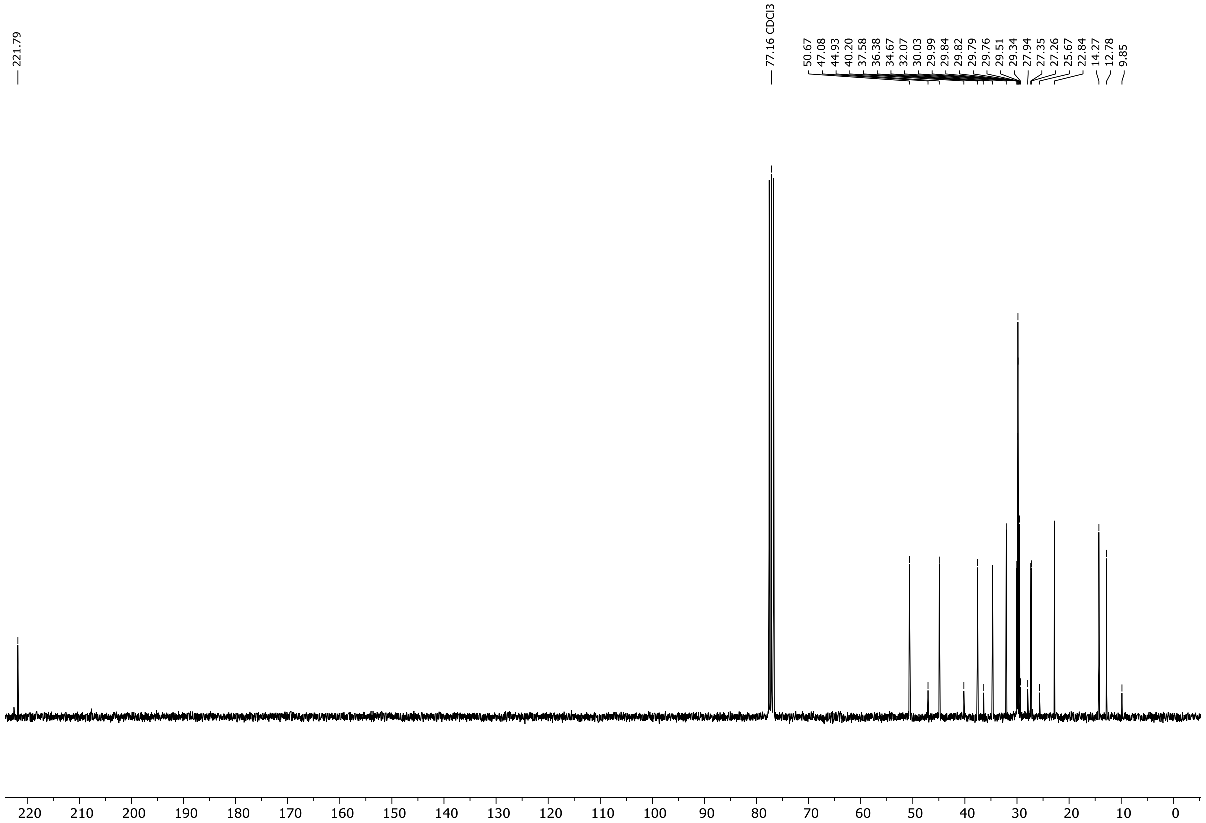
221.79
77.16 CDCl3
50.67
47.08
44.93
40.20
37.58
36.38
34.67
32.07
30.03
29.99
29.84
29.82
29.79
29.76
29.51
29.34
27.94
27.35
27.26
25.67
22.84
14.27
12.78
9.85
220 210 200 190 180 170 160 150 140 130 120 110 100 90 80 70 60 50 40 30 20 10 0
f1 (ppm)

Figure S124. *^{1}H-and ^{13}C-NMR of rac-**4d** [300 MHz/75 MHz, $CDCl_3$].*

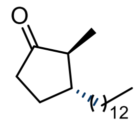

(2*S*/3*R*)-**4d**

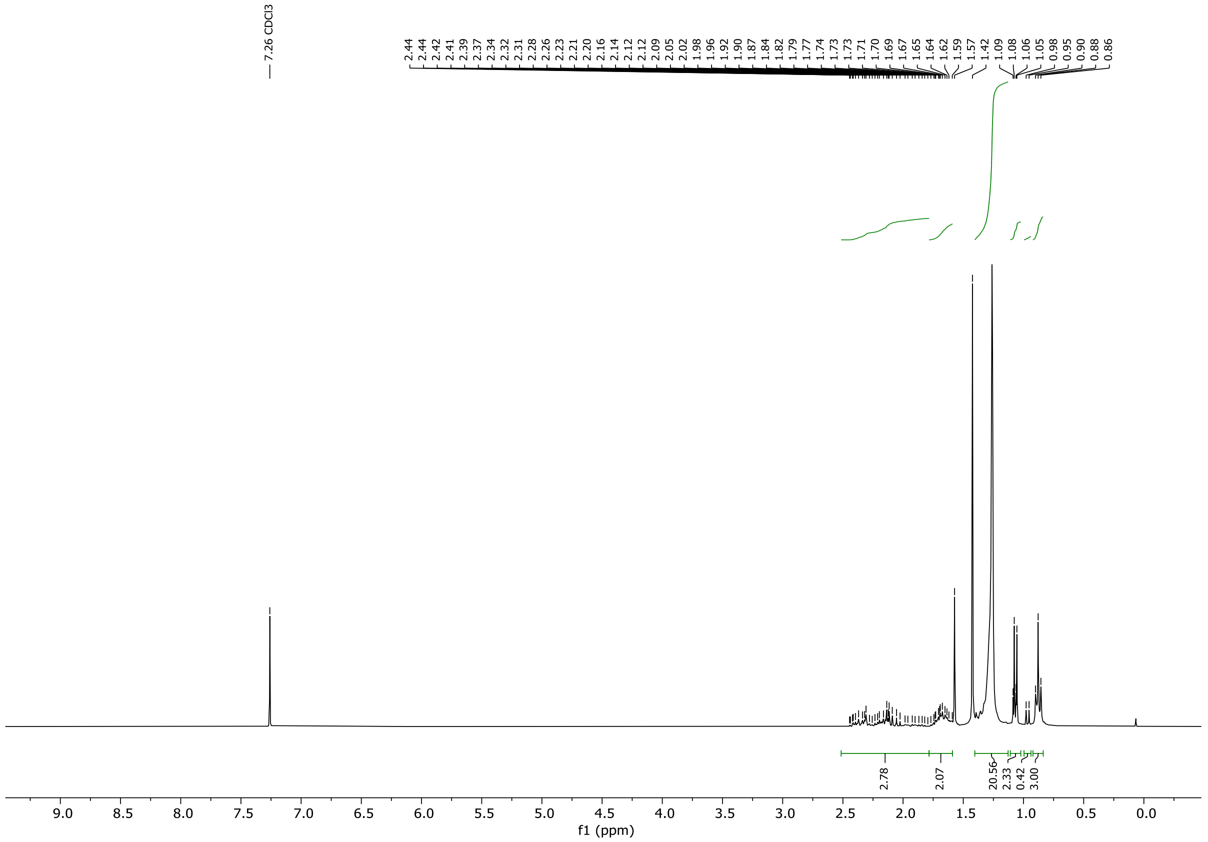

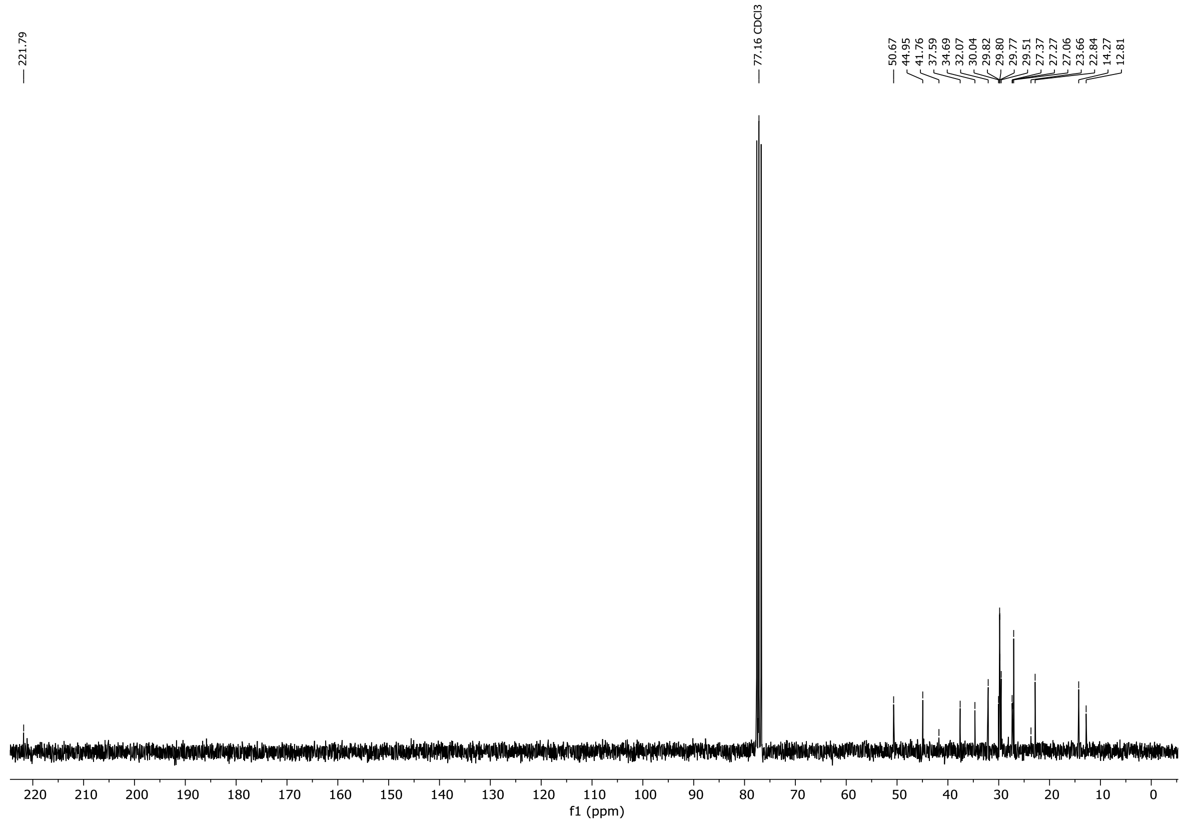

Figure S125. *^{1}H-and ^{13}C-NMR of (2S/3R)-**4d** [300 MHz/75 MHz, CDCl$_3$].*

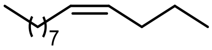

8b

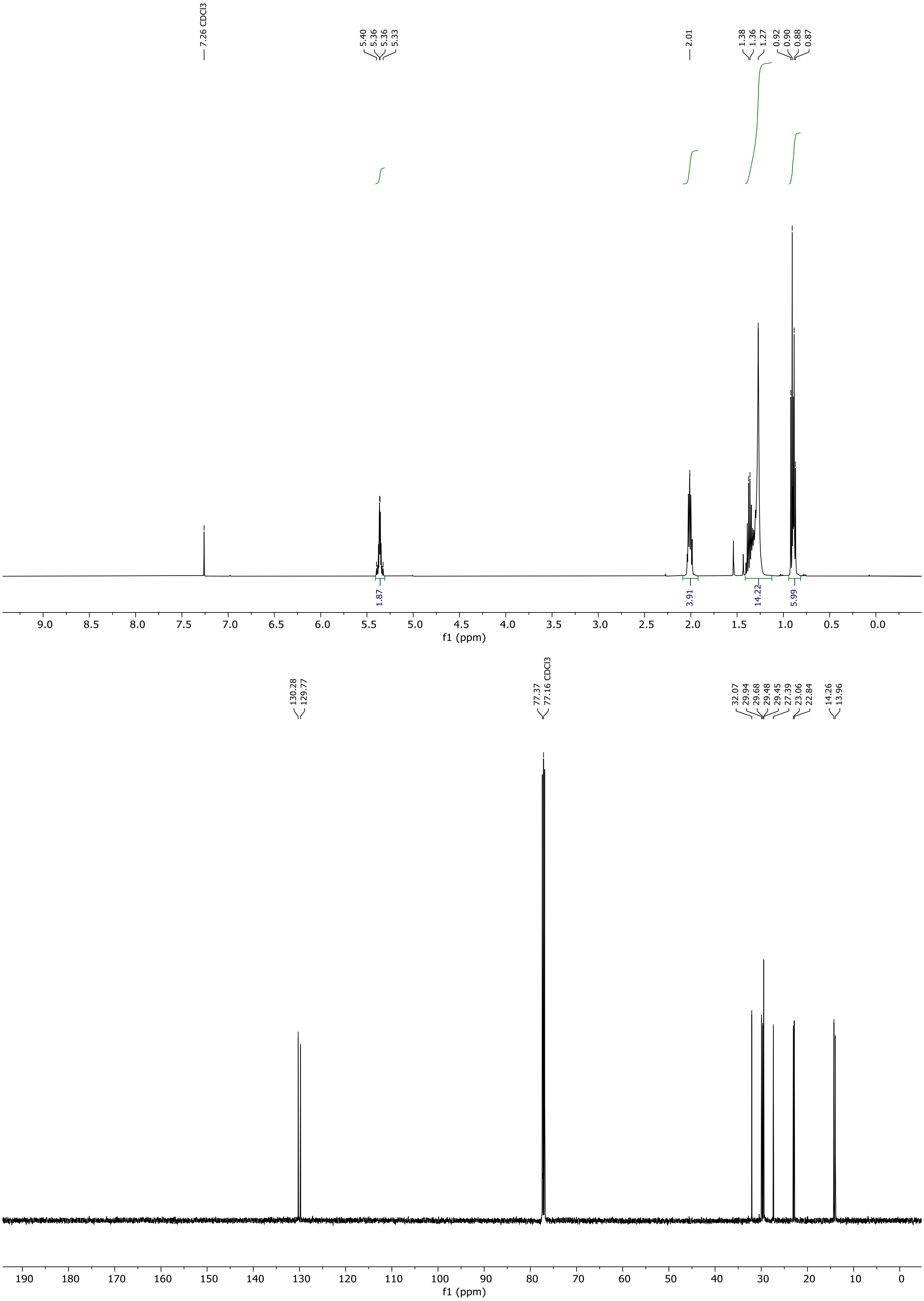

Figure S126. *^{1}H-and ^{13}C-NMR of **8b** [500 MHz/125 MHz, $CDCl_3$].*

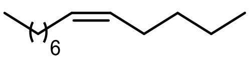

9b

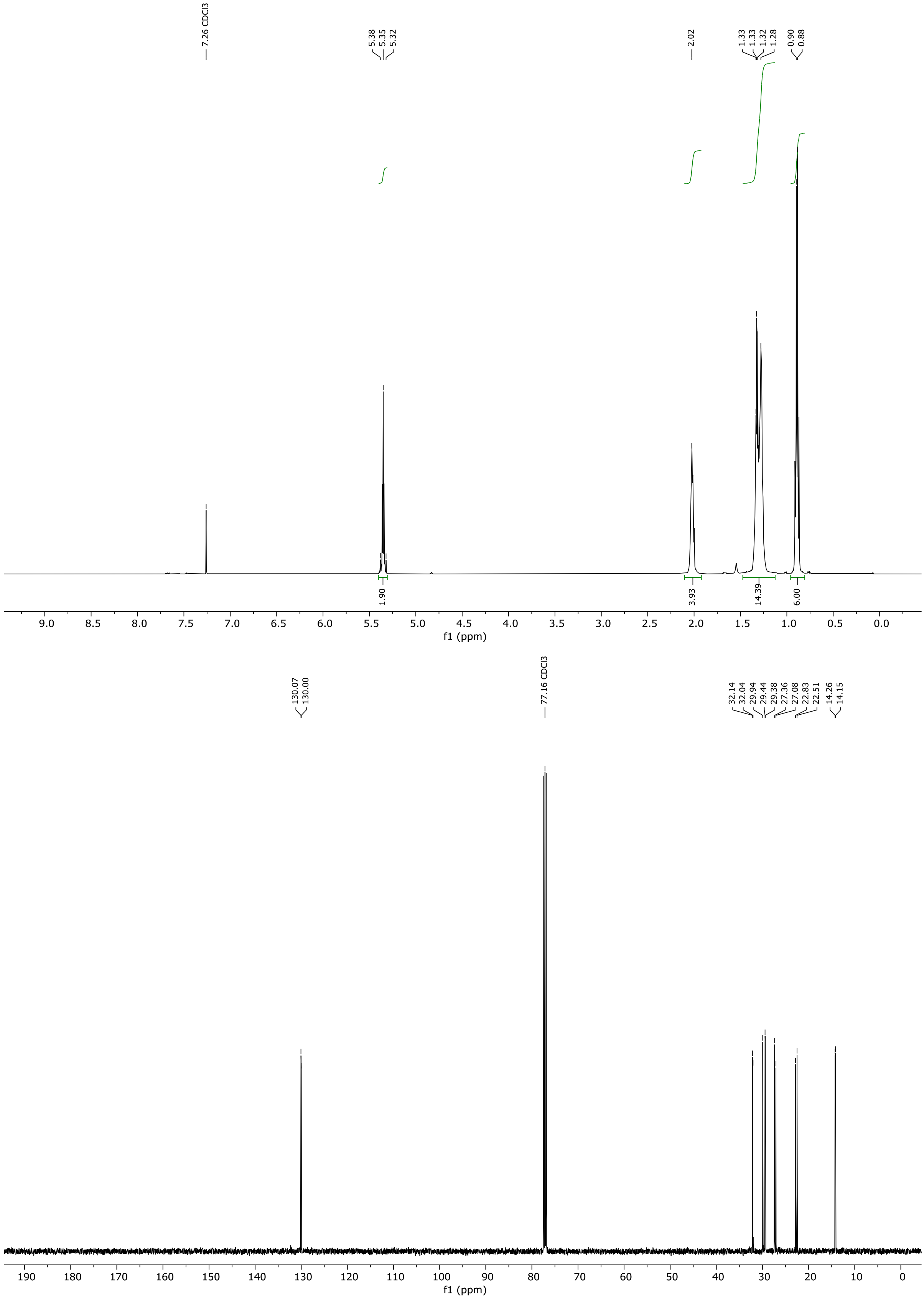

Figure S127. *^{1}H-and ^{13}C-NMR of **9b** [500 MHz/125 MHz, $CDCl_3$].*

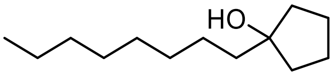

16

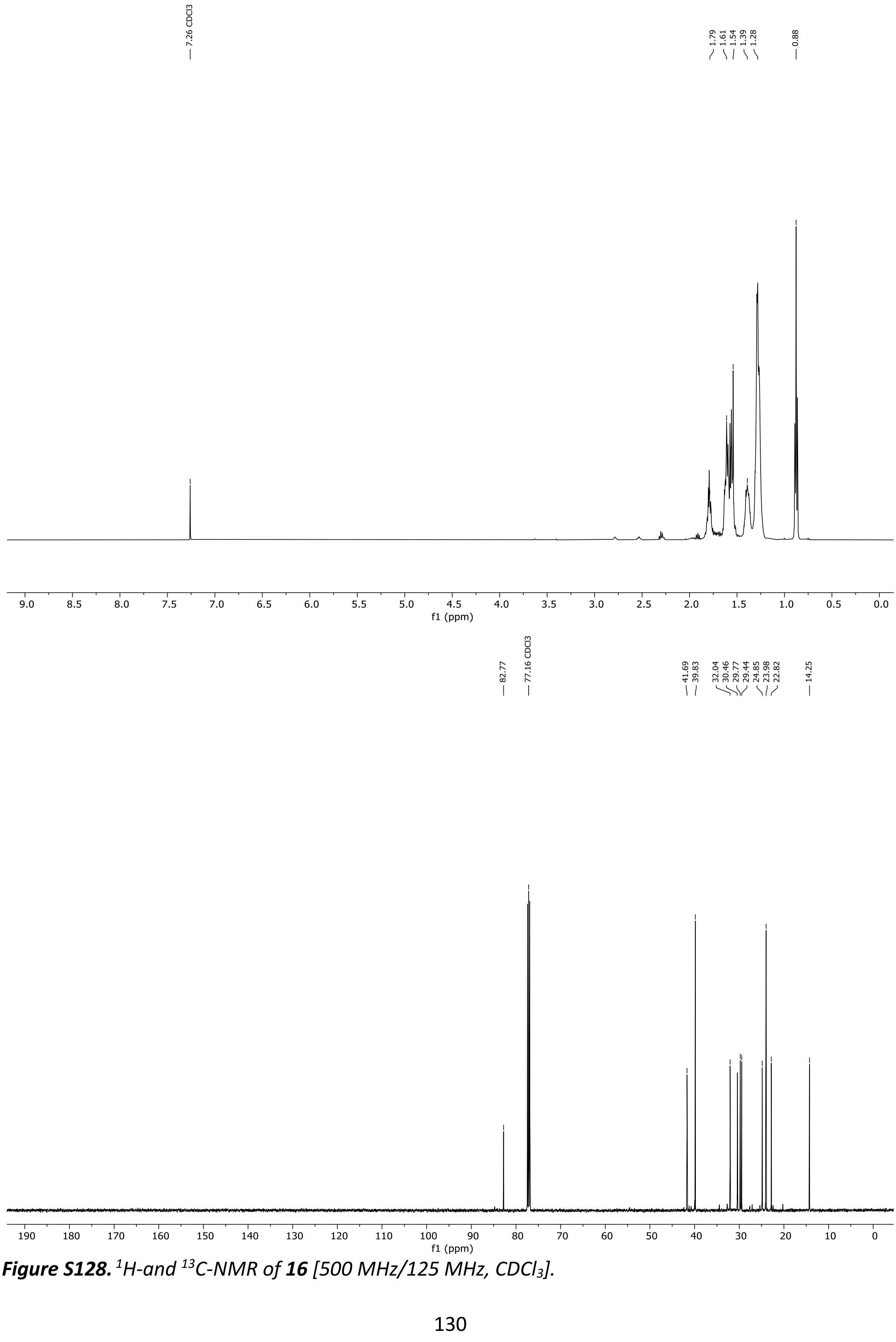

Figure S128. *^{1}H-and ^{13}C-NMR of **16** [500 MHz/125 MHz, $CDCl_3$].*

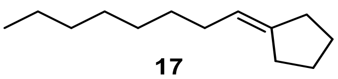

17

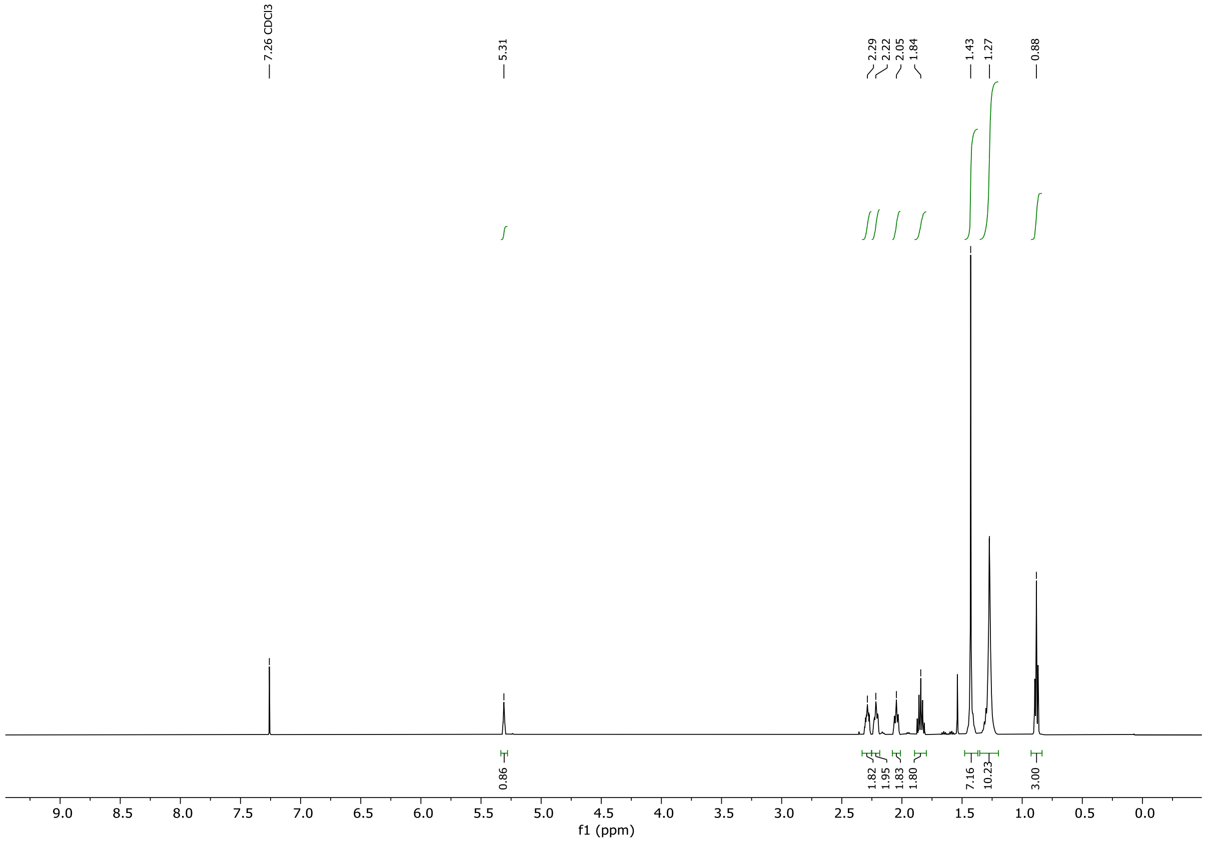

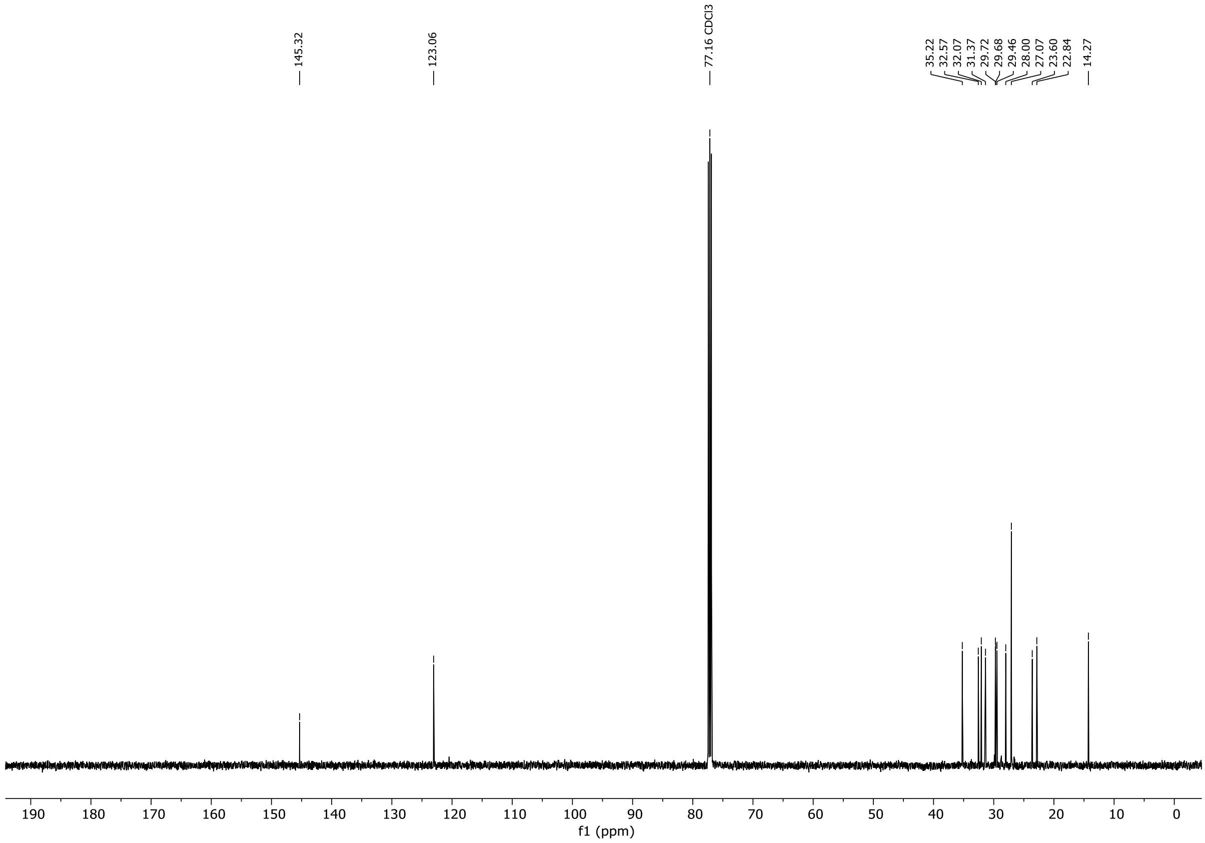

Figure S129. *^{1}H-and ^{13}C-NMR of **17** [500 MHz/125 MHz, CDCl$_3$].*

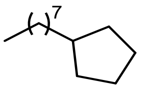

9c

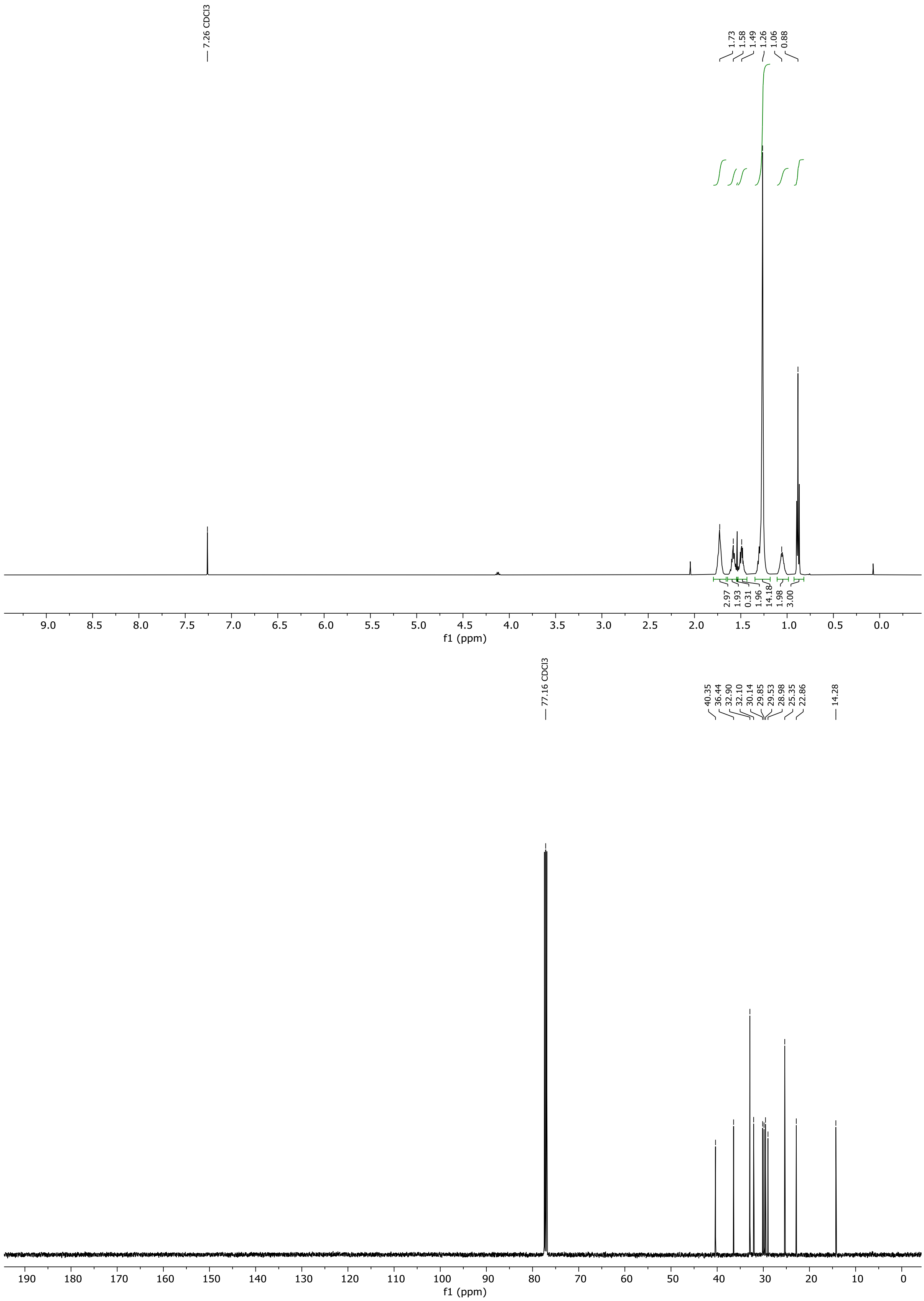

Figure S130. *^{1}H-and ^{13}C-NMR of **9c** [500 MHz/125 MHz, $CDCl_3$].*

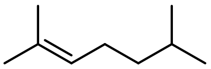

10b

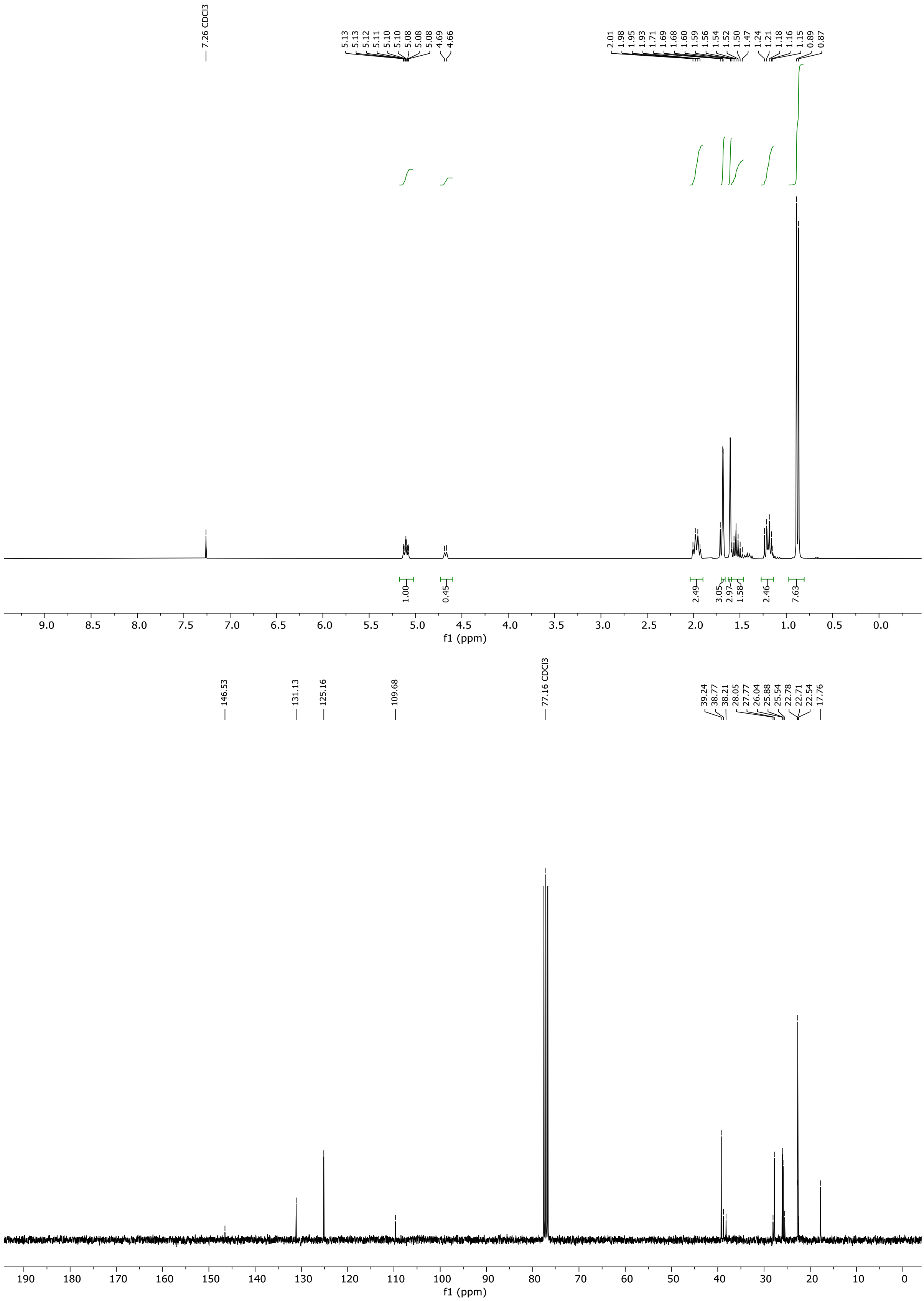

Figure S131. *^{1}H-and ^{13}C-NMR of **10b** [300 MHz/75 MHz, $CDCl_3$].*

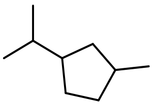

10c

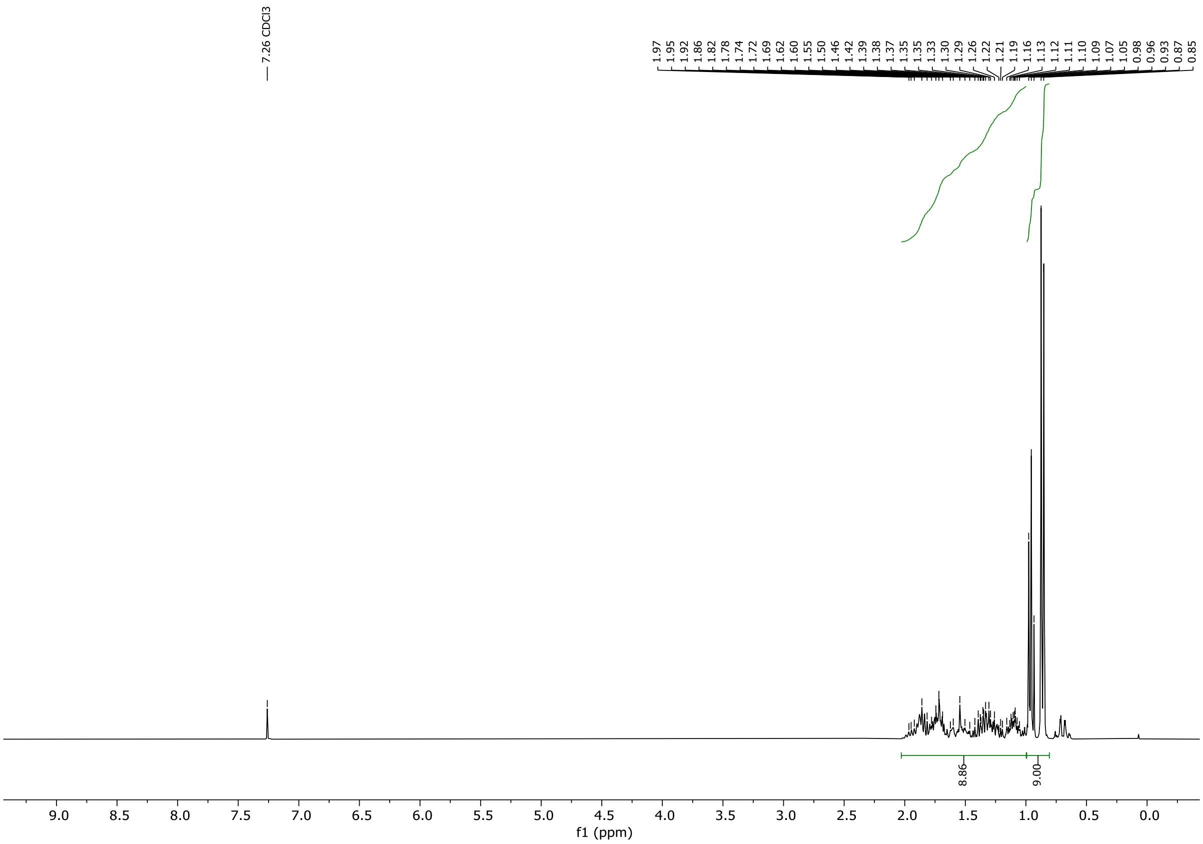
7.26 CDCl3
1.97
1.95
1.92
1.86
1.82
1.78
1.74
1.72
1.69
1.62
1.60
1.55
1.50
1.46
1.42
1.39
1.38
1.37
1.35
1.35
1.33
1.30
1.29
1.26
1.22
1.21
1.19
1.16
1.13
1.12
1.11
1.10
1.09
1.07
1.05
0.98
0.96
0.93
0.87
0.85
8.86
9.00
9.0 8.5 8.0 7.5 7.0 6.5 6.0 5.5 5.0 4.5 4.0 3.5 3.0 2.5 2.0 1.5 1.0 0.5 0.0
f1 (ppm)

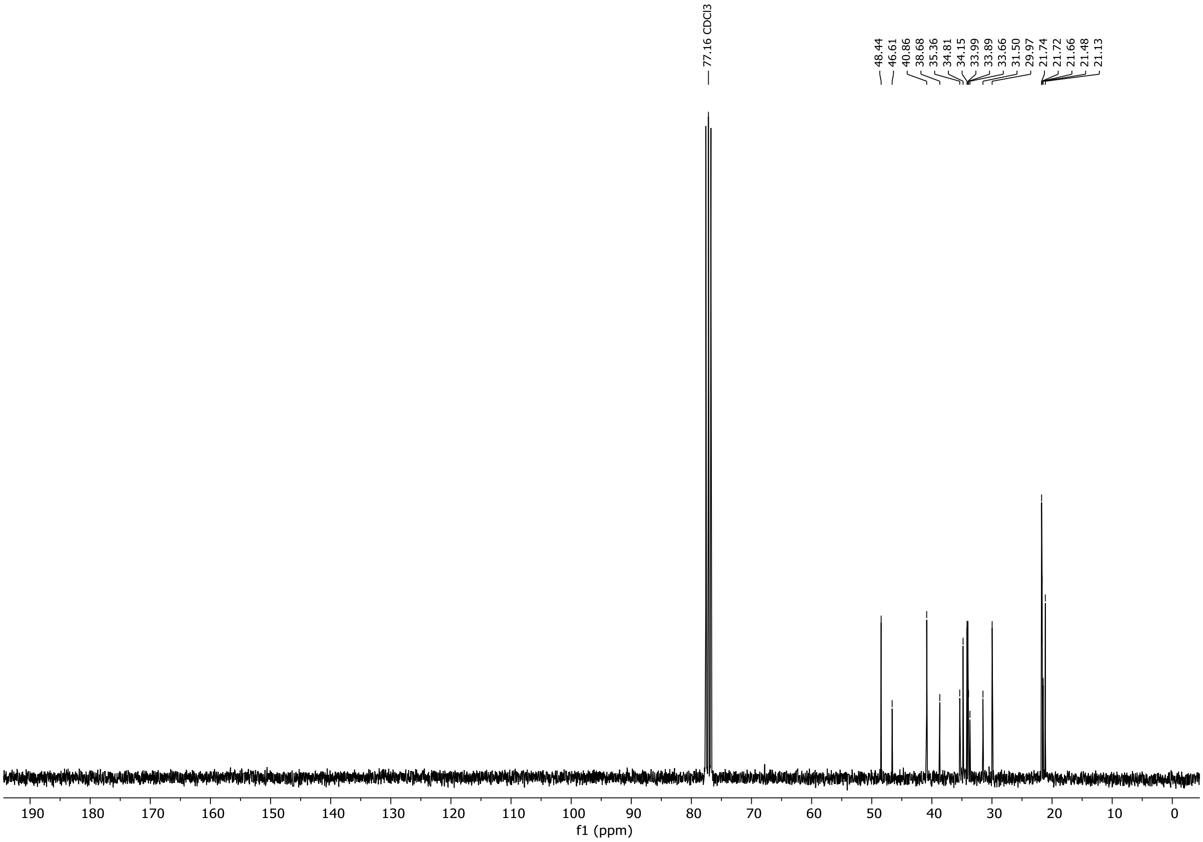
77.16 CDCl3
48.44
46.61
40.86
38.68
35.36
34.81
34.15
33.99
33.89
33.66
31.50
29.97
21.74
21.72
21.66
21.48
21.13
190 180 170 160 150 140 130 120 110 100 90 80 70 60 50 40 30 20 10 0
f1 (ppm)

Figure S132. *^{1}H-and ^{13}C-NMR of **10c** [700 MHz/175 MHz, CDCl$_3$].*

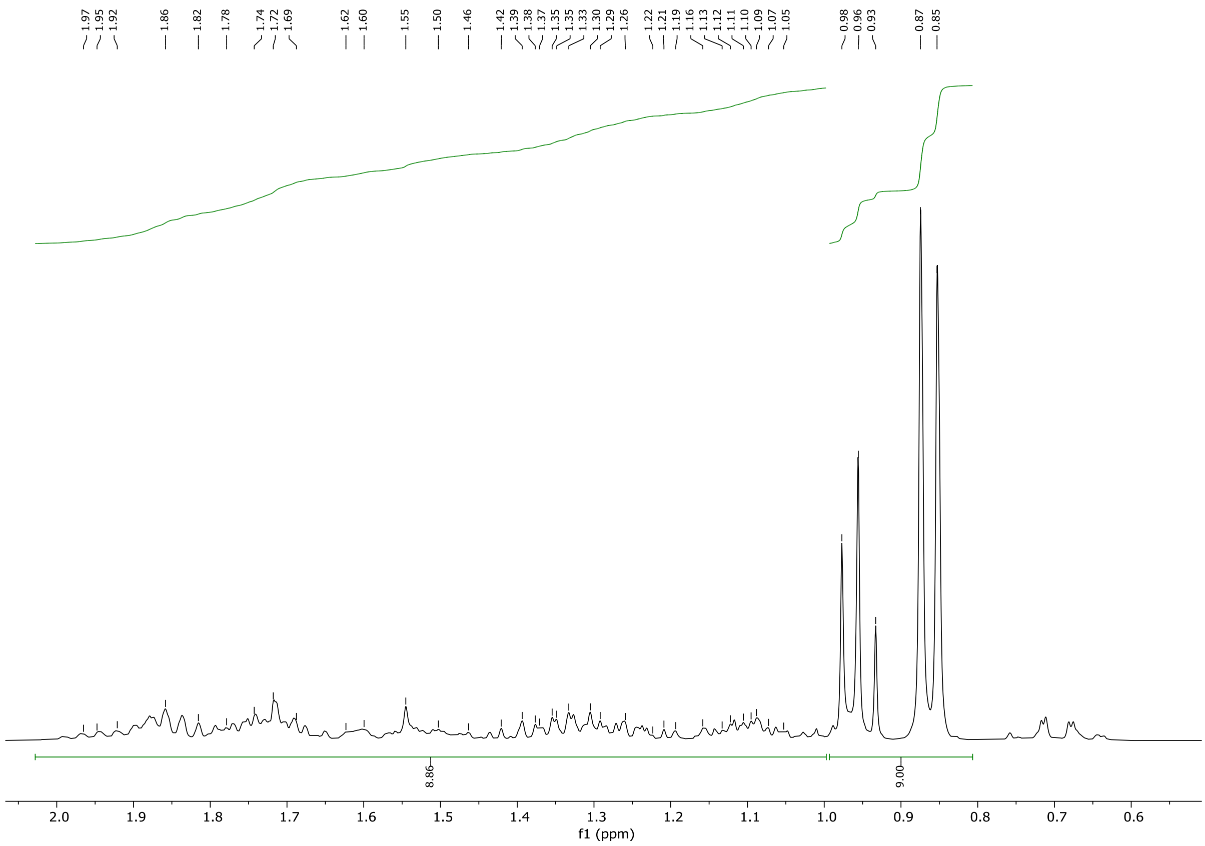

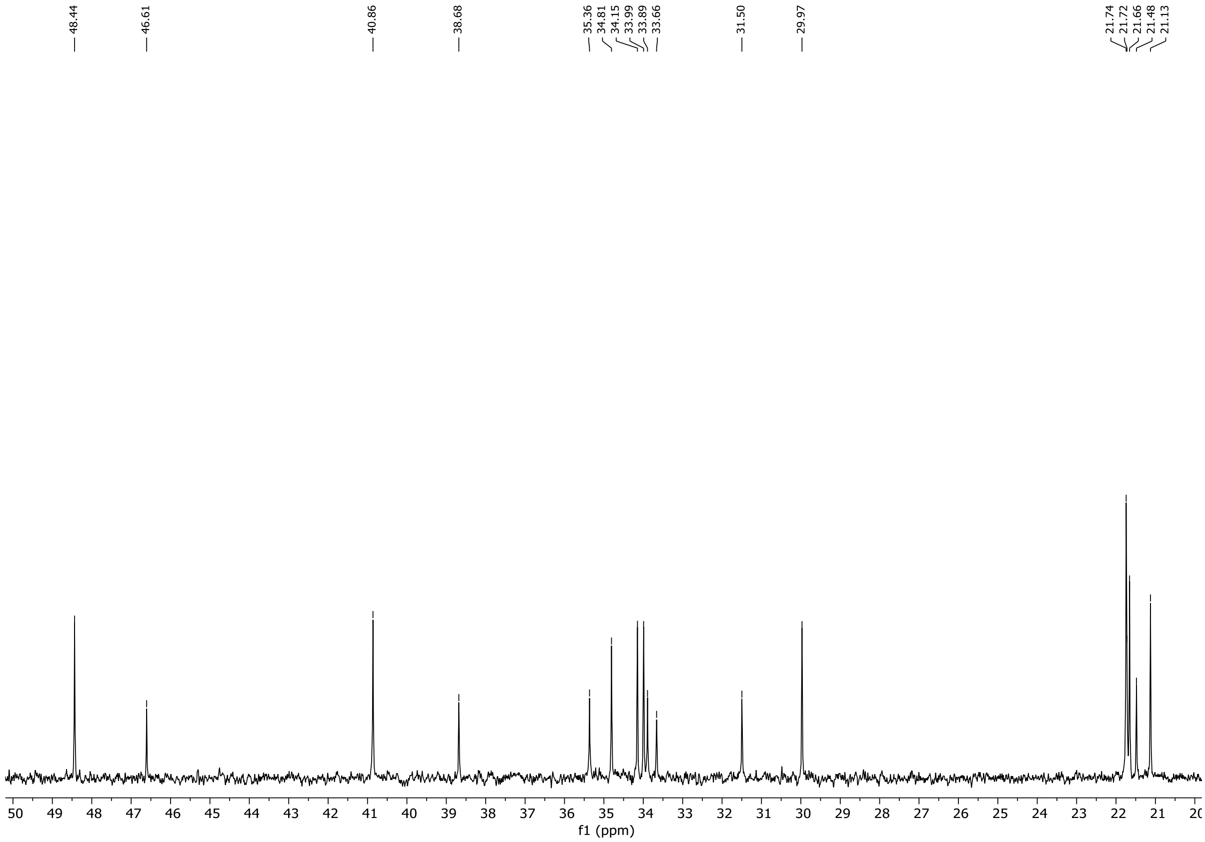

Figure S133. *^{1}H-and ^{13}C-NMR of **10c** (Zoomed in) [700 MHz/175 MHz, CDCl$_3$].*

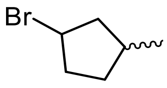

18

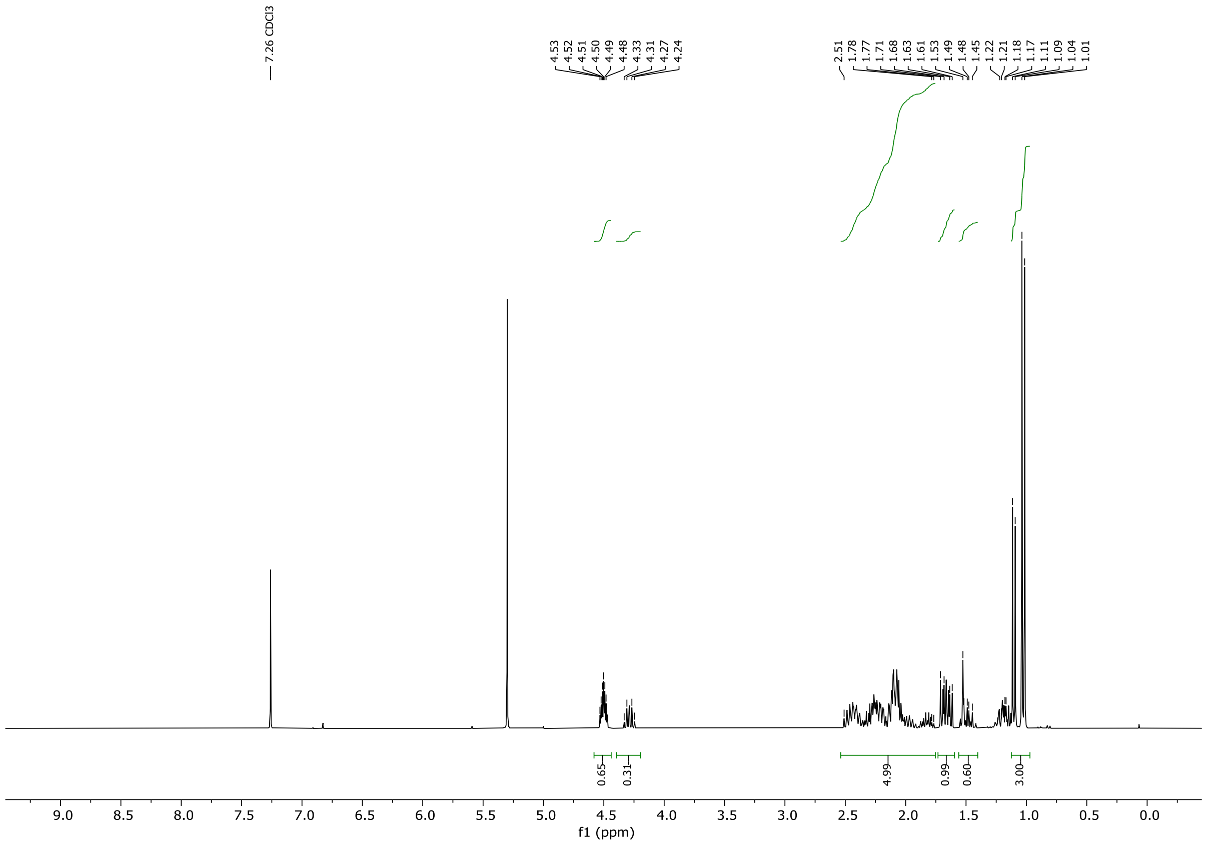

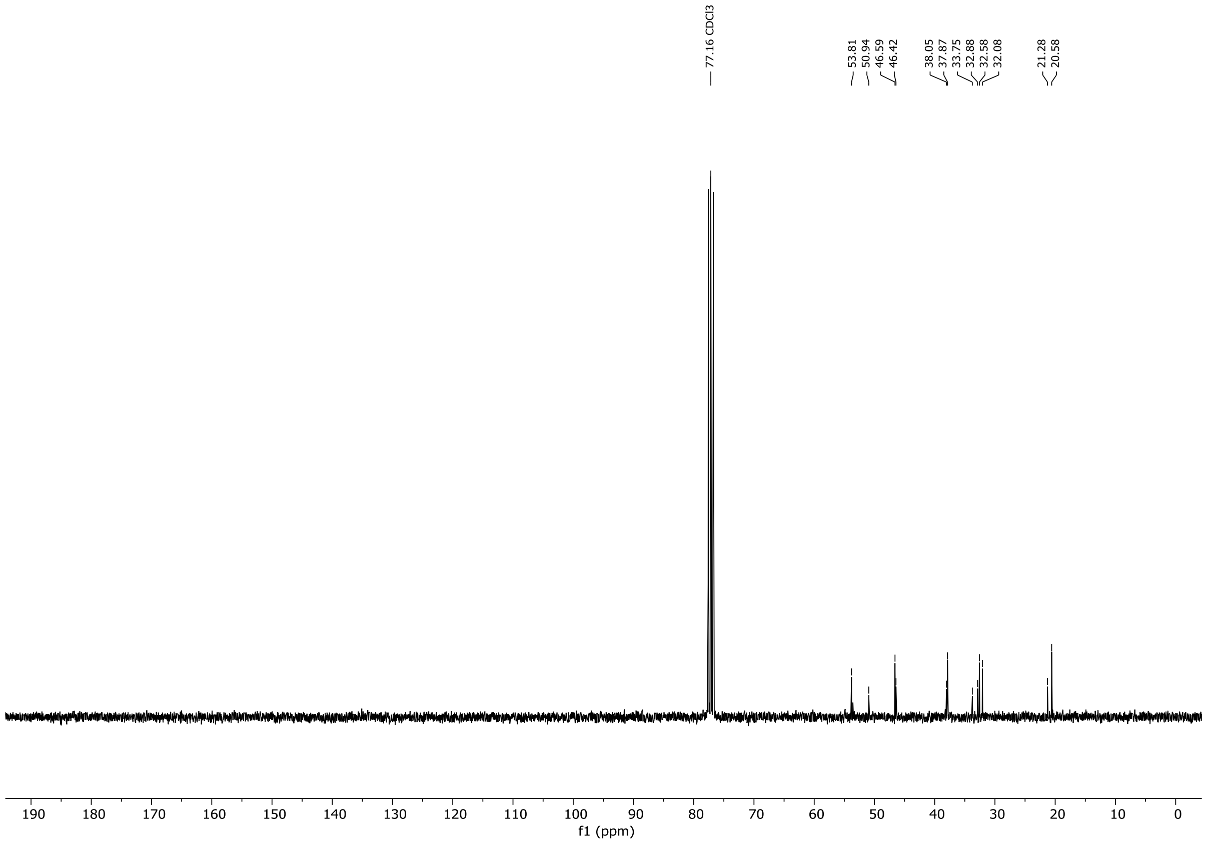

Figure S134. *^{1}H-and ^{13}C-NMR of **18** [300 MHz/75 MHz, $CDCl_3$].*

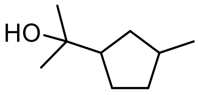

10d

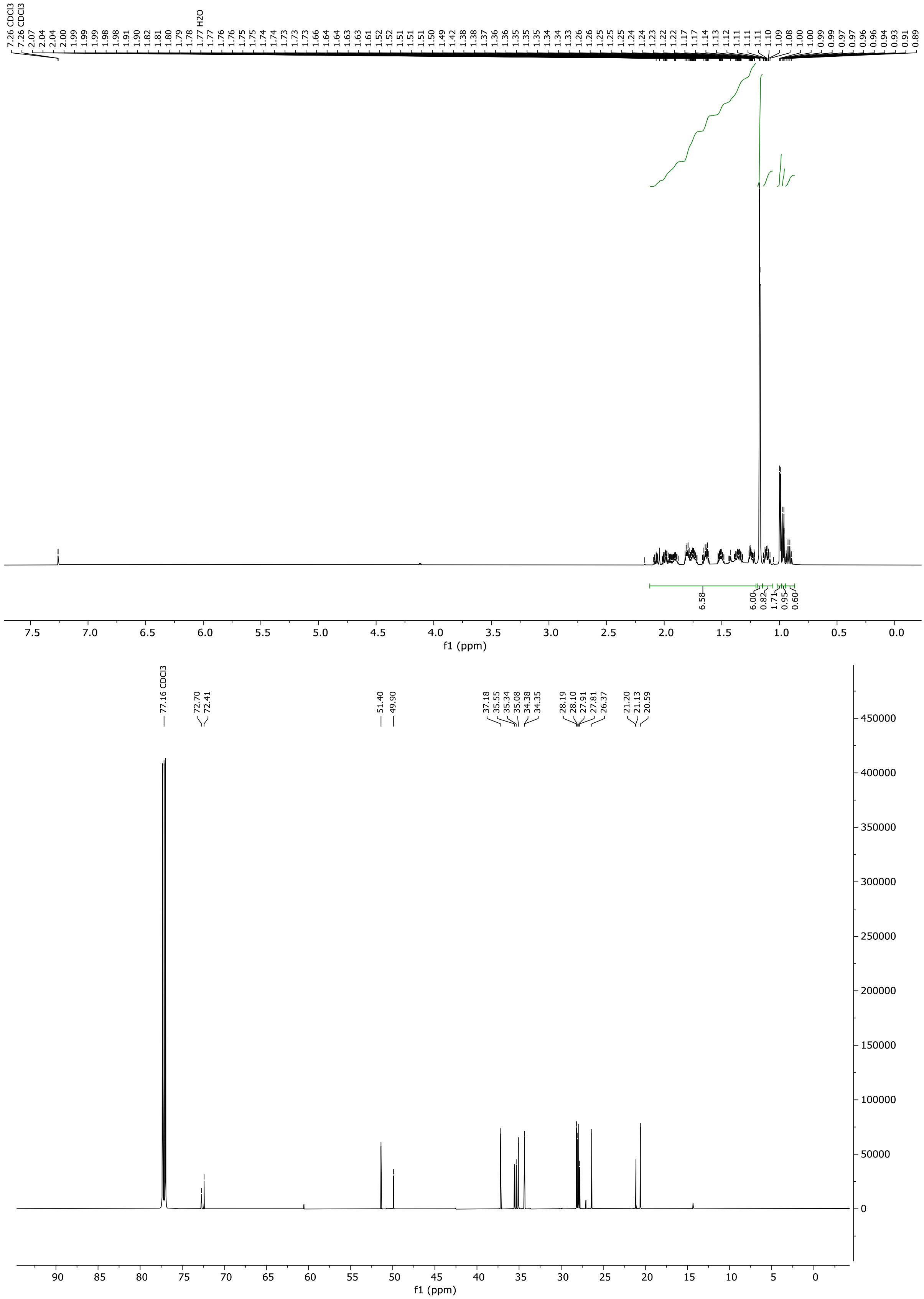

Figure S135. *^{1}H-and ^{13}C-NMR of **10d** [700 MHz/175 MHz, $CDCl_3$].*

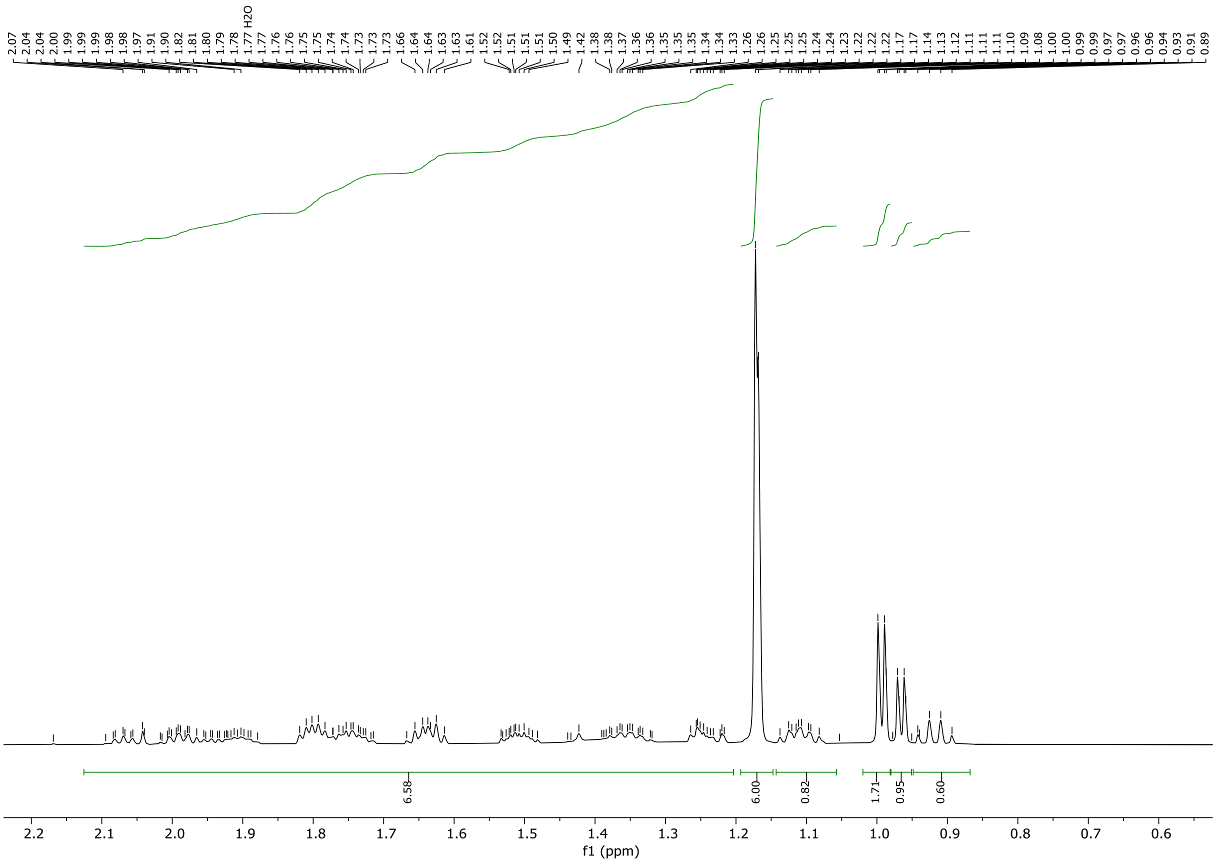

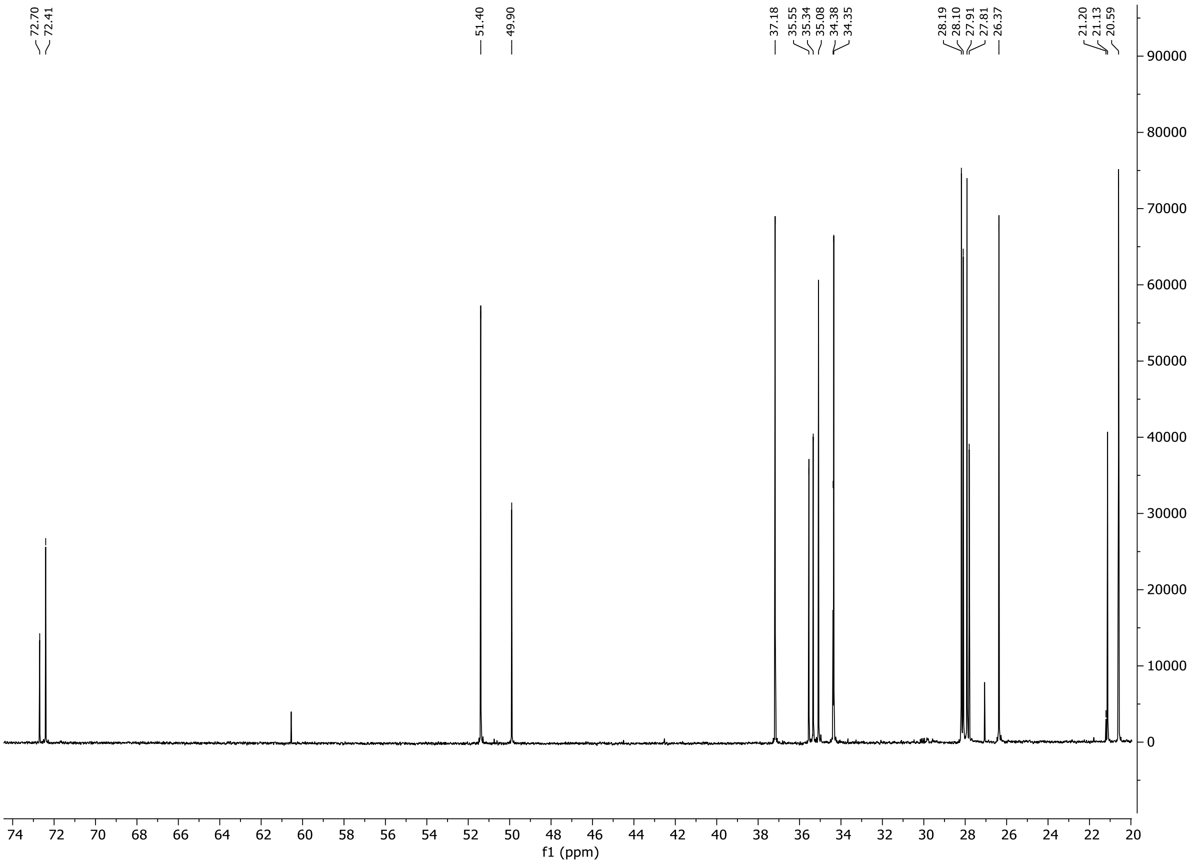

Figure S136. *^{1}H-and ^{13}C-NMR of **10d** (Zoomed in) [700 MHz/175 MHz, CDCl$_3$].*

9 Primers and Sequences

9.1 Primers

Table S31. *Primers used for the site directed and site-saturation mutagenesis of CvFAP.*

template	primer name	primer sequence
*Cv*FAP Wt	V453A-Fw	GGTTCGCTTC**GCT**CCAGGTATGGCG
	V453A-RP	TGCAGGTCCGGCAGCGCC
*Cv*FAP Wt	I398L-Fw	ATACGACGGT**CTC**GCCATTTCTG
	I398L-RP	TTTTCTTTAACCGGAGCC
*Cv*FAP Y466A	G462A-Fw	GGACCCGGAC**GCG**GTTAGCACC
	G462A-RP	AGCGCCATACCTGGAACG
	Y466X_NDT-Fw	TGTTAGCACC**NDT**GTTCGTTTTGCTAAATTCCAGAGCCAGGGTC
*Cv*FAP	Y466X_VHG-Fw	TGTTAGCACC**VHG**GTTCGTTTTGCTAAATTCCAGAGCCAGGGTC
Wt	Y466X_TGG-Fw	TGTTAGCACC**TGG**GTTCGTTTTGCTAAATTCCAGAGCCAGGGTC
	Y466X-RP	CCGTCCGGGTCCAGCGCC
	L386X_NDT-Fw	GCCGGCGTGC**NDT**ACCGCGGCTC
*Cv*FAP	L386X_VHG-Fw	GCCGGCGTGC**VHG**ACCGCGGCTC
Y466A	L386X_TGG-Fw	GCCGGCGTGC**TGG**ACCGCGGCTC
	L386X-RP	TGATCCTGCAGGTTCTGGCC
	I398X_NDT-Fw	AATACGACGGT**NDT**GCCATTTCTGATCAC
*Cv*FAP	I398X_VHG-Fw	AATACGACGGT**VHG**GCCATTTCTGATCAC
Y466A	I398X_TGG-Fw	AATACGACGGT**TGG**GCCATTTCTGATCAC
	I398X-RP	TTTCTTTAACCGGAGCCGC
	G431X_NDT-Fw	ACTTCCACC**NDT**TGCGATCGCGGTGCCTTC
*Cv*FAP	G431X_VHG-Fw	ACTTCCACC**VHG**TGCGATCGCGGTGCCTTC
Y466A	G431X_TGG-Fw	ACTTCCACC**TGG**TGCGATCGCGGTGCCTTC
	G431X-RP	CAGACCGCCACGACCACCCAG
	V453X_NDT-Fw	TGCAGGTTCGCTTC**NDT**CCAGGTATGGC
*Cv*FAP	V453X_VHG-Fw	TGCAGGTTCGCTTC**VHG**CCAGGTATGGC
Y466A	V453X_TGG-Fw	TGCAGGTTCGCTTC**TGG**CCAGGTATGGC
	V453X-RP	GGTCCGGCAGCGCCTG
	G462X_NDT-Fw	GGACCCGGAC**NDT**GTTAGCACC
*Cv*FAP	G462X_VHG-Fw	GGACCCGGAC**VHG**GTTAGCACC
Y466A	G462X_TGG-Fw	GGACCCGGAC**TGG**GTTAGCACC
	G462X -RP	AGCGCCATACCTGGAAC
	S574X_NDT-Fw	TATCCACTCG**NDT**AACGCTATCAC
*Cv*FAP	S574X_VHG-Fw	TATCCACTCG**VHG**AACGCTATCAC
Y466A	S574X_TGG-Fw	TATCCACTCG**TGG**AACGCTATCAC
	S574X -RP	GAACGACGGATATATTCATC
	F134X_NDT-Fw	ATCACCCGCCTG**NDT**CGCTCCCCGC
*Cv*FAP	F134X_VHG-Fw	ATCACCCGCCTG**VHG**CGCTCCCCGC
Y466A/V453S	F134X_TGG-Fw	ATCACCCGCCTG**TGG**CGCTCCCCGC
	F134X -RP	CGCCGCCGGAATCTTAACGTC
	G431X_NDT-Fw	ACTTCCACC**NDT**TGCGATCGCGGTGCCTTC
*Cv*FAP	G431X_VHG-Fw	ACTTCCACC**VHG**TGCGATCGCGGTGCCTTC
Y466A/V453S	G431X_TGG-Fw	ACTTCCACC**TGG**TGCGATCGCGGTGCCTTC
	G431X -RP	CAGACCGCCACGACCACCCAG
*Cv*FAP	A384X_NDT-Fw	ATCAGCCG**NDT**TGCCTGACCG
Y466A/V453S	A384X_VHG-Fw	ATCAGCCG**VHG**TGCCTGACCG

	A384X_TGG-Fw	ATCAGCCG**TGG**TGCCTGACCG
	A384X -RP	CCTGCAGGTTCTGGCCAACAC
*Cv*FAP Y466A/V453S	L386X_NDT-Fw	GCCGGCGTGC**NDT**ACCGCGGCTC
	L386X_VHG-Fw	GCCGGCGTGC**VHG**ACCGCGGCTC
	L386X_TGG-Fw	GCCGGCGTGC**TGG**ACCGCGGCTC
	L386X -RP	TGATCCTGCAGGTTCTGGCC
*Cv*FAP Y466A/V453S /G431S	I130X_NDT-Fw	GTTAAGATTCCGGCGGCG**NDT**ACCCGC
	I130X_VHG-Fw	GTTAAGATTCCGGCGGCG**VHG**ACCCGC
	I130X_TGG-Fw	GTTAAGATTCCGGCGGCG**TGG**ACCCGC
	I130X -RP	GTCGCGGGAGGTGTTATCCG
*Cv*FAP Y466A/V453S /G431S	C432X_NDT-Fw	TTCCACCAGT**NDT**GATCGCGGTG
	C432X_VHG-Fw	TTCCACCAGT**VHG**GATCGCGGTG
	C432X_TGG-Fw	TTCCACCAGT**TGG**GATCGCGGTG
	C432X -RP	GTCAGACCGCCACGACC

9.2 *Cv*FAP wild type construct

For expression of the fatty acid photodecarboxylase (*Cv*FAP) in *E. coli*, a gene was designed based on a reported construct[43], consisting of an *N*-terminal His6-tag, a thioredoxin (TrxA) tag, a tobacco etch virus (TEV) protease cleavage site and residues 62-654 of the *Cv*FAP gene, which is also His-tagged (GenBank: KY511411). The sequence was codon optimized for expression in *E. coli*. The construct was ordered from global biosystems, subcloned into a pET28a(+), vector flanked by the NdeI and HindIII restriction sites. Competent *E. coli* BL21 (DE3) cells (NEB) were transformed with the plasmid for recombinant enzyme production. The start codon is not visible here and is in the NcoI restriction site prior to the NdeI restriction site.[43,44]

- NdeI and HindIII restriction sites (**bold**)
- His6-tag
- Thioredoxin TrxA (Uniprot: P0AA25)
- TEV Protease
- Residue 62-654 of *Cv*FAP
- Stop Codon

CATATGAAATCTTCTCACCATCACCATCACCATGGTTCTTCTATGAGCGATAAAATTATTCACCTGACTGACGAC
AGTTTTGACACGGATGTACTCAAAGCGGACGGGGCGATCCTCGTCGATTTCTGGGCAGAGTGGTGCGGTCCGT
GCAAAATGATCGCCCCGATTCTGGATGAAATCGCTGACGAATATCAGGGCAAACTGACCGTTGCAAAACTGAA
CATCGATCAAAACCCTGGCACTGCGCCGAAATATGGCATCCGTGGTATCCCGACTCTGCTGCTGTTCAAAAACG
GTGAAGTGGCGGCAACCAAAGTGGGTGCACTGTCTAAAGGTCAGTTGAAAGAGTTCCTCGACGCTAACCTGG
CCGGGATCGAGGAAAACCTGTACTTCCAATCCGCGTCTGCCGTTGAAGACATCCGTAAAGTCCTGTCCGATTCT
TCGTCTCCGGTGGCGGGTCAGAAATATGACTACATCCTGGTTGGCGGTGGCACCGCGGCGTGCGTGCTGGCA
AACCGTCTGAGCGCTGACGGTTCCAAACGTGTACTGGTTCTGGAAGCAGGCCCGGATAACACCTCCCGCGACG

TTAAGATTCCGGCGGCGATCACCCGCCTGTTCCGCTCCCCGCTGGACTGGAACCTGTTCTCTGAACTGCAGGAA

CAGCTTGCGGAACGTCAGATCTACATGGCGCGTGGCCGTCTGCTGGGCGGTTCCAGCGCGACTAACGCCACTC

TGTACCACCGTGGTGCGGCGGGTGATTACGACGCATGGGGTGTTGAAGGCTGGTCCAGCGAAGACGTTCTGT

CTTGGTTCGTCCAGGCGGAAACCAACGCGGACTTCGGTCCGGGCGCTTATCATGGCAGCGGCGGCCCGATGC

GTGTGGAAAACCCGCGTTACACCAACAAACAGCTGCACACTGCTTTCTTCAAGGCTGCTGAAGAAGTTGGTCTT

ACCCCGAACTCCGATTTCAACGATTGGAGCCATGACCACGCCGGTTACGGCACCTTTCAGGTGATGCAGGATA

AAGGCACCCGCGCGGATATGTACCGTCAGTATCTGAAACCTGTGCTGGGTCGTCGCAACCTGCAGGTACTGAC

CGGCGCTGCAGTGACCAAAGTCAACATCGACCAGGCTGCGGGCAAAGCGCAGGCTCTGGGTGTTGAATTCTC

CACCGACGGCCCAACCGGCGAACGCCTGTCTGCGGAACTGGCTCCGGGTGGTGAGGTCATCATGTGCGCAGG

TGCTGTTCACACCCCGTTCCTGCTGAAACATTCCGGCGTTGGCCCGTCTGCTGAGCTGAAAGAATTCGGCATCC

CGGTTGTTAGCAACCTGGCTGGTGTTGGCCAGAACCTGCAGGATCAGCCGGCGTGCCTGACCGCGGCTCCGGT

TAAAGAAAAATACGACGGTATTGCCATTTCTGATCACATCTACAACGAAAAAGGCCAGATCCGTAAACGTGCA

ATCGCATCCTACCTGCTGGGTGGTCGTGGCGGTCTGACTTCCACCGGTTGCGATCGCGGTGCCTTCGTTCGTAC

CGCGGGTCAGGCGCTGCCGGACCTGCAGGTTCGCTTCGTTCCAGGTATGGCGCTGGACCCGGACGGTGTTAG

CACCTACGTTCGTTTTGCTAAATTCCAGAGCCAGGGTCTGAAATGGCCGAGCGGCATCACCATGCAGCTGATC

GCTTGCCGTCCGCAGTCTACCGGCTCCGTCGGTCTTAAATCCGCTGACCCGTTTGCGCCGCCGAAACTGTCACC

AGGTTACCTGACCGACAAAGACGGTGCTGATCTGGCTACCCTGCGTAAAGGCATCCATTGGGCACGTGATGTT

GCGCGTAGCTCTGCTCTGTCCGAATACCTGGATGGTGAGCTGTTCCCAGGTAGCGGCGTTGTTTCTGATGATCA

GATCGATGAATATATCCGTCGTTCTATCCACTCGTCCAACGCTATCACTGGCACCTGTAAAATGGGTAACGCAG

GTGACAGCAGCTCTGTGGTAGACAACCAGCTGCGTGTTCACGGTGTTGAAGGCCTGCGCGTTGTTGACGCTAG

CGTTGTTCCGAAAATTCCGGGTGGTCAGACCGGTGCGCCGGTAGTTATGATCGCTGAACGCGCAGCAGCTCTG

CTGACGGGGAAAGCAACCATTGGTGCATCTGCTGCTGCACCGGCGACCGTAGCTGCATAA**AAGCTT**

9.3 *Cv*FAP Wt

GCGTCTGCCGTTGAAGACATCCGTAAAGTCCTGTCCGATTCTTCGTCTCCGGTGGCGGGTCAGAAATATGACTA
CATCCTGGTTGGCGGTGGCACCGCGGCGTGCGTGCTGGCAAACCGTCTGAGCGCTGACGGTTCCAAACGTGT
ACTGGTTCTGGAAGCAGGCCCGGATAACACCTCCCGCGACGTTAAGATTCCGGCGGCGATCACCCGCCTGTTC
CGCTCCCCGCTGGACTGGAACCTGTTCTCTGAACTGCAGGAACAGCTTGCGGAACGTCAGATCTACATGGCGC
GTGGCCGTCTGCTGGGCGGTTCCAGCGCGACTAACGCCACTCTGTACCACCGTGGTGCGGCGGGTGATTACGA
CGCATGGGGTGTTGAAGGCTGGTCCAGCGAAGACGTTCTGTCTTGGTTCGTCCAGGCGGAAACCAACGCGGA
CTTCGGTCCGGGCGCTTATCATGGCAGCGGCGGCCCGATGCGTGTGGAAAACCCGCGTTACACCAACAAACA
GCTGCACACTGCTTTCTTCAAGGCTGCTGAAGAAGTTGGTCTTACCCCGAACTCCGATTTCAACGATTGGAGCC
ATGACCACGCCGGTTACGGCACCTTTCAGGTGATGCAGGATAAAGGCACCCGCGCGGATATGTACCGTCAGTA
TCTGAAACCTGTGCTGGGTCGTCGCAACCTGCAGGTACTGACCGGCGCTGCAGTGACCAAAGTCAACATCGAC
CAGGCTGCGGGCAAAGCGCAGGCTCTGGGTGTTGAATTCTCCACCGACGGCCCAACCGGCGAACGCCTGTCT
GCGGAACTGGCTCCGGGTGGTGAGGTCATCATGTGCGCAGGTGCTGTTCACACCCCGTTCCTGCTGAAACATT
CCGGCGTTGGCCCGTCTGCTGAGCTGAAAGAATTCGGCATCCCGGTTGTTAGCAACCTGGCTGGTGTTGGCCA
GAACCTGCAGGATCAGCCGGCGTGCCTGACCGCGGCTCCGGTTAAAGAAAAATACGACGGTATTGCCATTTCT
GATCACATCTACAACGAAAAAGGCCAGATCCGTAAACGTGCAATCGCATCCTACCTGCTGGGTGGTCGTGGCG
GTCTGACTTCCACCGGTTGCGATCGCGGTGCCTTCGTTCGTACCGCGGGTCAGGCGCTGCCGGACCTGCAGGT
TCGCTTCGTTCCAGGTATGGCGCTGGACCCGGACGGTGTTAGCACCTACGTTCGTTTTGCTAAATTCCAGAGCC
AGGGTCTGAAATGGCCGAGCGGCATCACCATGCAGCTGATCGCTTGCCGTCCGCAGTCTACCGGCTCCGTCGG
TCTTAAATCCGCTGACCCGTTTGCGCCGCCGAAACTGTCACCAGGTTACCTGACCGACAAAGACGGTGCTGATC
TGGCTACCCTGCGTAAAGGCATCCATTGGGCACGTGATGTTGCGCGTAGCTCTGCTCTGTCCGAATACCTGGAT
GGTGAGCTGTTCCCAGGTAGCGGCGTTGTTTCTGATGATCAGATCGATGAATATATCCGTCGTTCTATCCACTC
GTCCAACGCTATCACTGGCACCTGTAAAATGGGTAACGCAGGTGACAGCAGCTCTGTGGTAGACAACCAGCTG
CGTGTTCACGGTGTTGAAGGCCTGCGCGTTGTTGACGCTAGCGTTGTTCCGAAAATTCCGGGTGGTCAGACCG
GTGCGCCGGTAGTTATGATCGCTGAACGCGCAGCAGCTCTGCTGACGGGGAAAGCAACCATTGGTGCATCTG
CTGCTGCACCGGCGACCGTAGCTGCATAA

ASAVEDIRKVLSDSSSPVAGQKYDYILVGGGTAACVLANRLSADGSKRVLVLEAGPDNTSRDVKIPAAITRLFRSPLD
WNLFSELQEQLAERQIYMARGRLLGGSSATNATLYHRGAAGDYDAWGVEGWSSEDVLSWFVQAETNADFGPGA
YHGSGGPMRVENPRYTNKQLHTAFFKAAEEVGLTPNSDFNDWSHDHAGYGTFQVMQDKGTRADMYRQYLKPV
LGRRNLQVLTGAAVTKVNIDQAAGKAQALGVEFSTDGPTGERLSAELAPGGEVIMCAGAVHTPFLLKHSGVGPSAE
LKEFGIPVVSNLAGVGQNLQDQPACLTAAPVKEKYDGIAISDHIYNEKGQIRKRAIASYLLGGRGGLTSTGCDRGAFV
RTAGQALPDLQVRFVPGMALDPDGVSTYVRFAKFQSQGLKWPSGITMQLIACRPQSTGSVGLKSADPFAPPKLSP
GYLTDKDGADLATLRKGIHWARDVARSSALSEYLDGELFPGSGVVSDDQIDEYIRRSIHSSNAITGTCKMGNAGDSS
SVVDNQLRVHGVEGLRVVDASVVPKIPGGQTGAPVVMIAERAAALLTGKATIGASAAAPATVAA-

9.4 V453A

GCGTCTGCCGTTGAAGACATCCGTAAAGTCCTGTCCGATTCTTCGTCTCCGGTGGCGGGTCAGAAATATGACTA
CATCCTGGTTGGCGGTGGCACCGCGGCGTGCGTGCTGGCAAACCGTCTGAGCGCTGACGGTTCCAAACGTGT
ACTGGTTCTGGAAGCAGGCCCGGATAACACCTCCCGCGACGTTAAGATTCCGGCGGCGATCACCCGCCTGTTC
CGCTCCCCGCTGGACTGGAACCTGTTCTCTGAACTGCAGGAACAGCTTGCGGAACGTCAGATCTACATGGCGC
GTGGCCGTCTGCTGGGCGGTTCCAGCGCGACTAACGCCACTCTGTACCACCGTGGTGCGGCGGGTGATTACGA
CGCATGGGGTGTTGAAGGCTGGTCCAGCGAAGACGTTCTGTCTTGGTTCGTCCAGGCGGAAACCAACGCGGA
CTTCGGTCCGGGCGCTTATCATGGCAGCGGCGGCCCGATGCGTGTGGAAAACCCGCGTTACACCAACAAACA
GCTGCACACTGCTTTCTTCAAGGCTGCTGAAGAAGTTGGTCTTACCCCGAACTCCGATTTCAACGATTGGAGCC
ATGACCACGCCGGTTACGGCACCTTTCAGGTGATGCAGGATAAAGGCACCCGCGCGGATATGTACCGTCAGTA
TCTGAAACCTGTGCTGGGTCGTCGCAACCTGCAGGTACTGACCGGCGCTGCAGTGACCAAAGTCAACATCGAC
CAGGCTGCGGGCAAAGCGCAGGCTCTGGGTGTTGAATTCTCCACCGACGGCCCAACCGGCGAACGCCTGTCT
GCGGAACTGGCTCCGGGTGGTGAGGTCATCATGTGCGCAGGTGCTGTTCACACCCCGTTCCTGCTGAAACATT
CCGGCGTTGGCCCGTCTGCTGAGCTGAAAGAATTCGGCATCCCGGTTGTTAGCAACCTGGCTGGTGTTGGCCA
GAACCTGCAGGATCAGCCGGCGTGCCTGACCGCGGCTCCGGTTAAAGAAAAATACGACGGTATTGCCATTTCT
GATCACATCTACAACGAAAAAGGCCAGATCCGTAAACGTGCAATCGCATCCTACCTGCTGGGTGGTCGTGGCG
GTCTGACTTCCACCGGTTGCGATCGCGGTGCCTTCGTTCGTACCGCGGGTCAGGCGCTGCCGGACCTGCAGGT
TCGCTTCGCGCCAGGTATGGCGCTGGACCCGGACGGTGTTAGCACCTACGTTCGTTTTGCTAAATTCCAGAGCC
AGGGTCTGAAATGGCCGAGCGGCATCACCATGCAGCTGATCGCTTGCCGTCCGCAGTCTACCGGCTCCGTCGG
TCTTAAATCCGCTGACCCGTTTGCGCCGCCGAAACTGTCACCAGGTTACCTGACCGACAAAGACGGTGCTGATC
TGGCTACCCTGCGTAAAGGCATCCATTGGGCACGTGATGTTGCGCGTAGCTCTGCTCTGTCCGAATACCTGGAT
GGTGAGCTGTTCCCAGGTAGCGGCGTTGTTTCTGATGATCAGATCGATGAATATATCCGTCGTTCTATCCACTC
GTCCAACGCTATCACTGGCACCTGTAAAATGGGTAACGCAGGTGACAGCAGCTCTGTGGTAGACAACCAGCTG
CGTGTTCACGGTGTTGAAGGCCTGCGCGTTGTTGACGCTAGCGTTGTTCCGAAAATTCCGGGTGGTCAGACCG
GTGCGCCGGTAGTTATGATCGCTGAACGCGCAGCAGCTCTGCTGACGGGGAAAGCAACCATTGGTGCATCTG
CTGCTGCACCGGCGACCGTAGCTGCATAA

ASAVEDIRKVLSDSSSPVAGQKYDYILVGGGTAACVLANRLSADGSKRVLVLEAGPDNTSRDVKIPAAITRLFRSPLD
WNLFSELQEQLAERQIYMARGRLLGGSSATNATLYHRGAAGDYDAWGVEGWSSEDVLSWFVQAETNADFGPGA
YHGSGGPMRVENPRYTNKQLHTAFFKAAEEVGLTPNSDFNDWSHDHAGYGTFQVMQDKGTRADMYRQYLKPV
LGRRNLQVLTGAAVTKVNIDQAAGKAQALGVEFSTDGPTGERLSAELAPGGEVIMCAGAVHTPFLLKHSGVGPSAE
LKEFGIPVVSNLAGVGQNLQDQPACLTAAPVKEKYDGIAISDHIYNEKGQIRKRAIASYLLGGRGGLTSTGCDRGAFV
RTAGQALPDLQVRF**A**PGMALDPDGVSTYVRFAKFQSQGLKWPSGITMQLIACRPQSTGSVGLKSADPFAPPKLSP
GYLTDKDGADLATLRKGIHWARDVARSSALSEYLDGELFPGSGVVSDDQIDEYIRRSIHSSNAITGTCKMGNAGDSS
SVVDNQLRVHGVEGLRVVDASVVPKIPGGQTGAPVVMIAERAAALLTGKATIGASAAAPATVAA-

9.5 Y466A

GCGTCTGCCGTTGAAGACATCCGTAAAGTCCTGTCCGATTCTTCGTCTCCGGTGGCGGGTCAGAAATATGACTA
CATCCTGGTTGGCGGTGGCACCGCGGCGTGCGTGCTGGCAAACCGTCTGAGCGCTGACGGTTCCAAACGTGT
ACTGGTTCTGGAAGCAGGCCCGGATAACACCTCCCGCGACGTTAAGATTCCGGCGGCGATCACCCGCCTGTTC
CGCTCCCCGCTGGACTGGAACCTGTTCTCTGAACTGCAGGAACAGCTTGCGGAACGTCAGATCTACATGGCGC
GTGGCCGTCTGCTGGGCGGTTCCAGCGCGACTAACGCCACTCTGTACCACCGTGGTGCGGCGGGTGATTACGA
CGCATGGGGTGTTGAAGGCTGGTCCAGCGAAGACGTTCTGTCTTGGTTCGTCCAGGCGGAAACCAACGCGGA
CTTCGGTCCGGGCGCTTATCATGGCAGCGGCGGCCCGATGCGTGTGGAAAACCCGCGTTACACCAACAAACA
GCTGCACACTGCTTTCTTCAAGGCTGCTGAAGAAGTTGGTCTTACCCCGAACTCCGATTTCAACGATTGGAGCC
ATGACCACGCCGGTTACGGCACCTTTCAGGTGATGCAGGATAAAGGCACCCGCGCGGATATGTACCGTCAGTA
TCTGAAACCTGTGCTGGGTCGTCGCAACCTGCAGGTACTGACCGGCGCTGCAGTGACCAAAGTCAACATCGAC
CAGGCTGCGGGCAAAGCGCAGGCTCTGGGTGTTGAATTCTCCACCGACGGCCCAACCGGCGAACGCCTGTCT
GCGGAACTGGCTCCGGGTGGTGAGGTCATCATGTGCGCAGGTGCTGTTCACACCCCGTTCCTGCTGAAACATT
CCGGCGTTGGCCCGTCTGCTGAGCTGAAAGAATTCGGCATCCCGGTTGTTAGCAACCTGGCTGGTGTTGGCCA
GAACCTGCAGGATCAGCCGGCGTGCCTGACCGCGGCTCCGGTTAAAGAAAAATACGACGGTATTGCCATTTCT
GATCACATCTACAACGAAAAAGGCCAGATCCGTAAACGTGCAATCGCATCCTACCTGCTGGGTGGTCGTGGCG
GTCTGACTTCCACCGGTTGCGATCGCGGTGCCTTCGTTCGTACCGCGGGTCAGGCGCTGCCGGACCTGCAGGT
TCGCTTCGTTCCAGGTATGGCGCTGGACCCGGACGGTGTTAGCACCGCGGTTCGTTTTGCTAAATTCCAGAGCC
AGGGTCTGAAATGGCCGAGCGGCATCACCATGCAGCTGATCGCTTGCCGTCCGCAGTCTACCGGCTCCGTCGG
TCTTAAATCCGCTGACCCGTTTGCGCCGCCGAAACTGTCACCAGGTTACCTGACCGACAAAGACGGTGCTGATC
TGGCTACCCTGCGTAAAGGCATCCATTGGGCACGTGATGTTGCGCGTAGCTCTGCTCTGTCCGAATACCTGGAT
GGTGAGCTGTTCCCAGGTAGCGGCGTTGTTTCTGATGATCAGATCGATGAATATATCCGTCGTTCTATCCACTC
GTCCAACGCTATCACTGGCACCTGTAAAATGGGTAACGCAGGTGACAGCAGCTCTGTGGTAGACAACCAGCTG
CGTGTTCACGGTGTTGAAGGCCTGCGCGTTGTTGACGCTAGCGTTGTTCCGAAAATTCCGGGTGGTCAGACCG
GTGCGCCGGTAGTTATGATCGCTGAACGCGCAGCAGCTCTGCTGACGGGGAAAGCAACCATTGGTGCATCTG
CTGCTGCACCGGCGACCGTAGCTGCATAA

ASAVEDIRKVLSDSSSPVAGQKYDYILVGGGTAACVLANRLSADGSKRVLVLEAGPDNTSRDVKIPAAITRLFRSPLD
WNLFSELQEQLAERQIYMARGRLLGGSSATNATLYHRGAAGDYDAWGVEGWSSEDVLSWFVQAETNADFGPGA
YHGSGGPMRVENPRYTNKQLHTAFFKAAEEVGLTPNSDFNDWSHDHAGYGTFQVMQDKGTRADMYRQYLKPV
LGRRNLQVLTGAAVTKVNIDQAAGKAQALGVEFSTDGPTGERLSAELAPGGEVIMCAGAVHTPFLLKHSGVGPSAE
LKEFGIPVVSNLAGVGQNLQDQPACLTAAPVKEKYDGIAISDHIYNEKGQIRKRAIASYLLGGRGGLTSTGCDRGAFV
RTAGQALPDLQVRFVPGMALDPDGVST**A**VRFAKFQSQGLKWPSGITMQLIACRPQSTGSVGLKSADPFAPPKLSP
GYLTDKDGADLATLRKGIHWARDVARSSALSEYLDGELFPGSGVVSDDQIDEYIRRSIHSSNAITGTCKMGNAGDSS
SVVDNQLRVHGVEGLRVVDASVVPKIPGGQTGAPVVMIAERAAALLTGKATIGASAAAPATVAA-

9.6 Y466A/V453S:

GCGTCTGCCGTTGAAGACATCCGTAAAGTCCTGTCCGATTCTTCGTCTCCGGTGGCGGGTCAGAAATATGACTA
CATCCTGGTTGGCGGTGGCACCGCGGCGTGCGTGCTGGCAAACCGTCTGAGCGCTGACGGTTCCAAACGTGT
ACTGGTTCTGGAAGCAGGCCCGGATAACACCTCCCGCGACGTTAAGATTCCGGCGGCGATCACCCGCCTGTTC
CGCTCCCCGCTGGACTGGAACCTGTTCTCTGAACTGCAGGAACAGCTTGCGGAACGTCAGATCTACATGGCGC
GTGGCCGTCTGCTGGGCGGTTCCAGCGCGACTAACGCCACTCTGTACCACCGTGGTGCGGCGGGTGATTACGA
CGCATGGGGTGTTGAAGGCTGGTCCAGCGAAGACGTTCTGTCTTGGTTCGTCCAGGCGGAAACCAACGCGGA
CTTCGGTCCGGGCGCTTATCATGGCAGCGGCGGCCCGATGCGTGTGGAAAACCCGCGTTACACCAACAAACA
GCTGCACACTGCTTTCTTCAAGGCTGCTGAAGAAGTTGGTCTTACCCCGAACTCCGATTTCAACGATTGGAGCC
ATGACCACGCCGGTTACGGCACCTTTCAGGTGATGCAGGATAAAGGCACCCGCGCGGATATGTACCGTCAGTA
TCTGAAACCTGTGCTGGGTCGTCGCAACCTGCAGGTACTGACCGGCGCTGCAGTGACCAAAGTCAACATCGAC
CAGGCTGCGGGCAAAGCGCAGGCTCTGGGTGTTGAATTCTCCACCGACGGCCCAACCGGCGAACGCCTGTCT
GCGGAACTGGCTCCGGGTGGTGAGGTCATCATGTGCGCAGGTGCTGTTCACACCCCGTTCCTGCTGAAACATT
CCGGCGTTGGCCCGTCTGCTGAGCTGAAAGAATTCGGCATCCCGGTTGTTAGCAACCTGGCTGGTGTTGGCCA
GAACCTGCAGGATCAGCCGGCGTGCCTGACCGCGGCTCCGGTTAAAGAAAAATACGACGGTATTGCCATTTCT
GATCACATCTACAACGAAAAAGGCCAGATCCGTAAACGTGCAATCGCATCCTACCTGCTGGGTGGTCGTGGCG
GTCTGACTTCCACCGGTTGCGATCGCGGTGCCTTCGTTCGTACCGCGGGTCAGGCGCTGCCGGACCTGCAGGT
TCGCTTCAGTCCAGGTATGGCGCTGGACCCGGACGGTGTTAGCACCGCGGTTCGTTTTGCTAAATTCCAGAGC
CAGGGTCTGAAATGGCCGAGCGGCATCACCATGCAGCTGATCGCTTGCCGTCCGCAGTCTACCGGCTCCGTCG
GTCTTAAATCCGCTGACCCGTTTGCGCCGCCGAAACTGTCACCAGGTTACCTGACCGACAAAGACGGTGCTGA
TCTGGCTACCCTGCGTAAAGGCATCCATTGGGCACGTGATGTTGCGCGTAGCTCTGCTCTGTCCGAATACCTGG
ATGGTGAGCTGTTCCCAGGTAGCGGCGTTGTTTCTGATGATCAGATCGATGAATATATCCGTCGTTCTATCCAC
TCGTCCAACGCTATCACTGGCACCTGTAAAATGGGTAACGCAGGTGACAGCAGCTCTGTGGTAGACAACCAGC
TGCGTGTTCACGGTGTTGAAGGCCTGCGCGTTGTTGACGCTAGCGTTGTTCCGAAAATTCCGGGTGGTCAGAC
CGGTGCGCCGGTAGTTATGATCGCTGAACGCGCAGCAGCTCTGCTGACGGGGAAAGCAACCATTGGTGCATC
TGCTGCTGCACCGGCGACCGTAGCTGCATAA

ASAVEDIRKVLSDSSSPVAGQKYDYILVGGGTAACVLANRLSADGSKRVLVLEAGPDNTSRDVKIPAAITRLFRSPLD
WNLFSELQEQLAERQIYMARGRLLGGSSATNATLYHRGAAGDYDAWGVEGWSSEDVLSWFVQAETNADFGPGA
YHGSGGPMRVENPRYTNKQLHTAFFKAAEEVGLTPNSDFNDWSHDHAGYGTFQVMQDKGTRADMYRQYLKPV
LGRRNLQVLTGAAVTKVNIDQAAGKAQALGVEFSTDGPTGERLSAELAPGGEVIMCAGAVHTPFLLKHSGVGPSAE
LKEFGIPVVSNLAGVGQNLQDQPACLTAAPVKEKYDGIAISDHIYNEKGQIRKRAIASYLLGGRGGLTSTGCDRGAFV
RTAGQALPDLQVRF**S**PGMALDPDGVSTAVRFAKFQSQGLKWPSGITMQLIACRPQSTGSVGLKSADPFAPPKLSP
GYLTDKDGADLATLRKGIHWARDVARSSALSEYLDGELFPGSGVVSDDQIDEYIRRSIHSSNAITGTCKMGNAGDSS
SVVDNQLRVHGVEGLRVVDASVVPKIPGGQTGAPVVMIAERAAALLTGKATIGASAAAPATVAA-

9.7 Y466A/C432A

GCGTCTGCCGTTGAAGACATCCGTAAAGTCCTGTCCGATTCTTCGTCTCCGGTGGCGGGTCAGAAATATGACTA
CATCCTGGTTGGCGGTGGCACCGCGGCGTGCGTGCTGGCAAACCGTCTGAGCGCTGACGGTTCCAAACGTGT
ACTGGTTCTGGAAGCAGGCCCGGATAACACCTCCCGCGACGTTAAGATTCCGGCGGCGATCACCCGCCTGTTC
CGCTCCCCGCTGGACTGGAACCTGTTCTCTGAACTGCAGGAACAGCTTGCGGAACGTCAGATCTACATGGCGC
GTGGCCGTCTGCTGGGCGGTTCCAGCGCGACTAACGCCACTCTGTACCACCGTGGTGCGGCGGGTGATTACGA
CGCATGGGGTGTTGAAGGCTGGTCCAGCGAAGACGTTCTGTCTTGGTTCGTCCAGGCGGAAACCAACGCGGA
CTTCGGTCCGGGCGCTTATCATGGCAGCGGCGGCCCGATGCGTGTGGAAAACCCGCGTTACACCAACAAACA
GCTGCACACTGCTTTCTTCAAGGCTGCTGAAGAAGTTGGTCTTACCCCGAACTCCGATTTCAACGATTGGAGCC
ATGACCACGCCGGTTACGGCACCTTTCAGGTGATGCAGGATAAAGGCACCCGCGCGGATATGTACCGTCAGTA
TCTGAAACCTGTGCTGGGTCGTCGCAACCTGCAGGTACTGACCGGCGCTGCAGTGACCAAAGTCAACATCGAC
CAGGCTGCGGGCAAAGCGCAGGCTCTGGGTGTTGAATTCTCCACCGACGGCCCAACCGGCGAACGCCTGTCT
GCGGAACTGGCTCCGGGTGGTGAGGTCATCATGTGCGCAGGTGCTGTTCACACCCCGTTCCTGCTGAAACATT
CCGGCGTTGGCCCGTCTGCTGAGCTGAAAGAATTCGGCATCCCGGTTGTTAGCAACCTGGCTGGTGTTGGCCA
GAACCTGCAGGATCAGCCGGCGTGCCTGACCGCGGCTCCGGTTAAAGAAAAATACGACGGTATTGCCATTTCT
GATCACATCTACAACGAAAAAGGCCAGATCCGTAAACGTGCAATCGCATCCTACCTGCTGGGTGGTCGTGGCG
GTCTGACTTCCACCGGTGCGGATCGCGGTGCCTTCGTTCGTACCGCGGGTCAGGCGCTGCCGGACCTGCAGGT
TCGCTTCGTTCCAGGTATGGCGCTGGACCCGGACGGTGTTAGCACCGCGGTTCGTTTTGCTAAATTCCAGAGCC
AGGGTCTGAAATGGCCGAGCGGCATCACCATGCAGCTGATCGCTTGCCGTCCGCAGTCTACCGGCTCCGTCGG
TCTTAAATCCGCTGACCCGTTTGCGCCGCCGAAACTGTCACCAGGTTACCTGACCGACAAAGACGGTGCTGATC
TGGCTACCCTGCGTAAAGGCATCCATTGGGCACGTGATGTTGCGCGTAGCTCTGCTCTGTCCGAATACCTGGAT
GGTGAGCTGTTCCCAGGTAGCGGCGTTGTTTCTGATGATCAGATCGATGAATATATCCGTCGTTCTATCCACTC
GTCCAACGCTATCACTGGCACCTGTAAAATGGGTAACGCAGGTGACAGCAGCTCTGTGGTAGACAACCAGCTG
CGTGTTCACGGTGTTGAAGGCCTGCGCGTTGTTGACGCTAGCGTTGTTCCGAAAATTCCGGGTGGTCAGACCG
GTGCGCCGGTAGTTATGATCGCTGAACGCGCAGCAGCTCTGCTGACGGGGAAAGCAACCATTGGTGCATCTG
CTGCTGCACCGGCGACCGTAGCTGCATAA-

ASAVEDIRKVLSDSSSPVAGQKYDYILVGGGTAACVLANRLSADGSKRVLVLEAGPDNTSRDVKIPAAITRLFRSPLD
WNLFSELQEQLAERQIYMARGRLLGGSSATNATLYHRGAAGDYDAWGVEGWSSEDVLSWFVQAETNADFGPGA
YHGSGGPMRVENPRYTNKQLHTAFFKAAEEVGLTPNSDFNDWSHDHAGYGTFQVMQDKGTRADMYRQYLKPV
LGRRNLQVLTGAAVTKVNIDQAAGKAQALGVEFSTDGPTGERLSAELAPGGEVIMCAGAVHTPFLLKHSGVGPSAE
LKEFGIPVVSNLAGVGQNLQDQPACLTAAPVKEKYDGIAISDHIYNEKGQIRKRAIASYLLGGRGGLTSTG**A**DRGAFV
RTAGQALPDLQVRFVPGMALDPDGVSTAVRFAKFQSQGLKWPSGITMQLIACRPQSTGSVGLKSADPFAPPKLSP
GYLTDKDGADLATLRKGIHWARDVARSSALSEYLDGELFPGSGVVSDDQIDEYIRRSIHSSNAITGTCKMGNAGDSS
SVVDNQLRVHGVEGLRVVDASVVPKIPGGQTGAPVVMIAERAAALLTGKATIGASAAAPATVAA-

9.8 Y466A/C432S

GCGTCTGCCGTTGAAGACATCCGTAAAGTCCTGTCCGATTCTTCGTCTCCGGTGGCGGGTCAGAAATATGACTA
CATCCTGGTTGGCGGTGGCACCGCGGCGTGCGTGCTGGCAAACCGTCTGAGCGCTGACGGTTCCAAACGTGT
ACTGGTTCTGGAAGCAGGCCCGGATAACACCTCCCGCGACGTTAAGATTCCGGCGGCGATCACCCGCCTGTTC
CGCTCCCCGCTGGACTGGAACCTGTTCTCTGAACTGCAGGAACAGCTTGCGGAACGTCAGATCTACATGGCGC
GTGGCCGTCTGCTGGGCGGTTCCAGCGCGACTAACGCCACTCTGTACCACCGTGGTGCGGCGGGTGATTACGA
CGCATGGGGTGTTGAAGGCTGGTCCAGCGAAGACGTTCTGTCTTGGTTCGTCCAGGCGGAAACCAACGCGGA
CTTCGGTCCGGGCGCTTATCATGGCAGCGGCGGCCCGATGCGTGTGGAAAACCCGCGTTACACCAACAAACA
GCTGCACACTGCTTTCTTCAAGGCTGCTGAAGAAGTTGGTCTTACCCCGAACTCCGATTTCAACGATTGGAGCC
ATGACCACGCCGGTTACGGCACCTTTCAGGTGATGCAGGATAAAGGCACCCGCGCGGATATGTACCGTCAGTA
TCTGAAACCTGTGCTGGGTCGTCGCAACCTGCAGGTACTGACCGGCGCTGCAGTGACCAAAGTCAACATCGAC
CAGGCTGCGGGCAAAGCGCAGGCTCTGGGTGTTGAATTCTCCACCGACGGCCCAACCGGCGAACGCCTGTCT
GCGGAACTGGCTCCGGGTGGTGAGGTCATCATGTGCGCAGGTGCTGTTCACACCCCGTTCCTGCTGAAACATT
CCGGCGTTGGCCCGTCTGCTGAGCTGAAAGAATTCGGCATCCCGGTTGTTAGCAACCTGGCTGGTGTTGGCCA
GAACCTGCAGGATCAGCCGGCGTGCCTGACCGCGGCTCCGGTTAAAGAAAAATACGACGGTATTGCCATTTCT
GATCACATCTACAACGAAAAAGGCCAGATCCGTAAACGTGCAATCGCATCCTACCTGCTGGGTGGTCGTGGCG
GTCTGACTTCCACCGGTAGCGATCGCGGTGCCTTCGTTCGTACCGCGGGTCAGGCGCTGCCGGACCTGCAGGT
TCGCTTCGTTCCAGGTATGGCGCTGGACCCGGACGGTGTTAGCACCGCGGTTCGTTTTGCTAAATTCCAGAGCC
AGGGTCTGAAATGGCCGAGCGGCATCACCATGCAGCTGATCGCTTGCCGTCCGCAGTCTACCGGCTCCGTCGG
TCTTAAATCCGCTGACCCGTTTGCGCCGCCGAAACTGTCACCAGGTTACCTGACCGACAAAGACGGTGCTGATC
TGGCTACCCTGCGTAAAGGCATCCATTGGGCACGTGATGTTGCGCGTAGCTCTGCTCTGTCCGAATACCTGGAT
GGTGAGCTGTTCCCAGGTAGCGGCGTTGTTTCTGATGATCAGATCGATGAATATATCCGTCGTTCTATCCACTC
GTCCAACGCTATCACTGGCACCTGTAAAATGGGTAACGCAGGTGACAGCAGCTCTGTGGTAGACAACCAGCTG
CGTGTTCACGGTGTTGAAGGCCTGCGCGTTGTTGACGCTAGCGTTGTTCCGAAAATTCCGGGTGGTCAGACCG
GTGCGCCGGTAGTTATGATCGCTGAACGCGCAGCAGCTCTGCTGACGGGGAAAGCAACCATTGGTGCATCTG
CTGCTGCACCGGCGACCGTAGCTGCATAA-

ASAVEDIRKVLSDSSSPVAGQKYDYILVGGGTAACVLANRLSADGSKRVLVLEAGPDNTSRDVKIPAAITRLFRSPLD
WNLFSELQEQLAERQIYMARGRLLGGSSATNATLYHRGAAGDYDAWGVEGWSSEDVLSWFVQAETNADFGPGA
YHGSGGPMRVENPRYTNKQLHTAFFKAAEEVGLTPNSDFNDWSHDHAGYGTFQVMQDKGTRADMYRQYLKPV
LGRRNLQVLTGAAVTKVNIDQAAGKAQALGVEFSTDGPTGERLSAELAPGGEVIMCAGAVHTPFLLKHSGVGPSAE
LKEFGIPVVSNLAGVGQNLQDQPACLTAAPVKEKYDGIAISDHIYNEKGQIRKRAIASYLLGGRGGLTSTG**S**DRGAFV
RTAGQALPDLQVRFVPGMALDPDGVSTAVRFAKFQSQGLKWPSGITMQLIACRPQSTGSVGLKSADPFAPPKLSP
GYLTDKDGADLATLRKGIHWARDVARSSALSEYLDGELFPGSGVVSDDQIDEYIRRSIHSSNAITGTCKMGNAGDSS
SVVDNQLRVHGVEGLRVVDASVVPKIPGGQTGAPVVMIAERAAALLTGKATIGASAAAPATVAA-

9.9 Y466A/V453S/G431S

GCGTCTGCCGTTGAAGACATCCGTAAAGTCCTGTCCGATTCTTCGTCTCCGGTGGCGGGTCAGAAATATGACTA
CATCCTGGTTGGCGGTGGCACCGCGGCGTGCGTGCTGGCAAACCGTCTGAGCGCTGACGGTTCCAAACGTGT
ACTGGTTCTGGAAGCAGGCCCGGATAACACCTCCCGCGACGTTAAGATTCCGGCGGCGATCACCCGCCTGTTC
CGCTCCCCGCTGGACTGGAACCTGTTCTCTGAACTGCAGGAACAGCTTGCGGAACGTCAGATCTACATGGCGC
GTGGCCGTCTGCTGGGCGGTTCCAGCGCGACTAACGCCACTCTGTACCACCGTGGTGCGGCGGGTGATTACGA
CGCATGGGGTGTTGAAGGCTGGTCCAGCGAAGACGTTCTGTCTTGGTTCGTCCAGGCGGAAACCAACGCGGA
CTTCGGTCCGGGCGCTTATCATGGCAGCGGCGGCCCGATGCGTGTGGAAAACCCGCGTTACACCAACAAACA
GCTGCACACTGCTTTCTTCAAGGCTGCTGAAGAAGTTGGTCTTACCCCGAACTCCGATTTCAACGATTGGAGCC
ATGACCACGCCGGTTACGGCACCTTTCAGGTGATGCAGGATAAAGGCACCCGCGCGGATATGTACCGTCAGTA
TCTGAAACCTGTGCTGGGTCGTCGCAACCTGCAGGTACTGACCGGCGCTGCAGTGACCAAAGTCAACATCGAC
CAGGCTGCGGGCAAAGCGCAGGCTCTGGGTGTTGAATTCTCCACCGACGGCCCAACCGGCGAACGCCTGTCT
GCGGAACTGGCTCCGGGTGGTGAGGTCATCATGTGCGCAGGTGCTGTTCACACCCCGTTCCTGCTGAAACATT
CCGGCGTTGGCCCGTCTGCTGAGCTGAAAGAATTCGGCATCCCGGTTGTTAGCAACCTGGCTGGTGTTGGCCA
GAACCTGCAGGATCAGCCGGCGTGCCTGACCGCGGCTCCGGTTAAAGAAAAATACGACGGTATTGCCATTTCT
GATCACATCTACAACGAAAAAGGCCAGATCCGTAAACGTGCAATCGCATCCTACCTGCTGGGTGGTCGTGGCG
GTCTGACTTCCACCAGTTGCGATCGCGGTGCCTTCGTTCGTACCGCGGGTCAGGCGCTGCCGGACCTGCAGGT
TCGCTTCAGTCCAGGTATGGCGCTGGACCCGGACGGTGTTAGCACCGCGGTTCGTTTTGCTAAATTCCAGAGC
CAGGGTCTGAAATGGCCGAGCGGCATCACCATGCAGCTGATCGCTTGCCGTCCGCAGTCTACCGGCTCCGTCG
GTCTTAAATCCGCTGACCCGTTTGCGCCGCCGAAACTGTCACCAGGTTACCTGACCGACAAAGACGGTGCTGA
TCTGGCTACCCTGCGTAAAGGCATCCATTGGGCACGTGATGTTGCGCGTAGCTCTGCTCTGTCCGAATACCTGG
ATGGTGAGCTGTTCCCAGGTAGCGGCGTTGTTTCTGATGATCAGATCGATGAATATATCCGTCGTTCTATCCAC
TCGTCCAACGCTATCACTGGCACCTGTAAAATGGGTAACGCAGGTGACAGCAGCTCTGTGGTAGACAACCAGC
TGCGTGTTCACGGTGTTGAAGGCCTGCGCGTTGTTGACGCTAGCGTTGTTCCGAAAATTCCGGGTGGTCAGAC
CGGTGCGCCGGTAGTTATGATCGCTGAACGCGCAGCAGCTCTGCTGACGGGGAAAGCAACCATTGGTGCATC
TGCTGCTGCACCGGCGACCGTAGCTGCATAA

ASAVEDIRKVLSDSSSPVAGQKYDYILVGGGTAACVLANRLSADGSKRVLVLEAGPDNTSRDVKIPAAITRLFRSPLD
WNLFSELQEQLAERQIYMARGRLLGGSSATNATLYHRGAAGDYDAWGVEGWSSEDVLSWFVQAETNADFGPGA
YHGSGGPMRVENPRYTNKQLHTAFFKAAEEVGLTPNSDFNDWSHDHAGYGTFQVMQDKGTRADMYRQYLKPV
LGRRNLQVLTGAAVTKVNIDQAAGKAQALGVEFSTDGPTGERLSAELAPGGEVIMCAGAVHTPFLLKHSGVGPSAE
LKEFGIPVVSNLAGVGQNLQDQPACLTAAPVKEKYDGIAISDHIYNEKGQIRKRAIASYLLGGRGGLTST**S**CDRGAFV
RTAGQALPDLQVRFSPGMALDPDGVSTAVRFAKFQSQGLKWPSGITMQLIACRPQSTGSVGLKSADPFAPPKLSP
GYLTDKDGADLATLRKGIHWARDVARSSALSEYLDGELFPGSGVVSDDQIDEYIRRSIHSSNAITGTCKMGNAGDSS
SVVDNQLRVHGVEGLRVVDASVVPKIPGGQTGAPVVMIAERAAALLTGKATIGASAAAPATVAA-

9.10 Y466A/V453S/G431S/C432A

GCGTCTGCCGTTGAAGACATCCGTAAAGTCCTGTCCGATTCTTCGTCTCCGGTGGCGGGTCAGAAATATGACTA
CATCCTGGTTGGCGGTGGCACCGCGGCGTGCGTGCTGGCAAACCGTCTGAGCGCTGACGGTTCCAAACGTGT
ACTGGTTCTGGAAGCAGGCCCGGATAACACCTCCCGCGACGTTAAGATTCCGGCGGCGATCACCCGCCTGTTC
CGCTCCCCGCTGGACTGGAACCTGTTCTCTGAACTGCAGGAACAGCTTGCGGAACGTCAGATCTACATGGCGC
GTGGCCGTCTGCTGGGCGGTTCCAGCGCGACTAACGCCACTCTGTACCACCGTGGTGCGGCGGGTGATTACGA
CGCATGGGGTGTTGAAGGCTGGTCCAGCGAAGACGTTCTGTCTTGGTTCGTCCAGGCGGAAACCAACGCGGA
CTTCGGTCCGGGCGCTTATCATGGCAGCGGCGGCCCGATGCGTGTGGAAAACCCGCGTTACACCAACAAACA
GCTGCACACTGCTTTCTTCAAGGCTGCTGAAGAAGTTGGTCTTACCCCGAACTCCGATTTCAACGATTGGAGCC
ATGACCACGCCGGTTACGGCACCTTTCAGGTGATGCAGGATAAAGGCACCCGCGCGGATATGTACCGTCAGTA
TCTGAAACCTGTGCTGGGTCGTCGCAACCTGCAGGTACTGACCGGCGCTGCAGTGACCAAAGTCAACATCGAC
CAGGCTGCGGGCAAAGCGCAGGCTCTGGGTGTTGAATTCTCCACCGACGGCCCAACCGGCGAACGCCTGTCT
GCGGAACTGGCTCCGGGTGGTGAGGTCATCATGTGCGCAGGTGCTGTTCACACCCCGTTCCTGCTGAAACATT
CCGGCGTTGGCCCGTCTGCTGAGCTGAAAGAATTCGGCATCCCGGTTGTTAGCAACCTGGCTGGTGTTGGCCA
GAACCTGCAGGATCAGCCGGCGTGCCTGACCGCGGCTCCGGTTAAAGAAAAATACGACGGTATTGCCATTTCT
GATCACATCTACAACGAAAAAGGCCAGATCCGTAAACGTGCAATCGCATCCTACCTGCTGGGTGGTCGTGGCG
GTCTGACTTCCACCAGTGCGGATCGCGGTGCCTTCGTTCGTACCGCGGGTCAGGCGCTGCCGGACCTGCAGGT
TCGCTTCAGTCCAGGTATGGCGCTGGACCCGGACGGTGTTAGCACCGCGGTTCGTTTTGCTAAATTCCAGAGC
CAGGGTCTGAAATGGCCGAGCGGCATCACCATGCAGCTGATCGCTTGCCGTCCGCAGTCTACCGGCTCCGTCG
GTCTTAAATCCGCTGACCCGTTTGCGCCGCCGAAACTGTCACCAGGTTACCTGACCGACAAAGACGGTGCTGA
TCTGGCTACCCTGCGTAAAGGCATCCATTGGGCACGTGATGTTGCGCGTAGCTCTGCTCTGTCCGAATACCTGG
ATGGTGAGCTGTTCCCAGGTAGCGGCGTTGTTTCTGATGATCAGATCGATGAATATATCCGTCGTTCTATCCAC
TCGTCCAACGCTATCACTGGCACCTGTAAAATGGGTAACGCAGGTGACAGCAGCTCTGTGGTAGACAACCAGC
TGCGTGTTCACGGTGTTGAAGGCCTGCGCGTTGTTGACGCTAGCGTTGTTCCGAAAATTCCGGGTGGTCAGAC
CGGTGCGCCGGTAGTTATGATCGCTGAACGCGCAGCAGCTCTGCTGACGGGGAAAGCAACCATTGGTGCATC
TGCTGCTGCACCGGCGACCGTAGCTGCATAA

ASAVEDIRKVLSDSSSPVAGQKYDYILVGGGTAACVLANRLSADGSKRVLVLEAGPDNTSRDVKIPAAITRLFRSPLD
WNLFSELQEQLAERQIYMARGRLLGGSSATNATLYHRGAAGDYDAWGVEGWSSEDVLSWFVQAETNADFGPGA
YHGSGGPMRVENPRYTNKQLHTAFFKAAEEVGLTPNSDFNDWSHDHAGYGTFQVMQDKGTRADMYRQYLKPV
LGRRNLQVLTGAAVTKVNIDQAAGKAQALGVEFSTDGPTGERLSAELAPGGEVIMCAGAVHTPFLLKHSGVGPSAE
LKEFGIPVVSNLAGVGQNLQDQPACLTAAPVKEKYDGIAISDHIYNEKGQIRKRAIASYLLGGRGGLTSTS**A**DRGAFV
RTAGQALPDLQVRFSPGMALDPDGVSTAVRFAKFQSQGLKWPSGITMQLIACRPQSTGSVGLKSADPFAPPKLSP
GYLTDKDGADLATLRKGIHWARDVARSSALSEYLDGELFPGSGVVSDDQIDEYIRRSIHSSNAITGTCKMGNAGDSS
SVVDNQLRVHGVEGLRVVDASVVPKIPGGQTGAPVVMIAERAAALLTGKATIGASAAAPATVAA-

9.11 Y466A/V453S/G431S/I130K

GCGTCTGCCGTTGAAGACATCCGTAAAGTCCTGTCCGATTCTTCGTCTCCGGTGGCGGGTCAGAAATATGACTA
CATCCTGGTTGGCGGTGGCACCGCGGCGTGCGTGCTGGCAAACCGTCTGAGCGCTGACGGTTCCAAACGTGT
ACTGGTTCTGGAAGCAGGCCCGGATAACACCTCCCGCGACGTTAAGATTCCGGCGGCGAAGACCCGCCTGTTC
CGCTCCCCGCTGGACTGGAACCTGTTCTCTGAACTGCAGGAACAGCTTGCGGAACGTCAGATCTACATGGCGC
GTGGCCGTCTGCTGGGCGGTTCCAGCGCGACTAACGCCACTCTGTACCACCGTGGTGCGGCGGGTGATTACGA
CGCATGGGGTGTTGAAGGCTGGTCCAGCGAAGACGTTCTGTCTTGGTTCGTCCAGGCGGAAACCAACGCGGA
CTTCGGTCCGGGCGCTTATCATGGCAGCGGCGGCCCGATGCGTGTGGAAAACCCGCGTTACACCAACAAACA
GCTGCACACTGCTTTCTTCAAGGCTGCTGAAGAAGTTGGTCTTACCCCGAACTCCGATTTCAACGATTGGAGCC
ATGACCACGCCGGTTACGGCACCTTTCAGGTGATGCAGGATAAAGGCACCCGCGCGGATATGTACCGTCAGTA
TCTGAAACCTGTGCTGGGTCGTCGCAACCTGCAGGTACTGACCGGCGCTGCAGTGACCAAAGTCAACATCGAC
CAGGCTGCGGGCAAAGCGCAGGCTCTGGGTGTTGAATTCTCCACCGACGGCCCAACCGGCGAACGCCTGTCT
GCGGAACTGGCTCCGGGTGGTGAGGTCATCATGTGCGCAGGTGCTGTTCACACCCCGTTCCTGCTGAAACATT
CCGGCGTTGGCCCGTCTGCTGAGCTGAAAGAATTCGGCATCCCGGTTGTTAGCAACCTGGCTGGTGTTGGCCA
GAACCTGCAGGATCAGCCGGCGTGCCTGACCGCGGCTCCGGTTAAAGAAAAATACGACGGTATTGCCATTTCT
GATCACATCTACAACGAAAAAGGCCAGATCCGTAAACGTGCAATCGCATCCTACCTGCTGGGTGGTCGTGGCG
GTCTGACTTCCACCAGTTGCGATCGCGGTGCCTTCGTTCGTACCGCGGGTCAGGCGCTGCCGGACCTGCAGGT
TCGCTTCAGTCCAGGTATGGCGCTGGACCCGGACGGTGTTAGCACCGCGGTTCGTTTTGCTAAATTCCAGAGC
CAGGGTCTGAAATGGCCGAGCGGCATCACCATGCAGCTGATCGCTTGCCGTCCGCAGTCTACCGGCTCCGTCG
GTCTTAAATCCGCTGACCCGTTTGCGCCGCCGAAACTGTCACCAGGTTACCTGACCGACAAAGACGGTGCTGA
TCTGGCTACCCTGCGTAAAGGCATCCATTGGGCACGTGATGTTGCGCGTAGCTCTGCTCTGTCCGAATACCTGG
ATGGTGAGCTGTTCCCAGGTAGCGGCGTTGTTTCTGATGATCAGATCGATGAATATATCCGTCGTTCTATCCAC
TCGTCCAACGCTATCACTGGCACCTGTAAAATGGGTAACGCAGGTGACAGCAGCTCTGTGGTAGACAACCAGC
TGCGTGTTCACGGTGTTGAAGGCCTGCGCGTTGTTGACGCTAGCGTTGTTCCGAAAATTCCGGGTGGTCAGAC
CGGTGCGCCGGTAGTTATGATCGCTGAACGCGCAGCAGCTCTGCTGACGGGGAAAGCAACCATTGGTGCATC
TGCTGCTGCACCGGCGACCGTAGCTGCATAA

ASAVEDIRKVLSDSSSPVAGQKYDYILVGGGTAACVLANRLSADGSKRVLVLEAGPDNTSRDVKIPAA**K**TRLFRSPLD
WNLFSELQEQLAERQIYMARGRLLGGSSATNATLYHRGAAGDYDAWGVEGWSSEDVLSWFVQAETNADFGPGA
YHGSGGPMRVENPRYTNKQLHTAFFKAAEEVGLTPNSDFNDWSHDHAGYGTFQVMQDKGTRADMYRQYLKPV
LGRRNLQVLTGAAVTKVNIDQAAGKAQALGVEFSTDGPTGERLSAELAPGGEVIMCAGAVHTPFLLKHSGVGPSAE
LKEFGIPVVSNLAGVGQNLQDQPACLTAAPVKEKYDGIAISDHIYNEKGQIRKRAIASYLLGGRGGLTSTSCDRGAFV
RTAGQALPDLQVRFSPGMALDPDGVSTAVRFAKFQSQGLKWPSGITMQLIACRPQSTGSVGLKSADPFAPPKLSP
GYLTDKDGADLATLRKGIHWARDVARSSALSEYLDGELFPGSGVVSDDQIDEYIRRSIHSSNAITGTCKMGNAGDSS
SVVDNQLRVHGVEGLRVVDASVVPKIPGGQTGAPVVMIAERAAALLTGKATIGASAAAPATVAA-

10 References

(1) Winkler, C. K.; Simić, S.; Jurkaš, V.; Bierbaumer, S.; Schmermund, L.; Poschenrieder, S.; Berger, S. A.; Kulterer, E.; Kourist, R.; Kroutil, W. Accelerated Reaction Engineering of Photo(Bio)Catalytic Reactions through Parallelization with an Open-Source Photoreactor. *ChemPhotoChem* **2021,** *5*, 957-965, DOI: 10.1002/cptc.202100109.

(2) Kille, S.; Acevedo-Rocha, C. G.; Parra, L. P.; Zhang, Z. G.; Opperman, D. J.; Reetz, M. T.; Acevedo, J. P. Reducing Codon Redundancy and Screening Effort of Combinatorial Protein Libraries Created by Saturation Mutagenesis. *ACS Synth. Biol.* **2013,** *2*, 83-92, DOI: 10.1021/sb300037w.

(3) Anandakrishnan, R.; Aguilar, B.; Onufriev, A. V. H++ 3.0: Automating Pk Prediction and the Preparation of Biomolecular Structures for Atomistic Molecular Modeling and Simulations. *Nucleic Acids Res.* **2012,** *40*, W537-541, DOI: 10.1093/nar/gks375.

(4) Gordon, J. C.; Myers, J. B.; Folta, T.; Shoja, V.; Heath, L. S.; Onufriev, A. H++: A Server for Estimating Pkas and Adding Missing Hydrogens to Macromolecules. *Nucleic Acids Res.* **2005,** *33*, W368-371, DOI: 10.1093/nar/gki464.

(5) Myers, J.; Grothaus, G.; Narayanan, S.; Onufriev, A. A Simple Clustering Algorithm Can Be Accurate Enough for Use in Calculations of Pks in Macromolecules. *Proteins* **2006,** *63*, 928-938, DOI: 10.1002/prot.20922.

(6) D.A. Case; H.M. Aktulga; K. Belfon; I.Y. Ben-Shalom; J.T. Berryman; S.R. Brozell; D.S. Cerutti; T.E. Cheatham; G.A. Cisneros; V.W.D. Cruzeiro; T.A. Darden; R.E. Duke; G. Giambasu; M.K. Gilson; H. Gohlke; A.W. Goetz; R. Harris; S. Izadi; S.A. Izmailov; K. Kasavajhala; M.C. Kaymak; E. King; A. Kovalenko; T. Kurtzman; T.S. Lee; S. LeGrand; P. Li; C. Lin; J. Liu; T. Luchko; R. Luo; M. Machado; V. Man; M. Manathunga; K.M. Merz; Y. Miao; O. Mikhailovskii; G. Monard; H. Nguyen; K.A. O'Hearn; A. Onufriev; F. Pan, S.; Pantano; R. Qi; A. Rahnamoun; D.R. Roe; A. Roitberg; C. Sagui; S. Schott-Verdugo; A. Shajan; J. Shen; C.L. Simmerling; N.R. Skrynnikov; J. Smith; J. Swails; Walker, R. C.; J. Wang; J. Wang; H. Wei; R.M. Wolf; X. Wu; Y. Xiong; Y. Xue; D.M. York; S. Zhao; Kollman, P. A. "Amber22", https://ambermd.org, Access Date: 10.01.2026.

(7) Wang, J.; Wolf, R. M.; Caldwell, J. W.; Kollman, P. A.; Case, D. A. Development and Testing of a General Amber Force Field. *J. Comput. Chem.* **2004,** *25*, 1157-1174, DOI: 10.1002/jcc.20035.

(8) Bayly, C. I.; Cieplak, P.; Cornell, W.; Kollman, P. A. A Well-Behaved Electrostatic Potential Based Method Using Charge Restraints for Deriving Atomic Charges: The Resp Model. *J. Phys. Chem.* **1993,** *97*, 10269-10280, DOI: 10.1021/j100142a004.

(9) Frisch, M. J.; Trucks, G. W.; Schlegel, H. B.; Scuseria, G. E.; Robb, M. A.; Cheeseman, J. R.; Scalmani, G.; Barone, V.; Petersson, G. A.; Nakatsuji, H.; Li, X.; Caricato, M.; Marenich, A. V.; Bloino, J.; Janesko, B. G.; Gomperts, R.; Mennucci, B.; Hratchian, H. P.; Ortiz, J. V.; Izmaylov, A. F.; Sonnenberg, J. L.; Williams; Ding, F.; Lipparini, F.; Egidi, F.; Goings, J.; Peng, B.; Petrone, A.; Henderson, T.; Ranasinghe, D.; Zakrzewski, V. G.; Gao, J.; Rega, N.; Zheng, G.; Liang, W.; Hada, M.; Ehara, M.; Toyota, K.; Fukuda, R.; Hasegawa, J.; Ishida, M.; Nakajima, T.; Honda, Y.; Kitao, O.; Nakai, H.; Vreven, T.; Throssell, K.; Montgomery Jr., J. A.; Peralta, J. E.; Ogliaro, F.; Bearpark, M. J.; Heyd, J. J.; Brothers, E. N.; Kudin, K. N.; Staroverov, V. N.; Keith, T. A.; Kobayashi, R.; Normand, J.; Raghavachari, K.; Rendell, A. P.; Burant, J. C.; Iyengar, S. S.; Tomasi, J.; Cossi, M.; Millam, J. M.; Klene, M.; Adamo, C.; Cammi, R.; Ochterski, J. W.; Martin, R. L.; Morokuma, K.; Farkas, O.; Foresman, J. B.; Fox, D. J. *Gaussian 16 Rev. C.01*: Wallingford, CT, 2016.

(10) Besler, B. H.; Merz, K. M.; Kollman, P. A. Atomic Charges Derived from Semiempirical Methods. *J. Comput. Chem.* **1990,** *11*, 431-439, DOI: 10.1002/jcc.540110404.

(11) Singh, U. C.; Kollman, P. A. An Approach to Computing Electrostatic Charges for Molecules. *J. Comput. Chem.* **1984,** *5*, 129-145, DOI: 10.1002/jcc.540050204.

(12) Jorgensen, W. L.; Chandrasekhar, J.; Madura, J. D.; Impey, R. W.; Klein, M. L. Comparison of Simple Potential Functions for Simulating Liquid Water. *J. Chem. Phys.* **1983,** *79*, 926-935, DOI: 10.1063/1.445869.

(13) Miyamoto, S.; Kollman, P. A. Settle: An Analytical Version of the Shake and Rattle Algorithm for Rigid Water Models. *J. Comput. Chem.* **1992,** *13*, 952-962, DOI: 10.1002/jcc.540130805.

(14) Ryckaert, J.-P.; Ciccotti, G.; Berendsen, H. J. C. Numerical Integration of the Cartesian Equations of Motion of a System with Constraints: Molecular Dynamics of N-Alkanes. *J. Comput. Phys.* **1977,** *23*, 327-341, DOI: 10.1016/0021-9991(77)90098-5.

(15) Crowley, M.; Darden, T.; Cheatham, T.; Deerfield, D. Adventures in Improving the Scaling and Accuracy of a Parallel Molecular Dynamics Program. *J. Supercomput.* **1997,** *11*, 255-278, DOI: 10.1023/a:1007907925007.

(16) Darden, T.; York, D.; Pedersen, L. Particle Mesh Ewald: An N·Log(N) Method for Ewald Sums in Large Systems. *J. Chem. Phys.* **1993,** *98*, 10089-10092, DOI: 10.1063/1.464397.

(17) Essmann, U.; Perera, L.; Berkowitz, M. L.; Darden, T.; Lee, H.; Pedersen, L. G. A Smooth Particle Mesh Ewald Method. *J. Chem. Phys.* **1995,** *103*, 8577-8593, DOI: 10.1063/1.470117.

(18) Wu, X.; Brooks, B. R. Self-Guided Langevin Dynamics Simulation Method. *Chem. Phys. Lett.* **2003,** *381*, 512-518, DOI: 10.1016/j.cplett.2003.10.013.

(19) Berendsen, H. J. C.; Postma, J. P. M.; van Gunsteren, W. F.; DiNola, A.; Haak, J. R. Molecular Dynamics with Coupling to an External Bath. *J. Chem. Phys.* **1984,** *81*, 3684-3690, DOI: 10.1063/1.448118.

(20) Roe, D. R.; Cheatham, T. E., 3rd. Ptraj and Cpptraj: Software for Processing and Analysis of Molecular Dynamics Trajectory Data. *J. Chem. Theory Comput.* **2013,** *9*, 3084-3095, DOI: 10.1021/ct400341p.

(21) Chai, J. D.; Head-Gordon, M. Long-Range Corrected Hybrid Density Functionals with Damped Atom-Atom Dispersion Corrections. *Phys. Chem. Chem. Phys.* **2008,** *10*, 6615-6620, DOI: 10.1039/b810189b.

(22) Petersson, G. A.; Al-Laham, M. A. A Complete Basis Set Model Chemistry. II. Open-Shell Systems and the Total Energies of the First-Row Atoms. *J. Chem. Phys.* **1991,** *94*, 6081-6090, DOI: 10.1063/1.460447.

(23) Petersson, G. A.; Bennett, A.; Tensfeldt, T. G.; Al-Laham, M. A.; Shirley, W. A.; Mantzaris, J. A Complete Basis Set Model Chemistry. I. The Total Energies of Closed-Shell Atoms and Hydrides of the First-Row Elements. *J. Chem. Phys.* **1988,** *89*, 2193-2218, DOI: 10.1063/1.455064.

(24) Page, M.; McIver, J. W. On Evaluating the Reaction Path Hamiltonian. *J. Chem. Phys.* **1988,** *88*, 922-935, DOI: 10.1063/1.454172.

(25) Page, M.; Doubleday, C.; McIver, J. W. Following Steepest Descent Reaction Paths. The Use of Higher Energy Derivatives with Ab Initio Electronic Structure Methods. *J. Chem. Phys.* **1990,** *93*, 5634-5642, DOI: 10.1063/1.459634.

(26) Marenich, A. V.; Cramer, C. J.; Truhlar, D. G. Universal Solvation Model Based on Solute Electron Density and on a Continuum Model of the Solvent Defined by the Bulk Dielectric Constant and Atomic Surface Tensions. *J. Phys. Chem. B* **2009,** *113*, 6378-6396, DOI: 10.1021/jp810292n.

(27) Runge, E.; Gross, E. K. U. Density-Functional Theory for Time-Dependent Systems. *Phys. Rev. Lett.* **1984,** *52*, 997-1000, DOI: 10.1103/PhysRevLett.52.997.

(28) ChemCraft "Chemcraft - Graphical Software for Visualization of Quantum Chemistry Computations. Version 1.8, Build 682", https://www.chemcraftprog.com, Access Date: 21.01.2026.

(29) Wilkinson, M. D.; Dumontier, M.; Aalbersberg, I. J.; Appleton, G.; Axton, M.; Baak, A.; Blomberg, N.; Boiten, J. W.; da Silva Santos, L. B.; Bourne, P. E.; Bouwman, J.; Brookes, A. J.; Clark, T.; Crosas, M.; Dillo, I.; Dumon, O.; Edmunds, S.; Evelo, C. T.; Finkers, R.; Gonzalez-Beltran, A.; Gray, A. J.; Groth, P.; Goble, C.; Grethe, J. S.; Heringa, J.; t Hoen, P. A.; Hooft, R.; Kuhn, T.; Kok, R.; Kok, J.; Lusher, S. J.; Martone, M. E.; Mons, A.; Packer, A. L.; Persson, B.; Rocca-Serra, P.; Roos, M.; van Schaik, R.; Sansone, S. A.; Schultes, E.; Sengstag, T.; Slater, T.; Strawn, G.; Swertz, M. A.; Thompson, M.; van der Lei, J.; van Mulligen, E.; Velterop, J.; Waagmeester, A.; Wittenburg, P.; Wolstencroft, K.; Zhao, J.; Mons, B. The Fair Guiding Principles for Scientific Data Management and Stewardship. *Sci. Data* **2016,** *3*, 160018, DOI: 10.1038/sdata.2016.18.

(30) Berga, C.; Garcia-Borràs, M.; Feixas, F. Computational Data for This Study. **2026**, DOI: 10.19061/iochem-bd-4-96.
(31) Suganuma, Y.; Kobayashi, Y. Formation of Cooh-Ylides, and Their Reactivities and Selectivities in Wittig Reactions. *Synlett* **2019,** *30*, 333-337, DOI: 10.1055/s-0037-1611958.
(32) Reid, B. T.; Mailyan, A. K.; Zakarian, A. Total Synthesis of (+)-Guadinomic Acid Via Hydroxyl-Directed Guanidylation. *J. Org. Chem.* **2018,** *83*, 9492-9496, DOI: 10.1021/acs.joc.8b01214.
(33) Li, X.; Yang, X.; Chen, P.; Liu, G. Palladium-Catalyzed Remote Hydro-Oxygenation of Internal Alkenes: An Efficient Access to Primary Alcohols. *J. Am. Chem. Soc.* **2022,** *144*, 22877-22883, DOI: 10.1021/jacs.2c11428.
(34) Tian, J. S.; Xu, S. W.; Bi, Y. H.; Cao, Z. Z.; Loh, T. P. Oxidative Amination of Aldehydes with Amines into α-Amino Ketones. *Org. Lett.* **2023,** *25*, 8922-8926, DOI: 10.1021/acs.orglett.3c03771.
(35) Newcomb, M.; Varick, T. R.; Ha, C.; Manek, M. B.; Yue, X. Picosecond Radical Kinetics - Rate Constants for Reaction of Benzeneselenol with Primary Alkyl Radicals and Calibration of the 6-Cyano-5-Hexenyl Radical Cyclization. *J. Am. Chem. Soc.* **1992,** *114*, 8158-8163, DOI: DOI 10.1021/ja00047a026.
(36) Calvo, B. C.; Madduri, A. V. R.; Harutyunyan, S. R.; Minnaard, A. J. Copper-Catalysed Conjugate Addition of Grignard Reagents to 2-Methylcyclopentenone and Sequential Enolate Alkylation. *Adv. Synth. Catal.* **2014,** *356*, 2061-2069, DOI: 10.1002/adsc.201400085.
(37) da Silva, M. R.; Vidal, D. M.; Figueiredo, L.; Bandeira, P. T.; Bergmann, J.; Zarbin, P. H. G. Tergal Gland Secretion of the Rove Beetle Aleochara Pseudochrysorrhoa (Staphylinidae: Aleocharinae): Chemical Composition and Biological Roles. *Chem Biodivers* **2020,** *17*, e2000483, DOI: 10.1002/cbdv.202000483.
(38) Willand-Charnley, R.; Dussault, P. H. Tandem Application of C-C Bond-Forming Reactions with Reductive Ozonolysis. *J. Org. Chem.* **2013,** *78*, 42-47, DOI: 10.1021/jo3015775.
(39) Zou, Y. L.; Wang, D. R.; Wurst, K.; Kühnel, C.; Reinhardt, I.; Decker, U.; Gurram, V.; Camadanli, S.; Buchmeiser, M. R. Group 4 Dimethylsilylenebisamido Complexes Bearing the 6-[2-(Diethylboryl)Phenyl]Pyrid-2-Yl Motif: Synthesis and Use in Tandem Ring-Opening Metathesis/Vinyl-Insertion Copolymerization of Cyclic Olefins with Ethylene. *Chem. Eur. J.* **2011,** *17*, 13832-13846, DOI: 10.1002/chem.201101829.
(40) Guan, Y.; Shree Sowndarya, S. V.; Gallegos, L. C.; St John, P. C.; Paton, R. S. Real-Time Prediction of (1)H and (13)C Chemical Shifts with Dft Accuracy Using a 3d Graph Neural Network. *Chem. Sci.* **2021,** *12*, 12012-12026, DOI: 10.1039/d1sc03343c.
(41) Abhijeet S. Bhadauria, Y. G. R. S. P. "Cascade - Chemical Shift Calculation with Deep Learning", https://nova.chem.colostate.edu/cascade/predict, Access Date: 10.09.2025.
(42) Adamo, C.; Barone, V. Exchange Functionals with Improved Long-Range Behavior and Adiabatic Connection Methods without Adjustable Parameters: The Mpw and Mpw1pw Models. *J. Chem. Phys.* **1998,** *108*, 664-675, DOI: 10.1063/1.475428.
(43) Simic, S.; Jakstaite, M.; Huck, W. T. S.; Winkler, C. K.; Kroutil, W. Strategies for Transferring Photobiocatalysis to Continuous Flow Exemplified by Photodecarboxylation of Fatty Acids. *ACS Catal.* **2022,** *12*, 14040-14049, DOI: 10.1021/acscatal.2c04444.
(44) Simic, S.; Cespugli, M.; Hetmann, M. C.; Kahler, U.; Jurkas, V.; Di Giacomo, M.; Russo, M. E.; Marzocchella, A.; Gruber, C. C.; Nestl, B. M.; Winkler, C. K.; Kroutil, W. Cavity-Based Discovery of New Fatty Acid Photodecarboxylases. *ChemBioChem* **2024**, e202400631, DOI: 10.1002/cbic.202400631.
